# Nonstoichiometric acid–base reaction as reliable synthetic route to highly stable CH_3_NH_3_PbI_3_ perovskite film

**DOI:** 10.1038/ncomms13503

**Published:** 2016-11-15

**Authors:** Mingzhu Long, Tiankai Zhang, Yang Chai, Chun-Fai Ng, Thomas C. W. Mak, Jianbin Xu, Keyou Yan

**Affiliations:** 1 Department of Electronic Engineering, The Chinese University of Hong Kong, Shatin, New Territories, 999077 Hong Kong, China; 2 Department of Applied Physics, The Hong Kong Polytechnic University, Hung Hom, 999077 Hong Kong, China; 3 Department of Chemistry, The Chinese University of Hong Kong, Shatin, New Territories, 999077 Hong Kong, China

## Abstract

Perovskite solar cells have received worldwide interests due to swiftly improved efficiency but the poor stability of the perovskite component hampers the device fabrication under normal condition. Herein, we develop a reliable nonstoichiometric acid–base reaction route to stable perovskite films by intermediate chemistry and technology. Perovskite thin-film prepared by nonstoichiometric acid–base reaction route is stable for two months with negligible PbI_2_-impurity under ∼65% humidity, whereas other perovskites prepared by traditional methods degrade distinctly after 2 weeks. Route optimization involves the reaction of PbI_2_ with excess HI to generate HPbI_3_, which subsequently undergoes reaction with excess CH_3_NH_2_ to deliver CH_3_NH_3_PbI_3_ thin films. High quality of intermediate HPbI_3_ and CH_3_NH_2_ abundance are two important factors to stable CH_3_NH_3_PbI_3_ perovskite. Excess volatile acid/base not only affords full conversion in nonstoichiometric acid–base reaction route but also permits its facile removal for stoichiometric purification, resulting in average efficiency of 16.1% in forward/reverse scans.

## Abstract (web summary)

The stability of perovskite thin films and devices depends on a number of environmental factors, amongst which humidity. Here, Long *et al*. develop a synthetic route using a nonstoichiometric acid-base reaction to prepare films stable in humid environments for two months.

Solution processed perovskite solar cells (PSC) using AMX_3_ (A=Cs^+^, CH_3_NH_3_^+^ (MA^+^) or NH=HCNH_3_^+^ (FA^+^); M=Sn^2+^, Pb^2+^, Ge^2+^; X=Cl^−^, Br^−^, I^−^) as light absorber have received broad interest in photovoltaics and light-emitting application in the past 5 years due to their ease of fabrication, cost-effectiveness and high efficiency. In terms of photovoltaic performance, it exceeds 22% power conversion efficiency in a short time, which is competitive with long-term developed thin-film-based solar cells (such as CIGS, polycrystalline Si solar cell)^1^,^2^,^3^,^4^,^5^,^6^,^7^,^8^,^9^,^10^. As confirmed, perovskite has a 100–1,000 nm diffusion length in polycrystalline films and over 100 microns diffusion length in single crystals, affording 100% internal quantum efficiency in thin-film-based PSC^11^,^12^,^13^,^14^. In combination with much higher photovoltage and light extinction coefficient, PSC can potentially outperform the efficiency of silicon solar cell in the foreseeable future^11^,^14^,^15^,^16^.

However, as organic–inorganic ionic crystals with intrinsic humidity and thermal instabilities, perovskites encounter difficulties in large-scale application and commercialization. Recently, a layered and/or two-dimensional perovskite as well as pseudo-halide perovskite light absorbers were demonstrated to possess enhanced moisture stability, but unfortunately the efficiency was low (2–8%), probably due to poor electronic properties attributable to their long-chain cation and large-size anion components^17^,^18^,^19^. Besides, a crystal crosslinking strategy was performed with improved stability at ∼55% humidity in the dark, retaining 9–10% *PCE* after 1,000 h under 10% one-sun illumination^20^. Moreover, Chen *et al*.^21^ developed a stable PSC adopting highly doped inorganic charge extraction layer with shielding capacity from moisture and delivered 15% *PCE* with 1 cm^2^ large area. However, the stability of perovskite itself without shielding was still <1 week in a humid environment^20^,^21^. Without device encapsulation, MAPbI_3_/FAPbI_3_ perovskites were regarded to be unstable under high humidity. The degradation kinetics had been studied to some extent using thermal gravity analysis (TGA)^22^ and ultrafast spectroscopy^23^, which revealed some possible routes, but the degradation process related to transition states and intermediate products is not well-resolved. For example, the sequence of HI and CH_3_NH_2_ release was ambiguous and some believed that HI was released before CH_3_NH_2_ (ref. 24). Besides, CH_3_NH_3_I was very thermally stable even when the temperature increased to 150 °C (refs 22, 25), but the perovskites quickly decayed to PbI_2_ in association with CH_3_NH_2_/HI release at 80–150 °C after 24 h, which meant the decomposition kinetic pathways were different from each other^26^. Thus, this degradation kinetics of perovskite requires a closer examination to address these stability issues, and an alternative strategy is expected for enhancement of perovskite stability while keeping its high performance.

In this work, we report our investigation of the degradation and recovery of CH_3_NH_3_PbI_3_ perovskite, which revealed the role of the intermediate product as well as methylamine amount in achieving stability. Hybrid perovskite decomposes sequentially in terms of thermodynamics based on MA-recoverable degradation, such that it first loses CH_3_NH_2_ and then HI, leaving behind PbI_2_ solid. On the basis of this, we have developed an alternative two-step nonstoichiometric acid–base reaction route (NABR) for the synthesis of moisture-resistive perovskite, that is the production of starting HPbI_3_ using excess HI to react with PbI_2_, followed by perovskite conversion from HPbI_3_ using excess CH_3_NH_2_. We have established that CH_3_NH_2_ abundance in synthesis and the formation of high-quality HPbI_3_ built of columnar face-sharing PbI_6_ octahedra are two key parameters for stabilized perovskite. In NABR, excess and volatile reagents lead to complete reaction as well as stoichiometry, respectively. Therefore, a perovskite thin-film prepared via this route possesses reduced lattice vacancy, thus eliminating the penetration of undesirable H_2_O molecules into vacant sites and avoiding formation of monohydrate degradation product in association with H-bonding between H_2_O and MA^+^. We have demonstrated that the CH_3_NH_3_PbI_3_ perovskite thin-film remained highly stable in ∼65% humidity for up to 2 months with negligible PbI_2_-impurity, whereas other perovskites prepared by traditional one-step or two-step methods degrade distinctly after 2 weeks. The PSC using thin-film after humidity exposure delivered even better efficiency than that from freshly prepared film probably due to slight doping. This work provides an important insight into perovskite intrinsic stability and the utilization of simple chemical reaction for material control in PSC.

## Results

### Recoverable degradation

**Figure 1:**
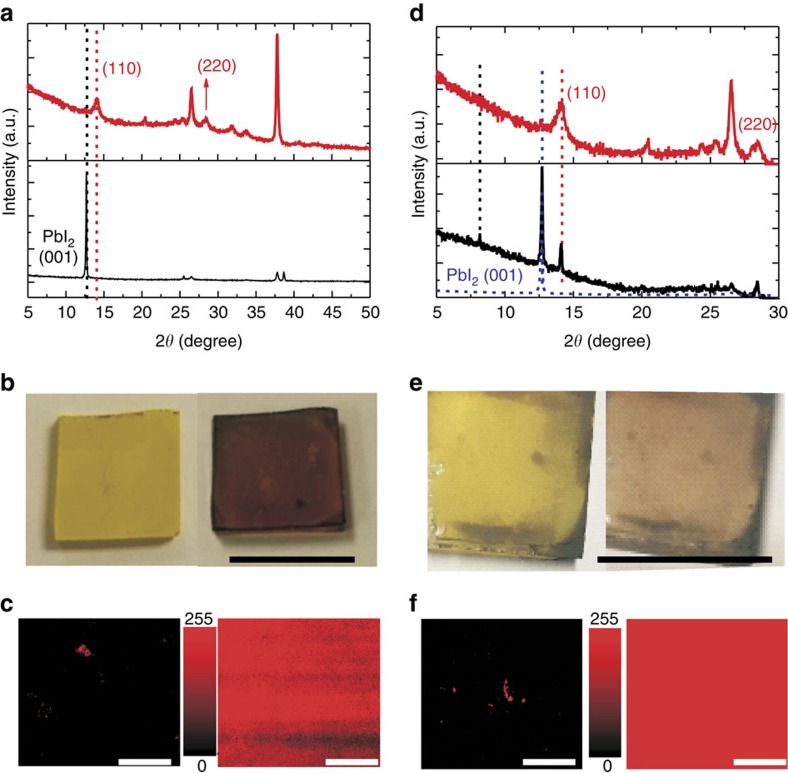
Recoverable perovskite degradation. (**a**) X-ray diffraction patterns of degraded (black line) and recovered film (red line) prepared by traditional two-step method. (**b**) Photographs and (**c**) photoluminescence mapping of degraded perovskite film (left) and recovered perovskite film (right), for a thin-film prepared by two-step method, with the colour bar in the middle representing photoluminescence in counts. (**d**) X-ray diffraction patterns of degraded films under 3 days moisture (black line) and 21 days moisture (blue dash line) and recovered film (red line) for a thin-film prepared by traditional one-step method. (**e**) Photographs and (**f**) photoluminescence mapping of degraded perovskite film (left) and recovered perovskite film (right) prepared by one-step method, with the colour bar in the middle representing photoluminescence in counts. Scale bar, 1.5 cm (**b**,**e**), 50 μm (**c**,**f**).

To find a way to improve the stability of CH_3_NH_3_PbI_3_, we have first investigated the degradation of traditional perovskites and used the recent defect-healing process for the recovery of degraded perovskite to check the transition products^27^. Figure 1a–c show the X-ray diffraction, optical images and photoluminescence mapping for degraded and recovered perovskite thin-film (prepared by traditional two-step method). We found that after degradation for some time, perovskite became yellow (Fig. 1b left) and had the distinct X-ray diffraction pattern of PbI_2_ at 2*θ*=12.6° (Fig. 1a). Although we thought it was fully degraded, it was immediately recovered to great extent by exposure to CH_3_NH_2_ vapour (Fig. 1b right). The CH_3_NH_2_-recovered perovskite film was confirmed by the X-ray diffraction peaks (Fig. 1a) at 2*θ*=14.1°, 28.4° that were indicative of CH_3_NH_3_PbI_3_ (110), (220) facets. The recovered perovskite films existed in the form of nanoscale crystals judging from the broad X-ray diffraction peaks. The photoluminescence mapping contrasts (Fig. 1c) for the degraded (left) and recovered (right) films indicated their uniform reversion to perovskite. In comparison, the degraded film prepared by traditional one-step method was used for recovery test. These films had fast degradation rates and yielded some transition product under observation during the degradation. Figure 1d–f show the basic results. After 3-day degradation in 65% humidity, we observed a small-angle X-ray diffraction peak at 2*θ*=8.1°, which was identical to that in the monohydrate (CH_3_NH_3_PbI_3_˙H_2_O, with X-ray diffraction peaks at 2*θ*=8.10°, 8.66° and 10.66°) (refs 23, 25, 28). After 3 weeks, the degraded film had only one PbI_2_ peak at 2*θ*=12.6°. However, these films were partially recovered as perovskite nanoscale crystals (Fig. 1d), with photoluminescence mapping so as to confirm the uniform recovery judged from the strong photoluminescence at 760 nm (ref. 29; Fig. 1f). The similar process of degradation and recovery was also observed in the mixed CH_3_NH_3_PbI_3−*x*_Cl_*x*_ perovskite using 3:1 mole combination of CH_3_NH_3_I and PbCl_2_, which was high-performing in PSC but presumably the most unstable perovskite compared with the iodide perovskite. We saw that the degradation process clearly exhibited the transition monohydrate product of CH_3_NH_3_PbI_3−*x*_Cl_*x*_·H_2_O in the degradation, as shown in small angles of X-ray diffraction at 2*θ*=8.10°, 8.66° and 10.66°, corresponding to the (001), (100) and 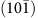 reflections of a monoclinic P21/*m* crystal structure, and could be recovered to some extent (Supplementary Fig. 1a). The monohydrate is similar to CH_3_NH_3_PbI_3_˙DMF (dimethylformamide), in which the MA^+^ is connected with solvent molecules (H_2_O/DMF) through H-bonding (Supplementary Fig. 1b,c) and thus degradation reaction occurs.

To evaluate the reaction process of perovskite more clearly, large crystals were prepared by immersing PbI_2_ into 2–5 mg ml^−1^ MAI IPA solution for *in situ* observation of the recovery process via optical microscope. We found that after degradation, the yellow phase crystals did not differ much in morphology from that of the parent black perovskite (Supplementary Fig. 2). After recovery using CH_3_NH_2_, the degraded crystals re-crystallized as much smaller and more compact grains (Supplementary Fig. 3). This general recovery suggested that the degraded film with PbI_2_ and monohydrate could be re-converted to perovskite using methylamine and thus inferred that degradation of perovskite was also related to the loss of methylamine.

We have analysed the chemical reactions for perovskite formation in DMF solution and degradation with the following scheme:


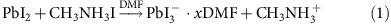



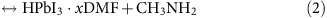



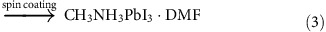



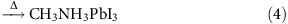


On the basis of previous work^25^,^28^, we observed the colloidal characteristics and redshift of perovskite precursor compared with PbI_2_, which verified that either (1) or (2) was right, which could yield CH_3_NH_3_PbI_3_˙DMF(3). The intermediate CH_3_NH_3_PbI_3_˙DMF (Supplementary Fig. 1c,d) has been detected by X-ray diffraction in another report^28^ and will be confirmed in the following. Noted that these routes could yield possible byproducts shown below due to coordination of DMF to Pb(II) in the solution accompanied by its subsequent removal in the film:


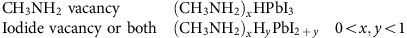


In the degradation, we could simply consider the following step reactions:


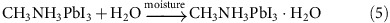



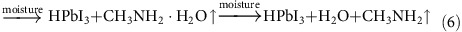



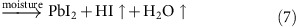


Actually, reaction (5) has been verified through *in situ* time-resolved X-ray diffraction techniques and heating-recovery in a previous study and reaction (7) can be easily concluded from the final products. Degradation reaction (6) was apparently judged from CH_3_NH_2_-recovery in the glovebox (Fig. 1 and Supplementary Fig. 3) and directly proved in the following investigation. Therefore, we conclude that in the humidity degradation, the perovskite is sequentially decomposed in terms of thermodynamics, first forming an intermediate monohydrate (5), then liberating CH_3_NH_2_ molecules (6), and finally yielding a PbI_2_ solid and releasing HI/H_2_O vapour (7), although the kinetic pathways could produce different final products (for example, CH_3_NH_3_PbI_3_˙H_2_O, PbI_2_ and HPbI_3_, and so on)^23^,^30^. Hence, we can make two conclusions. First, from the formation and degradation analysis, we can see that the formation of a fully coordinated perovskite [PbI_3_]^−^ framework is the first important parameter, which can reduce the I-vacancy. Second, as CH_3_NH_2_ can recover the degraded perovskite through the reverse reactions of (5–7), CH_3_NH_2_ abundance is able to improve the stability of perovskite through retarding the degradation reaction. Therefore, the key to stable perovskite is to build a fully coordinated and robust [PbI_3_]^−^ scaffold that accommodates CH_3_NH_2_ at A sites of perovskite AMX_3_ lattice, thus eliminating the inclusion of undesirable water molecules and suppressing the degradation reaction.

### Acid–base reaction

To synthesize stable perovskite, we propose a two-step route through NABR based on the above analysis where *Δ*_r_*G*_1_ and *Δ*_r_*G*_2_ are the changes of Gibbs free-energy for reaction (8) and (9), respectively.


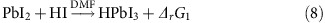



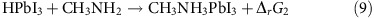


In reaction (8), excess hydriodic acid promotes the reaction completely and yields HPbI_3_ that is stoichiometrically identical to the [PbI_3_]^−^ framework of perovskite, permitting it to form perovskite without I-vacancy. In reaction (9), excess CH_3_NH_2_ allows complete conversion of HPbI_3_ to perovskite and thus eliminates the CH_3_NH_2_ vacancy. Different from the use of excess CH_3_NH_3_I to react with PbI_2_, both CH_3_NH_2_ and HI are facile for removal to yield vacancy-free perovskite due to their volatility at room temperature.

Faint yellow HPbI_3_ crystal shows a hexagonal array of [PbI_3_]^−^ anionic columns each composing of [PbI_6_] coordination octahedra that are stacked along their opposite triangular facets, with exact molecular formula HPbI_3_. Presumably, the protons in HPbI_3_ can move freely in the intervening space between hexagonal arrays of anionic coordination columns. Powdery HPbI_3_ as starting material for device fabrication was prepared following the literature procedure^31^, with careful modification using ethanol instead of diethyl ether to remove excess HI and intercalated solvents (H_2_O, DMF) and precipitate our products. (see the Methods section and Supplementary Fig. 4).

**Figure 2:**
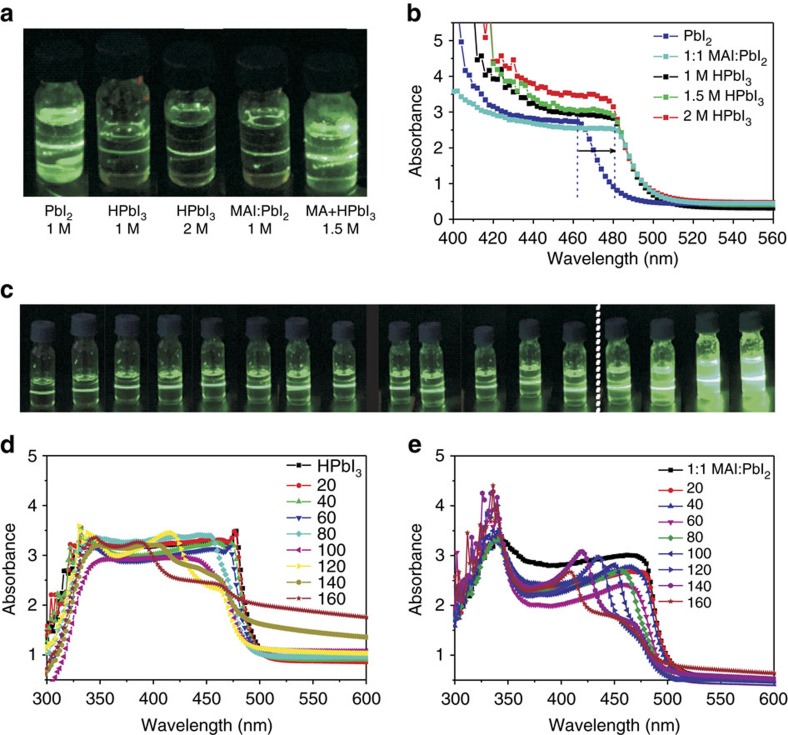
Acid–base reacted colloidal solution. (**a**) Tyndall effect using 532 nm laser suggests the colloidal behaviour of starting 1.0 M PbI_2_ and 1.0 M, 2.0 M HPbI_3_, control 1:1 combination of 1 M CH_3_NH_3_I:PbI_2_ precursor and acid–base precursors prepared via adding 0.2 ml CH_3_NH_2_ ethanol solution into 0.5 ml of 1.5 M HPbI_3_ solution. (**b**) Ultraviolet spectra of starting PbI_2_ and HPbI_3_ precursors, as well as control 1:1 combination of 1 M CH_3_NH_3_I:PbI_2_ precursor. Array indicates redshift after full iodine coordination. (**c**) Colloidal variation of the acid–base precursors prepared by stepwise addition of 25-μl CH_3_NH_2_ ethanol solution into 0.5 ml of 2M HPbI_3_ (from left to right, 0 to 400 μl, white dash line lies after 300 μl). (**d**) Absorption spectrum variation of the acid–base precursors prepared by stepwise addition of 20-μl CH_3_NH_2_/ethanol solution into 0.3 ml of 1 M HPbI_3_. (**e**) Absorption spectrum variation of 0.3 ml of 1 M control 1:1 CH_3_NH_3_I:PbI_2_ precursors through stepwise addition of 20-μl CH_3_NH_2_/ethanol solution. The spikes at around 335 nm are caused by the precipitate after adding too much CH_3_NH_2_/ethanol solution.

All the lead polyiodide precursors in DMF exhibited colloidal behaviour judging from their Tyndall effects using 532 nm laser beam (Fig. 2a). PbI_2_ was coordinated by DMF ligands for dissolution and formed colloids in high concentration^25^. Precursors of both HPbI_3_ and 1:1 combination of CH_3_NH_3_I:PbI_2_ had smaller colloidal size judging from weaker light scattering than that of PbI_2_ due to increased coordination number of Pb(II) (Fig. 2a). Therefore, HPbI_3_ had the advantage of solubility in much higher concentration (>2.5 M) than PbI_2_. We compared the absorption spectra of HPbI_3_, PbI_2_ and 1:1 CH_3_NH_3_I:PbI_2_ (one-step precursor) and found that those of HPbI_3_ and 1:1 CH_3_NH_3_I:PbI_2_ had 20–30 nm red-shifted absorption edge compared with that of PbI_2_, suggesting that coordination occurred between iodide and PbI_2_ in both HPbI_3_ and the 1:1 complex CH_3_NH_3_I:PbI_2_ (Fig. 2b). Moreover, the absorption of HPbI_3_ solution was slightly red-shifted with respect to that of 1:1 CH_3_NH_3_I:PbI_2_ solution, indicating complete coordination due to the use of excess HI. In the NABR precursors, the addition of too much CH_3_NH_2_ ethanol solution decreased the solubility in the mixed solution and precipitated into some solid phase (Fig. 2c).The increased beam size and distinct light scattering effects were monitored, which were used for optimizing organic–inorganic ratios of CH_3_NH_2_:HPbI_3_ (ref. 25). Due to acid–base neutralization (reaction (9)), CH_3_NH_2_ could be spontaneously arranged in the HPbI_3_ framework. With increasing addition of CH_3_NH_2_ solution, the absorption began to exhibit a blue-shift (Fig. 2d). This blue-shift was actually the bleaching state of perovskite compound with excess CH_3_NH_2_ intercalation inside, which thus did confirm that the formation towards perovskite configuration started in the solution and could be readily converted to perovskite after DMF removal^24^,^32^. As control experiment, we also added CH_3_NH_2_ solution into a 1:1 molar solution of CH_3_NH_3_I:PbI_2_. We observed not only the blue-shift suggesting CH_3_NH_2_ intercalation but also the strong absorption peak at 335 nm indicative of PbI_2_ monomer, which meant it lacked iodine coordination in this recipe^33^ (Fig. 2e). This iodine deficiency was consistent with a slightly bluer absorption edge than aforementioned HPbI_3_ solution, which would lead to iodine vacancy by the control method.

### Crystalline phase conversion

**Figure 3:**
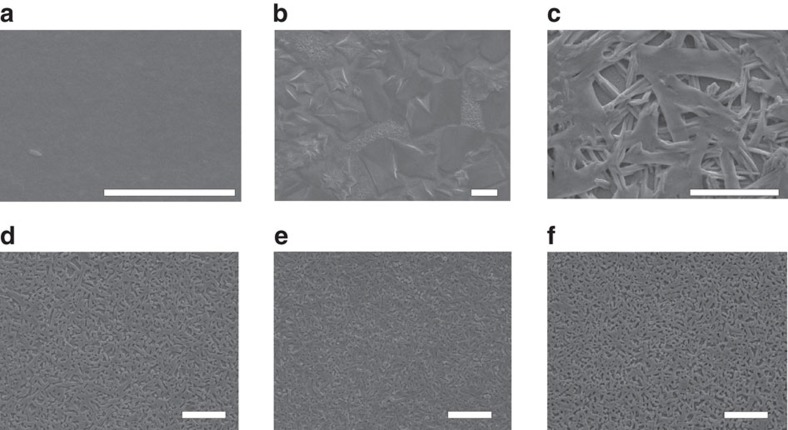
SEM of the thin films. (**a**) The PbI_2_ film. (**b**) HPbI_3_ film. (**c**) Controlled perovskite film by one-step method. (**d**–**f**) NABR prepared perovskite films with increasing amounts of CH_3_NH_2_: 0.3 ml (**d**), 0.35 ml (**e**) and 0.4 ml (**f**) MA in 1.5 M 1 ml HPbI_3_. Scale bar, 5 μm (**a**), 10 μm (**b**–**f**).

We first checked the morphology of thin films (Fig. 3). PbI_2_ film prepared from DMF solution did not show the anisotropic rod-shape of its powders that should be essentially in accordance with the trigonal phase, but afforded uniform coverage and flatness on the substrates due to DMF coordination (Fig. 3a). HPbI_3_ deposited as large-sized bundle-like crystals from solution by spin-coating, with poor coverage in film formation due to Ostwald ripening (Fig. 3b). In the control method, 1:1 combination of CH_3_NH_3_I:PbI_2_ produced dendritic bundles of perovskite (Fig. 3c), which is related to CH_3_NH_3_PbI_3_˙DMF solvate and will be discussed later. In NABR, perovskites precipitated in the similar morphology to that from the control method due to similar iodide coordination (Fig. 3d–f). However, CH_3_NH_2_ was able to form a liquid interface (HPbI_3_˙*x*(CH_3_NH_2_)) with perovskite^27^ through intercalation/coordination and thus served as surfactants to refine and passivate the grain. Therefore, the increased CH_3_NH_2_ in NABR could reduce the crystal size (Fig. 3e,f) and improve film coverage.

**Figure 4:**
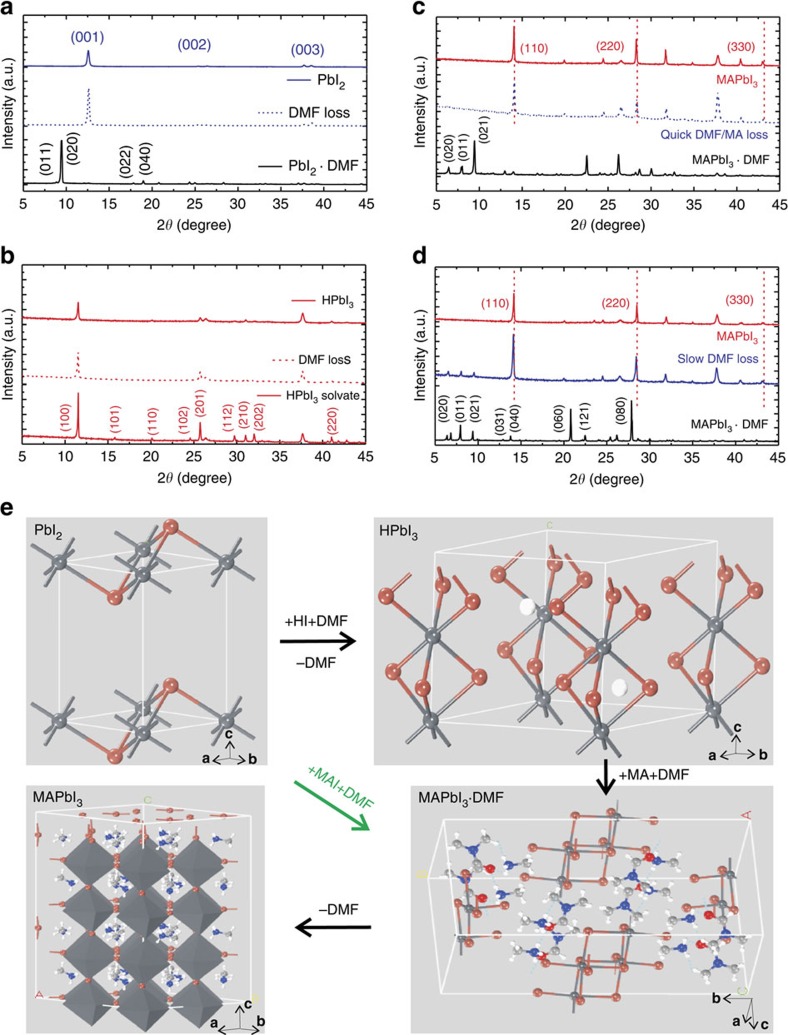
Crystalline phase conversion analysis. (**a**–**d**) X-ray diffraction patterns of PbI_2_ film (**a**), HPbI_3_ film (**b**), CH_3_NH_3_PbI_3_ films by NABR (**c**) and control one-step method (**d**) at different reaction stages represented by dip-coated wet film (bottom curve), spin-coated films before baking (middle curve) and after baking (top curve). (**e**) Crystallographic illustration between NABR conversion from PbI_2_, to HPbI_3_ (H^+^ ions are included to indicate stoichiometry but are actually mobile around [PbI_3_]^−^ column), then to intermediate CH_3_NH_3_PbI_3_·DMF, finally to CH_3_NH_3_PbI_3_ and conventional conversion from MAI+PbI_2_ using DMF as solvent, to CH_3_NH_3_PbI_3_·DMF, then to CH_3_NH_3_PbI_3_.

We have correlated the morphology with crystallographic information in detail through X-ray diffraction monitoring the conversion (Fig. 4). Dip-coated wet film, spin-coated films before baking and after baking are deposited to represent the three basic conversion stages. The wet PbI_2_ film had small-angle peak at 2*θ*=9.5°, which is due to ligand behaviour of DMF (PbI_2_˙DMF; Fig. 4a black curve) that is similar to that of dimethyl sulfoxide (DMSO) in PbI_2_˙DMSO^9^. This soft coordination complex facilitated film formation of PbI_2_ as aforementioned through gradual release of coordinated DMF during spin-coating without baking (Fig. 4a blue dashed curve). Baking increased the crystallinity from the strong (001) peak at 2*θ*=12.6° (Fig. 4a blue solid curve). HPbI_3_ film displayed thin-film X-ray diffraction peaks at 2*θ*=11.5°, 15.8°, 20.1°, 25.8°, corresponding to (100), (101), (110) and (201) facets of hexagonal HPbI_3_, respectively. Even when the film was a little wet, it displayed the same indexed X-ray diffraction pattern as the dry films without/with baking after DMF loss, which is consistent with the single-crystal X-ray diffraction analysis on their solvate and dry crystal (Fig. 4b and Supplementary Fig. 4). The transition products of perovskites were recognized by their small-angle X-ray diffraction peaks in wet films at 2*θ*=6.5°, 7.9° and 9.4°, corresponding to monoclinic CH_3_NH_3_PbI_3_˙DMF (also see Supplementary Fig. 1c,d). However, in the spin-coated films before baking, these small-angle peaks of NABR film disappeared, suggesting its complete conversion to perovskite (Fig. 4c), but the control one-step sample still showed such peaks, which meant that DMF was still incorporated in the lattice (Fig. 4d). This information suggests that there exist iodine/CH_3_NH_2_ vacant sites with larger binding energy in the control sample and thus DMF is not easily removed at room temperature. NABR method reduces the vacancies owing to full coordination of HI and CH_3_NH_2_ abundance. Namely, DMF molecules just play the role in solvation and/or intercalation through H-bonding to MA but are not directly coordinated to lead ions in NABR (Supplementary Fig. 1c). Hence, NABR facilitates quick transformation towards perovskite and affords crystalline perovskite even without baking (Fig. 4c and Supplementary Fig. 5a). Strong Bragg peaks of perovskite were observed at 14.08°, 28.41° and 43.19° corresponding to the (110), (220) and (330) facets, respectively (Fig. 4c).

Figure 4e schematically illustrates the whole reaction process in conformity with crystallographic information. The Pb(II) centre in PbI_2_ is coordinated by DMF, forming PbI_2_˙DMF after spin-coating^28^. In the first-step of acid–base reaction, HI replaces DMF for direct coordination to Pb(II) in forming linear columns each composed of stacked face-sharing PbI_6_ octahedra, which are further arranged to give a hexagonal array in HPbI_3_ with the aid of DMF intercalation between them. In the second step, CH_3_NH_2_/DMF is inserted into the inter-columnar region of HPbI_3_, forming CH_3_NH_3_PbI_3_˙DMF at first. The film morphology of perovskite is determined by the preformed monoclinic CH_3_NH_3_PbI_3_˙DMF containing [PbI_3_]^−^ double chains that is thus needle-like (see Supplementary Fig. 1c). The CH_3_NH_3_PbI_3_˙DMF opens its [PbI_3_]^−^ double chain after DMF removal and undergoes transformation to the tetragonal perovskite structure. In traditional route, stoichiometric MAI/PbI_2_ cannot ensure full iodine coordination for stoichiometric perovskite due to coordination competition by DMF at X sites.

Perovskite prepared by NABR has strong absorption in the green and weak absorption in the red, which is a feature of high-quality perovskite. The one-step method produces a weak flat light absorption spectrum probably due to the defect absorption^34^ (Supplementary Fig. 5b). Hexagonal HPbI_3_ has an absorption edge at 420 nm, different from the theoretical 0.3 eV band gap in its cubic perovskite phase^24^, like FAPbI_3_ with yellow and black phases^35^.

### Intrinsic stability of as-prepared films

In principle, PbI_2_ component is difficult to dissolve in water and thus the unstable component is the organic CH_3_NH_3_I counterpart. In general, a 2D perovskite bearing a long alkyl chain for shielding from moisture has much higher stability. For CH_3_NH_2_, the −CH_3_ group is hydrophobic and thus good humidity stability could be expected if -NH_3_ is well bonded to inorganic [PbI_3_] framework.

**Figure 5:**
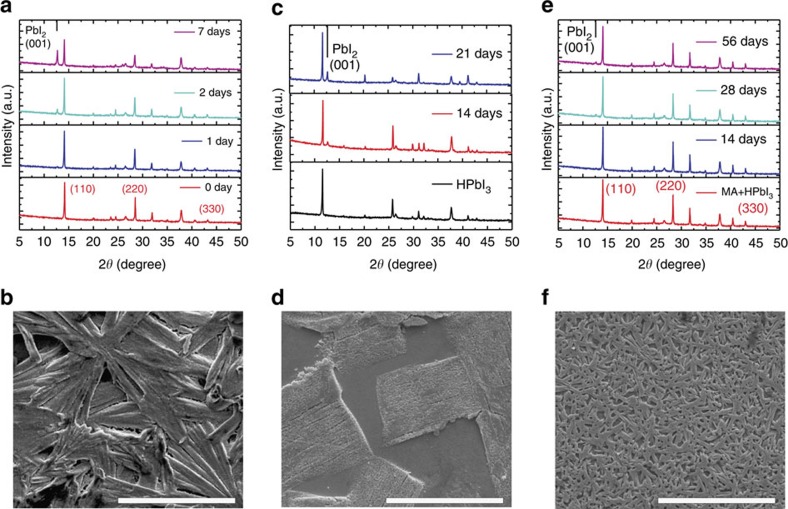
Humidity stability of as-prepared film. (**a**) X-ray diffraction patterns of control perovskite film before and during degradation. (**b**) SEM micrograph of control perovskite film after 7 days degradation. (**c**) X-ray diffraction patterns of HPbI_3_ before and during degradation. (**d**) SEM micrograph of HPbI_3_ after 21 days degradation. (**e**) X-ray diffraction patterns of perovskite by NABR before and during degradation. (**f**) SEM micrograph of perovskite by NABR after 56 days degradation. Notes: the films were deposited on FTO glass and exposed to 65% humidity in a cabinet containing water in a beaker. Scale bar, 30 μm.

We have monitored the degradation of these perovskites (Fig. 5) in ∼65% humidity and found that the control perovskite prepared by the one-step method was severely jeopardized by moisture after 1 week (Fig. 5a). The final products contained large amounts of PbI_2_ as evidenced by PbI_2_ (001) peak at 12.6° (Fig. 5a), together with amorphous solvate judging from the morphology (Fig. 5b). The perovskite prepared by traditional two-step method change to yellow for about 2 weeks under the same condition (Supplementary Fig. 3e), and mixed CH_3_NH_3_PbI_3−*x*_Cl_*x*_ perovskite degraded quickly within 1 h in such humidity to CH_3_NH_3_PbI_3−*x*_Cl_*x*_˙H_2_O (Supplementary Fig. 1).

We have also checked the stability of starting HPbI_3_. HPbI_3_ displayed much better stability than traditional perovskites after 3 weeks, judging from the latter appearance of PbI_2_ (001) peak (Fig. 5c). Except for some erosion traces on the surface, we did not observe too much change in SEM (Fig. 5d). It has to be mentioned that the HPbI_3_ directly precipitated by adding anti-solvents (such as diethyl ether) does not endure high humidity, probably due to HI/H_2_O residues.

The perovskite prepared by NABR had robust stability after optimizing the amount of CH_3_NH_2_ in this work. The best film remained stable for about 2 months in ∼65% humidity, without distinct PbI_2_-impurity from X-ray diffraction pattern and significant morphology change from freshly prepared samples (Fig. 5e,f). However, we found that stability was CH_3_NH_2_-amount-dependent (Supplementary Figs 6–12). The CH_3_NH_2_ used should be largely in excess in NABR, and CH_3_NH_2_ deficiency leads to incomplete conversion of HPbI_3_ with poor stability (Supplementary Fig. 6). Therefore, we confirm that HPbI_3_ permits the formation of a stable well-defined perovskite framework, and excess CH_3_NH_2_ ensures sufficient filling in the lattice and surface passivation to resist H_2_O erosion in combination.

We have further performed TGA to check the thermal stability of perovskite films and starting materials. TGA curve for CH_3_NH_3_I shows nearly 100% weight loss between 260 and 320 °C (Supplementary Fig. 13). HPbI_3_ has a large weight loss at a temperature range between 300 and 360 °C, which is indicative of the release of HI. Consistent with its low humidity stability, the perovskite prepared by one-step method was also not thermally stable. Weight loss onset occurred at 60 °C, which was consistent with a previous report^22^ and meant that the organic–inorganic components were not tightly bounded. Through careful observation, the sequential thermal decomposition mechanism could be identified for control perovskite (see reaction 10) judging from two different weight loss regions at 60–150 °C and 250–350 °C, with the similar weight loss at 250–350 °C to HPbI_3_. For NABR, the weight loss of the organic component was much larger than the others, being indicative of a fully coordinated [PbI_3_]^−^ scaffold with sufficient CH_3_NH_2_ filling in the perovskite lattice.


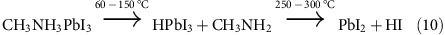


Probably, perovskite prepared by the two-step method and NABR also decomposed sequentially under heating stress in terms of thermodynamics. Due to the similar release rates of MA and HI in kinetic pathways, we were unable to detect sequential events. In principle, the sequential decomposition thermodynamics is acceptable because when iodide is well coordinated to the metal centre of PbI_2_, and then the bind energy of MAI is reduced, leading to releasing MA easily.

TGA was performed in the low-temperature region for release of the organic component, with 30 and 120 min heat preservation at 100 and 200 °C for clear observation of weight loss, respectively. We can see the better stability in NABR after optimization (Supplementary Fig. 13b). Besides, NABR affords to endure high-temperature calcination below 150 °C for high-quality films with negligible impurity (Supplementary Fig. 14). Therefore, NABR represents a controllable way towards the preparation of highly stable perovskite in humid environment and under heat stress.

### Film optimization and stability check

**Figure 6:**
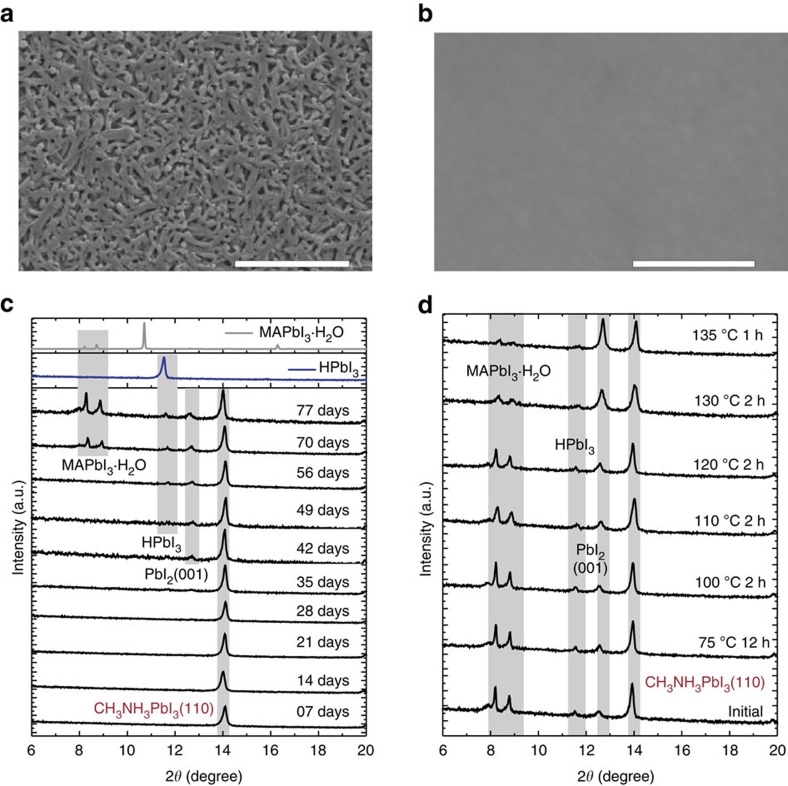
Pin-hole-free film and stability. (**a**,**b**) Perovskite film coverage using nucleation agent (NA) (**b**) and without NA (**a**) by NABR. Scale bars,10 μm (**a**) and 0.5 μm (**b**). (**c**) Further stability check for optimized film with even longer exposure times over 2 months. (**d**) Reversible test of degraded film through heating.

We have introduced dripping of nucleation agent during spin-coating, which enhanced the film coverage for photovoltaics. The nucleation agent made of anti-solvent increased the heterogeneous nucleation sites and reduced the height of the free-energy barrier (*ΔG*) for nucleation. Therefore, it accelerated nucleation exponentially according to classical nucleation theory (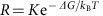, *R*, nucleation rate; *K*, constant; *k*_B_*T* thermal energy) and suppressed the Ostwald ripening in sequential nucleation, resulting in 100% film coverage by this key step (Fig. 6a,b, and Supplementary Figs 15–17). Through nucleation control, we could obtain pin-hole-free thin-film with optimization (Fig. 6b).

We have found that optimized perovskite thin-film with nucleation agent remained stable through colour and X-ray diffraction monitoring (Fig. 6c). Under ∼65% humidity, there was no signature of PbI_2_-impurity after 1 month of exposure and negligible PbI_2_ (∼7%) from X-ray diffraction monitoring. However, degradation beyond 2 months of the pin-hole-free film was quite different from that of the mesoporous as-prepared film without nucleation agent. We found a plausible trace of HPbI_3_ after 7 weeks at 2*θ*=11.6°, and then peaks at 2*θ*=8.1° and 8.7° indicating (001), (100) facets of CH_3_NH_3_PbI_3_˙H_2_O after 9 weeks, respectively. This can be explained by the sequential degradation thermodynamics in combination with degradation kinetics if we consider the surface effect. On the surface of pin-hole-free film, due to the ready release of both HI and CH_3_NH_2_, the sequential degradation reaction (5–7) occurred nearly simultaneously and thus only yielded PbI_2_ that we could observe. When degradation went to the interior that was single crystal-like^36^, CH_3_NH_2_ and HI were difficult to be released from the interior of high-quality of the film, thus as formed internal HPbI_3_ and CH_3_NH_3_PbI_3_˙H_2_O were detected by X-ray diffraction patterns after long-time degradation. This explanation has been further tested by the following experiments. First, we tried to convert the CH_3_NH_3_PbI_3_˙H_2_O back to CH_3_NH_3_PbI_3_ through heating (Fig. 6d). To our surprise, after 12 h heating at 75 °C and 2 h heating at 100 °C, we found there was no observable change of the monohydrated phase. We then increased the calcination temperature gradually and found a change happened at above 110 °C, which was actually the decomposition threshold of defective perovskite. Second, we also checked the morphology after 70 days degradation and found that the rod-like degradation products were really embedded in the film (compare Supplementary Fig. 18 with Fig. 5b), which thus confirmed our assumption. Finally, we once again draw attention to degradation of the perovskite film without nucleation agent above. Due to the mesoporous structure that was fully exposed to moisture, we did not observe the transition products as the bulk-like pin-hole-free film.

### Performance evaluation

**Figure 7:**
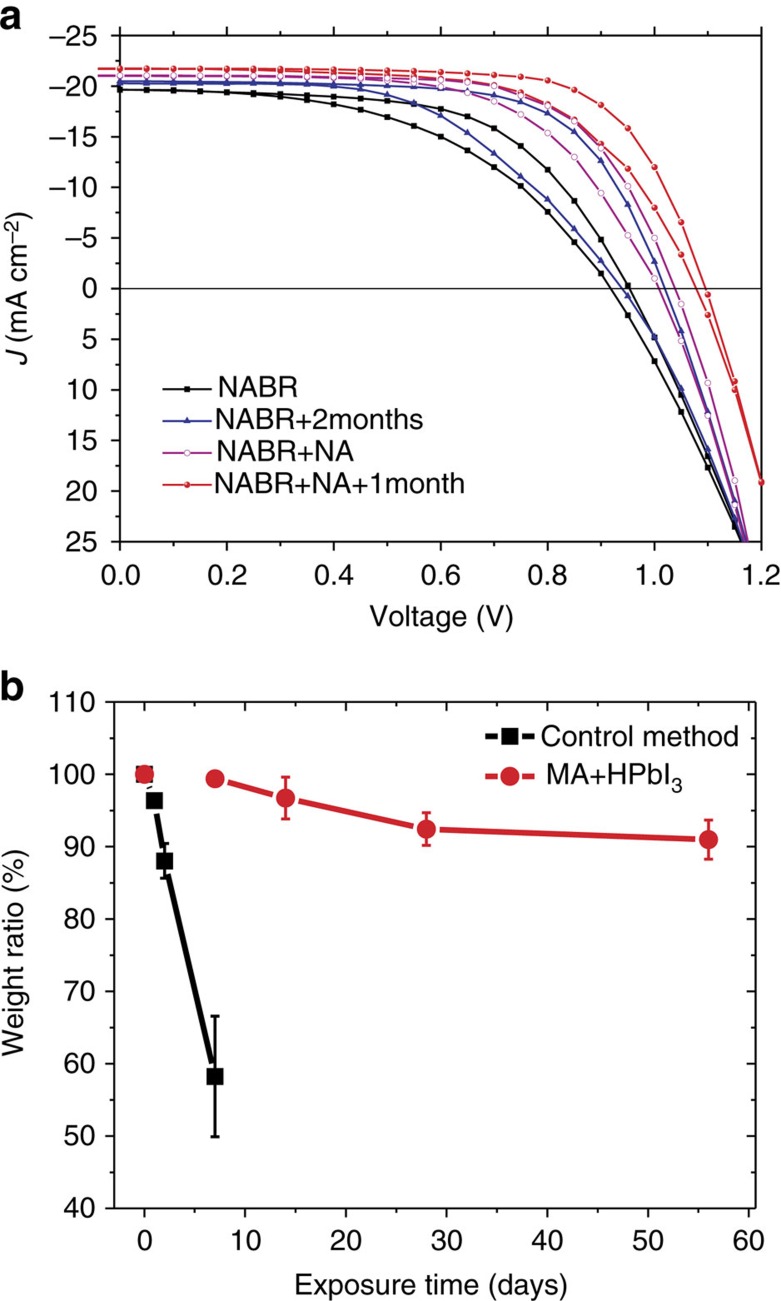
Photovoltaic optimization. (**a**) Optimized solar cell performance with NA (pink line) and without NA (black line) and corresponding solar cell performance after moisture exposure with NA (blue line) and without NA (red line). Notes: perovskite thin films are exposed to moisture and then fabricated as devices. (**b**) Quantitative estimation of degradation through X-ray diffraction external standard method (65% moisture with ambient light soaking). Error bars represent s.d. calculated from five thin films prepared at the same conditions.

The PV performance for freshly prepared and humidity-exposed films on planar compact TiO_2_ and mesoscopic TiO_2_ was carefully conducted in this work (see the Methods section, Fig. 7a, Supplementary Figs 19–22 and Supplementary Tables 1–4). Without nucleation agent, the solar cell displayed low performance due to the poor coverage. The humidity-exposed films had much better power conversion efficiency (14.0%) than freshly prepared film (11.1%) in this system, which meant the robust stability of NABR produced perovskite. In detail, the freshly prepared film produced *V*_oc_=0.90 V, *J*_sc_=19.6 mA cm^−2^, *FF*=0.520 and overall *PCE*=9.1% in forward scan, and *V*_oc_=0.96 V, *J*_sc_=19.6 mA cm^−2^, *FF*=0.590, overall *PCE*=11.1% in reverse scan. After humidity exposure, it produced *V*_oc_=0.94 V, *J*_sc_=20.3 mA cm^−2^, *FF*=0.541, and overall *PCE*=10.3% in forward scan, and *V*_oc_=1.01 V, *J*_sc_=20.4 mA cm^−2^, *FF*=0.681, overall *PCE*=14.0% in reverse scan (Fig. 7a).

After adding nucleation agent, the solar cell displayed much higher performance due to the improved film coverage after series of device optimization (Fig. 7a). The freshly prepared film produced *V*_oc_=1.05 V, *J*_sc_=21.1 mA cm^−2^, *FF*=0.684 and overall *PCE*=15.1% in reverse scan. After 1 month humidity exposure, it produced *V*_oc_=1.08 and 1.07 V, *J*_sc_=21.7 and 21.7 mA cm^−2^, *FF*= 0.725 and 0.651, and overall *PCE*=17.0 and 15.2% in reverse/forward scans. The average 16.1% *PCE* is among the highest efficiency PSC using stable material. This improved performance after humidity exposure demonstrated the improved stability as well.

The improved performance suggests that a thimbleful amount of H_2_O is beneficial to solar cell efficiency. We have tried to track the doping effect and/or surface passivation on solar performance and quantitatively characterize the concentration of pure perovskite film using external standard method with X-ray diffraction patterns (Fig. 7b). The weight concentration was characterized for the control film and NABR produced film. We can see that over 90% perovskite remained after about 2 months, while only about 60% perovskite residue was found after 7 days degradation from the control method. The best performance of perovskite against humidity has doping concentration of about 7%, which is generally consistent with the concentration range in previous work using PbI_2_-rich inorganic/organic composition^37^.

Besides, it is notable that stable PSCs through layer shielding reported in previous works are generally lower than 16.2% efficiency in reverse scan^20^,^38^. According to the detailed reports, MAPbI_3_ perovskite itself could not endure high humidity (55%) for 1–6 days without encapsulation of its high-efficiency photovoltaics, which indicates that its high stability primarily comes from device encapsulation. However, here we demonstrate that NABR using excess CH_3_NH_2_ to react with well-defined HPbI_3_ provides a reliable route to producing material-stable perovskites superior than those reported in previous work^20^,^38^.

This alternative NABR method is probably beneficial for long-term development and large-scale production under ambient conditions, thus reducing the cost in device fabrication and encapsulation. First, in NABR, both CH_3_NH_2_ and HPbI_3_ are chemically stable and thus readily facilitate fabrication. Moreover, the synthesis of HPbI_3_ is much easier than CH_3_NH_3_I and can be collected using ethanol. Second, fundamentally speaking, the use of well-defined HPbI_3_ that is stoichiometrically identical to the intermediate complex as starting material is more chemically reasonable. Besides, we have fabricated a large area PSC up to 1.0 × 0.5 cm^2^ in area, which successfully delivered 15.0% *PCE* at the present stage. Thus, the present study demonstrates up-scaling potential for the assembly of modules in solar cells that function efficiently under ambient condition (Supplementary Fig. 23, Supplementary Table 5).

## Discussion

In conclusion, we have investigated the degradation and recovery of CH_3_NH_3_PbI_3_ perovskite and established an improved stability process. The degraded perovskite can be recovered as fresh perovskite using methylamine CH_3_NH_2_, which means that methylamine can substantially retard the degradation. On the basis of this understanding, we have developed an alternative route using NABR to facilitate the synthesis of perovskite. This NABR procedure, involving the production of HPbI_3_ using excess HI acid and PbI_2_ as well as subsequent reaction between excess CH_3_NH_2_ base and HPbI_3_ acid, provides CH_3_NH_3_PbI_3_ perovskite thin-film that is highly stable under ∼65% humidity for 2 months without appreciable PbI_2_-impurity, whereas other perovskites prepared by the traditional one-step and two-step methods withstand degradation <1 week and 2 weeks, respectively. We have identified a high-quality form of HPbI_3_ with identical Pb(II) coordination number to perovskite and CH_3_NH_2_ abundance as two important factors towards stable perovskite with as less site vacancies as possible. Excess and volatile acid/base leads to full coordination and stoichiometry, respectively, thus eliminating the penetration of water vapour and improving the stability in highly humid environments. The device has been optimized to 17.0/15.2% *PCE*s in forward/reverse scans after 1 month exposure in ∼65% humidity. This work provides an important insight into intrinsic stability and efficiency of perovskite as well as the utilization of simple reaction procedure with up-scaling potential via bottom-up synthetic chemistry for high-performance photovoltaics. Through the reaction demonstration, the vacancy-free insight into improving stability is probably a general paradigm for other perovskites. For example, FA_*x*_Cs_1−*x*_PbI_*y*_Br_3−*y*_, in which the FA^+^/Cs^+^ is also difficult to be removed, delivers as less vacancy as possible at the A sites of AMX_3_ perovskite for high stability. It is challenging to make MAPbI_3_ stable in traditional routes so far. Our NABR route provides a way to enhance the stability through smart reaction control to reduce vacancy without any other material composition. The intermediate structure of HPbI_3_ is especially interesting, in which the proton seems to move freely around the [PbI_3_] column. The perovskite after humidity test has even higher performance in this work, which may arise from protons in HPbI_3_.

## Methods

### Materials

Methylammonium iodide (MAI, Dyesol), PbI_2_ (Sigma-Aldrich, 99%), *N*,*N*-dimethylformamide (DMF, Sigma-Aldrich, anhydrous, 99.8%), spiro-OMeTAD (Merck), 4-tert-butylpyridine (Sigma-Aldrich, 96%), Titania paste (TiO_2_, 30 nm, Dyesol), Titanium(IV) isopropoxide (Sigma-Aldrich, 99.999%), methylamine (Sigma-Aldrich, 33% in ethanol), hydroiodic acid (HI, 57% in water), lithium bistrifluoromethanesulfonimidate (LiTFSI, Sigma-Aldrich, 99.95%), chlorobenzene (Sigma-Aldrich, anhydrous, 99.8%), and all other chemicals were used as received without further purification.

*HPbI_3_ preparation:* HPbI_3_ powder was prepared by mixing PbI_2_ and excess HI (1.5:1 molar HI:PbI_2_) in DMF to ensure complete conversion, and stirring at 40 °C overnight. The light yellow precipitates were obtained by washing the precursor in abundant ethanol to remove excess HI until the supernatant turned to white. The excess HI and ethanol were then removed through filtration. The resulting powders were further dried and stored in an oven at 60 °C. It was then re-dissolved in DMF solution and different amounts of CH_3_NH_2_ ethanol solution were freshly added to obtain the NABR perovskite precursors. The resulting HPbI_3_ sample washed by diethyl ether did not exhibit high stability according to our control experiment. Needle-shaped semi-transparent HPbI_3_ solvate single crystals were grown by dissolving 1.5:1 molar HI:PbI_2_ in DMF followed by vapour diffusion of chlorobenzene into the mother liquor at 80 °C for 6 hours, yielding HPbI_3_ solvate crystal. After heating at 80 °C for 30 min, it turned faint yellow quickly and converted to HPbI_3_ crystal according to single crystal X-ray diffraction. The weight (HI+solvent):PbI_2_ ratio was 37.8% judged from TGA at 385 °C for single-crystal HPbI_3_ solvate, which was freshly collected from the mother liquor and placed on filter paper for 5 min, probably suggesting chemical formula HPbI_3_˙*x*DMF (*x*≤1) with disordered DMF inside the lattice (Supplementary Figs 4 and 24). The quick weight loss before 60 °C suggests the easy release of DMF in HPbI_3_˙*x*DMF even at room temperature, yielding pure HPbI_3_ indicated by 28% weight ratio at 385 °C (HI/PbI_2_) in weight loss after 60 °C. The HPbI_3_ powder recorded a 27.8% HI/PbI_2_ weight ratio at 350 °C by TGA (Supplementary Fig. 13), which confirms the molecular formula HPbI_3_.

### Crystal structure analysis

X-ray intensities of HPbI_3_ solvate and HPbI_3_ were collected at 296 K on a Bruker AXS Kappa Apex II Duo diffractometer with Mo*K*_α_ radiation (*λ*=0.71073 Å) from a sealed-tube generator. Crystal data: hexagonal, *a*=*b*=8.7517 Å, 8.7339 Å, *c*=8.1802 Å, 8.1770 Å for HPbI_3_ solvate and HPbI_3_ with 1:3 atomic Pb/I ratio, respectively. The systematic absences are consistent with both noncentric space group P63*mc* (No. 186) and centric space group P63*mcm* (No. 193). As the proton has negligible X-ray scattering and the DMF molecule exhibits severe orientational disorder, structure determination was based on space group P63*mc* with only the Pb and I atoms subjected to anisotropic least-squares refinement using the SHELXL-97 program; 7,944 reflections measured, of which 509 are unique, *R*_int_=0.099, *R1*=0.044, *wR2*=0.134 and *GOF*=1.05. The crystallographic data for this paper have been deposited with the Cambridge Crystallographic Data Centre (CCDC) as No.1479488. These data can be obtained free of charge from CCDC via www.ccdc.cam.ac.uk/data_request/cif and supporting crystal information files (Supplementary Table 6).

### Device preparation

F-doped SnO_2_ (FTO) (TEC08) substrates were cleaned in an ultrasonic bath sequentially with acetone, 2-propanol and ethanol for 15 min, separately. The TiO_2_ precursor was prepared by 0.6 ml titanium isopropoxide and 0.15 ml 37%w/w HCl solution dissolved in 15 ml ethanol. The dense blocking layer TiO_2_ was coated onto FTO substrate by spin-coating of titanium precursor at 5,000 r.p.m. for 40 s, followed by annealing in air at 500 °C for 30 min. A 14 wt% solution of TiO_2_ nanoparticles in ethanol was spin-coated onto the dense TiO_2_ layer at 5,000 r.p.m. for 40 s to form a mesoporous scaffold and sintered in air at 550 °C for 30 min. After cooling to room temperature, the mesoporous TiO_2_ was immersed in aqueous 30 mM TiCl_4_ at 70 °C for 30 min, rinsed with DI water and annealed at 500 °C for 20 min. The perovskite precursor was prepared by mixing equimolar ratio of MAI and PbI_2_ in DMF by stirring at 60 °C. This solution was then spin-cast onto the TiO_2_ films at 3,000 r.p.m. for 30 s and annealed at 100 °C for 15 min to form the control CH_3_NH_3_PbI_3_ samples. For the perovskite precursors formed by HPbI_3_, 33 wt% MA solution in ethanol was mixed with 1.5 M HPbI_3_ DMF solution with increased volume of MA, and the fresh precursor was spin-coated on TiO_2_ at 2,500 r.p.m. and annealed at 100 °C for 30 min. With device optimization, we also used other concentration HPbI_3_ in association with excess MA and introduced nucleation agent (toluene, 1 ml) during the spin-coating at 10 s for full coverage. The spiro-OMeTAD was prepared by dissolving 75 mg spiro-OMeTAD with 25 μl LiTFSI solution (520 mg in 1 ml acetonitrile) and 35 μl 4-tert-butylpyridine in 1 ml chlorobenzene. Hole transport layer was deposited on the annealed perovskite film by spin-coating at 5,000 r.p.m. for 40 s to get the optimized efficiency. The devices were placed in a moisture-controlled cabinet overnight for oxidization of spiro-OMeTAD. Finally, 80 nm Au electrode was deposited by thermal evaporation with shadow mask area of 0.1 cm^2^. All device fabrication procedures were carried out in N_2_-purged glovebox.

### Stability experiments

For all the humidity stability studies, the perovskite films were spin-coated on FTO glasses, and the films were stored at room temperature (measured as 25±1 °C) in a controlled-humidity cabinet with a transparent glass door, with a low-power fluorescent light in the room. The relative humidity in the chambers was controlled to the desired humidity 65±5% by a beaker of water. And the air humidity in the room is floating from 60 to 75%. The relative humidity in the cabinet was precisely measured periodically throughout the stability experiments using a calibrated hygrometer. The cabinet was only opened just when taking sample out. As the humidity difference is very small, and the humidity cannot be greatly affected when opening the cabinet door and return to the desired humidity by the beaker of water through measurement. To emphasize the stability of perovskite itself and check the performance after humidity exposure, we deposited perovskite films without and with nucleation agent during spin-coating and then expose them in ∼65% humidity for 1–2 months. Humidity-exposed films were assembled into solar cells in the configuration of FTO/TiO_2_/perovskite/spiro-OMeTAD/Au and compared with freshly prepared films.

### Basic characterization

The extinction and absorption spectra of solution samples were measured on a Hitachi U-3501 ultraviolet/visible/NIR spectrophotometer. The general images of the film morphology were obtained using an FEI Quanta 400 field emission scanning electron microscope (FESEM, FEI, Quanta 400 FEG) operated at 10 keV. X-ray diffraction measurements were performed with a Bruker D8 Advance Davinci powder X-ray diffractometer using a CuK_α_ source. TEM imaging was performed on an FEI Tecnai Spirit microscope operating at 120 kV.

### Solar cell test

The current density-voltage curves of solar cells were measured (Keithley Instruments, 2612 Series Source Meter) under simulated AM 1.5 sunlight generated by a 94011A-ES Sol series Solar Simulator. The solar cell devices were tested in N_2_-filled glovebox under room temperature. Solar cell performance was scanned at scan speed 0.5 V s^−1^, dwell time 0.1 s, voltage step 0.05 V in forward and reverse scan loop. The scanning parameters related to the performance were attached (Supplementary Fig. 25). We used the ‘Nicht abdecken’ sensor for the light source checking and then measured the devices. The effective solar cell area was defined by the shadow mask as 0.1 cm^2^.

### Data availability

The authors declare that the data that support the findings of this study are available from the corresponding author on reasonable request.

## Additional information

**How to cite this article:** Long, M. Z. *et al*. Nonstoichiometric acid–base reaction as reliable synthetic route to highly stable CH_3_NH_3_PbI_3_ perovskite film. *Nat. Commun.* **7,** 13503 doi: 10.1038/ncomms13503 (2016).

**Publisher's note:** Springer Nature remains neutral with regard to jurisdictional claims in published maps and institutional affiliations.

## Supplementary Material

Supplementary Information Supplementary Figures 1-25, Supplementary Tables 1-6.

Peer Review File
