# Supplementary material for: Nonstoichiometric acid–base reaction as reliable synthetic route to highly stable CH3NH3PbI3 perovskite film
Source: Nat Commun. 2016 Nov 15;7:13503. doi: 10.1038/ncomms13503 (PMC5476796; doi:10.1038/ncomms13503)
Supplement: Supplementary Information — Supplementary Figures 1-25, Supplementary Tables 1-6. [file ncomms13503-s1.pdf]

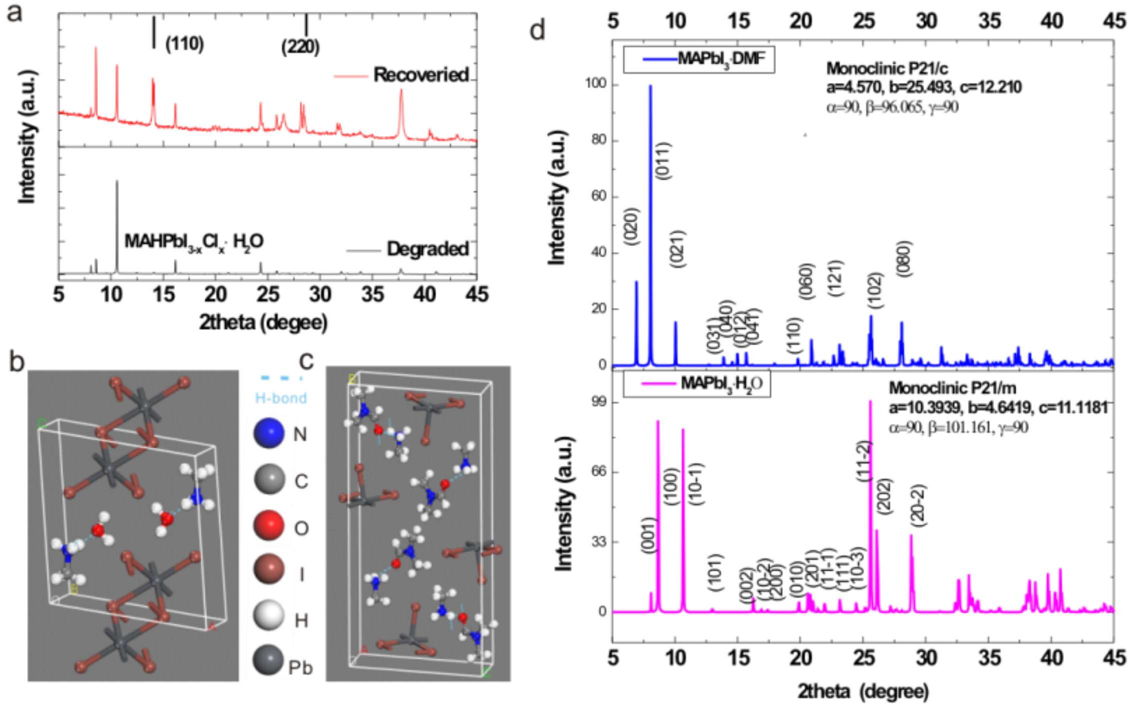

Supplementary Figure 1. (**a**) Degradation and recovery mixed $CH_3NH_3PbI_{3\text{-}x}Cl_x$ perovskite using methylamine. (**b**) Crystal model of $MAPbI_3{\cdot}H_2O$. (**c**) Crystal model of $MAPbI_3{\cdot}DMF$. (**d**) Simulated XRD for verification of transition products using crystal models. Note: The mixed $CH_3NH_3PbI_{3\text{-}x}Cl_x$ perovskite films were sensitive to moisture and the recovery characterization was a little difficult because of the monohydrate in the recovered film.

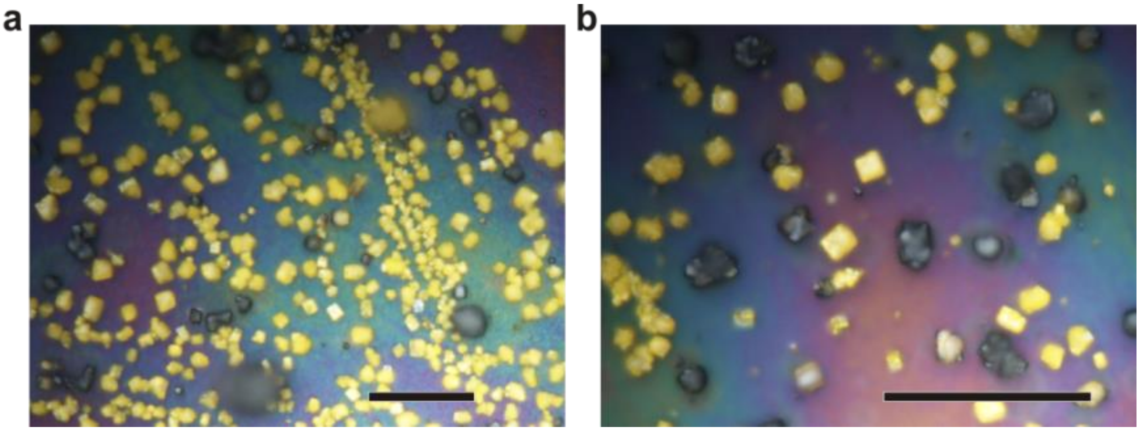

Supplementary Figure 2. Optical microscopic images of partially degraded perovskite suggested that crystal morphology changed little from the parent perovskite. Scale bar: 20μm. Note: The large crystal can be kept more stable in the same condition than small ones, suggesting that bulk-like stoichiometric ingredient leads to better moisture resistance.

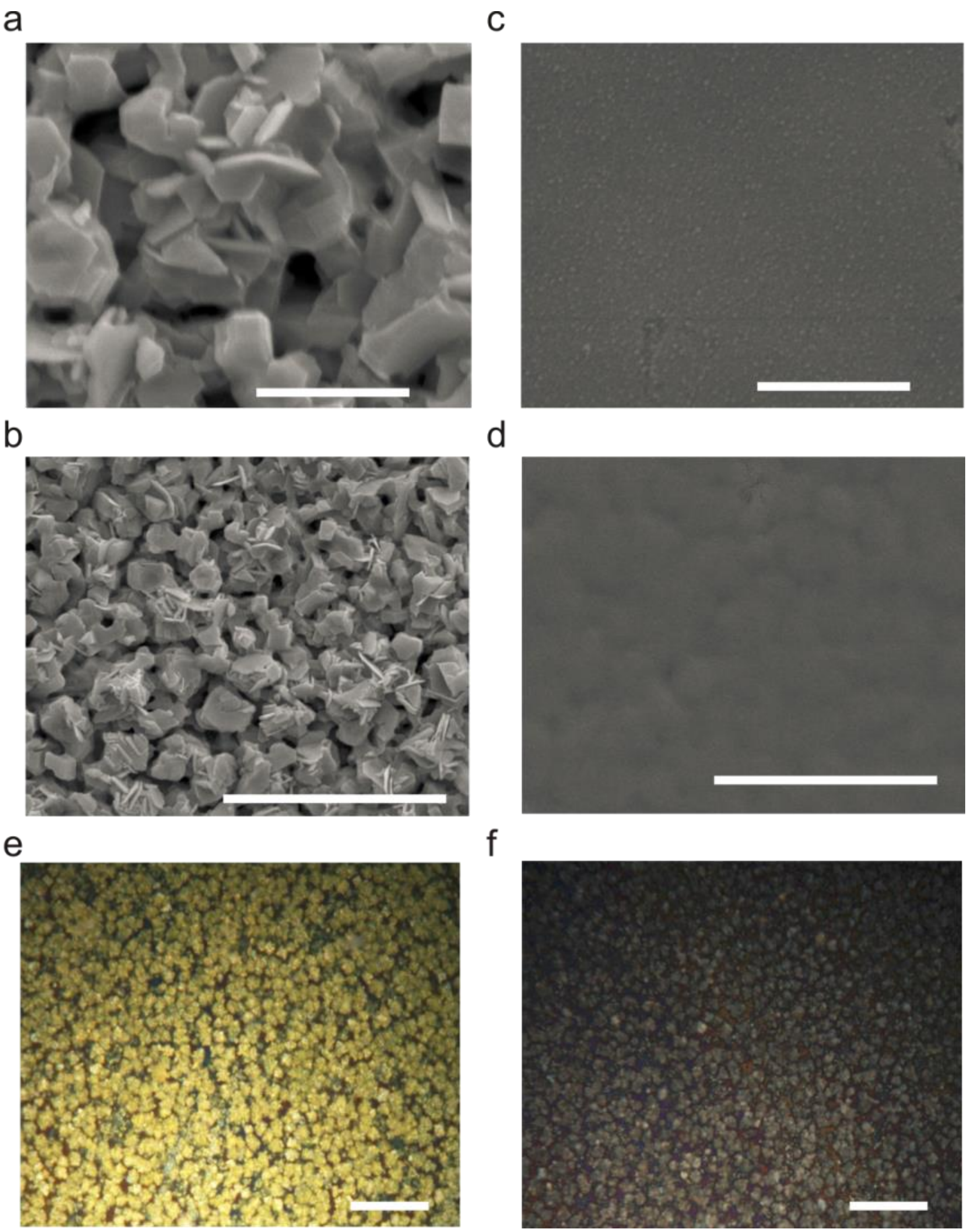

Supplementary Figure 3. SEM images of degraded perovskite films (**a**, **b**) and recovered perovskite films (**c**, **d**) for the observation of the re-crystalline nanocrystals. (**e**, **f**) The optical microscopic images of degraded and recovered films. Scale bar represents 1 μm in (**a**, **c**), 5 μm in (**b**, **d**), and 10 μm in (**e**, **f**).

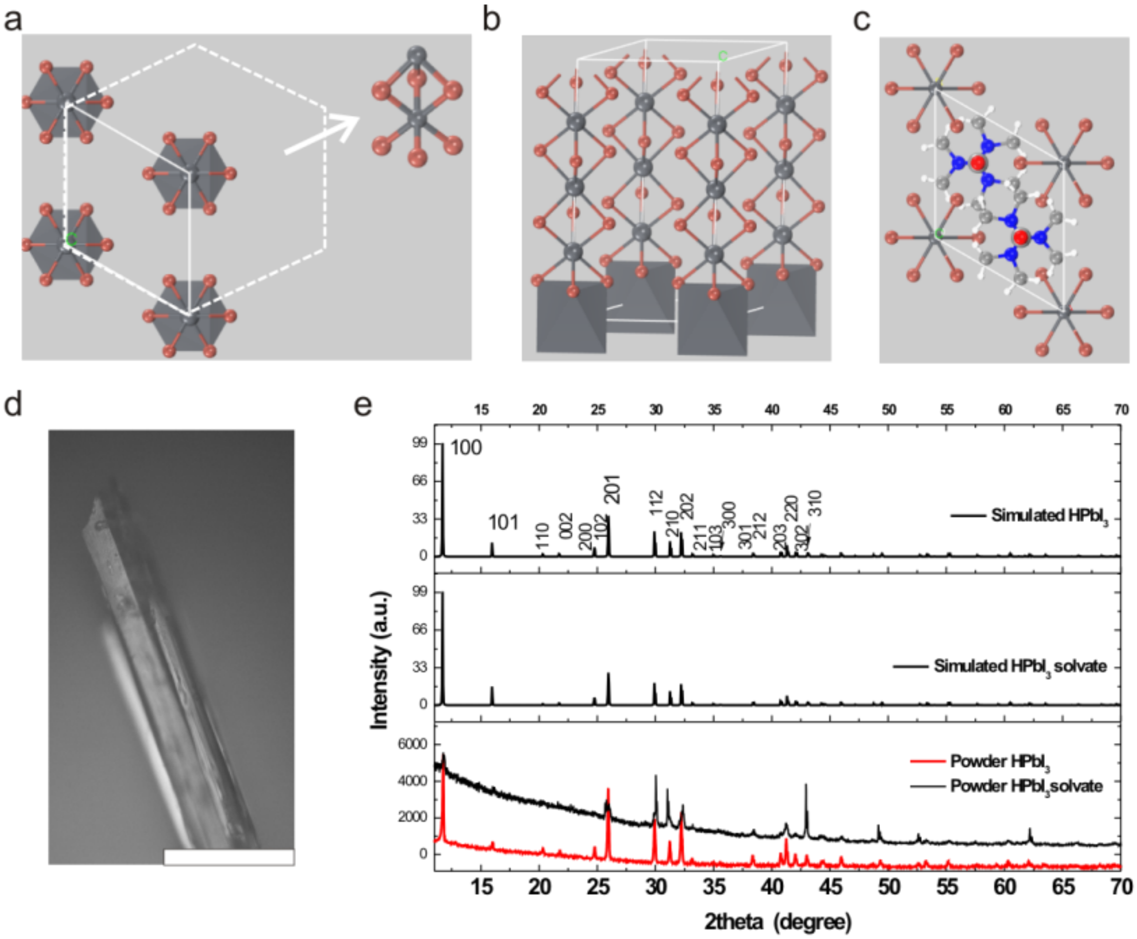

Supplementary Figure 4. Crystal information of $HPbI_3$ and $HPbI_3$ solvate based on single crystal XRD. (**a**) Top view of hexagonal $[PbI_3]^-$ columns and its primitive cell (arrow indicates the (001) face-sharing unit, which seems to be mechanically robust in the form of tripod-stacking) and (**b**) side view of super cell (highly disordered solvent molecule and H^+ ions are not shown, the mobile H^+ ions are expected to be located around $[PbI_3]^-$ columns after solvent release). (**c**) Top view of hexagonal $HPbI_3$ solvate. In order to show the disorder of DMF in the lattice, the occupation of 3 $-N-(CH_3)_2$ groups in DMF is set to 1/3, O of DMF with proton nearby is set to 1/2 occupation on both sides of close center C, and close center C atom (it is amplified for observation) of DMF ($HO-C-N(CH_3)_2$) is 100% occupation for simplicity. (**d**) optical micrograph of as-prepared $HPbI_3$ solvate crystal (scale bar: 1 mm) (After heating at 80 °C for 30 minutes, it loses DMF and becomes $HPbI_3$). (**e**) Simulated powder XRD patterns for $HPbI_3$, $HPbI_3$ solvate based on single crystal XRD produced crystal information, as well as the experimental XRD pattern for $HPbI_3$ powder (spin-coated) and $HPbI_3$ solvate powder (grounded from one ~30 mg crystal).

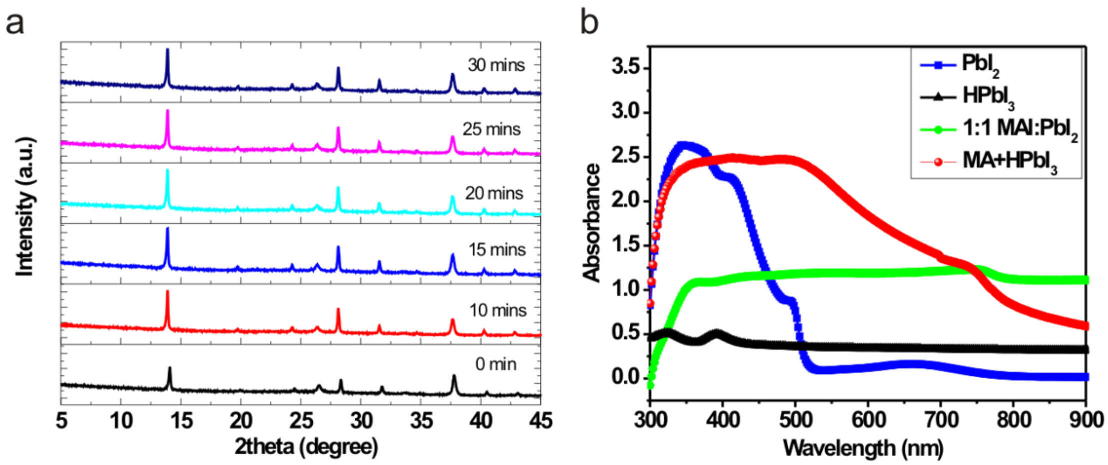

Supplementary Figure 5. (**a**) Variation of powder XRD of perovskite films subjected to thermal annealing at 100°C as a function of time demonstrates that NABR could be processed in an annealing-free way for the conversion to perovskite (100 °C). (**b**) UV absorption of PbI_2, $HPbI_3$, NABR product and control one-step produced perovskite films.

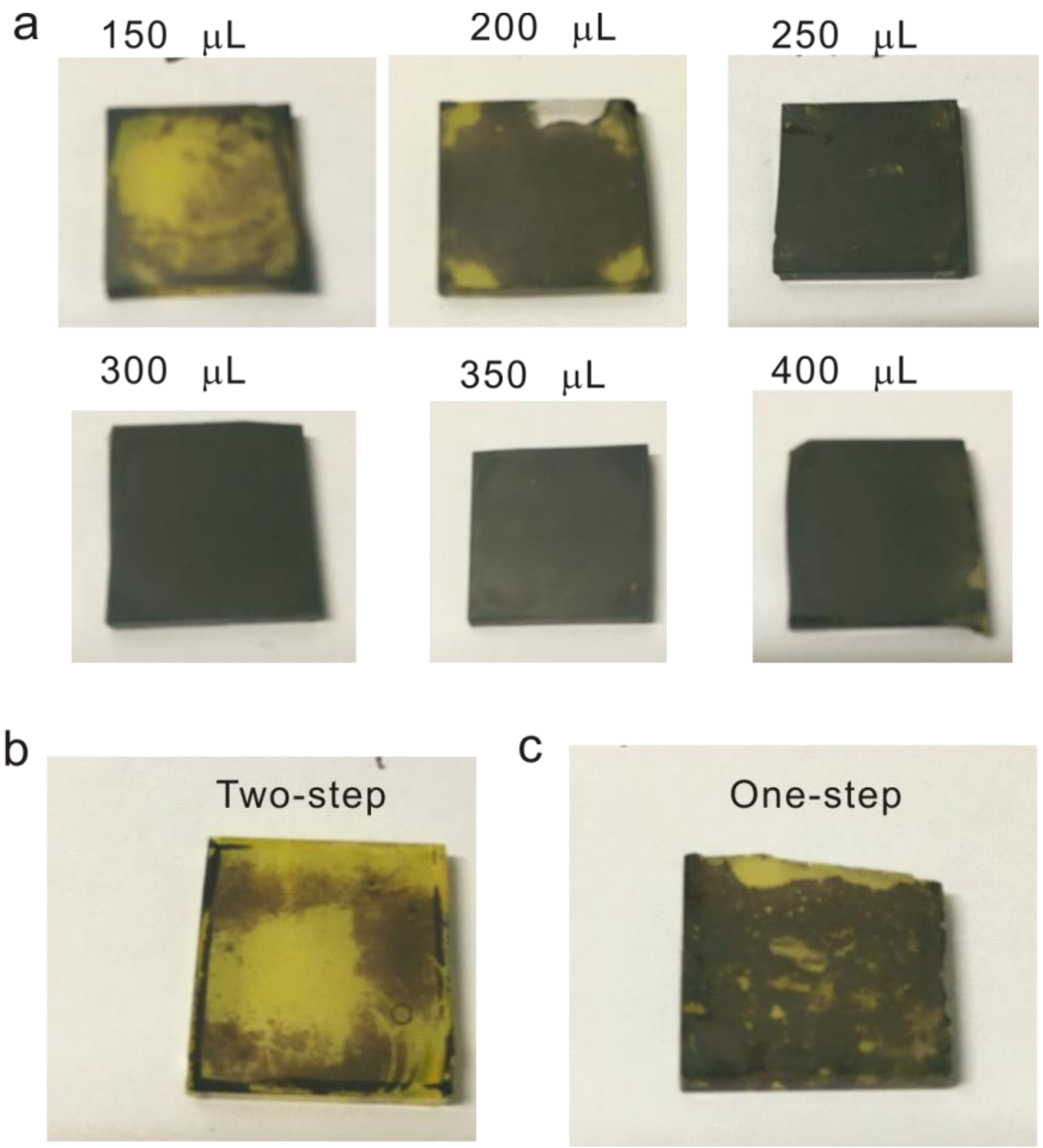

Supplementary Figure 6. (**a**) Photographs of films after stability test in 65% humidity for 2 months suggested the excess MA had high humidity resistance probably due to reduced lattice vacancy. Note: 150, 200, 250, 300, 350, 400 μL of MA are added in 1 mL volume of 1.5 M $HPbI_3$ precursor. (**b**) Degraded films prepared by traditional two-step method and exposed in 65% humidity for 2 weeks. (**c**) Degraded films prepared by traditional one-step method and exposed in 65% humidity for 2 weeks. The substrate size is 1.5×1.5 cm^2.

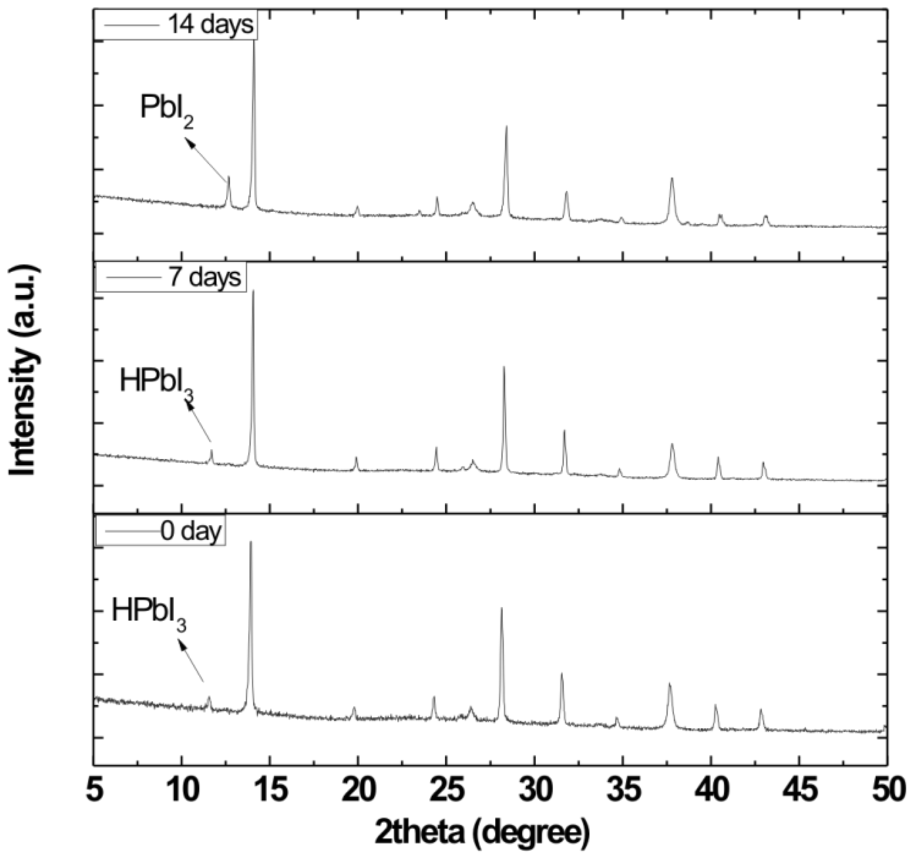

Supplementary Figure 7. XRD monitoring of perovskite prepared by 150 μL MA in 1 mL volume of 1.5 M $HPbI_3$ precursor in 65% humidity.

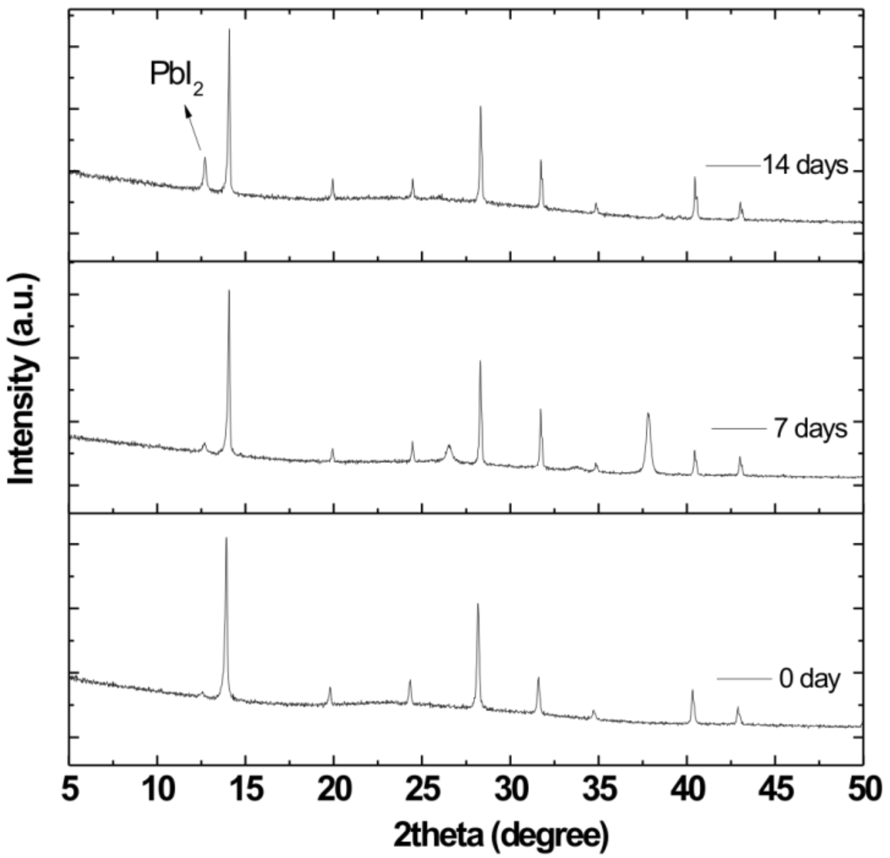

Supplementary Figure 8. XRD monitoring of perovskite prepared by 200 μL MA in 1 mL volume of 1.5 M $HPbI_3$ precursor in 65% humidity.

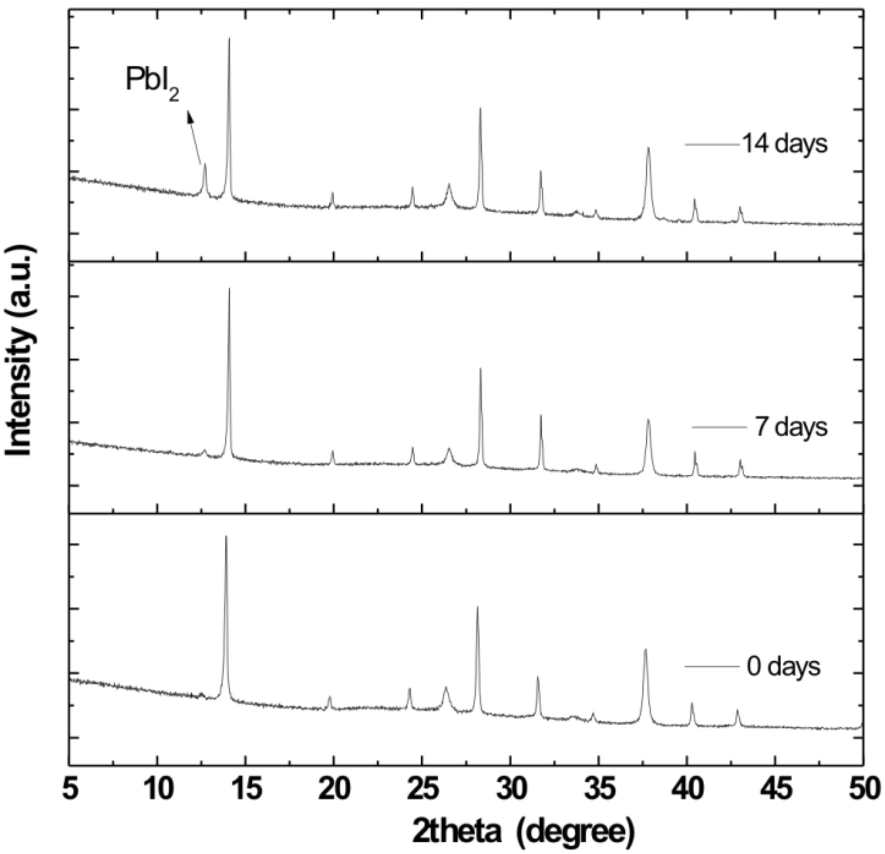

Supplementary Figure 9. XRD monitoring of perovskite prepared by 250 μL MA in 1 mL volume of 1.5 M $HPbI_3$ precursor in 65% humidity.

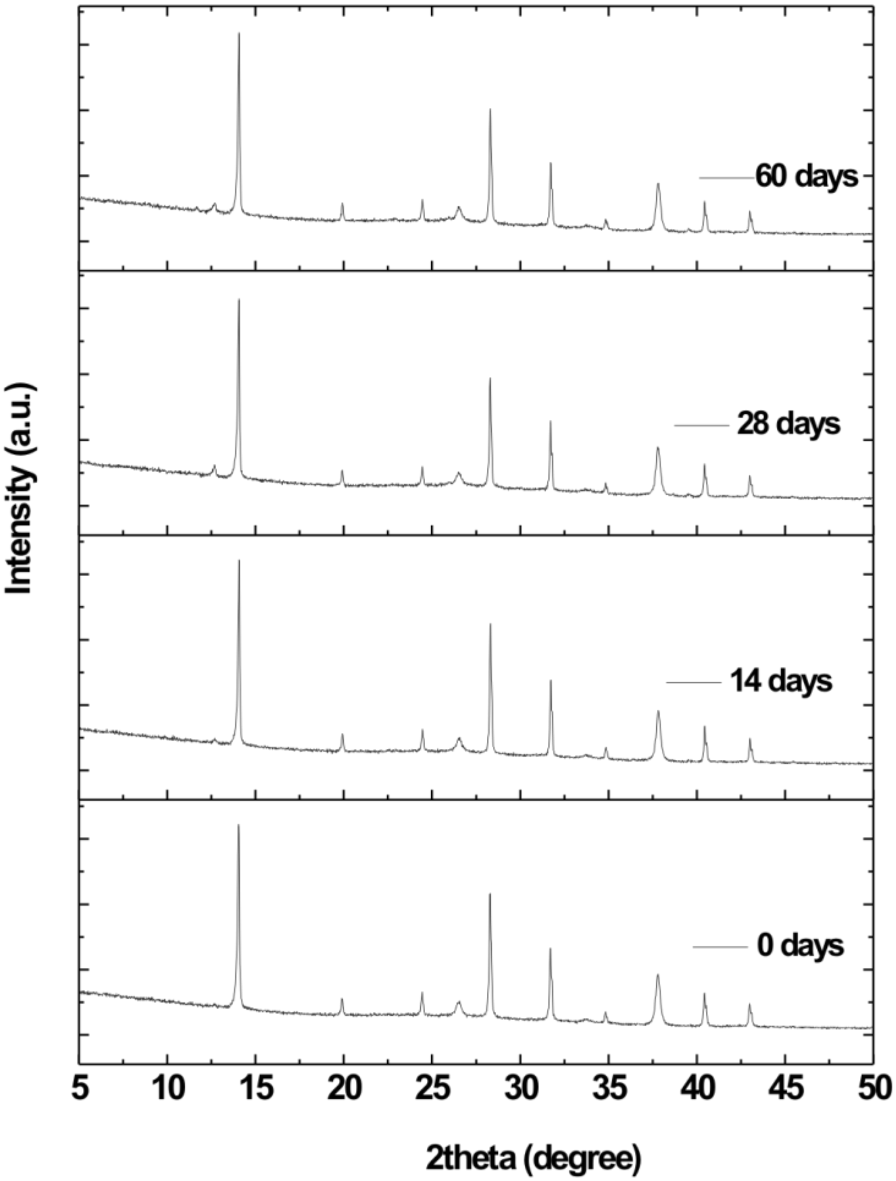

Supplementary Figure 10. XRD monitoring of perovskite prepared by 300 μL MA in 1 mL volume of 1.5 M $HPbI_3$ precursor in 65% humidity.

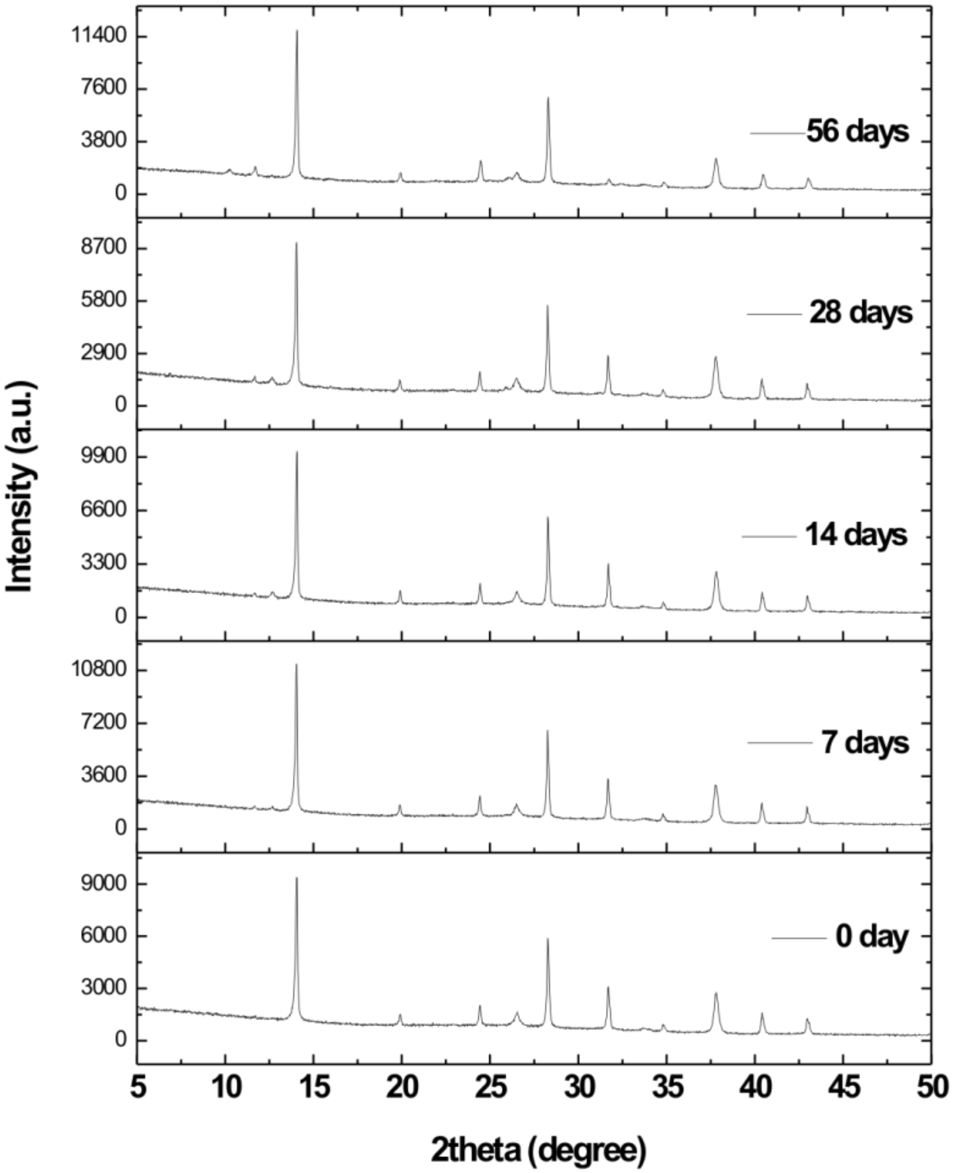

Supplementary Figure 11. XRD monitoring of perovskite prepared by 350 μL MA in 1 mL volume of 1.5 M $HPbI_3$ precursor in 65% humidity.

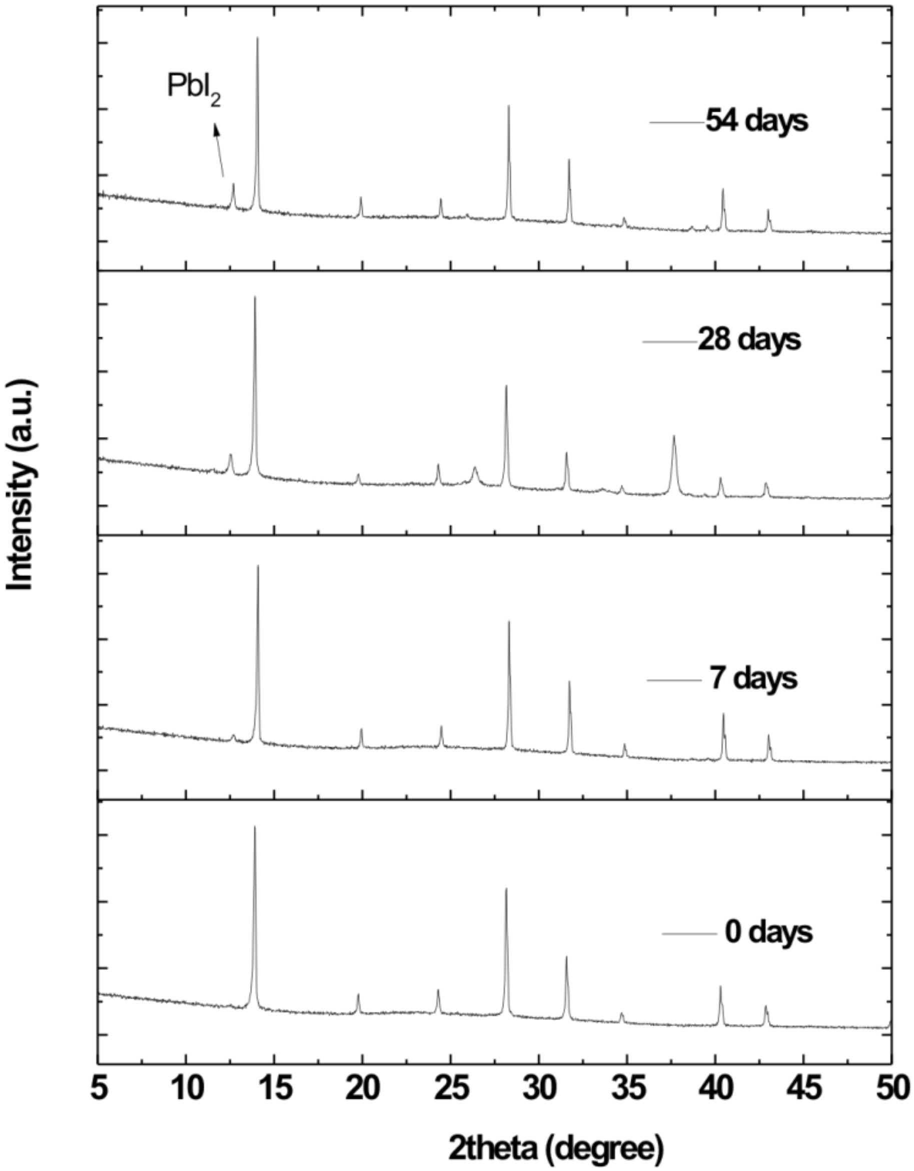

Supplementary Figure 12. XRD monitoring of perovskite prepared by 400 μL MA in 1 mL volume of 1.5 M $HPbI_3$ precursor in 65% humidity.

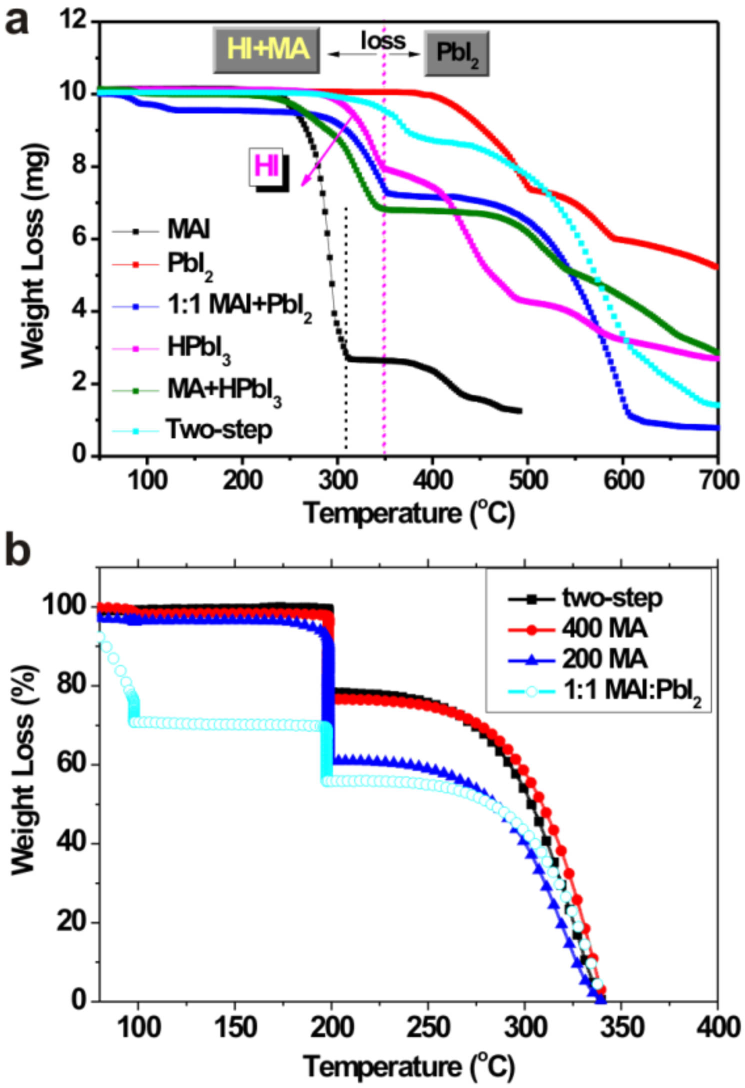

Supplementary Figure 13. Thermal behavior and stability. (**a**) TGA curves of CH_3NH_3I, PbI_2 and perovskites prepared by one-step, two-step and NABR methods. (**b**) TGA curves of four different perovskites with 30 mins heat preservation at 100 ^oC and 120 mins heat preservation at 200 ^oC. Note: weight loss was normalized to [0, 1]. 1:1 MAI: PbI_2 control sample have small weight loss at the onset (lower than 50 ^oC).

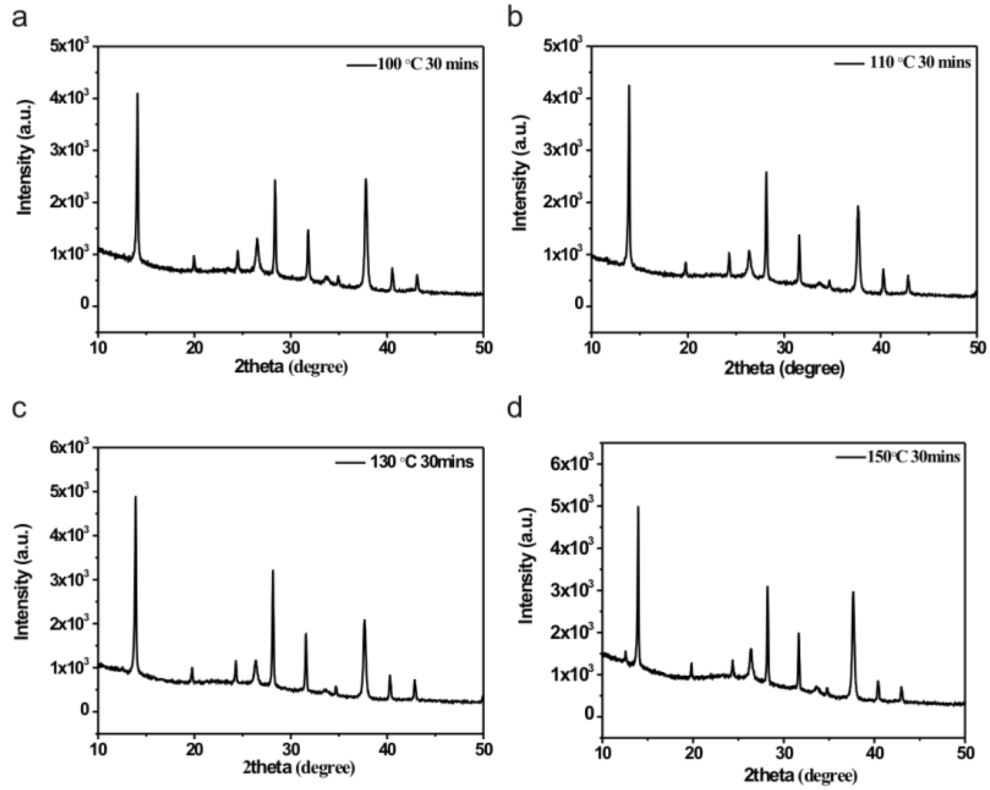

Supplementary Figure 14. XRD of perovskite thin films calcinated under different temperature for 30 mins. (**a**) 100 ^{o}C; (**b**) 110 ^{o}C; (**c**) 130 ^{o}C; (**d**)150 ^{o}C. Notes: NABR can endure a little higher and longer-time heat-stress with detectable PbI_2 impurity compared to traditional method.

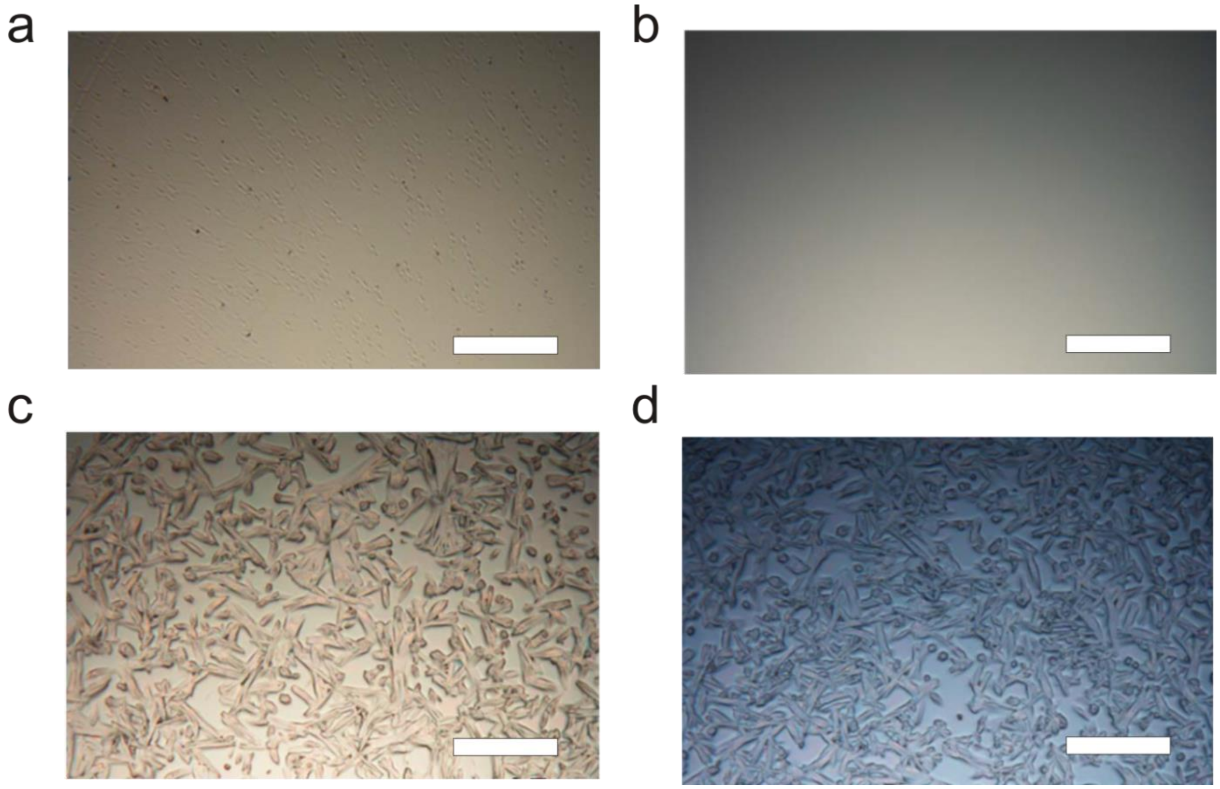

Supplementary Figure 15. Film coverage optimization through dribbling the nucleation agent at 10 seconds for increasing heterogeneous nucleation sites. (**a**) 5 seconds after spin-coating; (**b**) 10 seconds after spin-coating; (**c**) 15 seconds after spin-coating; (**d**) without dripping. Note: too early or too late dribbling time cannot lead to good film coverage. Scale bar: 10 μm.

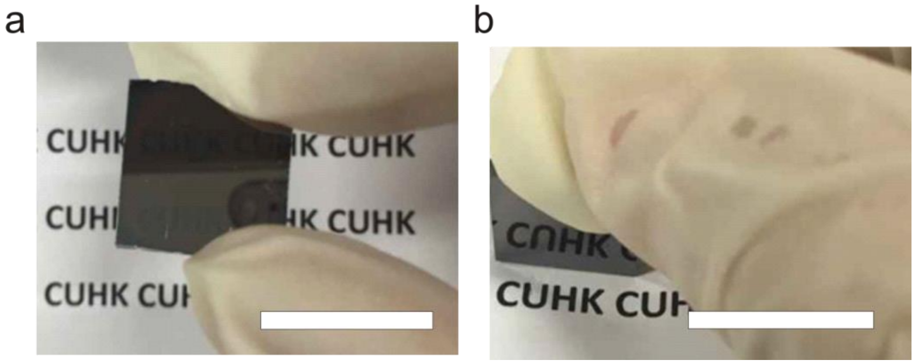

Supplementary Figure 16. Optical images of as-prepared perovskite thin film show good absorption (**a**) and mirror effect (**b**), which indicates that proper thickness and surface uniformity are satisfied for good light harvesting. Scale bar: 1.5 cm.

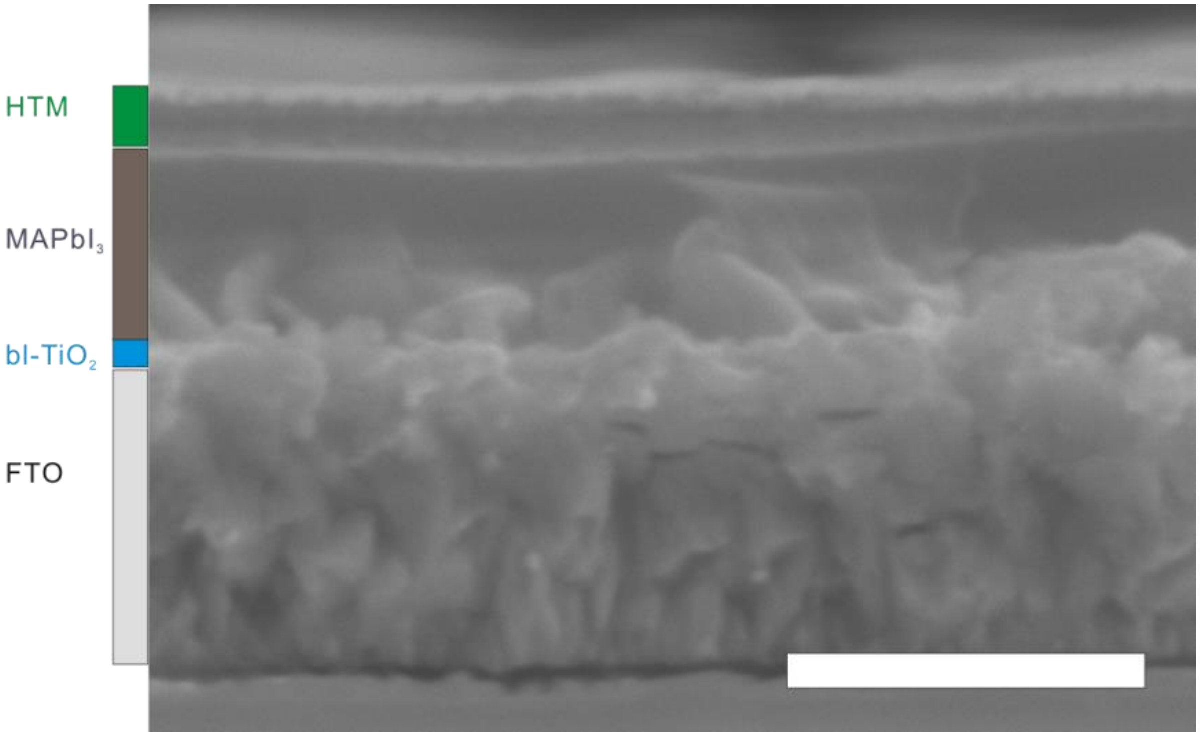

Supplementary Figure 17. Cross-sectional SEM of optimized perovskite solar cell without Au electrode. Note: the perovskite layer was made of polycrystalline bottom layer and spiro-OMeTAD infiltrated top layer free of grain boundary. Scale bar: 500 nm.

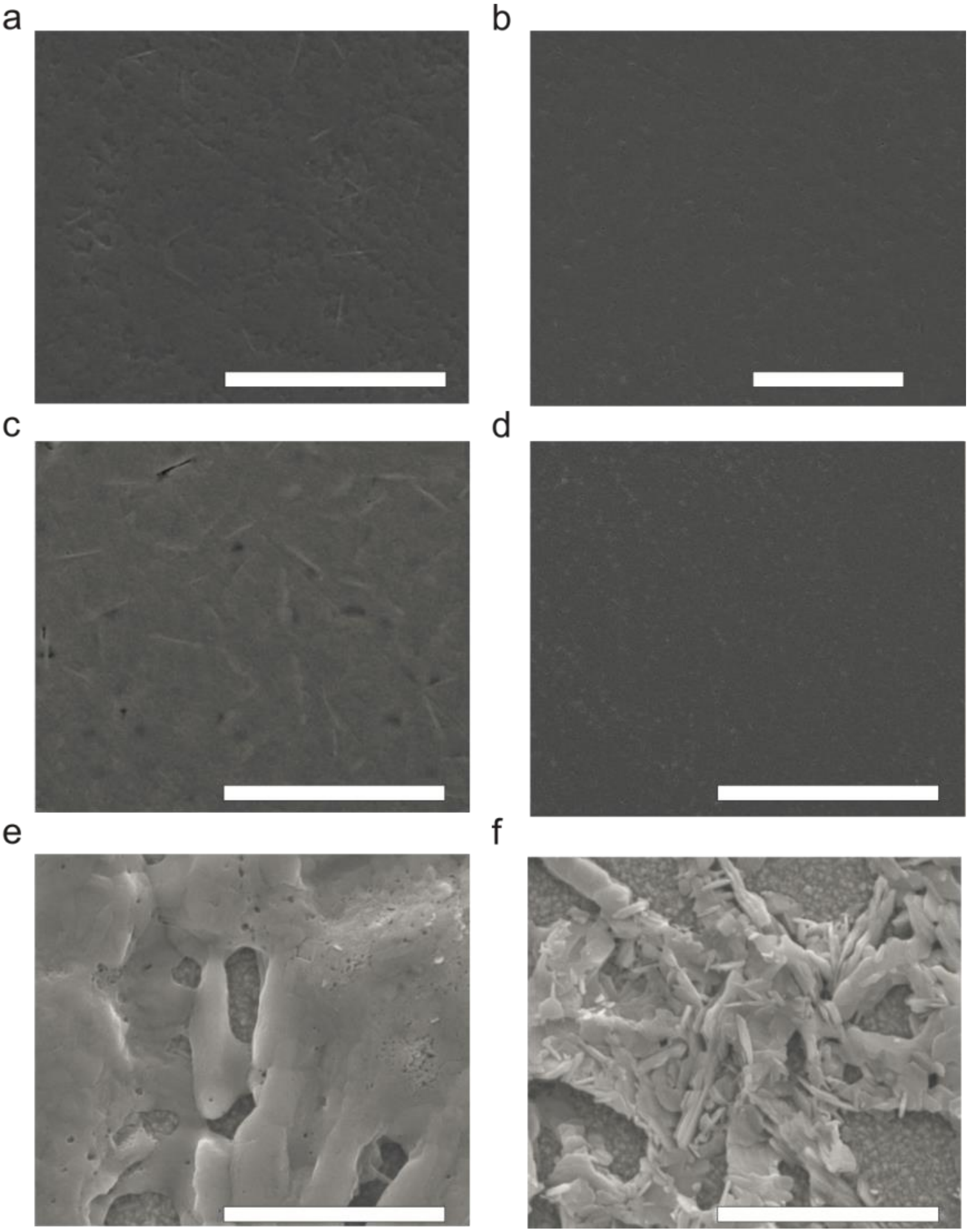

Supplementary Figure 18. Long-term degraded pin-hole free films before (**a**, **b**) and after series of heating (**c**, **d**). Long-term degraded mesoporous black film (**e**, 70 days) and yellow film (**f**, 90 days) without nucleation agent. Scale bar: 3 μm in (**a**, **c**, **e**) and f; 10 μm in (**b**) and 30 μm in (**d**).

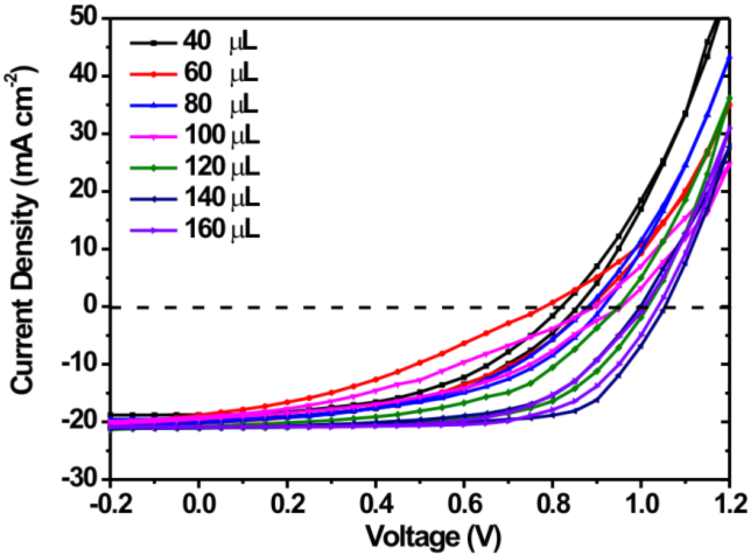

Supplementary Figure 19. MA/$HPbI_3$ composition optimization for high performance: different volumes of MA solution were added to 300 μL $HPbI_3$. Note: Adding a little more MA led to better performance.

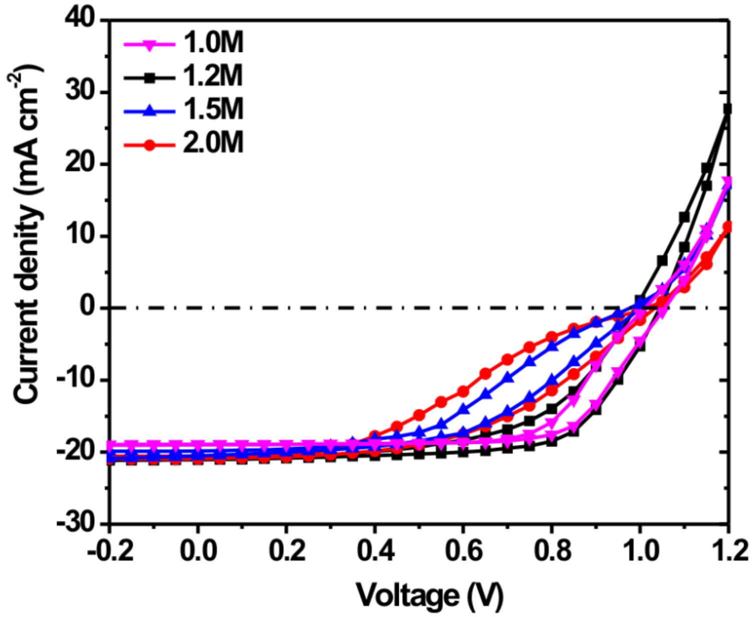

Supplementary Figure 20. Concentration optimization of precursors for high performance.

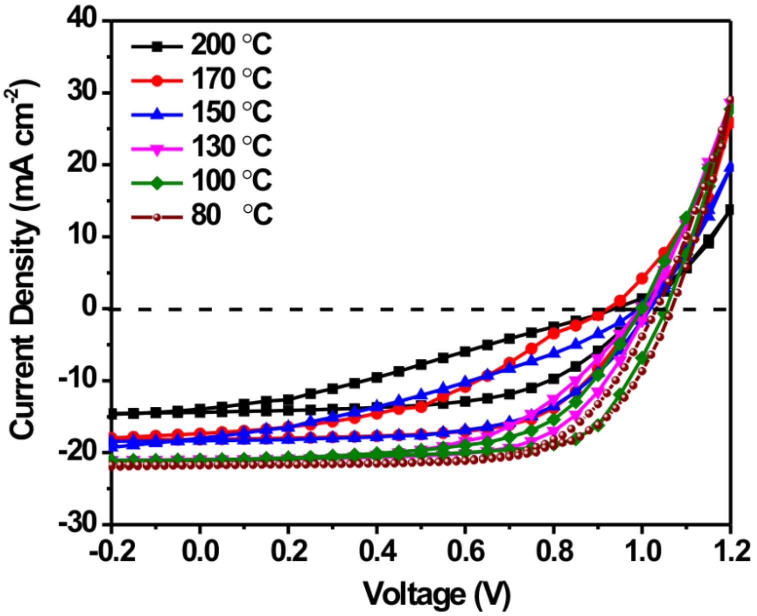

Supplementary Figure 21. Calcination temperature optimization of thin film for high performance. Note: We found temperature below 150 °C could generally result in high performance, and the low and long-time temperature calcination was especially good for reducing hysteresis.

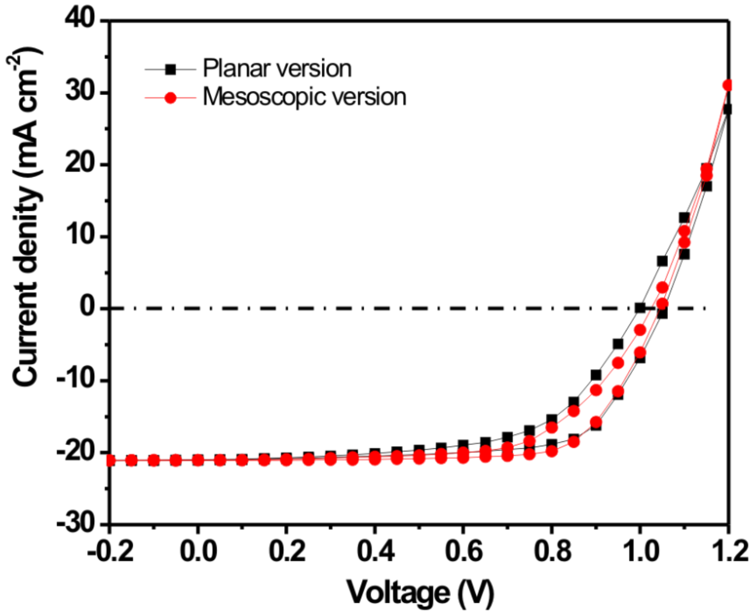

Supplementary Figure 22. Comparison between the planar version and mesoscopic version of perovskite solar cells. Note: There was a little difference between mesoscopic version and planar version in J-V performance, which facilitates simple fabrication.

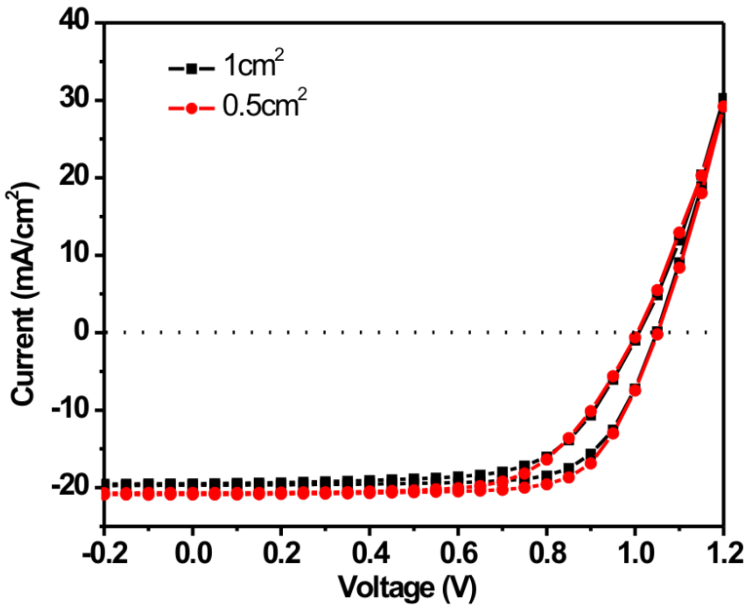

Supplementary Figure 23. Large area performance of perovskite solar cell along NABR method with nucleation agent.

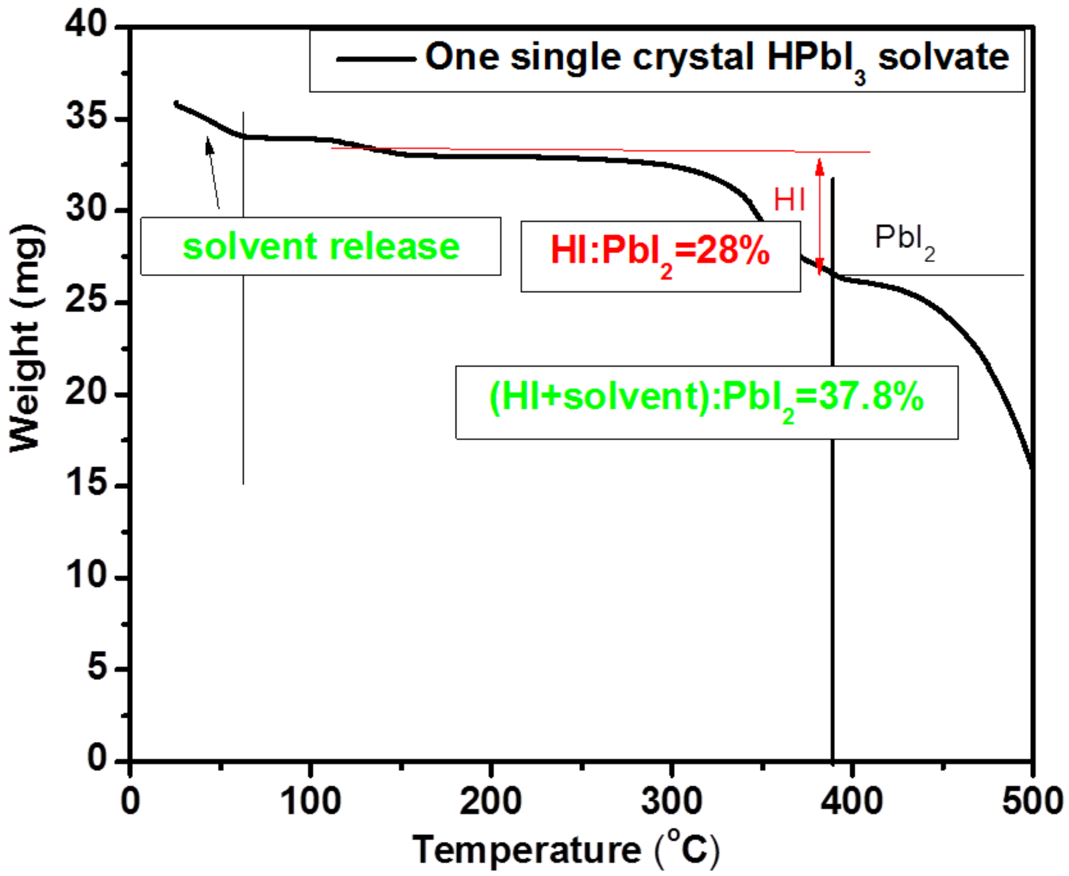

Supplementary Figure 24. TGA analysis for freshly prepared $HPbI_3$ solvate collected from mother liquor (Non-solvate solvents are removed using filter paper for immdiate test and crystal should be large enough), suggesting molecular formula $HPbI_3 \cdot x$DMF ($x \leq 1$) beyond our discussion.

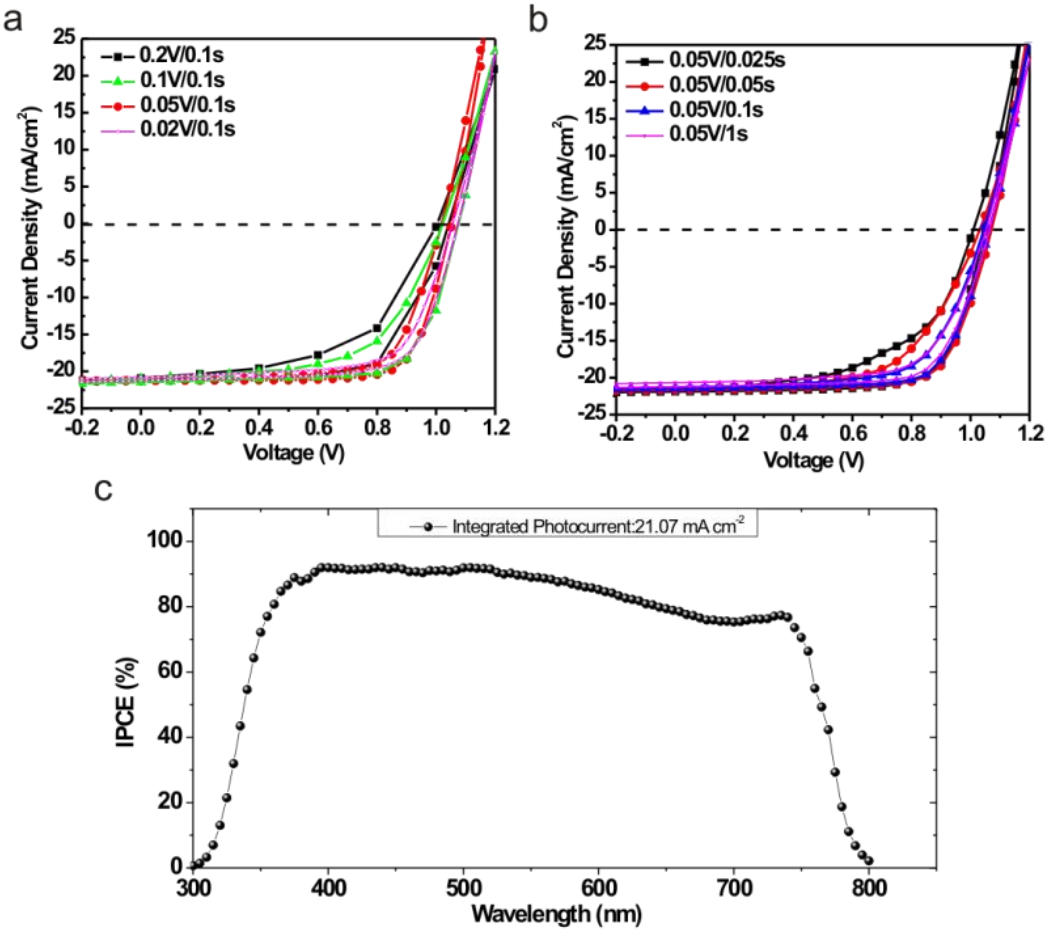

Supplementary Figure 25. The performance check related to the measurement (**a**) voltage scan step and (**b**) voltage scan dwell time effects on the performance; (**c**) IPCE check for the solar cell in (**a**, **b**).

Supplementary Table 1. Performance parameters summarization refers to Supplementary Figure 19

MA (μL)	Scan onset	V_{oc} (V)	J_{sc} (mA cm^{-2})	FF	PCE
40	Reverse	0.86	19.93	48.1	8.24
	Forward	0.83	18.76	47.9	7.47
60	Reverse	0.88	20.20	45.9	8.16
	Forward	0.78	18.79	34.7	5.09
80	Reverse	0.91	20.18	49.1	9.02
	Forward	0.88	19.48	44.3	7.60
100	Reverse	0.95	18.92	46.1	8.28
	Forward	0.89	18.61	37.4	6.19
120	Reverse	1.01	21.02	62.3	13.22
	Forward	0.95	20.76	52.9	10.43
140	Reverse	1.05	21.05	69.6	15.38
	Forward	1.00	20.99	60.4	12.69
160	Reverse	1.03	21.25	66.6	14.58
	Forward	1.00	21.19	61.6	13.05

Supplementary Table 2. Performance parameters summarization refers to Supplementary Figure 20.

$HPbI_3$	Scan onset	V_{oc}(V)	J_{sc}(mA cm^{-2})	FF	PCE
1.0M	Reverse	1.06	18.98	68.9	14.06
	Forward	1.01	18.92	68.5	13.10
1.2M	Reverse	1.05	21.05	69.6	15.38
	Forward	1.00	20.99	60.4	12.69
1.5M	Reverse	1.0	20.52	50.7	10.41
	Forward	0.98	19.84	45.8	8.91
2.0M	Reverse	1.03	20.90	49.5	10.66
	Forward	1.02	20.53	35.4	7.42

Supplementary Table 3. Performance parameters summarization refers to Supplementary Figure 21.

Temperature (^{o}C)	Scan onset	V_{oc} (V)	J_{sc} (mA cm^{-2})	FF	PCE
200 (10mins)	Reverse	1.00	14.39	57.7	8.31
	Forward	0.93	13.96	30.2	3.92
170(15mins)	Reverse	1.00	18.14	63.3	11.49
	Forward	0.92	17.34	42.7	6.81
150(30mins)	Reverse	1.02	18.34	59.7	11.17
	Forward	0.99	18.00	34.5	6.14
130(30mins)	Reverse	1.01	21.06	65.2	13.86
	Forward	0.99	21.06	55.4	11.55
100(30mins)	Reverse	1.05	21.05	69.6	15.38
	Forward	1.00	20.99	60.4	12.69
80 (30mins)	Reverse	1.07	21.70	67.6	15.69
	Forward	1.03	21.68	65.4	14.60

Supplementary Table 4. Performance parameters summarization refers to Supplementary Figure 22.

	**Scan onset**	**V_{oc} (V)**	**J_{sc} (mA cm^{-2})**	**FF**	**PCE**
Planar	Reverse	1.05	21.05	69.6	15.38
	Forward	1.00	20.99	60.4	12.69
Mesoscopic	Reverse	1.03	20.86	71.2	15.34
	Forward	1.01	20.82	65.6	13.79

Supplementary Table 5. Performance parameters summarization refers to Supplementary Figure 23.

Area	V_{oc} (V)	J_{sc} (mA cm^{-2})	FF	PCE
0.5 (Reverse)	1.05	20.8	69.4	15.0
Forward	1.00	20.4	63.9	13.1
1.0 (Reverse)	1.04	19.5	68.9	14.0
Forward	1.01	19.4	65.4	12.8

Supplementary Table 6. Single Crystal XRD for $HPbI_3$ solvate and dry $HPbI_3$

$HPbI_3$ solvate	$HPbI_3$
data_b<br><br>_audit_creation_method 'SHELXL-2014/7'<br>_shelx_SHELXL_version_number '2014/7'<br>_chemical_name_systematic ?<br>_chemical_name_common ?<br>_chemical_melting_point ?<br>_chemical_formula_moiety ?<br>_chemical_formula_sum 'I3 Pb'<br>_chemical_formula_weight 587.89<br><br>loop_<br>_atom_type_symbol<br>_atom_type_description<br>_atom_type_scat_dispersion_real<br>_atom_type_scat_dispersion_imag<br>_atom_type_scat_source<br>'I' 'I' -0.4742 1.8119<br>'International Tables Vol C Tables 4.2.6.8 and 6.1.1.4'<br>'Pb' 'Pb' -3.3944 10.1111<br>'International Tables Vol C Tables 4.2.6.8 and 6.1.1.4'<br><br>_space_group_crystal_system hexagonal<br>_space_group_IT_number 186<br>_space_group_name_H-M_alt 'P 63 m c'<br>_space_group_name_Hall 'P 6c -2c'<br><br>_shelx_space_group_comment<br>;<br>The symmetry employed for this shelxl refinement is uniquely defined<br>by the following loop, which should always be used as a source of symmetry information in preference to the above space-group names.<br>They are only intended as comments.<br>;<br><br>loop_<br><br>_space_group_symop_operation_xyz<br>'x, y, z'<br>'-y, x-y, z'<br>'-x+y, -x, z'<br>'-x, -y, z+1/2'<br>'y, -x+y, z+1/2'<br>'x-y, x, z+1/2'<br>'-y, -x, z'<br>'-x+y, y, z'<br>'x, x-y, z'<br>'y, x, z+1/2'<br>'x-y, -y, z+1/2'<br>'-x, -x+y, z+1/2'<br><br>_cell_length_a 8.7517(3)<br>_cell_length_b 8.7517(3)<br>_cell_length_c 8.1802(5)<br>_cell_angle_alpha 90<br>_cell_angle_beta 90<br>_cell_angle_gamma 120<br>_cell_volume 542.60(5)<br>_cell_formula_units_Z 2<br>_cell_measurement_temperature 296(2)<br>_cell_measurement_reflns_used 149<br>_cell_measurement_theta_min 15<br>_cell_measurement_theta_max 15<br><br>_exptl_crystal_description block<br>_exptl_crystal_colour yellow<br>_exptl_crystal_density_meas ?<br>_exptl_crystal_density_method ?<br>_exptl_crystal_density_diffrn 3.598<br>_exptl_crystal_F_000 482<br>_exptl_transmission_factor_min ?<br>_exptl_transmission_factor_max ?<br>_exptl_crystal_size_max ?<br>_exptl_crystal_size_mid ?<br>_exptl_crystal_size_min ?<br>_exptl_absorpt_coefficient_mu 23.992<br>_shelx_estimated_absorpt_T_min ?<br>_shelx_estimated_absorpt_T_max ?	data_a<br><br>_audit_creation_method 'SHELXL-2014/7'<br>_shelx_SHELXL_version_number '2014/7'<br>_chemical_name_systematic ?<br>_chemical_name_common ?<br>_chemical_melting_point ?<br>_chemical_formula_moiety ?<br>_chemical_formula_sum 'I3 Pb'<br>_chemical_formula_weight 587.89<br><br>loop_<br>_atom_type_symbol<br>_atom_type_description<br>_atom_type_scat_dispersion_real<br>_atom_type_scat_dispersion_imag<br>_atom_type_scat_source<br>'I' 'I' -0.4742 1.8119<br>'International Tables Vol C Tables 4.2.6.8 and 6.1.1.4'<br>'Pb' 'Pb' -3.3944 10.1111<br>'International Tables Vol C Tables 4.2.6.8 and 6.1.1.4'<br><br>_space_group_crystal_system hexagonal<br>_space_group_IT_number 186<br>_space_group_name_H-M_alt 'P 63 m c'<br>_space_group_name_Hall 'P 6c -2c'<br><br>_shelx_space_group_comment<br>;<br>The symmetry employed for this shelxl refinement is uniquely defined<br>by the following loop, which should always be used as a source of symmetry information in preference to the above space-group names.<br>They are only intended as comments.<br>;<br><br>loop_<br><br>_space_group_symop_operation_xyz<br>'x, y, z'<br>'-y, x-y, z'<br>'-x+y, -x, z'<br>'-x, -y, z+1/2'<br>'y, -x+y, z+1/2'<br>'x-y, x, z+1/2'<br>'-y, -x, z'<br>'-x+y, y, z'<br>'x, x-y, z'<br>'y, x, z+1/2'<br>'x-y, -y, z+1/2'<br>'-x, -x+y, z+1/2'<br><br>_cell_length_a 8.7339(10)<br>_cell_length_b 8.7339(10)<br>_cell_length_c 8.1770(11)<br>_cell_angle_alpha 90<br>_cell_angle_beta 90<br>_cell_angle_gamma 120<br>_cell_volume 540.18(14)<br>_cell_formula_units_Z 2<br>_cell_measurement_temperature 296(2)<br>_cell_measurement_reflns_used 95<br>_cell_measurement_theta_min 15<br>_cell_measurement_theta_max 15<br><br>_exptl_crystal_description block<br>_exptl_crystal_colour yellow<br>_exptl_crystal_density_meas ?<br>_exptl_crystal_density_method ?<br>_exptl_crystal_density_diffrn 3.614<br>_exptl_crystal_F_000 482<br>_exptl_transmission_factor_min ?<br>_exptl_transmission_factor_max ?<br>_exptl_crystal_size_max ?<br>_exptl_crystal_size_mid ?<br>_exptl_crystal_size_min ?<br>_exptl_absorpt_coefficient_mu 24.099
_exptl_absorpt_correction_type multi-scan<br>_exptl_absorpt_correction_T_min 0.0818<br>_exptl_absorpt_correction_T_max 0.7456<br>_exptl_absorpt_process_details ?<br>_exptl_absorpt_special_details ?<br>_diffrn_ambient_temperature 296(2)<br>_diffrn_radiation_wavelength 0.71073<br>_diffrn_radiation_type MoK\a<br>diffrn_source ?<br>_diffrn_measurement_device_type 'BRUKER D8VENTURE DIFFRACTOMETER'<br>_diffrn_measurement_method 'PHI & OMEGA SCAN'<br>_diffrn_detector_area_resol_mean ?<br>_diffrn_reflns_number 3517<br>_diffrn_reflns_av_unetI/netI 0.0538<br>_diffrn_reflns_av_R_equivalents 0.0922<br>_diffrn_reflns_limit_h_min -9<br>_diffrn_reflns_limit_h_max 11<br>_diffrn_reflns_limit_k_min -11<br>_diffrn_reflns_limit_k_max 9<br>_diffrn_reflns_limit_l_min -10<br>_diffrn_reflns_limit_l_max 10<br>_diffrn_reflns_theta_min 2.687<br>_diffrn_reflns_theta_max 27.818<br>_diffrn_reflns_theta_full 25.242<br>_diffrn_measured_fraction_theta_max 0.993<br>_diffrn_measured_fraction_theta_full 0.991<br>_diffrn_reflns_Laue_measured_fraction_max 0.993<br>_diffrn_reflns_Laue_measured_fraction_full 0.991<br>_diffrn_reflns_point_group_measured_fraction_max 0.987<br>_diffrn_reflns_point_group_measured_fraction_full 0.985<br>_reflns_number_total 514<br>_reflns_number_gt 441<br>_reflns_threshold_expression 'I > 2\s(I)'<br>_reflns_Friedel_coverage 0.856<br>_reflns_Friedel_fraction_max 0.979<br>_reflns_Friedel_fraction_full 0.978<br><br>_reflns_special_details<br>;<br>Reflections were merged by SHELXL according to the crystal class for the calculation of statistics and refinement.<br><br>_reflns_Friedel_fraction is defined as the number of unique Friedel pairs measured divided by the number that would be possible theoretically, ignoring centric projections and systematic absences.<br>;<br><br>_computing_data_collection 'BRUKER D8VENTURE DIFFRACTOMETER'<br>_computing_cell_refinement 'BRUKER D8VENTURE DIFFRACTOMETER'<br>_computing_data_reduction 'BRUKER D8VENTURE DIFFRACTOMETER'<br>_computing_structure_solution ?<br>_computing_structure_refinement 'SHELXL-2014/7 (Sheldrick, 2014)'<br>_computing_molecular_graphics ?<br>_computing_publication_material ?<br>_refine_special_details ?<br>_refine_ls_structure_factor_coef Fsqd<br>_refine_ls_matrix_type full<br>_refine_ls_weighting_scheme calc<br>_refine_ls_weighting_details 'w=1/[\s^2^(Fo^2^)+(0.1458P)^2^+1.0126P] where P=(Fo^2^+2Fc^2^)/3'<br>_atom_sites_solution_primary ?<br>_atom_sites_solution_secondary ?<br>_atom_sites_solution_hydrogens .<br>_refine_ls_hydrogen_treatment	_shelx_estimated_absorpt_T_min ?<br>_shelx_estimated_absorpt_T_max ?<br>_exptl_absorpt_correction_type multi-scan<br>_exptl_absorpt_correction_T_min 0.2013<br>_exptl_absorpt_correction_T_max 0.7456<br>_exptl_absorpt_process_details ?<br>_exptl_absorpt_special_details ?<br>_diffrn_ambient_temperature 296(2)<br>_diffrn_radiation_wavelength 0.71073<br>_diffrn_radiation_type MoK\a<br>diffrn_source ?<br>_diffrn_measurement_device_type "BRUKER KAPPA APEXII DUO DIFFRACTOMETER"<br>_diffrn_measurement_method 'PHI & OMEGA SCAN'<br>_diffrn_detector_area_resol_mean ?<br>_diffrn_reflns_number 8006<br>_diffrn_reflns_av_unetI/netI 0.0362<br>_diffrn_reflns_av_R_equivalents 0.0985<br>_diffrn_reflns_limit_h_min -11<br>_diffrn_reflns_limit_h_max 11<br>_diffrn_reflns_limit_k_min -11<br>_diffrn_reflns_limit_k_max 11<br>_diffrn_reflns_limit_l_min -10<br>_diffrn_reflns_limit_l_max 9<br>_diffrn_reflns_theta_min 2.693<br>_diffrn_reflns_theta_max 28.153<br>_diffrn_reflns_theta_full 25.242<br>_diffrn_measured_fraction_theta_max 1.000<br>_diffrn_measured_fraction_theta_full 1.000<br>_diffrn_reflns_Laue_measured_fraction_max 1.000<br>_diffrn_reflns_Laue_measured_fraction_full 1.000<br>_diffrn_reflns_point_group_measured_fraction_max 0.964<br>_diffrn_reflns_point_group_measured_fraction_full 0.980<br>_reflns_number_total 513<br>_reflns_number_gt 461<br>_reflns_threshold_expression 'I > 2\s(I)'<br>_reflns_Friedel_coverage 0.800<br>_reflns_Friedel_fraction_max 0.923<br>_reflns_Friedel_fraction_full 0.956<br><br>_reflns_special_details<br>;<br>Reflections were merged by SHELXL according to the crystal class for the calculation of statistics and refinement.<br><br>_reflns_Friedel_fraction is defined as the number of unique Friedel pairs measured divided by the number that would be possible theoretically, ignoring centric projections and systematic absences.<br>;<br><br>_computing_data_collection "BRUKER KAPPA APEXII DUO DIFFRACTOMETER"<br>_computing_cell_refinement "BRUKER KAPPA APEXII DUO DIFFRACTOMETER"<br>_computing_data_reduction "BRUKER KAPPA APEXII DUO DIFFRACTOMETER"<br>_computing_structure_solution ?<br>_computing_structure_refinement 'SHELXL-2014/7 (Sheldrick, 2014)'<br>_computing_molecular_graphics ?<br>_computing_publication_material ?<br>_refine_special_details ?<br>_refine_ls_structure_factor_coef Fsqd<br>_refine_ls_matrix_type full<br>_refine_ls_weighting_scheme calc<br>_refine_ls_weighting_details 'w=1/[\s^2^(Fo^2^)+(0.1000P)^2^] where P=(Fo^2^+2Fc^2^)/3'<br>_atom_sites_solution_primary ?

undef
_refine_ls_extinction_method
'SHELXL-2014/7 (Sheldrick 2014'
_refine_ls_extinction_coef
0.038(9)
_refine_ls_extinction_expression

'Fc^*^=kFc[1+0.001xFc^2^\l^3^/si
n(2\q)]^-1/4^'
_refine_ls_abs_structure_details
;
Flack x determined using 169
quotients [(I+)-(I-)]/[(I+)+(I-)]
(Parsons, Flack and Wagner, Acta
Cryst. B69 (2013) 249-259).
;
_refine_ls_abs_structure_Flack
0.6(2)
_chemical_absolute_configuration
?
_refine_ls_number_reflns 514
_refine_ls_number_parameters
11
_refine_ls_number_restraints 1
_refine_ls_R_factor_all
0.0700
_refine_ls_R_factor_gt
0.0624
_refine_ls_wR_factor_ref
0.2099
_refine_ls_wR_factor_gt
0.1938
_refine_ls_goodness_of_fit_ref
1.093
_refine_ls_restrained_S_all
1.092
_refine_ls_shift/su_max 0.000
_refine_ls_shift/su_mean
0.000

loop_
_atom_site_label
_atom_site_type_symbol
_atom_site_fract_x
_atom_site_fract_y
_atom_site_fract_z
_atom_site_U_iso_or_equiv
_atom_site_adp_type
_atom_site_occupancy
_atom_site_site_symmetry_order
_atom_site_calc_flag
_atom_site_refinement_flags_posn
_atom_site_refinement_flags_adp

_atom_site_refinement_flags_occu
pancy
_atom_site_disorder_assembly
_atom_site_disorder_group
Pb1 Pb 0.0000 0.0000 0.3752(3)
0.0557(10) Uani 1 6 d S T P . .
I1 I 0.3287(2) 0.16434(10) 0.6252(3)
0.0932(12) Uani 1 2 d S T P . .

loop_
_atom_site_aniso_label
_atom_site_aniso_U_11
_atom_site_aniso_U_22
_atom_site_aniso_U_33
_atom_site_aniso_U_23
_atom_site_aniso_U_13
_atom_site_aniso_U_12
Pb1 0.0586(11) 0.0586(11)
0.0500(12) 0.000 0.000 0.0293(5)
I1 0.0524(12) 0.1120(17) 0.0954(17)
-0.0035(8) -0.0070(16) 0.0262(6)

_geom_special_details
;
All esds (except the esd in the
dihedral angle between two l.s.
planes)
are estimated using the full covari-
ance matrix. The cell esds are
taken
into account individually in the
estimation of esds in distances,
angles
and torsion angles; correlations
between esds in cell parameters are
only
used when they are defined by
crystal symmetry. An approximate
(isotropic)
treatment of cell esds is used for
estimating esds involving l.s.
planes.
;

loop_
_geom_bond_atom_site_label_1
_geom_bond_atom_site_label_2
_geom_bond_distance
_geom_bond_site_symmetry_2
_geom_bond_publ_flag
Pb1 I1 3.223(2) 4_554 ?
Pb1 I1 3.223(2) 5_554 ?
Pb1 I1 3.223(2) 6_554 ?
Pb1 I1 3.223(2) 2 ?
Pb1 I1 3.223(2) 3 ?
Pb1 I1 3.223(2) . ?
I1 Pb1 3.223(2) 4 ?

loop_
_geom_angle_atom_site_label_1
_geom_angle_atom_site_label_2
_geom_angle_atom_site_label_3
_geom_angle

_atom_sites_solution_secondary ?
_atom_sites_solution_hydrogens .
_refine_ls_hydrogen_treatment
undef
_refine_ls_extinction_method
'SHELXL-2014/7 (Sheldrick 2014'
_refine_ls_extinction_coef
0.014(3)
_refine_ls_extinction_expression

'Fc^*^=kFc[1+0.001xFc^2^\l^3^/si
n(2\q)]^-1/4^'
_refine_ls_abs_structure_details
;
Flack x determined using 183
quotients [(I+)-(I-)]/[(I+)+(I-)]
(Parsons, Flack and Wagner, Acta
Cryst. B69 (2013) 249-259).
;
_refine_ls_abs_structure_Flack
0.39(8)
_chemical_absolute_configuration
?
_refine_ls_number_reflns 513
_refine_ls_number_parameters
11
_refine_ls_number_restraints 1
_refine_ls_R_factor_all
0.0533
_refine_ls_R_factor_gt
0.0494
_refine_ls_wR_factor_ref
0.1655
_refine_ls_wR_factor_gt
0.1591
_refine_ls_goodness_of_fit_ref
1.302
_refine_ls_restrained_S_all
1.301
_refine_ls_shift/su_max 0.000
_refine_ls_shift/su_mean
0.000

loop_
_atom_site_label
_atom_site_type_symbol
_atom_site_fract_x
_atom_site_fract_y
_atom_site_fract_z
_atom_site_U_iso_or_equiv
_atom_site_adp_type
_atom_site_occupancy
_atom_site_site_symmetry_order
_atom_site_calc_flag
_atom_site_refinement_flags_posn
_atom_site_refinement_flags_adp

_atom_site_refinement_flags_occu
pancy
_atom_site_disorder_assembly
_atom_site_disorder_group
Pb1 Pb 0.0000 0.0000 0.5180(3)
0.0454(7) Uani 1 6 d S T P . .
I1 I 0.32891(14) 0.16445(7)
0.7678(3) 0.0830(9) Uani 1 2 d S T
P . .

loop_
_atom_site_aniso_label
_atom_site_aniso_U_11
_atom_site_aniso_U_22
_atom_site_aniso_U_33
_atom_site_aniso_U_23
_atom_site_aniso_U_13
_atom_site_aniso_U_12
Pb1 0.0447(8) 0.0447(8) 0.0469(9)
0.000 0.000 0.0223(4)
I1 0.0384(9) 0.0978(12) 0.0929(14) -
0.0049(4) -0.0097(9) 0.0192(4)

_geom_special_details
;
All esds (except the esd in the
dihedral angle between two l.s.
planes)
are estimated using the full covari-
ance matrix. The cell esds are
taken
into account individually in the
estimation of esds in distances,
angles
and torsion angles; correlations
between esds in cell parameters are
only
used when they are defined by
crystal symmetry. An approximate
(isotropic)
treatment of cell esds is used for
estimating esds involving l.s.
planes.
;

loop_
_geom_bond_atom_site_label_1
_geom_bond_atom_site_label_2
_geom_bond_distance
_geom_bond_site_symmetry_2
_geom_bond_publ_flag
Pb1 I1 3.219(2) 2 ?
Pb1 I1 3.219(2) . ?
Pb1 I1 3.219(2) 3 ?
Pb1 I1 3.221(2) 5_554 ?
Pb1 I1 3.221(2) 4_554 ?
Pb1 I1 3.221(2) 6_554 ?
I1 Pb1 3.221(2) 4 ?

loop_

_geom_angle_site_symmetry_1
_geom_angle_site_symmetry_3
_geom_angle_publ_flag
I1 Pb1 I1 84.04(7) 4_554 5_554 ?
I1 Pb1 I1 84.04(7) 4_554 6_554 ?
I1 Pb1 I1 84.04(7) 5_554 6_554 ?
I1 Pb1 I1 95.96(3) 4_554 2 ?
I1 Pb1 I1 179.99(9) 5_554 2 ?
I1 Pb1 I1 95.96(3) 6_554 2 ?
I1 Pb1 I1 95.96(3) 4_554 3 ?
I1 Pb1 I1 95.96(3) 5_554 3 ?
I1 Pb1 I1 179.99(9) 6_554 3 ?
I1 Pb1 I1 84.03(7) 2 3 ?
I1 Pb1 I1 179.99(10) 4_554 . ?
I1 Pb1 I1 95.96(3) 5_554 . ?
I1 Pb1 I1 95.96(3) 6_554 . ?
I1 Pb1 I1 84.03(7) 2 . ?
I1 Pb1 I1 84.03(7) 3 . ?
Pb1 I1 Pb1 78.77(4) 4 . ?

_refine_diff_density_max 2.653
_refine_diff_density_min -1.239
_refine_diff_density_rms 0.430

_shelx_res_file
;

b.res created by SHELXL-2014/7

TITL b in P6(3)mc
CELL 0.71073 8.7517 8.7517
8.1802 90.000 90.000 120.000
ZERR 2 0.0003 0.0003 0.0005
0.000 0.000 0.000
LATT -1
SYMM -Y, X-Y, Z
SYMM -X+Y, -X, Z
SYMM -X, -Y, 0.5+Z
SYMM Y, -X+Y, 0.5+Z
SYMM X-Y, X, 0.5+Z
SYMM -Y, -X, Z
SYMM -X+Y, Y, Z
SYMM X, X-Y, Z
SYMM Y, X, 0.5+Z
SYMM X-Y, -Y, 0.5+Z
SYMM -X, -X+Y, 0.5+Z
SFAC I PB
UNIT 6 2
LIST 6 ! automatically inserted.
Change 6 to 4 for CHECKCIF!!
TEMP 23

L.S. 8
ACTA
CONF
BOND
FMAP 2
PLAN 20

WGHT 0.145800 1.012600
EXTI 0.038093
FVAR 0.15383
PB1 2 0.000000 0.000000
0.375163 10.16667 0.05861
0.05861 =
0.04999 0.00000 0.00000
0.02930
I1 1 0.328677 0.164338
0.625216 10.50000 0.05239
0.11197 =
0.09538 -0.00349 -0.00699
0.02620
HKLF 4

REM b in P6(3)mc
REM R1 = 0.0624 for 441 Fo >
4sig(Fo) and 0.0700 for all 514
data
REM 11 parameters refined
using 0 restraints

END

WGHT 0.1458 1.0126

REM Highest difference peak
2.653, deepest hole -1.239, 1-sigma
level 0.430
Q1 1 0.0000 0.0000 0.2807
10.16667 0.05 2.49
Q2 1 0.0000 0.0000 0.4703
10.16667 0.05 2.46
Q3 1 0.6667 0.3333 0.9795
10.16667 0.05 2.07
Q4 1 0.6667 0.3333 0.2722
10.16667 0.05 2.03
Q5 1 0.0563 0.1125 0.3723
10.50000 0.05 1.89
Q6 1 -0.0543 -0.1087 0.3751
10.50000 0.05 1.74
Q7 1 0.1591 0.3181 0.6249
10.50000 0.05 1.27
Q8 1 0.3687 -0.2625 0.6253
10.50000 0.05 1.19
Q9 1 0.0627 0.1255 0.2820
10.50000 0.05 1.05
Q10 1 -0.0592 -0.1184 0.4684
10.50000 0.05 1.04
Q11 1 0.0610 0.1220 0.4828
10.50000 0.05 1.01
Q12 1 -0.0579 -0.1157 0.2699
10.50000 0.05 0.95
Q13 1 0.2703 0.1870 0.6263
11.00000 0.05 0.95
Q14 1 0.3401 0.1700 0.7094

_geom_angle_atom_site_label_1
_geom_angle_atom_site_label_2
_geom_angle_atom_site_label_3
_geom_angle
_geom_angle_site_symmetry_1
_geom_angle_site_symmetry_3
_geom_angle_publ_flag
I1 Pb1 I1 84.03(6) 2 . ?
I1 Pb1 I1 84.03(6) 2 3 ?
I1 Pb1 I1 84.03(6) . 3 ?
I1 Pb1 I1 179.96(8) 2 5_554 ?
I1 Pb1 I1 96.004(19) . 5_554 ?
I1 Pb1 I1 96.00(2) 3 5_554 ?
I1 Pb1 I1 96.00(2) 2 4_554 ?
I1 Pb1 I1 179.96(8) . 4_554 ?
I1 Pb1 I1 96.004(19) 3 4_554 ?
I1 Pb1 I1 83.96(6) 5_554 4_554 ?
I1 Pb1 I1 96.00(2) 2 6_554 ?
I1 Pb1 I1 96.00(2) . 6_554 ?
I1 Pb1 I1 179.96(8) 3 6_554 ?
I1 Pb1 I1 83.96(6) 5_554 6_554 ?
I1 Pb1 I1 83.96(6) 4_554 6_554 ?
Pb1 I1 Pb1 78.82(3) . 4 ?

_refine_diff_density_max 2.749
_refine_diff_density_min -0.768
_refine_diff_density_rms 0.321

_shelx_res_file
;

a.res created by SHELXL-2014/7

TITL a in P6(3)mc
CELL 0.71073 8.7339 8.7339
8.1770 90.000 90.000 120.000
ZERR 2 0.0010 0.0010 0.0011
0.000 0.000 0.000
LATT -1
SYMM -Y, X-Y, Z
SYMM -X+Y, -X, Z
SYMM -X, -Y, 0.5+Z
SYMM Y, -X+Y, 0.5+Z
SYMM X-Y, X, 0.5+Z
SYMM -Y, -X, Z
SYMM -X+Y, Y, Z
SYMM X, X-Y, Z
SYMM Y, X, 0.5+Z
SYMM X-Y, -Y, 0.5+Z
SYMM -X, -X+Y, 0.5+Z
SFAC I Pb

UNIT 6 2
LIST 6 ! automatically inserted.
Change 6 to 4 for CHECKCIF!!
TEMP 23.000

L.S. 4
BOND
FMAP 2
PLAN 20
ACTA

WGHT 0.100000
EXTI 0.013813
FVAR 0.12244
PB1 2 0.000000 0.000000
0.518036 10.16667 0.04468
0.04468 =
0.04690 0.00000 0.00000
0.02234
I1 1 0.328908 0.164454
0.767839 10.50000 0.03841
0.09783 =
0.09290 -0.00486 -0.00972
0.01921
HKLF 4

REM a in P6(3)mc
REM R1 = 0.0494 for 461 Fo >
4sig(Fo) and 0.0533 for all 513
data
REM 11 parameters refined
using 0 restraints

END

WGHT 0.0901 1.3853

REM Highest difference peak
2.749, deepest hole -0.768, 1-sigma
level 0.321
Q1 1 0.6667 0.3333 1.1288
10.16667 0.05 2.75
Q2 1 0.6667 0.3333 0.4064
10.16667 0.05 2.71
Q3 1 0.3734 -0.2531 0.7700
10.50000 0.05 1.70
Q4 1 0.2985 0.5971 0.7624
10.50000 0.05 1.59
Q5 1 0.0675 -0.0011 0.5197
11.00000 0.05 1.28
Q6 1 0.0000 0.0000 0.3857
10.16667 0.05 1.10
Q7 1 0.0000 0.0000 0.6594
10.16667 0.05 1.05
Q8 1 0.3584 0.1792 0.8545
10.50000 0.05 0.77
Q9 1 0.2594 0.0761 0.7685
11.00000 0.05 0.76
Q10 1 0.3386 0.1693 0.6534
10.50000 0.05 0.70
Q11 1 0.4306 0.5018 0.7679
11.00000 0.05 0.42
Q12 1 0.1914 -0.1914 0.7038
10.50000 0.05 0.36

```
10.50000 0.05 0.87
Q15 1 -0.0451 -0.0902 0.5585
10.50000 0.05 0.85
Q16 1 0.3384 0.1692 0.5392
10.50000 0.05 0.85
Q17 1 0.0467 0.0934 0.1948
10.50000 0.05 0.84
Q18 1 -0.0404 -0.0808 0.1837
10.50000 0.05 0.79
Q19 1 0.1095 0.2191 0.5498
10.50000 0.05 0.77
Q20 1 0.2015 0.1007 0.6363
10.50000 0.05 0.77
;
_shelx_res_checksum 34015

_shelx_hkl_file
;
1 0 0 660.80 55.24
-2 1 0 120.03 8.65
1 1 0 88.82 8.60
1 -2 0 108.67 8.61
1 -2 0 98.92 8.60
-1 2 0 90.76 8.60
-1 -1 0 96.28 8.60
2 -1 0 105.45 8.64
-2 2 0 4.90 0.50
0 -2 0 5.36 0.45
2 -2 0 4.20 0.52
0 2 0 4.88 0.45
2 -2 0 5.06 0.44
2 -3 0 308.21 25.44
3 -1 0 323.58 25.54
-2 3 0 280.68 25.43
2 1 0 305.75 28.80
-3 2 0 381.42 28.77
3 0 0 124.14 9.86
0 -3 0 116.85 9.67
3 0 0 116.64 9.94
0 3 0 98.10 9.68
3 -3 0 127.68 9.75
3 -3 0 107.76 9.66
3 -4 0 267.89 23.65
1 3 0 276.60 23.69
4 -1 0 274.44 23.89
-3 4 0 291.82 23.72
4 -1 0 304.83 23.84
4 -2 0 1000.00 83.69
-4 3 0 340.82 29.14
3 1 0 363.81 29.25
4 -3 0 339.19 29.23
4 0 0 12.94 1.64
0 -4 0 12.30 1.04
-4 4 0 11.17 1.12
4 -4 0 14.23 1.23
0 4 0 9.92 1.07
1 4 0 27.82 2.44
4 -5 0 27.35 2.38
-4 5 0 30.84 2.49
5 -1 0 28.28 2.65
5 -1 0 25.37 2.75
3 -5 0 222.76 17.01
2 3 0 187.45 17.01
-2 -3 0 221.15 16.98
5 -2 0 194.42 17.13
5 -2 0 186.71 17.21
-3 5 0 202.73 17.01
3 2 0 211.74 19.04
2 -5 0 224.28 18.93
-5 3 0 218.15 18.90
2 -5 0 236.17 18.94
2 -5 0 224.44 18.95
5 -3 0 213.45 19.15
-3 -2 0 259.18 18.96
5 -3 0 220.93 19.07
1 -5 0 33.57 2.78
1 -5 0 33.43 2.77
4 1 0 35.22 3.09
-5 4 0 31.41 2.86
-5 4 0 31.71 2.80
5 -4 0 31.00 3.02
5 -4 0 31.00 2.93
0 -5 0 92.74 8.12
-5 5 0 93.57 8.20
5 0 0 95.52 8.43
0 -5 0 105.77 8.14
5 0 0 99.91 8.54
0 5 0 93.15 8.17
1 5 0 115.10 9.52
-5 6 0 122.30 9.55
6 -1 0 104.29 9.67
6 -1 0 107.85 9.83
4 -6 0 8.12 0.82
4 -6 0 11.00 1.00
2 4 0 8.82 0.94
6 -2 0 6.90 1.39
-2 -4 0 9.91 0.85
-4 6 0 9.68 0.95
6 -2 0 8.35 1.20
3 3 0 6.14 0.89
3 -6 0 8.66 0.80
3 -6 0 8.20 0.75
3 -6 0 5.99 0.72
6 -3 0 6.22 1.04
-3 -3 0 7.75 0.73
-3 6 0 8.62 0.80
4 2 0 11.28 1.36
-6 4 0 10.67 0.93
2 -6 0 10.11 0.98
2 -6 0 10.96 1.01
2 -6 0 10.60 1.08
1 -6 0 138.32 11.97
-6 5 0 166.97 11.95
5 1 0 134.69 12.26
1 -6 0 128.76 11.96
-6 5 0 121.80 12.05
6 -5 0 165.22 12.19
```

```
Q13 1 0.5008 0.4992 1.0133
10.50000 0.05 0.36
Q14 1 0.3564 -0.0267 0.7629
11.00000 0.05 0.34
Q15 1 0.4516 0.2258 0.7657
10.50000 0.05 0.33
Q16 1 0.1916 -0.1916 0.8318
10.50000 0.05 0.31
Q17 1 0.2035 0.2119 0.7704
11.00000 0.05 0.31
Q18 1 0.1648 0.1380 0.4647
11.00000 0.05 0.31
Q19 1 -0.1591 -0.1364 0.5689
11.00000 0.05 0.29
Q20 1 0.5233 0.0467 0.6677
10.50000 0.05 0.27
;
_shelx_res_checksum 95656

_shelx_hkl_file
;
0 0 -1 -0.04 0.06
0 0 -2 188.94 13.38
0 0 2 211.28 13.38
0 0 -3 -0.13 0.15
0 0 3 0.05 0.11
0 0 -4 637.26 46.07
0 0 4 749.45 46.09
0 0 -5 -0.08 0.25
0 0 5 -0.05 0.13
0 0 -6 2.84 0.49
0 0 6 3.70 0.37
0 0 -7 -0.12 0.45
0 0 -8 76.48 5.36
0 0 -9 0.18 0.80
0 0 -10 2.64 1.16
-1 1 -10 5.46 0.49
-1 1 -10 6.43 1.37
-1 1 -9 0.38 0.21
-1 1 -9 -0.20 0.89
-1 1 -8 29.63 2.01
-1 1 -8 26.30 2.32
-1 1 -7 1.41 0.56
-1 1 -7 1.64 0.33
1 -1 6 45.30 2.91
-1 1 -6 40.75 3.06
1 -1 5 7.07 0.55
1 -1 4 216.75 14.54
1 -1 4 225.05 14.53
-1 1 -4 220.29 14.59
-1 1 -4 212.59 14.58
1 -1 3 52.66 3.46
1 -1 3 53.02 3.45
-1 1 -3 50.34 3.47
-1 1 -3 49.87 3.49
1 -1 2 124.26 8.45
1 -1 2 129.03 8.44
-1 1 -2 136.74 8.47
-1 1 -2 131.02 8.47
-1 1 -2 114.35 8.44
1 -1 1 34.23 2.75
1 -1 1 41.44 2.75
1 -1 1 40.33 2.74
1 -1 1 43.11 2.74
-1 1 -1 42.46 2.74
-1 1 -1 41.02 2.75
-1 1 -1 39.74 2.75
-1 1 0 217.51 17.00
-1 1 0 278.84 16.94
-1 1 0 268.38 16.93
1 -1 -1 43.38 2.84
1 -1 -1 35.86 2.83
1 -1 -1 43.29 2.83
-1 1 1 39.78 2.86
-1 1 1 45.71 2.83
1 -1 -2 118.16 8.41
1 -1 -2 140.27 8.43
1 -1 -2 128.73 8.40
1 -1 -2 122.90 8.39
-1 1 2 122.16 8.41
1 -1 -3 14.95 1.11
-1 1 3 18.72 1.26
-1 1 4 71.53 4.88
-1 1 5 9.22 0.85
-1 1 6 33.41 2.64
-1 1 6 39.32 2.56
1 -1 -7 0.93 0.36
-1 1 7 1.27 0.48
-1 1 7 1.47 0.28
1 -1 -8 27.93 2.12
1 -1 -9 0.42 0.54
1 -1 -10 4.42 1.02
-2 2 -10 9.77 1.61
-2 2 -10 7.90 0.78
-2 2 -9 2.18 0.35
-2 2 -9 2.54 1.07
-2 2 -8 6.97 0.72
-2 2 -8 7.93 1.08
-2 2 -7 21.66 1.84
-2 2 -7 22.05 1.60
2 -2 6 137.89 8.76
-2 2 -6 127.91 8.91
-2 2 -6 128.51 8.82
2 -2 5 104.43 6.54
2 -2 5 95.70 6.58
-2 2 -5 100.95 6.60
-2 2 -5 87.96 6.58
-2 2 -5 101.80 6.66
2 -2 4 14.39 0.99
2 -2 4 14.06 1.03
-2 2 -4 15.47 1.07
-2 2 -4 14.77 1.02
-2 2 -4 13.40 1.12
2 -2 3 278.90 18.11
2 -2 3 273.62 18.09
2 -2 3 276.21 18.08
-2 2 -3 280.73 18.12
```

```
0 -6 0 267.08 22.63
-6 6 0 257.53 22.78
6 0 0 247.02 22.87
0 -6 0 309.23 22.66
0 6 0 270.64 22.70
1 6 0 85.47 6.52
7 -1 0 68.84 6.65
7 -1 0 72.33 6.87
2 5 0 45.17 3.81
5 -7 0 41.04 3.67
7 -2 0 40.51 4.21
-5 7 0 46.77 3.82
7 -2 0 43.01 4.02
3 4 0 59.73 5.12
7 -3 0 53.51 5.46
-4 7 0 60.82 5.06
7 -3 0 59.80 5.29
-7 4 0 73.53 5.95
3 -7 0 61.73 5.98
4 3 0 67.25 6.21
3 -7 0 79.00 6.03
3 -7 0 77.91 6.06
7 -4 0 64.13 6.27
2 -7 0 58.37 4.68
5 2 0 56.48 5.06
2 -7 0 58.67 4.73
7 -5 0 50.00 4.96
7 -5 0 49.01 5.16
1 -7 0 104.61 8.08
1 -7 0 93.64 7.95
6 1 0 84.74 8.27
-7 7 0 89.62 7.02
7 -7 0 71.57 7.16
7 -7 0 86.98 7.34
1 7 0 2.27 0.53
-1 -7 0 2.78 0.44
2 6 0 8.16 1.03
6 -8 0 7.94 0.75
8 -2 0 7.34 1.43
5 -8 0 49.27 4.91
3 5 0 63.98 5.13
8 -3 0 61.18 5.36
8 -3 0 60.63 5.66
4 4 0 156.56 12.76
8 -4 0 134.52 13.06
8 -4 0 154.74 12.99
3 -8 0 70.01 6.46
3 -8 0 88.74 6.49
5 3 0 75.45 6.83
8 -5 0 69.46 6.85
6 2 0 9.12 1.76
2 -8 0 10.88 1.03
2 -8 0 9.50 1.12
8 -6 0 12.51 2.21
8 -6 0 11.34 1.89
1 -8 0 2.69 0.53
7 1 0 1.59 0.87
1 -8 0 2.63 0.45
8 -7 0 3.48 1.24
8 -8 0 6.50 1.22
1 8 0 20.62 2.00
-1 -8 0 16.93 1.76
2 7 0 10.03 1.34
6 -9 0 5.53 0.54
9 -3 0 4.53 1.42
9 -3 0 4.14 0.90
5 -9 0 17.14 1.63
9 -4 0 22.76 2.46
9 -4 0 19.70 3.02
4 -9 0 19.61 1.78
5 4 0 24.34 3.28
9 -5 0 14.94 3.20
9 -5 0 22.35 2.65
3 -9 0 9.34 0.93
3 -9 0 7.53 0.88
6 3 0 5.66 1.56
9 -6 0 7.22 1.39
9 -6 0 7.05 1.69
2 -9 0 11.36 1.23
2 -9 0 10.52 1.09
1 -9 0 17.70 1.85
1 -9 0 18.53 1.66
0 -9 0 17.58 1.83
0 -9 0 17.11 1.59
-1 -9 0 14.15 1.63
-1 -9 0 13.21 1.31
2 8 0 30.39 3.02
-2 -8 0 28.43 2.57
3 7 0 15.63 1.80
10 -3 0 9.80 2.08
6 -10 0 1.77 0.28
4 6 0 2.60 0.75
10 -4 0 1.45 0.88
10 -4 0 1.54 1.40
5 5 0 0.35 0.73
10 -5 0 -1.34 0.85
6 4 0 2.01 0.95
4 -10 0 1.13 0.47
10 -6 0 0.67 1.45
10 -6 0 1.81 1.13
3 -10 0 13.96 1.42
7 3 0 12.76 2.56
3 -10 0 16.74 1.85
2 -10 0 28.63 2.78
2 -10 0 31.57 2.69
1 -10 0 14.57 1.35
1 -10 0 13.80 1.57
9 1 0 9.66 2.29
3 8 0 6.15 1.20
-3 -8 0 8.46 0.95
4 7 0 6.05 1.12
11 -4 0 3.03 1.27
11 -4 0 4.08 2.01
5 6 0 4.50 1.10
11 -5 0 2.66 1.37
11 -5 0 2.79 1.90
5 -11 0 7.90 1.10
```

```
-2 2 -3 265.65 18.13
-2 2 -3 258.16 18.10
2 -2 2 544.24 35.58
2 -2 2 551.71 35.60
2 -2 2 535.06 35.51
2 -2 2 504.24 35.53
-2 2 -2 538.27 35.52
-2 2 -2 551.44 35.52
-2 2 -2 519.16 35.51
2 -2 1 478.28 32.99
2 -2 1 478.81 32.97
2 -2 1 490.23 33.00
2 -2 1 473.94 33.00
2 -2 1 508.28 33.07
-2 2 -1 495.94 33.02
-2 2 -1 529.76 33.00
-2 2 -1 498.96 33.02
-2 2 -1 510.77 32.97
2 -2 0 3.57 0.35
2 -2 0 4.47 0.33
2 -2 0 4.19 0.33
2 -2 0 3.80 0.30
2 -2 0 3.59 0.35
-2 2 0 3.92 0.33
-2 2 0 3.53 0.32
2 -2 -1 579.24 35.41
2 -2 -1 492.82 35.37
2 -2 -1 498.08 35.40
2 -2 -1 536.89 35.42
2 -2 -1 534.48 35.43
-2 2 1 514.43 35.39
-2 2 1 529.42 35.41
-2 2 1 563.80 35.41
-2 2 1 541.10 35.37
2 -2 -2 582.30 39.28
2 -2 -2 642.69 39.29
2 -2 -2 595.42 39.30
2 -2 -2 579.71 39.25
2 -2 -2 547.86 39.31
-2 2 2 598.18 39.29
2 -2 -3 260.66 17.91
2 -2 -3 274.71 17.94
2 -2 -3 264.00 17.93
-2 2 3 278.60 17.99
2 -2 -4 13.62 0.95
2 -2 -4 13.80 0.97
2 -2 -4 14.55 0.99
2 -2 -4 14.69 1.06
-2 2 4 13.03 1.16
2 -2 -5 97.65 6.46
2 -2 -5 97.60 6.53
2 -2 -5 99.36 6.44
-2 2 5 92.01 6.61
2 -2 -6 130.98 8.71
2 -2 -7 21.78 1.40
2 -2 -7 21.46 1.61
-2 2 7 22.58 1.89
-2 2 7 16.66 1.62
2 -2 -8 6.48 0.88
2 -2 -8 7.88 0.55
2 -2 -9 2.95 0.64
2 -2 -9 2.50 0.25
2 -2 -10 8.55 1.21
-3 3 -10 6.46 1.68
-3 3 -9 0.16 0.31
-3 3 -9 -0.68 1.10
-3 3 -8 14.51 1.76
-3 3 -8 10.99 1.01
-3 3 -7 0.80 0.68
-3 3 -7 0.09 0.27
3 -3 6 73.37 4.63
-3 3 -6 60.49 4.69
-3 3 -6 73.82 4.86
3 -3 5 0.50 0.20
3 -3 5 0.91 0.14
-3 3 -5 0.53 0.25
-3 3 -5 1.02 0.47
3 -3 4 34.11 2.76
3 -3 4 43.36 2.75
3 -3 4 41.73 2.70
-3 3 -4 44.06 2.84
-3 3 -4 37.63 2.76
3 -3 3 1.43 0.19
3 -3 3 1.04 0.34
3 -3 3 1.63 0.21
3 -3 3 1.36 0.19
-3 3 -3 1.75 0.36
-3 3 -3 1.37 0.24
3 -3 2 286.53 18.85
3 -3 2 292.20 18.84
3 -3 2 287.46 18.85
3 -3 2 284.32 18.91
-3 3 -2 294.65 18.87
-3 3 -2 248.57 18.83
-3 3 -2 289.45 18.86
3 -3 1 1.83 0.22
3 -3 1 1.80 0.26
3 -3 1 1.91 0.23
3 -3 1 1.93 0.23
-3 3 -1 1.68 0.22
-3 3 -1 1.91 0.28
-3 3 -1 1.86 0.27
3 -3 0 79.95 5.72
3 -3 0 82.84 5.73
3 -3 0 92.33 5.71
-3 3 0 82.61 5.71
-3 3 0 86.67 5.74
3 -3 -1 1.55 0.26
3 -3 -1 1.89 0.40
3 -3 -1 1.72 0.22
3 -3 -1 2.34 0.34
-3 3 1 2.15 0.24
-3 3 1 2.08 0.33
-3 3 1 1.58 0.25
3 -3 -2 298.03 19.87
3 -3 -2 299.62 20.00
3 -3 -2 311.71 19.87
```

6	5	0	5.51	1.32
11	-6	0	4.19	2.00
11	-6	0	2.32	1.47
4	-11	0	9.75	1.22
11	-7	0	13.91	2.51
11	-7	0	6.88	1.74
3	-11	0	11.18	1.27
11	-8	0	12.05	2.13
0	0	-1	0.19	0.03
1	-1	-1	53.01	4.64
0	1	-1	48.54	4.63
-1	0	-1	52.66	4.66
-1	0	-1	56.30	4.65
-1	1	1	52.02	4.64
0	-1	1	52.19	4.63
1	0	1	59.56	4.70
1	0	1	56.21	4.65
-1	1	1	57.97	4.64
-1	1	-1	46.59	4.42
1	0	-1	54.48	4.43
1	-1	1	56.39	4.39
-1	0	1	56.59	4.42
0	1	1	48.86	4.39
1	-1	1	46.30	4.41
-1	-1	-1	0.19	0.17
2	-1	-1	0.00	0.16
2	-1	-1	0.39	0.32
1	1	1	0.13	0.17
-2	1	1	0.39	0.25
1	1	1	0.04	0.07
-2	1	1	0.22	0.07
1	-2	1	0.12	0.07
3	-2	-1	60.68	5.44
-3	2	1	58.91	5.27
1	-3	1	64.29	5.29
1	-3	1	60.64	5.28
2	1	1	68.24	5.37
2	0	1	640.92	52.38
2	0	1	610.78	52.37
1	1	-1	0.09	0.07
-2	1	-1	-0.05	0.09
1	-2	-1	0.00	0.09
2	-1	1	-0.08	0.10
-1	2	1	0.16	0.13
-1	2	1	0.15	0.05
-2	2	-1	446.64	37.39
3	-1	-1	57.48	5.27
-2	3	-1	56.38	5.00
3	-1	-1	64.88	5.18
-1	-2	-1	60.26	4.97
1	2	1	53.52	4.97
2	-3	1	57.58	4.96
2	-3	1	62.29	5.00
-2	4	-1	0.22	0.12
4	-2	-1	0.34	0.26
-2	-2	-1	0.61	0.19
-2	-2	-1	0.23	0.10
4	-2	-1	-0.14	0.44
2	-4	1	0.20	0.12
-4	2	1	0.26	0.24
2	2	1	0.39	0.16
2	-4	1	0.03	0.08
5	-3	-1	6.46	1.24
5	-3	-1	6.47	0.98
2	-5	1	7.42	0.70
2	-5	1	7.20	0.69
-5	3	1	7.14	0.64
3	2	1	6.87	0.87
4	-3	-1	61.09	5.64
4	-3	-1	61.95	5.53
3	1	1	67.86	5.57
1	-4	1	60.06	5.39
3	1	1	74.25	5.71
1	-4	1	61.67	5.40
-4	3	1	62.84	5.38
3	-3	-1	1.80	0.43
3	-3	-1	2.54	0.28
0	3	-1	2.07	0.30
3	0	1	2.61	0.57
-3	3	1	2.46	0.31
3	0	1	2.03	0.44
0	-3	1	2.40	0.27
1	2	-1	54.22	4.83
2	-3	-1	49.40	4.81
3	-1	1	59.93	4.93
-1	-2	1	59.61	4.81
3	-1	1	65.00	4.93
-2	3	1	54.13	4.82
1	-3	-1	64.32	5.07
2	1	-1	60.29	5.14
1	-3	-1	60.29	5.13
-3	2	-1	54.98	5.14
1	-3	-1	58.79	5.07
3	-2	1	56.78	5.14
-2	-1	1	64.37	5.10
0	-3	-1	2.86	0.27
-3	3	-1	2.73	0.40
3	0	-1	1.92	0.45
3	-3	1	2.51	0.40
0	3	1	2.23	0.30
4	-1	-1	57.54	4.97
4	-1	-1	52.38	5.11
-1	-3	-1	62.70	4.72
-3	4	-1	54.43	4.78
3	-4	1	49.87	4.68
3	-4	1	59.56	4.76
1	3	1	54.32	4.73
-2	-3	-1	7.68	0.65
5	-2	-1	7.33	1.03
-3	5	-1	7.35	0.74
5	-2	-1	6.26	1.07
3	-5	1	7.23	0.70
2	3	1	6.12	0.70
3	-5	1	5.92	0.62
6	-3	-1	-0.34	0.33
6	-3	-1	0.10	0.63
-3	-3	-1	0.11	0.12

-3	3	2	314.02	19.92
-3	3	2	278.44	19.88
-3	3	2	290.79	19.93
3	-3	-3	0.91	0.12
3	-3	-3	1.26	0.21
3	-3	-3	1.49	0.20
3	-3	-3	1.33	0.28
-3	3	3	0.69	0.18
-3	3	3	1.18	0.29
3	-3	-4	40.47	2.80
3	-3	-4	39.66	2.85
3	-3	-4	42.04	2.87
3	-3	-4	43.79	2.83
-3	3	4	42.69	2.95
3	-3	-5	0.96	0.18
3	-3	-5	0.79	0.14
3	-3	-5	0.84	0.18
-3	3	5	0.63	0.40
3	-3	-6	66.95	4.52
3	-3	-6	64.70	4.63
-3	3	6	71.12	4.72
3	-3	-7	0.17	0.33
3	-3	-7	0.24	0.11
-3	3	7	0.45	0.44
3	-3	-8	10.81	1.15
3	-3	-8	11.49	0.81
3	-3	-9	0.18	0.14
3	-3	-9	-0.27	0.64
3	-3	-10	5.75	0.54
3	-3	-10	6.31	1.12
-4	4	-9	1.16	0.39
-4	4	-8	6.49	1.23
-4	4	-8	5.66	0.75
-4	4	-7	10.21	0.97
-4	4	-7	11.13	1.30
4	-4	6	71.80	4.69
4	-4	6	67.20	4.72
-4	4	-6	65.31	4.89
-4	4	-6	69.73	4.70
4	-4	5	43.12	3.05
4	-4	5	44.74	3.00
-4	4	-5	44.74	3.23
-4	4	-5	44.78	3.08
4	-4	4	10.06	1.22
4	-4	4	12.87	0.95
-4	4	-4	12.80	1.00
4	-4	3	119.59	8.41
4	-4	3	125.65	8.30
4	-4	3	120.86	8.28
4	-4	3	132.29	8.32
-4	4	-3	121.91	8.33
4	-4	2	293.73	20.22
4	-4	2	329.67	20.24
4	-4	2	306.25	20.32
4	-4	2	301.62	20.23
-4	4	-2	290.46	20.24
-4	4	-2	301.34	20.25
-4	4	-2	304.91	20.22
4	-4	1	213.79	15.25
4	-4	1	216.46	15.23
4	-4	1	259.14	15.28
4	-4	1	238.74	15.24
-4	4	-1	221.56	15.25
-4	4	-1	235.03	15.27
-4	4	-1	216.54	15.25
4	-4	0	8.20	0.67
4	-4	0	8.56	0.73
4	-4	0	8.57	0.84
4	-4	0	9.38	0.67
-4	4	0	8.74	0.73
-4	4	0	8.10	0.68
-4	4	0	9.21	0.74
4	-4	-1	212.81	15.08
4	-4	-1	216.28	14.95
4	-4	-1	232.43	14.96
-4	4	1	226.24	14.96
-4	4	1	234.75	15.02
4	-4	-2	311.19	20.96
4	-4	-2	324.18	20.97
-4	4	2	293.21	21.00
-4	4	2	332.61	21.03
4	-4	-3	113.04	8.24
4	-4	-3	132.69	8.26
4	-4	-3	124.34	8.26
-4	4	3	122.62	8.28
-4	4	3	124.68	8.35
4	-4	-4	13.40	0.93
4	-4	-4	12.85	1.05
4	-4	-4	13.29	0.95
4	-4	-4	12.75	1.02
-4	4	4	10.44	1.01
-4	4	4	14.31	1.17
4	-4	-5	47.51	3.07
4	-4	-5	46.31	3.15
4	-4	-5	43.21	3.12
4	-4	-5	43.73	3.10
-4	4	5	46.38	3.25
4	-4	-6	71.95	4.69
4	-4	-6	67.07	4.77
-4	4	6	70.27	4.88
4	-4	-7	10.05	0.74
4	-4	-7	9.36	0.94
4	-4	-8	5.79	0.50
4	-4	-8	5.64	0.77
4	-4	-9	2.08	0.74
4	-4	-9	1.18	0.23
-5	5	-9	-0.28	0.38
-5	5	-8	7.49	0.90
5	-5	7	-0.65	0.67
-5	5	-7	0.14	0.34
5	-5	6	26.29	2.26
5	-5	6	26.53	1.93
-5	5	-6	25.62	1.95
5	-5	5	0.58	0.26
5	-5	5	0.33	0.65
5	-5	5	0.82	0.19
-5	5	-5	1.22	0.35

-3	6	-1	0.29	0.19
-3	-3	-1	-0.01	0.20
-6	3	1	1.06	0.52
3	-6	1	0.23	0.18
3	-6	1	0.08	0.15
3	3	1	0.02	0.22
3	-6	1	0.05	0.16
7	-4	-1	5.91	1.29
3	-7	1	7.98	0.78
4	3	1	6.63	1.04
3	-7	1	6.06	0.73
-7	4	1	7.42	0.66
6	-4	-1	100.53	10.36
6	-4	-1	118.86	10.21
2	-6	1	124.43	9.96
4	2	1	121.43	10.17
2	-6	1	120.15	9.96
-6	4	1	123.04	9.93
5	-4	-1	1.94	0.42
-5	4	1	2.16	0.26
1	-5	1	2.05	0.29
1	-5	1	1.86	0.29
4	1	1	1.28	0.42
4	-4	-1	265.99	21.21
4	-4	-1	270.92	21.42
0	4	-1	238.89	21.23
-4	4	1	237.50	21.24
0	-4	1	290.08	21.22
4	0	1	228.55	21.40
4	0	1	241.50	21.36
3	-4	-1	48.68	4.60
1	3	-1	51.88	4.61
-1	-3	1	61.24	4.60
4	-1	1	53.73	4.71
-3	4	1	55.79	4.62
4	-1	1	54.52	4.77
2	-4	-1	0.16	0.14
2	2	-1	0.18	0.15
-4	2	-1	0.29	0.21
2	-4	-1	0.05	0.12
4	-2	1	0.04	0.17
-2	-2	1	0.03	0.08
4	-2	1	0.42	0.31
-2	4	1	0.06	0.25
-2	4	1	0.01	0.08
1	-4	-1	65.08	5.18
3	1	-1	61.74	5.36
-4	3	-1	58.50	5.15
-4	3	-1	56.92	5.27
1	-4	-1	61.18	5.18
1	-4	-1	65.06	5.27
4	-3	1	61.87	5.29
-4	4	-1	252.12	21.97
4	0	-1	280.02	22.17
0	4	1	253.20	21.90
5	-1	-1	1.67	0.46
5	-1	-1	1.61	0.66
1	4	1	1.71	0.35
4	-5	1	1.72	0.20
4	-5	1	1.60	0.30
6	-2	-1	93.42	8.92
-4	6	-1	106.42	8.57
6	-2	-1	98.36	8.74
2	4	1	103.73	8.54
-4	7	-1	5.71	1.02
7	-3	-1	4.30	0.84
7	-3	-1	3.61	1.05
3	4	1	6.56	0.81
4	-7	1	7.10	0.74
8	-4	-1	0.14	0.66
8	-4	-1	-1.25	0.91
4	4	1	-0.13	0.34
-8	4	1	0.13	0.14
4	-8	1	0.09	0.16
4	-8	1	-0.07	0.22
9	-5	-1	-0.13	0.79
9	-5	-1	-1.21	1.16
4	-9	1	-0.01	0.18
5	4	1	-0.72	0.46
4	-9	1	-0.03	0.24
-8	5	1	17.12	1.37
5	3	1	12.93	1.81
3	-8	1	17.37	1.46
3	-8	1	13.11	1.41
7	-5	-1	-1.29	0.86
7	-5	-1	-0.19	0.50
5	2	1	1.35	0.51
2	-7	1	0.71	0.20
6	-5	-1	32.72	3.23
1	-6	1	36.14	2.93
-6	5	1	33.90	3.02
1	-6	1	33.92	2.92
5	1	1	33.29	3.20
5	-5	-1	3.28	0.42
5	-5	-1	3.05	0.71
0	5	-1	3.09	0.49
0	-5	1	3.53	0.38
5	0	1	2.35	0.51
0	-5	1	2.97	0.36
-5	5	1	4.12	0.50
5	0	1	2.82	0.65
4	-5	-1	1.55	0.27
4	-5	-1	2.15	0.44
1	4	-1	1.93	0.37
-1	-4	1	1.87	0.27
5	-1	1	2.04	0.52
-4	5	1	1.72	0.34
5	-1	1	1.19	0.35
3	-5	-1	5.15	0.57
2	3	-1	6.06	0.68
-3	5	1	6.29	0.63
5	-2	1	6.21	1.03
5	-2	1	7.20	0.84
2	-5	-1	6.77	0.78
-5	3	-1	7.87	0.90
2	-5	-1	7.86	0.75
2	-5	-1	7.66	0.70

5	-5	4	40.83	2.95
5	-5	4	43.56	3.00
5	-5	4	46.63	3.27
-5	5	-4	43.40	3.03
5	-5	3	2.21	0.31
5	-5	3	2.21	0.64
5	-5	3	2.41	0.35
5	-5	3	2.00	0.31
-5	5	-3	2.33	0.33
5	-5	2	79.01	5.44
5	-5	2	81.65	5.43
5	-5	2	80.68	5.42
-5	5	-2	81.49	5.47
-5	5	-2	79.38	5.45
5	-5	1	3.05	0.59
5	-5	1	2.93	0.46
5	-5	1	3.40	0.38
-5	5	-1	2.75	0.36
-5	5	-1	2.80	0.44
-5	5	-1	2.65	0.39
5	-5	0	82.23	5.57
5	-5	0	87.85	5.67
5	-5	0	79.95	5.61
-5	5	0	85.48	5.63
-5	5	0	78.77	5.58
-5	5	0	82.48	5.62
5	-5	-1	2.69	0.35
5	-5	-1	2.75	0.29
5	-5	-1	2.54	0.48
5	-5	-1	2.78	0.36
5	-5	-1	2.94	0.64
-5	5	1	2.54	0.40
-5	5	1	2.94	0.34
-5	5	1	2.22	0.38
5	-5	-2	79.74	5.27
5	-5	-2	82.86	5.28
5	-5	-2	69.90	5.50
5	-5	-2	75.16	5.25
-5	5	2	79.74	5.34
-5	5	2	81.95	5.37
5	-5	-3	1.12	0.55
5	-5	-3	2.96	0.34
5	-5	-3	1.73	0.27
5	-5	-3	2.00	0.28
-5	5	3	1.91	0.40
-5	5	3	1.82	0.38
5	-5	-4	44.45	2.88
5	-5	-4	42.41	2.85
5	-5	-4	41.10	2.87
-5	5	4	39.19	2.93
-5	5	4	41.16	3.03
5	-5	-5	0.81	0.31
5	-5	-5	0.90	0.21
5	-5	-5	0.82	0.23
-5	5	5	1.27	0.48
-5	5	5	1.23	0.39
5	-5	-6	26.62	1.85
5	-5	-6	25.88	1.93
-5	5	6	23.93	1.97
-5	5	6	30.10	2.14
5	-5	-7	0.36	0.33
5	-5	-7	0.18	0.18
5	-5	-8	7.89	1.02
5	-5	-8	6.21	0.69
5	-5	-9	1.04	0.72
6	-6	8	14.43	2.19
6	-6	7	0.11	0.97
6	-6	6	1.18	0.35
6	-6	6	0.60	0.95
6	-6	5	0.11	0.31
6	-6	5	0.08	0.26
6	-6	5	-0.56	0.83
6	-6	4	116.00	8.11
6	-6	4	114.81	8.07
6	-6	4	131.50	8.38
6	-6	3	0.17	0.57
6	-6	3	0.03	0.19
6	-6	3	0.20	0.24
-6	6	-3	0.18	0.27
6	-6	2	2.51	0.36
6	-6	2	3.79	0.74
-6	6	-2	2.27	0.40
-6	6	-2	2.58	0.46
6	-6	1	0.27	0.24
6	-6	1	0.03	0.53
-6	6	-1	0.04	0.26
-6	6	-1	0.54	0.36
6	-6	0	256.37	18.23
6	-6	0	286.36	18.36
6	-6	0	292.69	18.32
-6	6	0	257.51	18.27
-6	6	0	274.29	18.40
6	-6	-1	0.53	0.46
6	-6	-1	0.79	0.57
6	-6	-1	0.35	0.23
-6	6	1	0.13	0.36
6	-6	-2	2.36	0.33
6	-6	-2	2.69	0.34
6	-6	-2	2.97	0.77
-6	6	2	1.88	0.42
6	-6	-3	0.30	0.53
6	-6	-3	0.32	0.21
6	-6	-3	0.69	0.23
-6	6	3	0.08	0.42
6	-6	-4	121.35	7.75
6	-6	-4	115.40	7.77
-6	6	4	109.19	7.84
6	-6	-5	-0.03	0.26
6	-6	-5	0.15	0.22
-6	6	5	2.44	0.53
6	-6	-6	1.11	0.33
-6	6	6	0.88	0.49
6	-6	-7	-0.13	0.35
6	-6	-8	13.48	1.41
7	-7	7	-0.42	1.29
7	-7	6	7.91	0.91
7	-7	6	7.82	1.50

3	2	-1	7.28	0.89
5	-3	1	6.82	0.89
1	-5	-1	2.18	0.46
1	-5	-1	1.75	0.28
1	-5	-1	2.18	0.32
4	1	-1	1.31	0.48
-5	4	-1	2.55	0.23
-4	-1	1	2.63	0.51
5	-4	1	1.46	0.39
-4	-1	1	2.88	0.43
-5	5	-1	2.96	0.59
0	-5	-1	3.28	0.37
5	0	-1	5.02	1.00
0	-5	-1	3.86	0.39
5	-5	1	4.20	0.68
0	5	1	2.89	0.47
-5	6	-1	31.39	2.95
-1	-5	-1	39.34	2.83
6	-1	-1	31.58	3.49
6	-1	-1	28.13	3.11
1	5	1	32.28	2.87
-2	-5	-1	0.70	0.17
7	-2	-1	-0.45	0.69
7	-2	-1	0.72	0.45
5	-7	1	0.95	0.16
5	-7	1	0.63	0.27
2	5	1	0.65	0.26
8	-3	-1	9.97	1.74
8	-3	-1	10.18	1.70
5	-8	1	9.54	0.92
3	5	1	12.46	1.22
9	-4	-1	-1.62	1.18
9	-4	-1	-0.48	0.65
5	-9	1	-0.06	0.12
5	-9	1	0.59	0.31
4	5	1	-0.15	0.38
10	-5	-1	-1.20	0.82
5	-10	1	-0.15	0.32
5	-10	1	-0.05	0.16
5	5	1	-0.38	0.60
11	-6	-1	-1.10	1.34
11	-6	-1	-3.15	2.12
6	5	1	0.14	0.86
5	-11	1	0.35	0.33
10	-6	-1	1.27	1.81
10	-6	-1	3.30	1.34
6	4	1	3.84	0.97
4	-10	1	3.67	0.53
9	-6	-1	-1.61	1.32
9	-6	-1	-0.27	0.87
3	-9	1	0.28	0.27
3	-9	1	0.51	0.25
-9	6	1	0.38	0.25
6	3	1	0.04	1.01
8	-6	-1	22.34	3.80
8	-6	-1	28.97	3.17
2	-8	1	30.80	2.53
2	-8	1	29.20	2.47
6	2	1	25.54	2.90
7	-6	-1	10.01	1.56
7	-6	-1	9.43	1.54
6	1	1	6.27	1.22
1	-7	1	7.64	0.77
0	6	-1	0.27	0.21
6	-6	-1	0.98	0.40
6	-6	-1	-0.71	0.62
-6	6	1	0.81	0.22
6	0	1	0.35	0.48
0	-6	1	0.27	0.12
6	0	1	0.25	0.33
-6	6	1	0.47	0.25
5	-6	-1	66.19	6.05
-5	6	1	71.29	5.87
2	4	-1	100.69	8.32
4	-6	-1	87.20	8.23
6	-2	1	102.40	8.45
6	-2	1	96.10	8.53
-4	6	1	103.99	8.30
3	-6	-1	0.22	0.13
3	3	-1	0.34	0.25
-6	3	-1	0.52	0.47
3	-6	-1	-0.13	0.20
3	-6	-1	0.13	0.17
6	-3	1	0.20	0.31
6	-3	1	-0.29	0.39
-3	6	1	-0.08	0.16
4	2	-1	122.39	10.58
-6	4	-1	116.88	10.47
2	-6	-1	126.80	10.32
2	-6	-1	121.19	10.41
-6	4	-1	122.03	10.29
2	-6	-1	140.64	10.35
6	-4	1	120.39	10.57
6	-4	1	112.11	10.47
1	-6	-1	40.31	3.66
1	-6	-1	40.31	3.50
5	1	-1	37.25	3.91
1	-6	-1	38.21	3.49
6	-5	1	49.41	3.70
6	0	-1	-0.01	0.49
0	-6	-1	0.84	0.44
0	-6	-1	0.28	0.12
0	-6	-1	0.29	0.13
6	-6	1	0.64	0.37
0	6	1	0.36	0.21
-1	-6	-1	8.48	0.79
7	-1	-1	6.10	1.29
1	6	1	8.01	0.92
2	6	1	26.30	2.43
9	-3	-1	-0.56	1.10
9	-3	-1	-0.22	0.64
6	-9	1	0.26	0.17
3	6	1	0.49	0.40
6	-9	1	0.41	0.47
10	-4	-1	1.58	1.39
10	-4	-1	3.24	1.04
4	6	1	4.29	0.80

7	-7	5	0.22	1.05
7	-7	5	0.42	0.31
7	-7	4	22.93	2.13
7	-7	4	18.55	1.52
7	-7	3	0.90	0.72
7	-7	3	1.26	0.33
-7	7	-3	0.81	0.35
7	-7	2	24.39	1.85
7	-7	2	27.50	2.15
-7	7	-2	24.53	1.93
7	-7	1	1.26	0.57
7	-7	1	1.23	0.30
-7	7	-1	0.99	0.46
7	-7	0	37.31	2.70
7	-7	0	35.57	2.65
7	-7	0	32.69	2.46
-7	7	0	34.42	2.55
7	-7	-1	0.84	0.29
7	-7	-1	1.06	0.64
-7	7	1	1.33	0.40
7	-7	-2	26.90	2.23
7	-7	-2	23.89	1.77
-7	7	2	23.31	1.90
7	-7	-3	0.74	0.28
7	-7	-3	1.30	0.65
-7	7	3	1.65	0.51
7	-7	-4	19.62	1.54
-7	7	4	21.87	1.84
7	-7	-5	0.71	0.26
-7	7	5	0.66	0.60
7	-7	-6	7.65	0.79
-7	7	6	8.08	1.02
7	-7	-7	0.33	0.44
-7	7	7	0.73	0.69
8	-8	6	9.27	1.80
8	-8	6	10.22	1.09
8	-8	5	2.08	0.45
8	-8	5	1.56	1.01
8	-8	4	1.72	0.39
8	-8	4	1.46	1.11
8	-8	3	5.64	0.71
8	-8	3	7.05	1.20
-8	8	-3	6.36	0.85
8	-8	2	41.91	3.22
8	-8	2	41.65	2.97
8	-8	1	11.03	1.38
8	-8	1	9.90	0.94
8	-8	1	11.85	1.23
-8	8	-1	10.42	1.12
8	-8	0	1.90	0.38
8	-8	0	2.84	0.66
8	-8	0	2.79	0.71
-8	8	0	2.16	0.53
8	-8	-1	13.01	1.50
8	-8	-1	9.87	0.95
-8	8	1	9.85	1.13
8	-8	-2	44.23	3.16
8	-8	-2	34.64	2.66
-8	8	2	35.78	2.83
8	-8	-3	6.71	0.72
-8	8	3	5.51	0.80
8	-8	-4	2.30	0.39
-8	8	4	2.38	0.74
8	-8	-5	2.16	0.40
-8	8	5	2.19	0.80
-8	8	6	10.58	1.68
9	-9	4	8.28	0.97
9	-9	4	9.32	1.56
9	-9	3	-0.91	0.99
9	-9	3	0.30	0.34
9	-9	2	3.26	0.97
9	-9	2	2.75	0.67
9	-9	2	2.97	0.49
9	-9	1	0.54	0.34
9	-9	1	0.23	0.57
9	-9	1	-0.40	0.58
9	-9	0	17.46	1.75
9	-9	0	16.27	1.35
-9	9	0	15.27	1.53
9	-9	-1	0.22	0.32
9	-9	-1	-0.03	1.08
-9	9	1	0.36	0.63
9	-9	-2	2.37	0.43
-9	9	2	2.37	0.79
9	-9	-3	0.14	0.30
-9	9	3	-0.33	0.69
9	-9	-4	8.73	0.91
-9	9	4	7.74	1.06
10	-10	1	2.62	0.51
10	-10	0	1.84	0.47
10	-10	-1	1.85	0.46
-10	10	1	2.90	0.89
-9	10	3	0.80	0.78
9	-10	-3	0.81	0.39
-9	10	2	0.71	0.72
9	-10	-2	0.95	0.37
-9	10	1	1.47	0.73
9	-10	-1	1.99	0.45
-9	10	0	13.41	1.46
9	-10	0	12.72	1.16
9	-10	1	2.03	0.71
9	-10	1	1.05	0.40
9	-10	2	1.08	0.40
9	-10	2	0.73	0.93
9	-10	3	0.55	1.08
9	-10	3	0.76	0.40
-8	9	5	1.28	0.76
8	-9	-5	1.06	0.37
-8	9	4	7.77	1.12
8	-9	-4	8.75	0.87
-8	9	3	2.82	0.71
8	-9	-3	2.10	0.41
-8	9	2	6.12	0.91
8	-9	-2	7.53	0.76
-8	9	1	3.50	0.66
8	-9	-1	3.52	0.49
-8	9	0	15.53	1.47

11	-5	-1	-0.88	1.26
11	-5	-1	-2.88	1.91
6	-11	1	0.86	0.40
5	6	1	-0.30	0.73
11	-7	-1	-3.20	2.22
11	-7	-1	-2.25	1.48
4	-11	1	-0.05	0.29
7	4	1	-0.33	0.83
10	-7	-1	2.96	1.30
10	-7	-1	2.19	1.78
3	-10	1	3.91	0.65
7	3	1	2.64	0.86
9	-7	-1	-0.70	0.86
7	2	1	-0.87	0.53
2	-9	1	0.26	0.19
2	-9	1	0.61	0.36
8	-7	-1	0.72	0.74
8	-7	-1	-0.11	1.21
1	-8	1	0.39	0.17
7	1	1	-0.03	0.45
0	7	-1	1.10	0.32
7	-7	-1	1.88	0.98
0	-7	1	0.84	0.18
7	0	1	0.56	0.41
6	-7	-1	7.05	0.69
1	6	-1	7.11	0.86
7	-1	1	6.77	1.33
7	-1	1	5.48	1.00
5	-7	-1	0.82	0.20
5	-7	-1	0.94	0.44
2	5	-1	0.56	0.27
7	-2	1	0.38	0.60
-5	7	1	0.61	0.25
7	-2	1	0.51	0.36
3	4	-1	6.40	0.85
4	-7	-1	4.44	0.53
7	-3	1	6.37	1.04
7	-3	1	3.87	0.92
-4	7	1	5.79	0.66
3	-7	-1	8.07	0.78
3	-7	-1	8.39	0.89
4	3	-1	7.13	1.17
-7	4	-1	7.96	0.70
7	-4	1	4.93	1.20
5	2	-1	0.78	0.56
2	-7	-1	0.97	0.24
2	-7	-1	1.15	0.40
-7	5	-1	1.14	0.18
2	-7	-1	0.71	0.18
7	-5	1	0.55	0.60
7	-5	1	1.94	0.51
6	1	-1	6.00	1.18
1	-7	-1	9.11	1.12
1	-7	-1	8.64	0.81
1	-7	-1	8.34	0.81
0	-7	-1	2.06	0.54
0	-7	-1	1.17	0.21
7	0	-1	0.46	0.54
7	-7	1	1.50	0.50
-1	-7	-1	0.30	0.15
8	-1	-1	0.37	0.49
1	7	1	0.57	0.32
7	-8	1	0.39	0.54
9	-2	-1	0.64	0.58
2	7	1	0.95	0.44
7	-9	1	0.07	0.44
10	-3	-1	1.78	0.88
3	7	1	3.46	0.70
11	-4	-1	-1.58	1.06
4	7	1	0.60	0.72
7	-11	1	0.72	0.47
11	-8	-1	1.21	2.28
11	-8	-1	-0.45	1.64
3	-11	1	0.14	0.42
8	3	1	-1.13	0.90
3	-11	1	0.54	0.34
10	-8	-1	0.06	1.14
2	-10	1	0.41	0.39
8	2	1	-0.33	0.87
2	-10	1	0.29	0.23
9	-8	-1	7.49	2.39
1	-9	1	3.77	0.57
0	-8	1	10.28	1.00
7	-8	-1	0.72	0.83
1	7	-1	0.60	0.51
8	-1	1	-0.03	0.41
-7	8	1	1.18	0.42
-1	-7	1	0.29	0.16
8	-1	1	-0.77	0.60
2	6	-1	24.52	2.23
6	-8	-1	22.71	2.01
8	-2	1	21.82	2.40
8	-2	1	23.44	3.31
5	-8	-1	8.80	0.98
3	5	-1	12.38	1.36
8	-3	1	11.31	1.58
-5	8	1	12.27	1.27
8	-3	1	10.75	2.02
4	-8	-1	0.09	0.31
4	4	-1	0.51	0.46
4	-8	-1	-0.24	0.16
8	-4	1	-0.40	0.43
8	-4	1	-1.36	0.80
3	-8	-1	19.27	1.77
5	3	-1	18.41	2.35
-8	5	-1	20.06	1.63
8	-5	1	13.70	2.47
2	-8	-1	30.76	3.03
2	-8	-1	36.80	2.97
1	-8	-1	0.42	0.20
7	1	-1	0.56	0.70
1	-8	-1	0.84	0.50
8	-7	1	0.41	0.62
0	-8	-1	10.27	1.35
8	0	-1	7.90	1.74
0	-8	-1	11.17	1.00
-1	-8	-1	6.88	1.11

8	-9	0	16.10	1.33
8	-9	0	18.04	1.66
8	-9	1	4.64	0.64
8	-9	1	5.01	0.81
8	-9	2	8.25	1.10
8	-9	2	6.89	0.78
8	-9	3	2.36	0.98
8	-9	3	2.32	0.43
8	-9	4	8.51	1.37
8	-9	4	8.31	0.88
8	-9	5	0.89	0.40
8	-9	5	1.75	0.98
-7	8	7	0.53	0.67
7	-8	-7	0.40	0.51
-7	8	6	14.18	1.62
-7	8	5	0.38	0.61
7	-8	-5	0.23	0.26
-7	8	4	2.23	0.64
7	-8	-4	2.62	0.40
-7	8	3	0.83	0.40
7	-8	-3	0.36	0.27
-7	8	2	55.05	3.72
7	-8	-2	50.03	3.61
-7	8	1	0.57	0.36
7	-8	-1	0.33	0.26
7	-8	-1	1.06	0.63
-7	8	0	1.87	0.45
7	-8	0	2.47	0.65
7	-8	0	2.21	0.58
7	-8	0	1.48	0.32
-7	8	-1	0.10	0.42
7	-8	1	0.25	0.50
7	-8	1	0.48	0.26
-7	8	-2	51.75	3.76
7	-8	2	59.64	3.88
7	-8	2	48.61	3.65
-7	8	-3	0.08	0.35
7	-8	3	1.15	0.72
7	-8	3	0.59	0.30
7	-8	4	2.02	0.34
7	-8	4	3.45	0.83
7	-8	4	1.82	0.37
7	-8	5	0.10	0.32
7	-8	5	0.90	0.92
7	-8	6	13.69	1.20
7	-8	6	12.23	1.77
7	-8	7	0.14	1.35
6	-7	-8	6.33	0.88
-6	7	7	0.27	0.56
6	-7	-7	0.68	0.48
-6	7	6	7.29	1.12
6	-7	-6	7.09	0.78
-6	7	5	1.40	0.53
6	-7	-5	1.91	0.34
6	-7	-5	1.86	0.32
-6	7	4	41.32	2.92
6	-7	-4	40.24	2.78
6	-7	-4	38.29	2.77
-6	7	3	3.94	0.56
6	-7	-3	3.49	0.47
6	-7	-3	3.75	0.52
-6	7	2	12.35	1.12
6	-7	-2	12.92	1.01
6	-7	-2	13.47	1.10
6	-7	-2	14.28	1.48
-6	7	1	6.18	0.77
6	-7	-1	5.78	0.59
6	-7	-1	8.37	1.05
6	-7	-1	6.66	0.81
-6	7	0	76.02	5.16
6	-7	0	70.85	5.09
6	-7	0	78.97	5.24
-6	7	-1	7.17	0.79
-6	7	-1	6.71	0.82
6	-7	1	6.37	0.64
6	-7	1	7.56	1.09
6	-7	1	6.72	0.63
-6	7	-2	13.83	1.15
6	-7	2	12.95	1.04
6	-7	2	12.80	1.03
6	-7	2	14.16	1.34
-6	7	-3	3.40	0.50
6	-7	3	4.46	0.74
6	-7	3	4.11	0.52
6	-7	3	3.37	0.48
6	-7	4	39.84	2.91
6	-7	4	42.68	2.95
6	-7	4	45.23	3.34
6	-7	5	0.91	0.91
6	-7	5	1.02	0.25
6	-7	5	1.19	0.32
6	-7	6	5.87	0.73
6	-7	6	6.59	1.22
6	-7	7	0.54	1.08
6	-7	8	8.11	1.47
5	-6	-8	8.43	1.00
5	-6	-7	0.98	0.43
-5	6	6	14.55	1.22
5	-6	-6	12.96	1.13
-5	6	5	6.30	0.83
5	-6	-5	6.51	0.60
5	-6	-5	6.55	0.69
-5	6	4	51.46	3.74
5	-6	-4	55.53	3.69
5	-6	-4	53.96	3.66
-5	6	3	16.83	1.32
5	-6	-3	16.30	1.21
5	-6	-3	13.54	1.44
5	-6	-3	17.20	1.19
5	-6	-2	30.25	2.45
5	-6	-2	30.79	2.12
-5	6	1	27.94	2.07
5	-6	-1	29.05	1.99
5	-6	-1	27.99	2.08
5	-6	-1	25.81	2.16
5	-6	-1	32.32	2.24
-5	6	0	114.01	7.26

-1	-8	-1	4.44	0.61
1	8	1	5.76	1.10
-2	-8	-1	0.68	0.60
10	-2	-1	-0.29	0.78
8	-10	1	-0.24	0.54
2	8	1	0.75	0.56
11	-3	-1	-0.66	0.99
-3	-8	-1	0.49	0.31
8	-11	1	1.10	0.65
3	8	1	-0.05	0.74
10	-9	-1	2.77	1.31
10	-9	-1	3.71	1.83
1	-10	1	1.36	0.30
1	-10	1	1.53	0.54
9	1	1	1.04	0.76
9	-9	-1	-1.00	1.29
9	-9	-1	0.58	0.83
9	0	1	-0.59	0.53
0	-9	1	0.46	0.21
9	-1	1	2.54	0.65
-1	-8	1	3.68	0.51
7	-9	-1	0.13	0.78
2	7	-1	1.16	0.47
7	-9	-1	0.57	0.25
9	-2	1	-0.24	0.50
9	-2	1	-0.84	0.69
6	-9	-1	0.40	0.18
3	6	-1	0.69	0.48
9	-3	1	0.24	0.56
4	5	-1	-0.20	0.51
5	-9	-1	0.32	0.40
5	-9	-1	-0.09	0.17
9	-4	1	-0.85	0.89
9	-4	1	-0.22	0.65
4	-9	-1	-0.07	0.19
-9	5	-1	0.50	0.20
4	-9	-1	-0.09	0.44
5	4	-1	-0.27	0.67
9	-5	1	-0.53	0.73
9	-5	1	-0.91	0.97
3	-9	-1	0.34	0.31
6	3	-1	0.45	0.82
3	-9	-1	0.19	0.27
9	-6	1	-0.10	0.79
9	-6	1	-1.08	0.93
2	-9	-1	0.39	0.45
2	-9	-1	0.29	0.24
7	2	-1	0.89	0.82
9	-7	1	0.24	0.65
1	-9	-1	5.83	1.05
8	1	-1	2.47	0.98
1	-9	-1	4.55	0.63
0	-9	-1	0.63	0.53
9	0	-1	-0.43	0.69
0	-9	-1	0.55	0.23
9	-9	1	0.32	0.80
9	-10	1	2.90	0.92
9	-10	-1	3.32	0.99
9	-10	-1	2.66	1.42
-1	-9	1	1.10	0.27
10	-1	1	0.67	0.69
2	8	-1	0.49	0.64
8	-10	-1	0.73	1.00
10	-2	1	0.76	1.08
10	-2	1	-0.03	0.65
3	7	-1	3.83	0.82
7	-10	-1	3.04	0.45
10	-3	1	1.30	1.21
10	-3	1	1.90	0.81
6	-10	-1	3.86	0.73
4	6	-1	4.16	0.93
10	-4	1	1.54	1.34
10	-4	1	3.62	1.02
5	-10	-1	-0.02	0.22
5	5	-1	-0.75	0.77
5	-10	-1	0.14	0.46
10	-5	1	-1.09	1.31
10	-5	1	-0.77	0.85
4	-10	-1	4.64	0.71
6	4	-1	4.20	1.31
10	-6	1	3.44	1.13
10	-6	1	2.90	1.76
3	-10	-1	3.24	0.56
7	3	-1	2.29	1.21
10	-7	1	4.67	1.12
2	-10	-1	0.41	0.31
2	-10	-1	0.27	0.50
8	2	-1	-0.06	1.03
10	-8	1	0.51	0.89
10	-8	1	-0.64	0.99
9	1	-1	0.14	0.94
1	-10	-1	1.73	0.66
1	-10	-1	1.54	0.36
10	-9	1	2.90	1.22
10	-9	1	3.21	0.99
3	8	-1	0.64	0.84
8	-11	-1	1.88	1.03
11	-3	1	-1.99	1.43
-3	-8	1	-0.01	0.28
11	-3	1	-1.02	0.92
4	7	-1	0.71	0.98
7	-11	-1	-0.08	0.80
11	-4	1	-1.67	1.50
11	-4	1	-1.54	0.93
5	6	-1	-0.59	1.00
6	-11	-1	0.99	0.65
11	-5	1	-0.16	1.10
11	-5	1	-0.41	1.59
5	-11	-1	0.19	0.49
6	5	-1	0.51	1.23
5	-11	-1	0.29	0.46
11	-6	1	-2.10	1.47
11	-6	1	-0.96	1.28
4	-11	-1	0.50	0.46
7	4	-1	0.05	1.33
4	-11	-1	0.13	0.32
11	-7	1	-0.94	1.21

-5	6	0	109.06	7.24
5	-6	0	110.57	7.30
5	-6	0	105.20	7.21
5	-6	0	99.52	7.23
5	-6	0	105.26	7.19
-5	6	-1	28.23	2.06
-5	6	-1	28.17	2.06
5	-6	1	27.86	2.01
5	-6	1	28.11	2.00
5	-6	1	31.27	2.21
-5	6	-2	32.06	2.25
-5	6	-2	32.51	2.29
-5	6	-2	30.58	2.26
5	-6	2	33.79	2.23
5	-6	2	30.93	2.22
-5	6	-3	15.49	1.28
-5	6	-3	16.39	1.31
5	-6	3	18.99	1.63
5	-6	3	17.92	1.25
5	-6	3	17.00	1.24
-5	6	-4	53.33	3.72
5	-6	4	56.78	3.96
5	-6	4	52.73	3.67
5	-6	4	53.69	3.70
-5	6	-5	6.41	0.70
5	-6	5	5.94	0.57
5	-6	5	6.96	0.66
5	-6	5	6.30	0.97
-5	6	-6	13.19	1.14
5	-6	6	13.83	1.15
5	-6	6	15.25	1.75
-5	6	-7	1.25	0.37
5	-6	7	1.13	0.85
-5	6	-8	7.30	0.84
5	-6	8	8.06	1.21
-5	6	-9	0.06	0.37
4	-5	-9	0.11	0.72
4	-5	-9	-0.03	0.18
4	-5	-8	6.41	0.55
4	-5	-8	5.87	0.88
4	-5	-7	0.12	0.34
4	-5	-7	0.28	0.15
4	-5	-6	44.65	3.27
4	-5	-6	48.53	3.17
-4	5	5	0.75	0.30
4	-5	-5	0.33	0.32
4	-5	-5	1.04	0.21
-4	5	4	18.53	1.34
4	-5	-4	17.94	1.33
4	-5	-4	17.73	1.26
4	-5	-4	15.79	1.31
4	-5	-4	18.09	1.25
-4	5	3	0.98	0.28
-4	5	3	1.11	0.30
4	-5	-3	1.21	0.19
4	-5	-3	0.84	0.18
4	-5	-3	0.80	0.26
4	-5	-3	0.11	0.38
-4	5	2	204.64	13.39
-4	5	2	210.91	13.38
4	-5	-2	217.61	13.34
4	-5	-2	171.31	13.48
4	-5	-2	195.65	13.34
-4	5	1	0.67	0.19
-4	5	1	1.13	0.26
4	-5	-1	1.10	0.37
4	-5	-1	1.46	0.19
4	-5	-1	1.40	0.28
4	-5	-1	0.97	0.16
-4	5	0	25.31	1.86
-4	5	0	25.23	1.83
4	-5	0	28.04	1.83
4	-5	0	26.50	1.82
4	-5	0	25.31	1.86
4	-5	0	27.13	1.95
4	-5	0	26.52	1.84
-4	5	-1	1.43	0.23
-4	5	-1	1.31	0.28
-4	5	-1	1.39	0.26
4	-5	1	1.37	0.39
4	-5	1	0.98	0.20
-4	5	-2	198.45	13.32
-4	5	-2	190.60	13.30
-4	5	-2	183.80	13.29
4	-5	2	203.76	13.28
4	-5	2	197.88	13.30
4	-5	2	218.42	13.29
4	-5	2	201.78	13.38
-4	5	-3	0.99	0.25
4	-5	3	0.68	0.18
4	-5	3	1.52	0.23
4	-5	3	1.26	0.50
4	-5	3	1.02	0.20
-4	5	-4	18.22	1.39
4	-5	4	18.74	1.37
4	-5	4	19.45	1.34
4	-5	4	19.77	1.65
-4	5	-5	0.30	0.24
4	-5	5	0.66	0.18
4	-5	5	0.54	0.23
4	-5	5	0.24	0.54
-4	5	-6	47.13	3.25
4	-5	6	48.92	3.26
4	-5	6	47.78	3.24
4	-5	6	43.86	3.42
-4	5	-7	-0.07	0.26
4	-5	7	-0.87	0.64
-4	5	-8	6.34	0.71
-4	5	-9	0.09	0.29
3	-4	-10	1.89	0.98
3	-4	-10	2.96	0.40
3	-4	-9	0.04	0.69
3	-4	-9	0.26	0.16
3	-4	-8	19.16	1.33
3	-4	-8	18.64	1.67
3	-4	-7	1.67	0.21
3	-4	-7	1.28	0.38

11	-7	1	-0.89	1.51
8	3	-1	-0.97	1.26
3	-11	-1	0.08	0.41
11	-8	1	-0.24	1.64
11	-8	1	-1.50	1.21
1	-1	-2	157.13	14.26
-1	1	2	184.09	14.26
0	-1	2	151.22	14.25
1	0	2	189.15	14.27
-1	1	-2	150.06	12.59
1	0	-2	146.36	12.58
1	-1	2	149.25	12.59
1	-1	2	150.87	12.57
-1	0	2	155.46	12.58
-1	0	2	150.01	12.58
2	-1	-2	718.43	52.06
-1	-1	-2	524.38	51.93
-1	-1	-2	623.97	52.00
1	1	2	617.89	51.95
3	-2	-2	161.93	13.32
-3	2	2	146.56	13.14
2	1	2	172.89	13.22
1	-3	2	147.21	13.15
2	1	2	156.90	13.25
-2	0	-2	679.07	59.31
2	-2	-2	783.12	59.34
-2	0	-2	665.78	59.28
-2	1	-2	371.09	32.50
1	-2	-2	404.45	32.49
0	-2	-2	748.89	55.89
-2	2	-2	588.15	55.85
3	-1	-2	171.58	12.41
-1	-2	-2	143.04	12.29
-2	3	-2	138.58	12.19
-3	1	2	141.27	12.15
2	-3	2	147.68	12.18
1	2	2	130.12	12.14
-2	4	-2	53.75	4.55
4	-2	-2	53.95	4.77
2	2	2	47.28	4.56
-4	2	2	52.83	4.50
2	-4	2	58.47	4.53
2	-4	2	53.61	4.58
5	-3	-2	67.34	6.17
3	2	2	70.09	6.00
2	-5	2	72.37	5.92
2	-5	2	72.47	5.92
-5	3	2	67.65	5.89
4	-3	-2	74.98	6.48
3	1	2	77.28	6.42
1	-4	2	72.58	6.29
-4	3	2	77.92	6.28
3	1	2	75.82	6.47
1	-4	2	69.78	6.28
3	-3	-2	336.53	27.23
3	-3	-2	282.30	27.38
3	-3	-2	372.31	27.35
0	3	-2	304.59	27.24
3	0	2	330.79	27.32
3	0	2	326.14	27.31
1	2	-2	130.74	11.51
2	-3	-2	129.98	11.51
-3	1	-2	144.18	11.60
3	-1	2	140.20	11.55
3	-1	2	152.62	11.57
-2	3	2	126.43	11.49
1	-3	-2	150.02	12.31
2	1	-2	136.47	12.33
1	-3	-2	150.50	12.33
-3	2	-2	135.16	12.37
3	-2	2	157.90	12.34
-2	-1	2	149.97	12.30
3	0	-2	387.93	30.18
0	-3	-2	375.74	30.11
3	-3	2	313.01	30.02
-3	4	-2	66.22	5.78
-1	-3	-2	64.70	5.86
4	-1	-2	70.46	6.01
1	3	2	68.39	5.71
3	-4	2	69.10	5.74
-3	5	2	66.14	5.65
5	-2	-2	63.07	5.87
3	-5	2	70.80	5.61
-5	2	2	61.69	5.60
3	-5	2	65.12	5.54
3	-5	2	73.71	5.61
2	3	2	61.44	5.59
6	-3	-2	216.49	19.23
-3	6	-2	217.38	18.99
3	-6	2	248.41	18.96
-6	3	2	219.90	18.90
3	-6	2	222.73	18.90
3	3	2	208.29	18.98
3	-6	2	247.55	18.95
7	-4	-2	47.70	4.69
-7	4	2	50.19	4.18
3	-7	2	48.90	4.19
4	3	2	47.04	4.36
3	-7	2	52.33	4.23
6	-4	-2	212.74	18.19
4	2	2	212.11	18.02
2	-6	2	214.42	17.90
-6	4	2	221.43	17.84
2	-6	2	204.91	17.86
5	-4	-2	260.25	21.06
4	1	2	237.51	21.01
4	1	2	241.59	20.96
-5	4	2	282.88	20.80
1	-5	2	221.08	20.80
1	-5	2	248.07	20.81
0	4	-2	318.59	28.58
4	-4	-2	354.52	28.84
4	-4	-2	386.18	28.74
4	-4	-2	337.83	28.56
4	0	2	327.05	28.67
0	-4	2	373.40	28.56
4	0	2	295.85	28.67

-3	4	6	23.79	1.68
3	-4	-6	20.77	1.69
3	-4	-6	22.36	1.55
-3	4	5	10.51	0.86
3	-4	-5	8.99	0.88
3	-4	-5	9.10	0.69
3	-4	-5	8.78	0.69
-3	4	4	135.12	8.96
-3	4	4	129.44	8.96
-3	4	4	141.75	9.03
3	-4	-4	135.01	9.01
3	-4	-4	137.05	8.96
3	-4	-4	126.36	8.96
3	-4	-4	135.40	8.95
-3	4	3	29.62	2.16
-3	4	3	33.21	2.13
3	-4	-3	33.83	2.14
3	-4	-3	27.37	2.20
3	-4	-3	32.29	2.13
-3	4	2	57.94	3.92
-3	4	2	59.21	3.93
3	-4	-2	59.99	3.88
3	-4	-2	54.20	3.88
-3	4	1	42.69	3.13
-3	4	1	46.22	3.15
-3	4	1	49.08	3.12
3	-4	-1	52.11	3.12
3	-4	-1	45.66	3.14
3	-4	-1	44.41	3.16
3	-4	-1	39.42	3.16
3	-4	-1	48.91	3.11
-3	4	0	261.71	18.24
-3	4	0	288.46	18.26
-3	4	0	272.50	18.27
3	-4	0	261.23	18.24
3	-4	0	272.40	18.25
3	-4	0	283.63	18.29
3	-4	0	265.01	18.26
3	-4	0	289.34	18.24
-3	4	-1	46.71	3.33
-3	4	-1	48.79	3.35
3	-4	1	53.45	3.35
3	-4	1	48.97	3.34
3	-4	1	48.82	3.32
3	-4	1	48.74	3.33
-3	4	-2	50.03	3.84
-3	4	-2	60.69	3.88
-3	4	-2	56.50	3.86
3	-4	2	59.23	3.86
3	-4	2	57.89	3.85
3	-4	2	54.25	3.93
3	-4	2	61.82	3.85
-3	4	-3	30.53	2.16
3	-4	3	33.65	2.15
3	-4	3	30.97	2.14
3	-4	3	29.79	2.25
3	-4	3	31.45	2.14
-3	4	-4	133.84	8.96
3	-4	4	115.72	9.00
3	-4	4	142.65	8.94
3	-4	4	142.99	8.95
-3	4	-5	8.56	0.76
-3	4	-5	10.98	1.28
3	-4	5	8.71	0.74
3	-4	5	10.02	0.71
-3	4	-6	22.03	1.65
-3	4	-6	23.26	2.11
3	-4	6	23.74	1.60
-3	4	-7	1.77	0.96
-3	4	-7	1.54	0.30
-3	4	-8	19.39	1.45
-3	4	-8	20.39	2.39
-3	4	-9	0.15	0.27
-3	4	-9	-0.38	1.40
-3	4	-10	3.46	1.87
2	-3	-10	4.29	0.42
2	-3	-10	4.42	0.82
2	-3	-9	0.37	0.13
2	-3	-9	0.85	0.64
2	-3	-8	19.29	1.34
2	-3	-8	19.64	1.64
-2	3	7	0.63	0.52
2	-3	-7	1.73	0.22
2	-3	-7	1.77	0.43
-2	3	6	41.97	2.92
2	-3	-6	40.24	2.98
2	-3	-6	43.40	2.83
-2	3	5	11.32	0.92
2	-3	-5	10.49	0.97
2	-3	-5	11.51	0.79
-2	3	4	148.18	9.77
2	-3	-4	145.61	9.71
2	-3	-4	142.56	9.71
2	-3	-4	147.57	9.78
-2	3	3	39.32	2.28
-2	3	3	31.62	2.28
-2	3	3	36.94	2.29
-2	3	3	30.60	2.28
-2	3	3	28.93	2.26
2	-3	-3	35.25	2.24
2	-3	-3	32.60	2.38
2	-3	-3	30.11	2.23
-2	3	2	116.74	7.70
-2	3	2	113.15	7.69
-2	3	2	119.64	7.70
-2	3	2	111.12	7.69
2	-3	-2	117.51	7.67
2	-3	-2	123.66	7.68
2	-3	-2	104.68	7.73
-2	3	1	47.55	3.27
-2	3	1	48.32	3.27
-2	3	1	47.10	3.27
-2	3	1	47.76	3.25
-2	3	1	47.99	3.28
2	-3	-1	47.95	3.28
2	-3	-1	46.88	3.29
2	-3	-1	47.35	3.28

-4	4	2	338.81	28.57
3	-4	-2	71.94	5.89
3	-4	-2	60.60	5.71
1	3	-2	61.49	5.73
4	-1	2	68.61	5.79
4	-1	2	69.08	5.83
-1	-3	2	75.04	5.71
2	-4	-2	46.28	4.33
-4	2	-2	52.79	4.46
2	2	-2	49.07	4.37
-2	4	2	52.18	4.31
-2	-2	2	54.18	4.37
4	-2	2	51.53	4.39
4	-2	2	51.11	4.42
1	-4	-2	75.28	6.73
3	1	-2	73.49	6.79
1	-4	-2	80.80	6.65
4	-3	2	83.94	6.73
-3	-1	2	81.73	6.68
0	-4	-2	390.46	30.91
-4	4	-2	332.89	30.86
4	0	-2	394.11	31.07
0	4	2	336.95	30.77
4	-4	2	386.45	30.82
-1	-4	-2	256.60	19.62
-4	5	-2	212.64	19.72
4	-5	2	239.21	19.67
1	4	2	228.86	19.64
6	-2	-2	167.25	16.03
6	-2	-2	166.62	15.81
-4	6	-2	182.90	15.63
4	-6	2	217.75	15.58
2	4	2	189.24	15.57
7	-3	-2	39.52	4.38
7	-3	-2	42.17	4.00
4	-7	2	36.93	3.45
-7	3	2	43.58	3.51
3	4	2	42.86	3.61
8	-4	-2	0.07	0.67
8	-4	-2	-0.17	1.14
4	-8	2	0.58	0.24
4	-8	2	0.71	0.17
4	4	2	0.58	0.33
9	-5	-2	11.86	2.44
9	-5	-2	6.86	2.13
4	-9	2	13.83	1.19
5	4	2	12.61	1.55
4	-9	2	9.51	1.01
8	-5	-2	10.40	2.06
3	-8	2	10.62	1.03
3	-8	2	10.08	0.96
5	3	2	8.49	1.31
7	-5	-2	54.07	5.02
2	-7	2	52.43	4.45
5	2	2	50.67	4.68
6	-5	-2	39.59	3.78
5	1	2	34.90	3.52
1	-6	2	39.00	3.36
-6	5	2	43.69	3.43
5	1	2	37.63	3.64
5	-5	-2	96.17	7.46
5	-5	-2	98.77	7.74
0	5	-2	86.04	7.49
5	-5	-2	75.13	7.88
0	-5	2	99.29	7.46
5	0	2	80.46	7.63
0	-5	2	83.05	7.43
5	0	2	83.62	7.59
-5	5	2	91.23	7.48
4	-5	-2	208.42	17.66
4	-5	-2	204.90	17.43
1	4	-2	205.90	17.47
5	-1	2	210.78	17.60
-4	5	2	212.65	17.45
5	-1	2	206.04	17.56
2	3	-2	62.13	5.72
-2	-3	2	72.85	5.68
5	-2	2	66.66	5.77
5	-2	2	66.96	5.83
-3	5	2	66.85	5.68
2	-5	-2	77.18	5.86
-5	3	-2	67.94	6.02
2	-5	-2	62.77	5.90
3	2	-2	66.96	5.99
5	-3	2	70.29	6.00
5	-3	2	65.70	5.96
-3	-2	2	75.05	5.88
1	-5	-2	296.11	24.27
1	-5	-2	263.51	24.36
4	1	-2	270.53	24.52
1	-5	-2	269.45	24.26
5	-4	2	311.34	24.37
-4	-1	2	329.49	24.33
0	-5	-2	106.73	8.74
0	-5	-2	104.94	8.94
0	-5	-2	112.88	8.75
-5	5	-2	92.94	8.89
5	0	-2	100.33	9.15
0	5	2	93.64	8.78
5	-5	2	117.46	8.85
-1	-5	-2	43.32	3.32
-1	-5	-2	36.22	3.52
6	-1	-2	31.63	3.68
5	-6	2	43.75	3.41
1	5	2	37.35	3.36
-2	-5	-2	61.60	4.83
2	5	2	50.32	4.87
5	-7	2	58.22	4.89
8	-3	-2	6.17	1.63
8	-3	-2	6.99	1.42
3	5	2	10.29	1.03
5	-8	2	7.87	0.72
9	-4	-2	5.76	1.80
9	-4	-2	9.26	1.98
4	5	2	10.95	1.27
5	-9	2	8.81	0.81
10	-5	-2	16.68	3.07

2	-3	-1	50.56	3.25
2	-3	-1	51.72	3.25
2	-3	-1	47.79	3.29
-2	3	0	292.84	17.95
-2	3	0	266.13	17.93
2	-3	0	288.86	17.93
2	-3	0	265.79	17.94
2	-3	0	271.70	17.97
2	-3	0	254.66	17.92
2	-3	0	248.81	17.94
-2	3	-1	44.96	3.23
-2	3	-1	47.21	3.23
-2	3	-1	49.53	3.25
2	-3	1	49.33	3.24
2	-3	1	46.51	3.23
2	-3	1	49.55	3.22
2	-3	1	47.77	3.23
-2	3	-2	127.43	8.10
2	-3	2	121.10	8.09
2	-3	2	116.07	8.14
2	-3	2	120.41	8.10
2	-3	2	122.45	8.10
-2	3	-3	36.82	2.57
-2	3	-3	31.20	2.66
2	-3	3	39.48	2.56
2	-3	3	40.02	2.56
2	-3	3	40.53	2.58
-2	3	-4	131.06	9.48
-2	3	-4	140.49	9.48
-2	3	-4	135.22	9.56
2	-3	4	153.09	9.47
2	-3	4	149.87	9.47
-2	3	-5	11.15	1.16
-2	3	-5	11.16	0.83
2	-3	5	11.31	0.81
2	-3	5	10.96	0.83
-2	3	-6	42.89	3.18
-2	3	-6	40.82	2.93
2	-3	6	45.30	2.91
-2	3	-7	1.99	0.28
-2	3	-7	1.97	0.79
-2	3	-8	21.54	2.22
-2	3	-8	18.69	1.38
-2	3	-9	0.38	0.22
-2	3	-9	0.90	1.24
-2	3	-10	4.24	0.58
-2	3	-10	5.04	1.71
1	-2	-10	8.63	0.64
1	-2	-10	8.62	1.15
1	-2	-9	0.11	0.10
1	-2	-9	0.08	0.48
1	-2	-8	12.77	0.89
1	-2	-8	12.50	1.22
1	-2	-7	-0.10	0.32
1	-2	-7	0.00	0.10
-1	2	6	115.56	7.58
-1	2	6	115.62	7.52
1	-2	-6	105.74	7.47
-1	2	5	-0.14	0.24
1	-2	-5	-0.03	0.22
1	-2	-5	0.27	0.10
1	-2	-5	-0.13	0.07
-1	2	4	30.98	2.03
-1	2	4	27.84	2.04
1	-2	-4	28.38	2.06
1	-2	-4	29.63	1.99
1	-2	-4	30.36	1.98
-1	2	3	0.04	0.11
-1	2	3	0.47	0.10
-1	2	2	387.34	28.21
-1	2	2	424.05	28.14
-1	2	2	435.62	28.17
1	-2	-2	457.96	28.18
1	-2	-2	393.83	28.16
1	-2	-2	450.43	28.16
1	-2	-2	413.35	28.16
-1	2	1	0.24	0.12
-1	2	1	0.04	0.08
1	-2	-1	0.08	0.14
1	-2	-1	0.55	0.11
1	-2	-1	0.05	0.05
1	-2	-1	0.08	0.06
1	-2	-1	0.22	0.09
-1	2	0	92.67	5.78
-1	2	0	87.41	5.78
-1	2	0	88.37	5.80
-1	2	0	84.42	5.77
1	-2	0	86.78	5.80
1	-2	0	78.27	5.78
1	-2	0	86.51	5.77
1	-2	0	84.80	5.77
1	-2	0	79.49	5.78
-1	2	-1	0.09	0.09
-1	2	-1	0.08	0.07
-1	2	-1	0.10	0.09
1	-2	1	0.21	0.06
1	-2	1	-0.02	0.06
1	-2	1	0.11	0.08
1	-2	1	-0.10	0.16
1	-2	1	0.01	0.07
-1	2	-2	420.17	28.86
-1	2	-2	470.48	28.85
-1	2	-2	404.95	28.85
1	-2	2	442.04	28.86
1	-2	2	429.80	28.83
-1	2	-3	-0.11	0.26
-1	2	-3	0.19	0.09
-1	2	-3	-0.05	0.09
1	-2	3	0.09	0.08
1	-2	3	0.09	0.07
-1	2	-4	28.90	2.20
-1	2	-4	29.96	2.09
1	-2	4	32.11	2.08
1	-2	4	31.87	2.09
-1	2	-5	-0.13	0.38
-1	2	-5	-0.04	0.21
-1	2	-5	0.09	0.12

10	-5	-2	19.46	3.60
5	5	2	21.65	2.29
11	-6	-2	-1.27	2.40
11	-6	-2	1.68	1.79
6	5	2	3.32	0.88
10	-6	-2	12.75	2.24
4	-10	2	15.45	1.38
6	4	2	17.75	2.07
9	-6	-2	25.85	3.64
6	3	2	26.31	2.82
3	-9	2	27.23	2.40
3	-9	2	29.27	2.44
8	-6	-2	50.14	5.16
6	2	2	49.78	4.62
2	-8	2	52.17	4.35
7	-6	-2	14.11	2.20
6	1	2	11.97	1.52
1	-7	2	14.52	1.25
0	6	-2	1.92	0.37
6	-6	-2	0.34	0.94
6	-6	-2	3.10	0.70
6	0	2	2.44	0.62
0	-6	2	2.51	0.36
6	0	2	2.08	0.46
1	5	-2	35.90	2.99
5	-6	-2	35.13	2.91
-5	6	2	33.78	3.01
6	-1	2	33.75	3.11
6	-1	2	30.08	3.17
4	-6	-2	155.96	15.20
2	4	-2	182.68	15.29
6	-2	2	190.11	15.38
-4	6	2	186.33	15.24
6	-2	2	193.14	15.46
-6	3	-2	258.80	19.53
3	3	-2	217.10	19.40
-3	-3	2	233.52	19.30
6	-3	2	216.79	19.48
-3	6	2	228.48	19.29
6	-3	2	227.30	19.43
2	-6	-2	201.19	19.93
2	-6	-2	258.26	19.93
-6	4	-2	248.11	19.84
2	-6	-2	227.45	19.88
4	2	-2	233.42	20.14
6	-4	2	277.11	20.09
6	-4	2	217.74	20.01
1	-6	-2	44.83	3.88
1	-6	-2	42.80	4.05
1	-6	-2	52.07	3.91
5	1	-2	42.21	4.38
0	-6	-2	3.83	0.76
0	-6	-2	3.68	0.44
6	0	-2	3.08	0.90
0	-6	-2	3.65	0.42
6	-6	2	4.56	0.73
-1	-6	-2	13.84	1.71
-1	-6	-2	17.01	1.38
-1	-6	-2	15.35	1.36
1	6	2	14.54	1.49
-2	-6	-2	43.41	4.12
8	-2	-2	39.78	4.33
2	6	2	52.31	4.03
6	-9	2	37.12	3.09
3	6	2	31.18	3.13
10	-4	-2	12.83	2.69
4	6	2	17.97	1.95
11	-5	-2	2.52	1.57
11	-5	-2	0.23	2.41
5	6	2	3.34	0.87
11	-7	-2	-1.39	1.70
11	-7	-2	2.15	2.75
7	4	2	1.25	0.83
10	-7	-2	2.62	2.13
10	-7	-2	1.93	1.46
7	3	2	2.43	0.80
9	-7	-2	18.88	3.16
9	-7	-2	15.09	2.78
2	-9	2	20.45	1.82
7	2	2	15.80	2.14
2	-9	2	19.51	1.69
7	1	2	46.25	4.46
1	-8	2	51.56	4.21
0	7	-2	24.23	2.17
7	0	2	20.29	2.23
0	-7	2	23.95	2.00
7	0	2	22.06	2.44
1	6	-2	14.28	1.36
6	-7	-2	13.34	1.20
-6	7	2	13.38	1.27
7	-1	2	11.28	1.43
7	-1	2	11.80	1.68
5	-7	-2	40.65	3.82
2	5	-2	46.56	3.97
-5	7	2	48.19	3.88
7	-2	2	42.63	4.18
7	-2	2	46.71	4.09
3	4	-2	45.69	3.97
-4	7	2	45.78	3.82
7	-3	2	44.81	4.04
7	-3	2	40.50	4.15
4	3	-2	47.81	4.36
7	-4	2	47.69	4.29
7	-4	2	46.89	4.41
5	2	-2	58.01	5.82
2	-7	-2	70.57	5.40
2	-7	-2	61.24	5.34
2	-7	-2	55.20	5.44
7	-5	2	70.14	5.59
6	1	-2	15.30	2.43
1	-7	-2	17.81	1.61
1	-7	-2	19.96	1.65
1	-7	-2	18.12	1.88
0	-7	-2	32.85	2.72
0	-7	-2	28.62	2.97
0	-7	-2	31.67	2.69
-1	-7	-2	63.00	5.10

1	-2	5	0.10	0.11
1	-2	5	0.37	0.12
-1	2	-6	103.77	7.37
-1	2	-6	112.15	7.54
1	-2	6	115.74	7.38
-1	2	-7	0.02	0.14
-1	2	-7	0.29	0.59
1	-2	7	0.08	0.21
-1	2	-8	15.06	1.65
-1	2	-8	12.45	0.93
-1	2	-9	-0.04	1.02
-1	2	-9	-0.07	0.14
-1	2	-10	9.48	0.72
-1	2	-10	11.56	1.69
0	-1	-10	4.61	1.23
0	-1	-10	4.66	0.43
0	-1	-9	0.55	0.74
0	-1	-9	0.34	0.17
0	-1	-8	26.12	2.23
0	-1	-8	28.96	1.93
0	-1	-7	1.93	0.51
0	-1	-7	1.08	0.29
0	1	6	45.45	3.04
0	-1	-6	44.01	3.15
0	1	5	12.60	0.95
0	-1	-5	12.61	1.03
0	-1	-4	231.64	15.05
0	-1	-4	219.51	14.97
0	1	3	25.84	1.79
0	1	2	119.08	8.03
0	1	2	116.42	8.03
0	-1	-2	127.06	8.04
0	1	1	41.64	2.75
0	1	1	42.33	2.76
0	1	1	41.13	2.75
0	1	1	41.49	2.75
0	-1	-1	39.90	2.75
0	-1	-1	40.05	2.75
0	-1	-1	36.65	2.75
0	1	0	331.05	24.25
0	1	0	403.38	24.30
0	-1	0	361.24	24.29
0	1	-1	41.12	2.84
0	1	-1	44.03	2.88
0	1	-1	41.06	2.85
0	-1	1	41.63	2.84
0	-1	1	41.16	2.85
0	1	-2	126.59	8.40
0	1	-2	133.34	8.42
0	1	-2	118.49	8.37
0	-1	2	125.73	8.39
0	1	-3	50.60	3.47
0	1	-3	51.33	3.48
0	-1	3	50.64	3.41
0	-1	3	51.71	3.44
0	1	-4	229.34	14.68
0	1	-4	192.05	14.68
0	-1	4	219.92	14.59
0	-1	4	237.82	14.63
0	1	-5	12.89	1.08
0	-1	5	12.66	0.92
0	1	-6	43.43	3.09
0	-1	6	43.36	2.94
0	1	-7	1.78	0.58
0	-1	7	1.60	0.23
0	1	-8	32.98	2.63
0	1	-9	0.36	0.92
0	1	-10	4.15	1.36
-1	0	-10	3.95	0.57
-1	0	-10	5.87	1.33
-1	0	-10	4.90	0.61
-1	0	-9	1.08	0.83
-1	0	-8	25.95	2.06
-1	0	-8	24.04	1.76
-1	0	-7	1.70	0.51
1	0	6	45.92	3.02
-1	0	-6	44.22	3.18
1	0	5	12.85	0.91
1	0	5	13.70	0.91
-1	0	-5	13.41	1.07
1	0	4	244.47	16.38
1	0	4	271.23	16.40
-1	0	-4	224.37	16.45
1	0	3	49.41	3.48
-1	0	-3	53.95	3.51
1	0	2	89.78	6.00
1	0	1	39.26	2.77
1	0	1	43.72	2.76
1	0	1	41.13	2.77
-1	0	-1	39.11	2.77
-1	0	-1	39.49	2.77
1	0	0	295.06	17.93
1	0	0	281.01	17.93
1	0	0	247.25	17.90
1	0	0	265.77	17.90
-1	0	0	257.87	17.91
1	0	-1	40.02	2.65
1	0	-1	42.11	2.64
1	0	-1	39.88	2.66
1	0	-1	42.84	2.66
1	0	-1	32.36	2.66
-1	0	1	37.82	2.63
-1	0	1	40.73	2.64
-1	0	1	33.45	2.65
1	0	-2	122.68	7.63
1	0	-2	123.21	7.65
1	0	-2	117.89	7.62
-1	0	2	94.20	7.67
1	0	-3	49.61	3.13
-1	0	3	43.73	3.21
-1	0	4	218.38	14.58
1	0	-5	9.70	0.88
-1	0	5	11.72	0.87
1	0	-7	1.67	0.48
-1	0	7	1.61	0.28
1	0	-8	27.60	2.18
1	0	-9	0.70	0.77

-1	-7	-2	55.18	5.32
1	7	2	61.62	5.26
-2	-7	-2	20.17	2.18
2	7	2	21.22	2.12
10	-3	-2	2.31	0.99
3	7	2	4.07	0.74
11	-4	-2	0.85	1.28
-4	-7	-2	3.62	0.92
7	-11	2	3.39	0.58
4	7	2	3.28	0.84
10	-8	-2	-1.05	1.98
10	-8	-2	-0.60	1.25
8	2	2	-0.61	0.63
2	-10	2	0.05	0.72
2	-10	2	0.22	0.22
1	-9	2	7.21	0.74
8	1	2	4.41	1.12
8	-8	-2	118.20	10.08
8	-8	-2	97.87	9.59
7	-8	-2	158.86	13.10
7	-8	-2	167.43	13.35
-7	8	2	129.28	12.75
6	-8	-2	42.97	4.17
2	6	-2	46.68	4.60
-6	8	2	54.97	4.34
8	-2	2	52.26	4.50
3	5	-2	10.50	1.25
5	-8	-2	7.38	0.84
-5	8	2	9.61	0.94
8	-3	2	8.73	1.78
8	-3	2	8.19	1.35
4	4	-2	0.37	0.45
4	-8	-2	0.30	0.19
4	-8	-2	1.26	0.44
8	-4	2	-0.58	0.75
8	-4	2	0.84	0.50
3	-8	-2	8.78	0.96
5	3	-2	9.69	1.92
8	-5	2	9.02	1.60
2	-8	-2	76.35	5.57
2	-8	-2	58.99	5.60
6	2	-2	57.17	6.19
1	-8	-2	79.51	6.50
1	-8	-2	72.72	6.89
0	-8	-2	43.87	4.23
0	-8	-2	48.51	3.98
-1	-8	-2	9.93	1.49
-1	-8	-2	9.23	0.94
-2	-8	-2	0.97	0.73
-2	-8	-2	0.10	0.21
10	-2	-2	-1.08	0.73
2	8	2	1.26	0.55
8	-10	2	0.35	0.47
10	-9	-2	1.70	1.55
10	-9	-2	-2.20	1.95
1	-10	2	0.73	0.54
1	-10	2	1.23	0.28
9	1	2	0.79	0.68
9	-9	-2	5.01	1.29
0	-9	2	2.75	0.53
8	-9	-2	14.30	3.19
1	8	-2	9.27	1.39
9	-1	2	5.07	1.19
-1	-8	2	7.19	0.80
2	7	-2	19.83	1.96
7	-9	-2	17.64	1.55
9	-2	2	15.42	2.00
9	-2	2	13.43	2.38
3	6	-2	29.25	2.59
6	-9	-2	21.49	2.15
9	-3	2	23.75	2.97
9	-3	2	25.90	2.68
4	5	-2	13.25	2.13
5	-9	-2	9.01	1.09
9	-4	2	10.73	1.70
4	-9	-2	9.15	0.99
9	-5	2	10.94	1.81
9	-5	2	12.56	2.29
3	-9	-2	44.23	3.62
6	3	-2	31.60	4.32
9	-6	2	42.73	4.00
2	-9	-2	22.13	2.33
2	-9	-2	29.04	2.28
7	2	-2	19.07	3.29
1	-9	-2	8.08	1.43
1	-9	-2	11.57	1.11
9	-9	2	8.59	1.43
9	-9	2	6.39	1.08
-1	-9	-2	0.54	0.69
10	-1	-2	0.52	0.89
9	-10	2	1.98	0.77
9	-10	-2	1.08	1.67
1	9	-2	1.48	0.73
10	-1	2	0.11	0.62
-1	-9	2	1.05	0.25
8	-10	-2	0.41	1.30
2	8	-2	-0.41	0.64
8	-10	-2	1.60	0.95
10	-2	2	-0.88	0.95
-2	-8	2	-0.07	0.21
10	-2	2	-0.26	0.58
7	-10	-2	3.20	0.55
3	7	-2	3.46	0.83
10	-3	2	1.83	1.14
10	-3	2	2.73	0.85
6	-10	-2	12.59	1.39
10	-4	2	15.57	2.32
10	-4	2	15.05	2.75
5	-10	-2	18.84	1.91
10	-5	2	24.65	2.87
10	-5	2	28.53	3.42
6	4	-2	20.47	2.97
4	-10	-2	13.48	1.39
3	-10	-2	7.49	1.14
10	-7	2	8.37	1.53
10	-7	2	8.27	1.58
2	-10	-2	-0.32	0.49

1	0	-10	4.49	1.22
-2	1	-10	7.94	0.99
-2	1	-10	6.30	0.78
-2	1	-10	9.75	1.45
-2	1	-9	0.47	0.85
-2	1	-9	-0.11	0.46
-2	1	-8	8.84	1.11
-2	1	-8	11.45	1.31
-2	1	-7	-0.42	0.44
-2	1	-7	-0.35	0.42
-2	1	-6	34.60	2.50
2	-1	5	0.12	0.21
-2	1	-5	0.10	0.29
2	-1	4	29.35	1.76
2	-1	4	23.72	1.84
-2	1	-4	23.58	1.79
-2	1	-4	28.09	1.86
2	-1	3	0.09	0.15
2	-1	3	-0.01	0.04
2	-1	3	0.07	0.05
-2	1	-3	-0.04	0.06
-2	1	-3	-0.25	0.24
-2	1	-3	0.12	0.16
2	-1	2	437.63	30.70
2	-1	2	464.90	30.69
-2	1	-2	528.45	30.70
-2	1	-2	414.61	30.68
2	-1	1	0.19	0.12
2	-1	1	0.80	0.21
2	-1	1	0.01	0.13
2	-1	1	-0.10	0.16
2	-1	1	0.11	0.10
-2	1	-1	0.19	0.20
-2	1	-1	0.05	0.12
-2	1	-1	-0.07	0.10
-2	1	-1	0.08	0.10
2	-1	0	76.18	5.63
2	-1	0	81.98	5.67
2	-1	0	90.13	5.63
2	-1	0	81.57	5.64
2	-1	0	82.13	5.62
-2	1	0	83.23	5.62
-2	1	0	83.04	5.65
2	-1	-1	0.00	0.20
2	-1	-1	0.02	0.23
2	-1	-1	-0.11	0.10
2	-1	-1	-0.11	0.17
2	-1	-1	0.13	0.12
-2	1	1	-0.15	0.22
-2	1	1	0.06	0.15
-2	1	1	0.03	0.09
-2	1	1	-0.08	0.15
2	-1	-2	443.72	32.43
2	-1	-2	482.14	32.42
2	-1	-2	516.45	32.41
2	-1	-2	507.35	32.45
2	-1	-3	0.46	0.16
2	-1	-3	0.12	0.12
2	-1	-3	-0.10	0.18
2	-1	-3	0.39	0.14
2	-1	-3	0.05	0.04
-2	1	3	-0.01	0.26
2	-1	-4	28.28	1.75
2	-1	-4	22.98	1.72
2	-1	-4	27.62	1.78
2	-1	-4	21.78	1.74
2	-1	-5	0.25	0.22
2	-1	-5	0.03	0.28
-2	1	5	0.58	0.39
-2	1	5	0.14	0.34
2	-1	-7	-0.33	0.35
-2	1	7	0.62	0.48
-2	1	7	-0.10	0.29
2	-1	-8	13.11	1.23
2	-1	-9	0.53	0.52
2	-1	-10	9.51	1.31
-3	2	-10	3.97	0.62
-3	2	-10	6.57	1.45
-3	2	-9	0.95	0.95
-3	2	-9	0.37	0.42
-3	2	-8	20.96	1.90
-3	2	-8	17.97	1.58
-3	2	-7	2.08	0.49
-3	2	-7	1.47	0.58
-3	2	-6	40.23	2.85
-3	2	-6	38.83	2.85
3	-2	5	11.23	0.74
3	-2	5	10.39	0.88
-3	2	-5	10.21	1.02
-3	2	-5	11.06	0.97
-3	2	-5	10.00	0.80
3	-2	4	147.13	9.73
3	-2	4	136.81	9.79
3	-2	4	152.96	9.73
-3	2	-4	151.82	9.85
-3	2	-4	140.48	9.74
-3	2	-4	148.91	9.80
3	-2	3	39.41	2.52
3	-2	3	33.60	2.55
3	-2	3	39.52	2.57
3	-2	3	37.10	2.53
-3	2	-3	37.27	2.53
-3	2	-3	35.92	2.55
-3	2	-3	39.47	2.64
3	-2	2	120.89	7.98
3	-2	2	122.06	7.95
3	-2	2	109.57	7.94
3	-2	2	128.73	7.96
-3	2	-2	116.73	7.92
-3	2	-2	125.97	7.99
-3	2	-2	119.04	7.94
-3	2	-2	109.24	7.93
3	-2	1	49.58	3.15
3	-2	1	41.78	3.12
3	-2	1	47.43	3.13
3	-2	1	41.78	3.12
-3	2	-1	45.69	3.14

8	2	-2	-0.82	1.16
10	-8	2	1.21	0.88
10	-8	2	-0.28	0.92
9	1	-2	0.10	1.14
1	-10	-2	1.58	0.73
10	-9	2	2.08	0.97
10	-9	2	3.28	0.93
4	7	-2	3.36	1.24
11	-4	2	1.69	1.12
11	-4	2	1.05	1.58
5	6	-2	4.95	1.60
6	-11	-2	4.03	0.66
11	-5	2	2.45	1.31
11	-5	2	2.69	1.67
6	5	-2	5.51	1.76
5	-11	-2	3.56	0.67
11	-6	2	7.61	1.89
11	-6	2	2.72	1.38
7	4	-2	2.05	1.74
4	-11	-2	1.80	0.43
11	-7	2	3.85	1.67
11	-7	2	3.27	1.36
0	0	-3	0.06	0.05
0	0	3	-0.01	0.05
0	0	3	-0.01	0.04
0	0	3	0.01	0.08
1	-1	-3	69.03	5.98
1	-1	-3	62.36	5.94
0	1	-3	65.98	5.96
-1	0	-3	76.47	5.99
-1	0	-3	69.95	5.97
-1	1	3	76.24	5.98
0	-1	3	69.66	5.96
1	0	3	76.53	6.02
1	0	-3	60.38	5.48
0	-1	-3	67.62	5.52
-1	1	-3	70.89	5.48
-1	1	-3	62.64	5.53
-1	0	3	64.10	5.48
1	-1	3	64.64	5.48
1	-1	3	67.81	5.48
0	1	3	59.88	5.47
1	-1	3	67.14	5.50
2	-1	-3	0.34	0.17
-1	-1	-3	0.13	0.08
-1	-1	-3	0.35	0.28
2	-1	-3	0.00	0.14
1	1	3	0.08	0.14
1	1	3	0.17	0.09
-2	-1	-3	49.92	4.50
3	-2	-3	47.07	4.36
3	-2	-3	55.78	4.62
2	1	3	55.23	4.39
2	1	3	47.85	4.38
1	-3	3	51.31	4.35
1	-3	3	49.41	4.34
-3	2	3	53.99	4.34
2	-2	-3	321.96	26.77
-2	2	3	364.24	26.77
2	0	3	294.39	26.77
2	0	3	299.17	26.76
-2	1	-3	0.06	0.12
-2	1	-3	0.40	0.14
1	-2	-3	0.14	0.13
-1	-1	3	0.09	0.10
-1	2	3	0.41	0.11
2	-1	3	0.27	0.10
-1	2	3	0.28	0.15
2	0	-3	326.92	27.78
-2	2	-3	315.58	27.79
0	-2	-3	354.51	27.82
-2	0	3	363.15	27.77
-2	0	3	336.30	27.76
2	-2	3	332.13	27.73
2	-2	3	294.41	27.76
3	-1	-3	50.49	4.37
-1	-2	-3	54.72	4.35
-2	3	-3	47.40	4.22
2	-3	3	48.69	4.18
2	-3	3	50.53	4.20
1	2	3	47.02	4.14
2	-3	3	49.05	4.15
-3	1	3	47.68	4.17
4	-2	-3	0.10	0.37
-2	4	-3	0.68	0.19
2	-4	3	0.22	0.14
-4	2	3	0.03	0.28
2	2	3	0.46	0.14
5	-3	-3	3.13	0.79
2	-5	3	4.60	0.53
-5	3	3	5.63	0.81
-5	3	3	4.89	0.48
2	-5	3	4.90	0.53
3	2	3	4.02	0.63
4	-3	-3	39.14	3.73
-3	-1	-3	40.03	3.51
3	1	3	39.44	3.47
1	-4	3	40.03	3.38
3	1	3	33.27	3.52
1	-4	3	40.23	3.39
-4	3	3	45.25	3.39
-3	0	-3	1.45	0.33
3	-3	-3	1.78	0.41
3	-3	-3	1.91	0.50
3	-3	-3	2.08	0.32
-3	0	-3	2.33	0.56
0	3	-3	1.98	0.32
3	0	3	2.87	0.45
0	-3	3	1.81	0.27
1	2	-3	44.01	3.79
2	-3	-3	44.58	3.80
-3	1	-3	42.32	3.89
-2	3	3	42.70	3.74
-1	-2	3	49.14	3.79
3	-1	3	42.41	3.81
3	-1	3	45.96	3.80
-3	2	-3	41.95	4.03

-3	2	-1	47.57	3.14
-3	2	-1	47.22	3.13
-3	2	-1	46.16	3.12
3	-2	0	267.49	17.75
3	-2	0	255.86	17.78
3	-2	0	275.81	17.82
3	-2	0	254.37	17.79
3	-2	0	271.31	17.76
-3	2	0	277.78	17.79
-3	2	0	265.03	17.76
3	-2	-1	46.96	2.96
3	-2	-1	41.57	2.95
3	-2	-1	45.53	3.03
3	-2	-1	37.18	3.06
3	-2	-1	44.34	2.94
3	-2	-1	45.75	2.92
-3	2	1	38.41	2.97
-3	2	1	46.21	2.95
3	-2	-2	86.64	6.77
3	-2	-2	111.27	6.68
3	-2	-2	89.05	6.78
3	-2	-2	106.60	6.68
-3	2	2	95.52	6.68
-3	2	2	110.80	6.80
3	-2	-3	35.79	2.46
3	-2	-3	39.79	2.53
3	-2	-3	35.02	2.46
-3	2	3	32.87	2.59
3	-2	-4	142.07	9.60
3	-2	-4	148.71	9.62
3	-2	-4	144.38	9.57
3	-2	-4	139.88	9.63
3	-2	-5	10.51	0.89
3	-2	-5	10.84	0.81
3	-2	-5	9.87	0.81
3	-2	-5	10.69	0.73
-3	2	5	7.71	1.14
3	-2	-6	40.71	2.57
3	-2	-6	41.36	2.72
-3	2	6	34.82	2.81
-3	2	6	34.52	2.88
3	-2	-7	1.91	0.23
3	-2	-7	1.69	0.42
-3	2	7	1.55	0.48
-3	2	7	0.82	0.64
3	-2	-8	18.03	1.54
3	-2	-8	17.81	1.24
3	-2	-9	0.38	0.17
3	-2	-9	0.24	0.67
3	-2	-10	4.76	0.56
3	-2	-10	4.28	1.05
-4	3	-9	0.00	0.40
-4	3	-9	0.92	1.06
-4	3	-8	15.32	1.37
-4	3	-7	2.13	0.67
-4	3	-7	1.05	0.42
4	-3	6	20.89	1.60
4	-3	6	21.94	1.56
-4	3	-6	22.70	1.81
-4	3	-6	21.00	1.69
4	-3	5	9.61	0.67
4	-3	5	8.82	0.86
4	-3	5	8.37	0.80
-4	3	-5	9.28	0.99
-4	3	-5	9.11	0.93
4	-3	4	143.68	9.16
4	-3	4	123.87	9.18
4	-3	4	133.80	9.10
-4	3	-4	135.59	9.20
-4	3	-4	145.87	9.18
4	-3	3	32.81	2.12
4	-3	3	29.89	2.09
-4	3	-3	27.98	2.06
-4	3	-3	29.92	2.12
-4	3	-3	31.34	2.17
4	-3	2	55.02	3.63
4	-3	2	52.03	3.62
4	-3	2	45.36	3.71
-4	3	-2	55.08	3.61
-4	3	-2	58.56	3.67
-4	3	-2	54.21	3.64
4	-3	1	43.54	3.22
4	-3	1	43.14	3.14
4	-3	1	50.20	3.14
-4	3	-1	46.25	3.13
-4	3	-1	47.52	3.15
-4	3	-1	43.02	3.14
-4	3	-1	47.58	3.17
4	-3	0	259.35	17.72
4	-3	0	275.52	17.67
4	-3	0	265.51	17.67
4	-3	0	251.78	17.74
-4	3	0	296.70	17.73
-4	3	0	242.35	17.67
-4	3	0	265.92	17.69
4	-3	-1	45.17	3.14
4	-3	-1	41.30	3.27
4	-3	-1	46.36	3.06
4	-3	-1	49.48	3.11
4	-3	-1	43.50	3.10
-4	3	1	46.31	3.14
-4	3	1	44.24	3.10
-4	3	1	51.07	3.19
-4	3	1	42.85	3.19
4	-3	-2	53.56	3.45
4	-3	-2	51.30	3.43
-4	3	2	43.15	3.47
-4	3	2	55.57	3.57
4	-3	-3	30.19	1.92
4	-3	-3	24.28	2.07
4	-3	-3	30.16	1.91
-4	3	3	22.90	1.91
-4	3	3	30.68	2.11
4	-3	-4	130.18	8.82
4	-3	-4	125.35	8.82
4	-3	-4	136.18	8.79
4	-3	-4	134.08	8.90

2	1	-3	45.66	3.98
1	-3	-3	44.87	3.98
1	-3	-3	50.30	3.90
-2	-1	3	49.14	3.95
3	-2	3	46.44	3.98
3	0	-3	2.66	0.55
0	-3	-3	2.12	0.47
0	-3	-3	2.38	0.23
3	-3	3	1.61	0.36
-3	0	3	2.55	0.35
-3	0	3	2.32	0.39
-1	-3	-3	37.83	3.51
4	-1	-3	39.09	3.65
-3	4	-3	37.10	3.38
-1	-3	-3	40.09	3.33
3	-4	3	39.87	3.33
-4	1	3	36.41	3.29
1	3	3	40.48	3.28
-2	-3	-3	3.17	0.75
5	-2	-3	2.78	0.73
-3	5	-3	4.33	0.67
3	-5	3	4.83	0.55
3	-5	3	4.21	0.41
-5	2	3	4.37	0.45
3	-5	3	4.78	0.55
2	3	3	4.50	0.50
6	-3	-3	-0.09	0.76
6	-3	-3	0.49	0.56
-6	3	3	3.27	0.71
7	-4	-3	3.23	1.01
7	-4	-3	1.36	1.12
4	3	3	2.93	0.67
3	-7	3	4.57	0.54
6	-4	-3	62.01	5.91
4	2	3	61.14	5.52
-6	4	3	67.73	5.40
2	-6	3	63.96	5.41
5	-4	-3	0.73	0.54
-4	-1	-3	1.91	0.41
5	-4	-3	1.45	0.72
-5	4	3	1.61	0.28
4	1	3	1.20	0.34
1	-5	3	1.05	0.22
-5	4	3	1.65	0.33
1	-5	3	1.08	0.20
4	1	3	0.70	0.38
-4	0	-3	164.40	12.72
4	-4	-3	170.50	12.82
0	4	-3	133.63	12.56
4	-4	-3	146.15	12.55
-4	4	3	164.65	12.56
0	-4	3	158.68	12.55
4	0	3	135.01	12.63
4	0	3	140.97	12.62
0	-4	3	132.90	12.53
1	3	-3	35.29	3.21
3	-4	-3	42.90	3.41
-4	1	-3	36.47	3.36
3	-4	-3	35.12	3.20
4	-1	3	38.43	3.29
-3	4	3	34.25	3.17
-1	-3	3	40.74	3.19
4	-1	3	36.70	3.25
-4	2	-3	0.05	0.33
-4	2	-3	0.31	0.27
2	2	-3	0.15	0.16
2	-4	-3	-0.04	0.14
4	-2	3	-0.03	0.14
-2	4	3	0.03	0.06
4	-2	3	-0.18	0.15
-2	4	3	0.29	0.26
-2	-2	3	0.08	0.09
1	-4	-3	37.59	3.57
-4	3	-3	36.09	3.63
1	-4	-3	46.40	3.46
3	1	-3	38.03	3.61
-3	-1	3	43.60	3.52
4	-3	3	43.45	3.56
0	-4	-3	159.69	13.38
-4	4	-3	139.40	13.34
4	0	-3	165.61	13.52
0	-4	-3	172.03	13.20
4	-4	3	151.84	13.27
-4	5	-3	0.93	0.37
-1	-4	-3	1.43	0.25
-1	-4	-3	1.29	0.64
5	-1	-3	1.15	0.64
1	4	3	1.61	0.33
4	-5	3	1.18	0.26
-5	1	3	2.11	0.42
-5	1	3	2.10	0.45
6	-2	-3	53.89	5.59
4	-6	3	66.95	5.25
2	4	3	61.92	5.22
-3	-4	-3	4.05	0.79
7	-3	-3	2.01	0.74
4	-7	3	3.36	0.34
4	-7	3	4.48	0.59
3	4	3	3.64	0.60
-4	-4	-3	0.23	0.31
8	-4	-3	-0.26	0.80
4	4	3	-0.04	0.27
9	-5	-3	-1.80	1.58
9	-5	-3	-0.66	1.02
4	-9	3	0.04	0.16
5	4	3	-0.07	0.38
4	-9	3	0.08	0.24
8	-5	-3	6.27	1.44
3	-8	3	8.08	0.73
5	3	3	5.75	1.02
7	-5	-3	-0.12	1.07
-5	-2	-3	2.35	0.61
7	-5	-3	-0.10	0.79
5	2	3	0.55	0.35
6	-5	-3	18.68	2.50
5	1	3	20.73	1.99
1	-6	3	19.96	1.80
-4	3	4	132.49	8.96
4	-3	-5	10.10	0.82
4	-3	-5	8.15	0.81
4	-3	-5	8.56	0.75
4	-3	-5	8.67	0.68
-4	3	5	8.93	1.12
4	-3	-6	21.53	1.48
4	-3	-6	22.17	1.57
4	-3	-6	21.87	1.61
-4	3	6	17.14	1.79
-4	3	6	22.89	1.91
4	-3	-7	1.99	0.28
4	-3	-7	1.36	0.39
-4	3	7	1.39	0.53
-4	3	7	1.12	0.61
4	-3	-8	17.50	1.57
4	-3	-8	17.36	1.28
-4	3	8	22.11	2.13
4	-3	-9	0.49	0.70
4	-3	-9	0.42	0.20
4	-3	-10	2.82	0.48
4	-3	-10	2.64	1.00
-5	4	-9	-0.25	0.44
-5	4	-8	5.22	0.69
5	-4	7	-0.07	0.48
-5	4	-7	0.03	0.39
5	-4	6	43.39	2.98
-5	4	-6	40.83	3.03
5	-4	5	0.93	0.58
5	-4	5	0.44	0.28
-5	4	-5	0.48	0.35
5	-4	4	16.39	1.28
-5	4	-4	17.39	1.38
5	-4	3	0.35	0.55
5	-4	3	0.66	0.24
5	-4	3	1.03	0.23
-5	4	-3	0.84	0.23
-5	4	-3	1.27	0.36
5	-4	2	183.99	13.06
5	-4	2	199.38	13.07
-5	4	-2	202.37	13.10
-5	4	-2	196.55	13.09
5	-4	1	1.36	0.29
5	-4	1	0.93	0.49
5	-4	1	1.06	0.23
-5	4	-1	1.47	0.30
-5	4	-1	1.07	0.31
-5	4	-1	1.36	0.28
5	-4	0	24.01	1.80
5	-4	0	27.77	1.98
5	-4	0	25.81	1.93
5	-4	0	24.66	1.79
-5	4	0	25.99	1.88
-5	4	0	24.61	1.80
-5	4	0	25.50	1.86
5	-4	-1	1.45	0.55
5	-4	-1	1.74	0.26
5	-4	-1	1.42	0.20
5	-4	-1	1.24	0.25
-5	4	1	1.53	0.39
-5	4	1	1.27	0.23
-5	4	1	1.37	0.32
5	-4	-2	197.41	13.79
5	-4	-2	208.33	13.81
-5	4	2	224.70	13.90
-5	4	2	200.31	13.84
-5	4	2	203.72	13.92
5	-4	-3	0.23	0.49
5	-4	-3	0.67	0.21
5	-4	-3	0.71	0.17
5	-4	-3	0.77	0.21
-5	4	3	0.39	0.25
-5	4	3	0.44	0.35
-5	4	3	1.52	0.48
5	-4	-4	16.87	1.27
5	-4	-4	16.54	1.26
5	-4	-4	18.60	1.23
5	-4	-4	14.91	1.47
-5	4	4	17.53	1.52
5	-4	-5	0.41	0.24
5	-4	-5	0.51	0.29
5	-4	-5	0.46	0.34
5	-4	-5	0.55	0.18
-5	4	5	0.16	0.28
-5	4	5	0.60	0.50
5	-4	-6	44.95	3.15
5	-4	-6	42.13	3.20
5	-4	-6	45.08	3.09
-5	4	6	49.89	3.41
5	-4	-7	0.22	0.17
5	-4	-7	0.19	0.34
-5	4	7	0.00	0.57
-5	4	7	-0.14	0.50
5	-4	-8	5.39	0.80
5	-4	-8	6.34	0.57
-5	4	8	7.16	0.99
5	-4	-9	-0.10	0.72
5	-4	-9	0.41	0.24
-6	5	-8	7.03	1.03
6	-5	7	2.11	0.85
-6	5	-7	1.38	0.49
6	-5	6	11.13	1.08
-6	5	-6	12.55	1.20
6	-5	5	5.40	0.72
6	-5	5	8.96	1.11
-6	5	-5	6.44	0.88
6	-5	4	52.70	3.55
-6	5	-4	49.42	3.60
6	-5	3	12.01	1.55
6	-5	3	15.61	1.22
-6	5	-3	18.44	1.33
-6	5	-3	15.64	1.24
6	-5	2	33.24	2.21
-6	5	-2	31.03	2.24
-6	5	-2	29.02	2.26
6	-5	1	29.23	2.09
6	-5	1	30.24	2.40
-6	5	3	23.77	1.81
5	1	3	17.70	2.08
5	-5	-3	2.88	0.72
0	5	-3	2.41	0.50
5	-5	-3	0.85	0.70
5	-5	-3	2.42	0.37
-5	5	3	2.78	0.40
5	0	3	2.14	0.42
0	-5	3	2.32	0.34
5	0	3	1.81	0.48
-5	5	3	3.36	0.42
4	-5	-3	1.08	0.46
-5	1	-3	1.07	0.41
4	-5	-3	0.43	0.58
4	-5	-3	1.12	0.24
1	4	-3	0.92	0.24
-1	-4	3	0.86	0.18
5	-1	3	0.65	0.29
5	-1	3	0.83	0.33
-4	5	3	1.00	0.18
3	-5	-3	3.90	0.51
2	3	-3	4.30	0.59
-5	2	-3	3.10	0.61
5	-2	3	4.46	0.64
5	-2	3	3.20	0.58
-2	-3	3	4.91	0.49
-3	5	3	4.79	0.45
2	-5	-3	3.48	0.59
3	2	-3	4.72	0.73
-5	3	-3	6.01	0.92
2	-5	-3	5.02	0.47
-3	-2	3	4.56	0.54
5	-3	3	5.75	0.68
5	-3	3	4.62	0.72
1	-5	3	0.93	0.20
-5	4	-3	1.05	0.37
1	-5	-3	0.45	0.37
4	1	-3	1.16	0.45
-4	-1	3	1.14	0.24
5	-4	3	1.54	0.30
-4	-1	3	0.86	0.24
0	-5	-3	3.34	0.37
0	-5	-3	2.72	0.90
0	-5	3	3.81	0.39
5	-5	3	2.96	0.58
6	-1	-3	17.73	2.48
-1	-5	-3	20.01	2.26
-1	-5	-3	25.03	1.89
5	-6	3	21.74	1.99
1	5	3	21.15	1.95
7	-2	-3	0.86	0.66
-2	-5	-3	0.86	0.19
-2	-5	-3	1.27	0.69
2	5	3	0.73	0.26
5	-7	3	0.61	0.27
-3	-5	-3	6.99	1.30
5	-8	3	8.61	0.96
3	5	3	7.57	0.95
-4	-5	-3	0.76	0.52
9	-4	-3	-0.93	0.84
5	-9	3	0.08	0.23
4	5	3	-0.26	0.56
-5	-5	-3	0.65	0.68
5	-10	3	0.01	0.27
11	-6	-3	-2.18	1.82
6	5	3	-0.05	0.70
5	-11	3	0.05	0.31
10	-6	-3	-0.99	1.92
10	-6	-3	-0.14	1.35
6	4	3	2.00	0.65
9	-6	-3	-3.01	1.62
9	-6	-3	-0.70	1.29
3	-9	3	0.38	0.22
3	-9	3	0.29	0.23
6	3	3	-0.06	0.45
8	-6	-3	12.02	2.16
6	2	3	13.12	1.67
2	-8	3	16.24	1.36
7	-6	-3	2.19	1.05
1	-7	3	4.70	0.51
6	1	3	3.43	0.69
6	1	3	3.30	0.76
6	-6	-3	0.55	0.72
6	-6	-3	-0.34	0.76
0	6	-3	0.03	0.23
6	-6	-3	0.28	0.24
-6	6	3	0.49	0.26
0	-6	3	0.24	0.13
6	0	3	0.07	0.25
6	0	3	0.21	0.38
-6	6	3	0.44	0.19
1	5	-3	17.56	1.79
5	-6	-3	17.72	1.63
5	-6	-3	19.14	2.18
6	-1	3	16.10	1.89
-5	6	3	18.81	1.60
6	-1	3	18.10	1.79
2	4	-3	57.46	5.00
4	-6	-3	51.13	4.91
6	-2	3	63.41	5.07
6	-2	3	58.05	5.13
-4	6	3	59.67	4.91
3	3	-3	-0.10	0.26
3	-6	-3	-0.09	0.19
6	-3	3	0.38	0.34
-3	6	3	1.06	0.42
2	-6	-3	87.01	6.67
4	2	-3	65.62	6.83
2	-6	-3	79.36	6.59
6	-4	3	73.05	6.72
6	-4	3	86.01	6.77
1	-6	-3	24.54	2.15
1	-6	-3	26.00	2.20
1	-6	-3	23.01	2.37
5	1	-3	23.44	2.76
6	-5	3	27.30	2.32
6	0	-3	0.30	0.68
-6	5	-1	29.36	2.18
-6	5	-1	29.24	2.16
6	-5	0	113.73	7.48
6	-5	0	107.08	7.39
6	-5	0	112.02	7.54
-6	5	0	109.28	7.45
-6	5	0	106.97	7.47
6	-5	-1	29.85	2.36
6	-5	-1	28.17	2.02
6	-5	-1	27.74	2.14
-6	5	1	28.70	2.29
6	-5	-2	27.76	2.15
6	-5	-2	35.63	2.51
-6	5	2	29.75	2.27
6	-5	-3	15.12	1.25
6	-5	-3	16.20	1.20
-6	5	3	17.02	1.43
-6	5	3	19.40	1.57
6	-5	-4	48.50	3.41
6	-5	-4	50.21	3.45
-6	5	4	55.80	3.71
-6	5	4	46.24	3.52
6	-5	-5	3.81	0.66
6	-5	-5	5.85	0.64
6	-5	-5	5.08	0.59
-6	5	5	5.74	0.82
-6	5	5	4.52	0.66
6	-5	-6	12.75	1.11
-6	5	6	15.23	1.56
6	-5	-7	1.73	0.41
6	-5	-8	8.76	1.19
7	-6	8	8.65	1.51
7	-6	7	0.08	1.09
7	-6	6	7.70	1.38
7	-6	6	5.68	0.79
7	-6	5	0.98	1.07
7	-6	5	1.06	0.36
7	-6	4	37.52	2.95
7	-6	4	48.70	3.44
7	-6	3	4.37	0.56
7	-6	3	3.57	0.87
-7	6	-3	3.96	0.65
7	-6	2	12.89	1.59
7	-6	2	11.95	1.03
-7	6	-2	13.58	1.17
-7	6	-2	12.24	1.17
7	-6	1	8.59	0.99
7	-6	1	6.60	0.68
7	-6	1	7.34	0.92
-7	6	-1	6.55	0.97
-7	6	-1	6.55	0.92
7	-6	0	69.95	5.24
7	-6	0	77.58	5.37
7	-6	0	84.91	5.46
-7	6	0	76.56	5.32
7	-6	-1	6.99	1.20
7	-6	-1	7.07	0.97
7	-6	-1	6.33	0.68
-7	6	1	6.92	0.89
7	-6	-2	11.98	1.04
7	-6	-2	14.22	1.50
-7	6	2	13.14	1.24
7	-6	-3	3.90	0.53
-7	6	3	4.04	0.69
7	-6	-4	36.24	2.60
-7	6	4	37.27	2.79
7	-6	-5	1.05	0.31
7	-6	-5	0.69	0.30
-7	6	5	0.70	0.59
-7	6	5	0.17	0.66
7	-6	-6	6.24	0.68
-7	6	6	5.21	0.82
-7	6	6	5.97	0.95
7	-6	-7	0.35	0.41
-7	6	7	-0.27	0.61
7	-6	-8	5.95	0.84
8	-7	7	-1.44	1.38
8	-7	6	17.49	2.08
8	-7	6	13.27	1.31
8	-7	5	0.02	0.34
8	-7	5	-0.92	0.85
8	-7	4	1.89	0.40
8	-7	4	0.54	1.07
8	-7	3	0.12	0.30
8	-7	3	-0.33	0.93
-8	7	-3	0.54	0.35
8	-7	2	56.91	4.06
8	-7	2	52.05	3.77
8	-7	1	0.36	0.29
8	-7	1	0.08	0.49
8	-7	1	0.07	0.53
-8	7	-1	0.12	0.45
8	-7	0	2.15	0.65
8	-7	0	1.85	0.36
8	-7	0	2.63	0.67
-8	7	0	1.65	0.49
8	-7	-1	0.47	0.64
8	-7	-1	0.39	0.28
-8	7	1	0.22	0.44
8	-7	-2	48.78	3.58
-8	7	2	54.56	3.73
8	-7	-3	0.13	0.67
8	-7	-3	0.27	0.27
-8	7	3	0.85	0.52
8	-7	-4	2.17	0.40
-8	7	4	2.69	0.77
8	-7	-5	0.36	0.31
-8	7	5	-0.07	0.73
-8	7	6	13.16	1.74
-8	7	7	-0.49	0.78
9	-8	5	0.14	1.42
9	-8	5	1.15	0.44
9	-8	4	6.97	0.88
9	-8	4	6.04	1.52
9	-8	3	3.16	1.20
9	-8	3	2.36	0.46
9	-8	2	7.65	1.17

0	-6	-3	0.38	0.12
0	-6	-3	0.04	0.46
0	-6	-3	0.58	0.17
6	-6	3	0.31	0.26
-1	-6	-3	5.15	0.54
-1	-6	-3	5.10	0.53
-1	-6	-3	4.07	0.92
6	-7	3	5.74	0.80
1	6	3	4.47	0.70
-2	-6	-3	21.18	1.78
6	-8	3	19.68	1.89
2	6	3	17.95	1.87
-3	-6	-3	1.08	0.66
9	-3	-3	0.18	0.77
6	-9	3	0.17	0.29
3	6	3	0.27	0.35
10	-4	-3	2.33	1.22
-4	-6	-3	2.47	0.83
6	-10	3	2.66	0.46
4	6	3	2.46	0.61
11	-5	-3	0.36	1.66
-5	-6	-3	0.46	0.89
5	6	3	-0.58	0.66
6	-11	3	0.40	0.38
10	-7	-3	0.27	1.52
10	-7	-3	-1.30	1.99
7	3	3	0.56	0.61
9	-7	-3	-1.67	1.32
9	-7	-3	-1.42	1.68
2	-9	3	0.26	0.19
7	2	3	-0.24	0.54
7	1	3	-0.03	0.39
1	-8	3	0.30	0.16
0	7	-3	0.86	0.35
7	-7	-3	-1.09	0.91
7	0	3	0.60	0.32
7	0	3	0.23	0.49
1	6	-3	3.68	0.78
6	-7	-3	3.60	0.51
-6	7	3	4.68	0.54
-1	-6	3	3.86	0.49
7	-1	3	2.49	0.66
2	5	-3	0.49	0.29
5	-7	-3	0.54	0.20
-5	7	3	0.86	0.20
7	-2	3	0.54	0.36
7	-2	3	-0.19	0.44
3	4	-3	4.87	0.86
4	-7	-3	2.81	0.49
7	-3	3	3.45	0.63
7	-3	3	2.57	0.65
-4	7	3	3.62	0.49
3	-7	-3	5.12	0.57
-7	4	-3	6.54	1.05
3	-7	-3	5.05	0.75
4	3	-3	6.10	1.18
2	-7	-3	0.87	0.17
2	-7	-3	0.69	0.39
5	2	-3	1.06	0.67
7	-5	3	1.54	0.44
7	-5	3	0.52	0.45
1	-7	-3	6.08	0.58
1	-7	-3	3.96	0.84
1	-7	-3	5.85	0.77
7	-6	3	5.82	0.97
0	-7	-3	0.71	0.70
7	0	-3	0.25	0.84
0	-7	-3	1.13	0.25
0	-7	-3	1.37	0.30
7	-7	3	1.46	0.43
8	-1	-3	0.01	0.70
-1	-7	-3	0.19	0.19
-1	-7	-3	0.66	0.65
-1	-7	-3	0.25	0.17
7	-8	3	0.48	0.33
9	-2	-3	0.23	0.77
-2	-7	-3	-0.28	0.66
-2	-7	-3	0.11	0.15
2	7	3	0.31	0.45
7	-9	3	0.05	0.31
-3	-7	-3	1.73	0.77
10	-3	-3	-0.04	1.04
3	7	3	2.14	0.65
10	-8	-3	-2.26	2.03
2	-10	3	0.09	0.35
8	2	3	-0.61	0.59
2	-10	3	0.35	0.21
9	-8	-3	-1.14	1.64
9	-8	-3	2.21	1.55
8	1	3	1.11	0.64
8	-8	-3	2.45	1.55
8	0	3	3.78	0.88
8	0	3	3.61	0.72
0	-8	3	5.58	0.59
1	7	-3	0.44	0.41
7	-8	-3	0.01	0.28
7	-8	-3	0.49	0.95
-1	-7	3	0.36	0.19
8	-1	3	-0.12	0.56
6	-8	-3	16.23	2.38
2	6	-3	16.73	1.63
6	-8	-3	13.10	1.34
8	-2	3	12.32	2.01
8	-2	3	12.97	1.68
-6	8	3	15.37	1.37
3	5	-3	7.47	1.22
5	-8	-3	5.57	0.72
5	-8	-3	9.07	1.59
8	-3	3	5.81	1.24
8	-3	3	5.36	1.19
-5	8	3	8.02	0.77
4	4	-3	0.08	0.49
4	-8	-3	-0.44	0.52
4	-8	-3	-0.20	0.21
4	-8	-3	0.06	0.34
8	-4	3	-0.42	0.43
8	-4	3	-0.89	0.61
9	-8	2	8.18	0.85
9	-8	2	7.61	1.07
9	-8	1	4.32	0.80
9	-8	1	3.19	0.50
9	-8	1	4.26	0.79
-9	8	-1	3.73	0.71
9	-8	0	17.56	1.73
9	-8	0	14.67	1.28
-9	8	0	15.21	1.49
9	-8	-1	4.41	0.95
9	-8	-1	2.94	0.47
-9	8	1	3.35	0.74
9	-8	-2	7.63	0.82
9	-8	-2	10.39	1.47
-9	8	2	6.58	1.03
9	-8	-3	2.09	0.41
-9	8	3	1.94	0.78
9	-8	-4	7.67	0.92
-9	8	4	9.80	1.31
9	-8	-5	0.95	0.38
-9	8	5	0.53	0.91
10	-9	3	-0.58	1.14
10	-9	3	0.74	0.43
10	-9	2	1.40	0.65
10	-9	2	0.96	1.03
10	-9	2	1.19	0.43
10	-9	1	1.08	0.69
10	-9	1	1.18	0.41
10	-9	0	17.38	1.70
10	-9	0	12.28	1.24
-10	9	0	12.25	1.60
10	-9	-1	1.39	0.82
10	-9	-1	1.21	0.41
-10	9	1	1.14	0.82
10	-9	-2	1.18	0.42
-10	9	2	1.45	0.85
10	-9	-3	0.39	0.37
-10	9	3	0.06	0.86
-8	10	4	13.35	1.50
8	-10	-4	12.98	1.18
-8	10	3	0.21	0.67
8	-10	-3	0.01	0.32
-8	10	2	-0.08	0.60
8	-10	-2	0.07	0.31
-8	10	1	0.29	0.59
8	-10	-1	-0.01	0.30
-8	10	0	28.24	2.14
8	-10	0	25.24	1.97
8	-10	1	0.19	0.31
8	-10	1	0.29	0.63
8	-10	2	-0.10	0.78
8	-10	2	-0.07	0.31
8	-10	3	-0.12	0.33
8	-10	3	0.12	0.92
8	-10	4	12.83	1.21
8	-10	4	18.07	2.22
-7	9	6	6.35	0.93
-7	9	5	-0.35	0.61
7	-9	-5	-0.31	0.28
-7	9	4	4.88	0.69
7	-9	-4	5.24	0.64
-7	9	3	-0.10	0.49
7	-9	-3	0.02	0.27
-7	9	2	18.83	1.63
7	-9	-2	18.27	1.42
-7	9	1	0.07	0.38
7	-9	-1	0.23	0.25
-7	9	0	9.20	0.97
7	-9	0	9.80	1.22
7	-9	0	7.99	0.80
-7	9	-1	-0.03	0.47
7	-9	1	-0.13	0.52
7	-9	1	0.26	0.28
7	-9	2	19.12	1.80
7	-9	2	18.17	1.44
7	-9	3	0.01	0.27
7	-9	3	0.62	0.76
7	-9	4	4.67	1.01
7	-9	4	5.46	0.66
7	-9	5	-0.17	0.31
7	-9	5	0.61	0.70
7	-9	6	6.41	0.96
7	-9	6	7.86	1.39
-6	8	7	1.80	0.64
6	-8	-7	2.15	0.64
-6	8	6	13.06	1.41
6	-8	-6	13.06	1.19
-6	8	5	7.45	1.04
6	-8	-5	5.84	0.71
6	-8	-5	5.68	0.63
-6	8	4	7.32	1.00
6	-8	-4	6.35	0.67
6	-8	-4	7.52	0.73
-6	8	3	14.37	1.24
6	-8	-3	14.44	1.13
6	-8	-3	13.95	1.11
-6	8	2	45.48	3.23
6	-8	-2	46.13	3.16
-6	8	1	24.85	1.89
6	-8	-1	30.15	2.12
6	-8	-1	22.48	1.80
-6	8	0	7.22	0.82
6	-8	0	7.51	0.69
6	-8	0	10.19	1.24
6	-8	0	7.23	0.76
-6	8	-1	24.28	1.91
6	-8	1	26.48	2.01
6	-8	1	24.96	1.82
6	-8	1	24.30	1.79
-6	8	-2	47.94	3.40
6	-8	2	48.28	3.32
6	-8	2	52.85	3.50
6	-8	2	43.54	3.30
6	-8	3	14.33	1.16
6	-8	3	14.44	1.16
6	-8	3	15.88	1.49
6	-8	4	7.93	0.73
3	-8	-3	9.98	0.95
8	-5	3	9.50	1.69
8	-5	3	9.04	1.73
2	-8	-3	17.32	2.06
2	-8	-3	20.08	1.82
2	-8	-3	24.35	1.98
6	2	-3	18.88	3.04
7	1	-3	-0.49	1.01
1	-8	-3	0.60	0.62
1	-8	-3	0.43	0.20
1	-8	-3	0.50	0.29
8	-7	3	0.75	0.46
0	-8	-3	6.75	1.14
0	-8	-3	8.60	0.89
-1	-8	-3	2.17	0.83
8	-9	3	3.06	0.59
10	-2	-3	-0.74	0.94
-2	-8	-3	0.87	0.77
8	-10	3	-0.18	0.42
10	-9	-3	-2.86	2.09
10	-9	-3	-0.31	1.94
9	1	3	-0.55	0.64
9	-9	-3	-1.94	1.52
9	-9	-3	-1.40	1.31
9	0	3	-0.34	0.48
0	-9	3	0.04	0.18
1	8	-3	2.30	0.62
8	-9	-3	3.25	1.42
-1	-8	3	1.47	0.27
9	-1	3	1.14	0.54
9	-1	3	1.35	0.84
7	-9	-3	0.59	0.87
7	-9	-3	1.15	0.32
2	7	-3	0.30	0.46
9	-2	3	-0.01	0.47
9	-2	3	-0.60	0.75
-2	-7	3	-0.05	0.19
9	-3	3	-0.07	0.83
5	-9	-3	-0.09	0.25
4	5	-3	0.50	0.63
5	-9	-3	-0.54	0.68
9	-4	3	-1.05	0.59
4	-9	-3	-0.61	0.44
5	4	-3	-0.54	0.85
4	-9	-3	-0.02	0.51
9	-5	3	-0.22	0.77
9	-5	3	-0.72	0.61
3	-9	-3	-0.10	0.36
3	-9	-3	0.45	0.43
6	3	-3	-0.39	1.08
9	-6	3	0.05	0.58
9	-6	3	-0.22	0.75
2	-9	-3	0.17	0.49
7	2	-3	-0.23	1.19
9	-7	3	-0.38	0.56
9	-7	3	0.48	0.59
1	-9	-3	2.72	0.81
8	1	-3	1.68	1.16
9	-8	3	4.09	0.78
0	-9	-3	-0.14	0.75
9	0	-3	-0.20	1.08
9	-9	3	0.97	0.64
10	-1	-3	-0.54	1.31
-1	-9	-3	1.19	0.93
9	-10	3	1.19	0.73
9	-10	-3	-0.70	1.65
9	-10	-3	1.75	1.52
10	-1	3	-0.18	0.94
-1	-9	3	0.71	0.29
8	-10	-3	0.58	1.18
2	8	-3	0.36	0.72
-2	-8	3	-0.04	0.24
10	-2	3	-0.01	0.57
10	-2	3	-1.01	0.98
7	-10	-3	5.99	1.37
6	-10	-3	5.88	1.59
6	-10	-3	2.12	0.40
4	6	-3	3.73	1.11
10	-4	3	1.34	1.20
10	-4	3	1.11	0.81
5	5	-3	-0.65	1.03
5	-10	-3	-0.22	0.30
10	-5	3	-0.55	1.02
10	-5	3	-0.91	0.76
6	4	-3	3.10	1.47
4	-10	-3	2.07	0.47
10	-6	3	4.19	1.10
10	-6	3	5.24	1.33
7	3	-3	1.89	1.65
3	-10	-3	2.39	0.58
10	-7	3	3.38	1.03
10	-7	3	3.59	1.06
8	2	-3	-1.35	1.49
2	-10	-3	-0.10	0.57
10	-8	3	0.12	0.88
10	-8	3	0.00	0.82
9	1	-3	0.57	1.54
1	-10	-3	0.35	0.68
10	-9	3	1.69	0.93
10	-9	3	1.69	0.93
5	6	-3	-0.48	1.52
6	-11	-3	-0.15	0.34
11	-5	3	-2.00	1.42
11	-5	3	-0.39	1.06
6	5	-3	-0.37	1.76
5	-11	-3	-0.11	0.35
11	-6	3	-1.21	1.16
11	-6	3	-2.57	1.74
-1	0	-4	329.63	25.46
0	1	-4	284.98	25.42
1	-1	-4	281.72	25.44
0	-1	4	301.43	25.43
0	-1	4	303.37	25.44
-1	1	4	323.44	25.44
0	-1	-4	299.43	24.61
-1	1	-4	302.70	24.57
1	0	-4	273.02	24.58
6	-8	4	7.65	0.97
6	-8	4	6.29	0.70
6	-8	5	6.76	1.11
6	-8	5	6.41	0.70
6	-8	5	5.32	0.63
6	-8	6	13.62	2.13
6	-8	6	11.98	1.12
6	-8	7	1.21	1.15
5	-7	-8	5.42	0.91
-5	7	7	0.26	0.45
5	-7	-7	-0.13	0.50
-5	7	6	15.90	1.37
5	-7	-6	15.07	1.31
-5	7	5	0.38	0.41
5	-7	-5	0.62	0.33
5	-7	-5	0.75	0.23
-5	7	4	24.41	1.86
5	-7	-4	25.52	1.82
5	-7	-4	23.56	1.74
-5	7	3	0.56	0.34
5	-7	-3	0.98	0.22
-5	7	2	39.69	3.13
5	-7	-2	45.57	3.10
5	-7	-2	51.41	3.39
-5	7	1	0.98	0.34
5	-7	-1	0.92	0.52
5	-7	-1	0.55	0.26
5	-7	-1	0.70	0.31
5	-7	-1	0.54	0.21
-5	7	0	42.57	3.04
5	-7	0	42.89	2.97
5	-7	0	45.97	3.17
5	-7	0	42.14	3.00
-5	7	-1	0.62	0.28
-5	7	-1	0.64	0.35
5	-7	1	0.89	0.22
5	-7	1	0.56	0.24
5	-7	1	0.38	0.47
-5	7	-2	46.35	3.25
-5	7	-2	49.19	3.35
5	-7	2	44.55	3.16
5	-7	2	47.72	3.31
5	-7	2	43.89	3.17
5	-7	3	0.82	0.48
5	-7	3	0.74	0.23
5	-7	3	0.39	0.21
5	-7	4	23.48	1.63
5	-7	4	22.57	2.02
5	-7	4	21.91	1.64
5	-7	5	0.74	0.25
5	-7	5	0.36	0.22
5	-7	5	1.05	0.74
5	-7	6	15.76	1.26
5	-7	6	15.85	1.89
5	-7	7	0.71	0.89
5	-7	8	6.35	1.24
4	-6	-9	0.97	0.84
4	-6	-8	4.28	0.82
4	-6	-7	4.31	0.67
-4	6	6	42.79	2.91
4	-6	-6	39.76	2.94
-4	6	5	20.00	1.51
4	-6	-5	19.82	1.53
4	-6	-5	19.63	1.41
-4	6	4	8.27	0.82
4	-6	-4	8.68	0.69
-4	6	3	53.37	3.66
4	-6	-3	53.13	3.61
-4	6	2	183.45	12.43
4	-6	-2	187.37	12.33
4	-6	-2	183.60	12.48
-4	6	1	88.72	6.25
-4	6	1	93.96	6.26
4	-6	-1	100.94	6.28
4	-6	-1	85.26	6.31
4	-6	-1	96.67	6.26
4	-6	-1	90.07	6.21
-4	6	0	8.27	0.72
-4	6	0	7.27	0.66
-4	6	0	7.30	0.74
4	-6	0	8.45	0.69
4	-6	0	7.88	0.73
4	-6	0	8.44	0.98
4	-6	0	8.23	0.63
-4	6	-1	94.43	6.53
-4	6	-1	103.81	6.52
-4	6	-1	95.10	6.55
4	-6	1	93.04	6.50
4	-6	1	94.74	6.53
4	-6	1	100.47	6.60
-4	6	-2	182.63	11.83
-4	6	-2	175.76	11.79
4	-6	2	171.29	11.78
4	-6	2	172.97	11.78
4	-6	2	179.66	11.81
-4	6	-3	56.55	3.84
4	-6	3	55.87	3.83
4	-6	3	55.87	3.96
4	-6	3	57.10	3.82
-4	6	-4	8.68	0.77
4	-6	4	9.36	1.16
4	-6	4	9.13	0.73
4	-6	4	8.72	0.72
-4	6	-5	19.82	1.52
4	-6	5	20.22	1.48
4	-6	5	21.31	1.50
4	-6	5	22.19	1.82
-4	6	-6	43.10	3.15
4	-6	6	48.54	3.39
4	-6	6	43.15	3.12
4	-6	6	48.41	3.19
-4	6	-7	4.92	0.61
4	-6	7	4.35	0.85
-4	6	-8	3.61	0.45
-4	6	-9	0.74	0.32
3	-5	-9	-0.02	0.17
3	-5	-9	-0.07	0.80

0	1	4	261.35	24.55
1	-1	4	290.87	24.58
1	-1	4	313.81	24.57
-1	0	4	315.67	24.60
-1	-1	-4	46.25	3.53
-1	-1	-4	45.50	3.63
2	-1	-4	39.22	3.57
2	-1	-4	37.60	3.56
1	-2	4	40.54	3.54
1	-2	4	41.26	3.54
1	-2	4	41.35	3.55
-2	1	4	41.68	3.55
-2	-1	-4	170.38	15.75
3	-2	-4	193.68	15.80
3	-2	-4	172.78	15.70
2	1	4	187.51	15.69
1	-3	4	190.30	15.68
2	1	4	181.43	15.68
1	-3	4	194.04	15.68
1	-3	4	188.01	15.69
-3	2	4	205.55	15.69
-2	0	-4	20.18	1.85
0	2	-4	17.76	1.80
2	-2	-4	21.39	1.82
2	0	4	22.32	1.83
0	-2	4	19.41	1.80
0	-2	4	19.24	1.81
-2	2	4	23.67	1.81
-2	2	4	22.22	1.82
0	-2	4	21.11	1.80
1	1	-4	36.35	3.40
1	-2	-4	38.32	3.44
-2	1	-4	40.30	3.45
-1	-1	4	40.76	3.40
-1	2	4	41.98	3.37
2	-1	4	46.32	3.41
-1	-1	4	38.95	3.41
2	-1	4	36.70	3.41
0	-2	-4	19.62	1.73
-2	2	-4	19.21	1.71
-2	2	-4	19.56	1.63
2	0	-4	18.92	1.68
2	-2	4	19.04	1.66
2	-2	4	17.65	1.67
2	-2	4	18.36	1.66
-2	0	4	20.21	1.67
-2	0	4	18.95	1.67
-1	-2	-4	224.88	16.68
-2	3	-4	187.02	16.75
-1	-2	-4	216.16	16.86
3	-1	-4	195.40	16.79
-3	1	4	187.49	16.70
2	-3	4	192.25	16.72
2	-3	4	179.26	16.68
1	2	4	227.08	16.73
2	-3	4	185.66	16.71
-2	-2	-4	466.46	38.88
4	-2	-4	437.86	38.91
2	-4	4	482.43	38.69
2	-4	4	476.80	38.68
-4	2	4	472.91	38.71
2	2	4	387.99	38.67
2	2	4	513.65	38.75
2	-5	4	123.67	10.63
2	-5	4	120.52	10.59
3	2	4	136.72	10.72
-5	3	4	122.82	10.59
4	-3	-4	158.39	14.45
4	-3	-4	173.24	14.21
-3	-1	-4	151.59	14.37
1	-4	4	171.34	14.17
3	1	4	150.92	14.21
-4	3	4	196.47	14.18
1	-4	4	178.96	14.18
3	-3	-4	55.37	4.77
3	-3	-4	52.48	4.53
0	3	-4	49.41	4.52
0	-3	4	50.03	4.50
3	0	4	50.89	4.57
0	-3	4	58.53	4.51
3	0	4	55.50	4.55
2	-3	-4	173.00	15.12
-3	1	-4	173.52	15.20
1	2	-4	163.98	15.10
-2	3	4	211.58	15.07
3	-1	4	169.42	15.11
-1	-2	4	188.42	15.09
1	-3	-4	160.79	14.77
2	1	-4	173.37	14.76
-3	2	-4	180.20	14.84
-2	-1	4	181.56	14.76
-2	-1	4	186.21	14.74
3	-2	4	172.64	14.76
0	-3	-4	56.86	4.60
3	0	-4	52.68	4.56
-3	3	-4	51.29	4.56
3	-3	4	52.24	4.43
-3	0	4	51.89	4.48
3	-3	4	51.79	4.50
-3	4	-4	163.18	14.43
4	-1	-4	173.61	14.58
-1	-3	-4	180.57	14.55
-4	1	4	162.39	14.34
3	-4	4	188.67	14.43
3	-4	4	176.76	14.38
1	3	4	167.64	14.30
3	-4	4	156.58	14.30
5	-2	-4	101.86	9.75
3	-5	4	111.72	9.42
3	-5	4	124.74	9.39
3	-5	4	106.03	9.30
2	3	4	110.18	9.33
6	-3	-4	4.67	1.06
-3	-3	-4	5.20	0.75
3	-6	4	7.41	0.62
3	3	4	6.98	0.71
-4	-3	-4	28.77	2.60

3	-5	-8	15.48	1.58
3	-5	-8	15.34	1.12
3	-5	-7	0.36	0.40
3	-5	-7	0.23	0.16
3	-5	-6	18.89	1.36
3	-5	-6	19.91	1.53
-3	5	5	0.94	0.26
3	-5	-5	0.81	0.18
3	-5	-5	0.96	0.18
3	-5	-5	0.76	0.38
-3	5	4	94.21	6.44
-3	5	4	96.73	6.46
3	-5	-4	94.42	6.41
3	-5	-4	97.90	6.41
-3	5	3	4.62	0.48
-3	5	3	3.73	0.45
-3	5	3	3.75	0.45
3	-5	-3	3.96	0.36
3	-5	-3	3.69	0.38
-3	5	2	58.94	3.76
-3	5	2	57.50	3.75
-3	5	2	55.99	3.76
3	-5	-2	48.12	3.83
3	-5	-2	54.94	3.71
3	-5	-2	56.22	3.77
3	-5	-2	57.56	3.72
3	-5	-2	51.65	3.78
-3	5	1	5.48	0.51
-3	5	1	5.74	0.47
3	-5	-1	4.41	0.68
3	-5	-1	6.03	0.55
3	-5	-1	5.79	0.52
-3	5	0	182.94	12.11
-3	5	0	188.35	12.13
-3	5	0	185.96	12.14
3	-5	0	184.86	12.11
3	-5	0	192.29	12.10
3	-5	0	173.48	12.12
3	-5	0	173.65	12.14
3	-5	0	171.75	12.16
-3	5	-1	6.18	0.55
-3	5	-1	5.65	0.56
-3	5	-1	6.34	0.51
3	-5	1	6.10	0.51
3	-5	1	5.88	0.48
3	-5	1	5.66	0.66
3	-5	1	5.76	0.53
-3	5	-2	59.28	3.73
-3	5	-2	46.65	3.72
3	-5	2	53.97	3.73
3	-5	2	57.96	3.73
3	-5	2	54.63	3.71
3	-5	2	58.38	3.80
-3	5	-3	4.16	0.43
3	-5	3	3.93	0.42
3	-5	3	4.34	0.43
3	-5	3	4.40	0.40
3	-5	3	3.52	0.53
-3	5	-4	102.34	6.76
3	-5	4	104.32	6.75
3	-5	4	96.43	6.75
3	-5	4	99.75	6.84
-3	5	-5	1.42	0.25
3	-5	5	0.62	0.45
3	-5	5	0.82	0.20
3	-5	5	1.02	0.22
-3	5	-6	19.41	1.48
3	-5	6	20.55	1.67
3	-5	6	21.50	1.46
-3	5	-7	0.10	0.22
-3	5	-8	15.59	1.19
-3	5	-9	-0.05	0.23
2	-4	-10	2.09	0.91
2	-4	-10	1.70	0.26
2	-4	-9	0.31	0.15
2	-4	-9	-0.09	0.75
2	-4	-8	39.66	2.73
2	-4	-8	41.29	3.04
2	-4	-7	-0.04	0.32
2	-4	-7	0.00	0.13
2	-4	-6	0.80	0.16
2	-4	-6	0.42	0.32
-2	4	5	0.04	0.36
2	-4	-5	-0.16	0.34
2	-4	-5	-0.10	0.10
2	-4	-5	0.17	0.13
-2	4	4	369.40	24.84
-2	4	4	331.11	24.91
2	-4	-4	396.92	24.84
2	-4	-4	398.16	24.84
-2	4	3	0.07	0.20
-2	4	3	-0.01	0.09
-2	4	3	-0.09	0.14
2	-4	-3	-0.05	0.09
2	-4	-3	0.05	0.23
2	-4	-3	0.00	0.09
-2	4	2	42.62	2.86
-2	4	2	43.99	2.85
-2	4	2	42.98	2.89
2	-4	-2	35.59	2.89
2	-4	-2	40.42	2.87
2	-4	-2	41.82	2.83
2	-4	-2	45.70	2.88
2	-4	-2	42.91	2.83
2	-4	-2	41.40	2.89
-2	4	1	0.15	0.14
-2	4	1	0.02	0.10
-2	4	1	0.12	0.10
2	-4	-1	0.34	0.16
2	-4	-1	-0.10	0.23
2	-4	-1	0.15	0.09
-2	4	0	958.97	58.07
-2	4	0	932.88	58.12
-2	4	0	831.04	58.18
-2	4	0	899.69	57.98
2	-4	0	911.24	57.93
2	-4	0	927.49	58.07

7	-4	-4	28.32	3.44
3	-7	4	33.19	2.59
4	3	4	26.39	2.61
6	-4	-4	6.66	1.27
-4	-2	-4	8.11	0.96
2	-6	4	9.29	0.85
4	2	4	8.16	0.95
5	-4	-4	20.99	2.46
1	-5	4	21.17	1.74
4	1	4	17.53	1.87
4	1	4	18.98	1.82
-4	0	-4	12.64	1.82
4	-4	-4	16.10	2.06
4	-4	-4	19.03	1.52
0	4	-4	14.96	1.51
4	0	4	15.34	1.57
4	0	4	16.14	1.55
0	-4	4	18.99	1.48
0	-4	4	16.67	1.47
1	3	-4	152.45	13.32
-4	1	-4	135.88	13.45
3	-4	-4	158.59	13.32
3	-4	-4	167.52	13.88
-3	4	4	146.75	13.25
4	-1	4	161.73	13.33
4	-1	4	168.44	13.37
-1	-3	4	176.28	13.30
2	-4	-4	380.17	36.16
-4	2	-4	365.22	36.28
2	-4	-4	478.61	36.24
2	2	-4	421.89	36.17
4	-2	4	476.41	36.19
-2	-2	4	469.90	36.14
1	-4	-4	162.39	14.60
3	1	-4	172.59	14.68
1	-4	-4	153.66	14.65
-4	3	-4	151.74	14.73
-4	3	-4	192.47	14.55
1	-4	-4	189.57	14.54
4	-3	4	182.73	14.64
-3	-1	4	185.83	14.64
0	-4	-4	16.71	1.84
4	0	-4	19.04	1.93
-4	0	4	19.25	1.73
4	-4	4	18.48	1.69
-1	-4	-4	18.84	2.26
5	-1	-4	21.78	2.48
4	-5	4	22.59	1.94
2	4	4	11.48	1.07
4	-6	4	11.29	1.14
7	-3	-4	24.04	3.20
-3	-4	-4	26.44	2.40
3	4	4	29.91	2.44
8	-4	-4	46.26	5.50
4	4	4	56.80	4.70
4	-8	4	56.36	4.54
9	-5	-4	8.75	1.78
-5	-4	-4	6.54	1.19
5	4	4	9.64	1.14
3	-8	4	35.99	3.14
-5	-2	-4	26.74	2.32
7	-5	-4	20.98	3.29
2	-7	4	26.96	2.27
5	2	4	22.36	2.29
6	-5	-4	56.29	5.40
1	-6	4	58.08	4.65
5	1	4	54.23	4.81
5	1	4	49.99	4.74
5	-5	-4	47.45	3.73
0	5	-4	44.09	3.76
5	-5	-4	45.52	4.29
-5	0	-4	36.73	3.93
5	0	4	40.69	3.83
0	-5	4	45.23	3.68
5	0	4	41.88	3.78
1	4	-4	18.64	1.81
4	-5	-4	20.92	2.30
-5	1	-4	19.32	2.05
-5	1	-4	18.01	2.15
4	-5	-4	22.84	1.80
5	-1	4	19.28	1.92
-4	5	4	19.53	1.73
5	-1	4	20.34	1.86
2	3	-4	107.09	8.96
3	-5	-4	112.17	9.12
-5	2	-4	100.30	9.12
3	-5	-4	93.53	8.91
-3	5	4	103.95	8.85
5	-2	4	109.79	9.00
5	-2	4	112.41	8.97
2	-5	-4	134.65	10.20
-5	3	-4	104.74	10.45
3	2	-4	112.58	10.33
5	-3	4	138.94	10.33
-3	-2	4	116.70	10.28
1	-5	-4	26.42	2.14
4	1	-4	22.78	2.45
5	-4	4	25.62	2.27
5	0	-4	51.71	5.08
0	-5	-4	48.87	4.82
0	-5	-4	59.84	4.56
5	-5	4	53.63	4.65
6	-1	-4	55.66	5.63
-1	-5	-4	53.21	5.32
5	-6	4	66.40	5.09
7	-2	-4	21.56	3.01
-2	-5	-4	21.20	1.88
8	-3	-4	26.51	3.65
3	5	4	32.28	2.89
5	-8	4	36.16	2.91
9	-4	-4	9.14	1.73
-4	-5	-4	7.60	1.34
4	5	4	11.49	1.25
-5	-5	-4	0.60	0.82
10	-5	-4	-0.72	1.31
5	-10	4	0.35	0.28
5	5	4	0.70	0.89

2	-4	0	757.28	57.91
2	-4	0	789.42	58.02
2	-4	0	843.76	58.05
-2	4	-1	0.49	0.14
2	-4	1	0.22	0.22
2	-4	1	0.94	0.15
-2	4	-2	45.63	3.04
2	-4	2	44.48	3.03
2	-4	2	47.70	3.05
2	-4	2	43.84	3.05
2	-4	2	43.14	3.10
-2	4	-3	0.06	0.12
2	-4	3	0.17	0.10
2	-4	3	0.26	0.27
2	-4	3	0.32	0.13
2	-4	3	-0.03	0.15
-2	4	-4	397.87	25.83
2	-4	4	366.96	25.86
2	-4	4	393.74	25.83
2	-4	4	395.93	25.83
-2	4	-5	0.61	0.17
2	-4	5	0.07	0.14
2	-4	5	-0.11	0.14
-2	4	-6	0.99	0.21
-2	4	-6	1.43	0.87
2	-4	6	1.05	0.20
-2	4	-7	0.05	0.17
-2	4	-7	-0.39	0.96
-2	4	-8	40.67	2.79
-2	4	-8	40.91	3.48
-2	4	-9	-1.23	1.53
-2	4	-9	0.15	0.20
-2	4	-10	0.24	1.75
1	-3	-10	4.68	0.86
1	-3	-10	4.83	0.45
1	-3	-9	0.64	0.15
1	-3	-9	1.32	0.56
1	-3	-8	18.68	1.32
1	-3	-8	19.35	1.62
1	-3	-7	1.57	0.45
1	-3	-7	1.84	0.26
1	-3	-6	42.15	2.84
-1	3	5	11.67	0.82
1	-3	-5	11.02	0.79
1	-3	-5	11.09	0.81
-1	3	4	151.94	10.16
1	-3	-4	153.65	10.16
1	-3	-4	152.10	10.15
-1	3	3	28.68	2.45
-1	3	3	35.94	2.30
-1	3	3	35.18	2.31
1	-3	-3	36.99	2.30
1	-3	-3	31.64	2.37
1	-3	-3	35.47	2.30
-1	3	2	126.60	7.82
-1	3	2	126.11	7.83
-1	3	2	123.92	7.82
-1	3	2	110.46	7.90
1	-3	-2	100.18	7.82
1	-3	-2	122.24	7.82
1	-3	-2	120.62	7.81
1	-3	-2	112.36	7.84
1	-3	-2	113.26	7.84
-1	3	1	44.21	3.15
-1	3	1	47.20	3.16
-1	3	1	46.17	3.15
-1	3	1	48.77	3.16
-1	3	1	44.02	3.22
1	-3	-1	47.63	3.15
1	-3	-1	48.02	3.17
1	-3	-1	48.01	3.17
1	-3	-1	45.28	3.15
1	-3	-1	48.33	3.20
-1	3	0	252.21	17.40
-1	3	0	268.76	17.36
-1	3	0	276.21	17.34
-1	3	0	259.27	17.35
1	-3	0	274.34	17.34
1	-3	0	270.53	17.34
1	-3	0	264.33	17.35
1	-3	0	235.36	17.37
1	-3	0	247.88	17.36
-1	3	-1	47.18	3.22
-1	3	-1	47.04	3.20
-1	3	-1	46.09	3.28
1	-3	1	51.09	3.20
1	-3	1	44.47	3.23
1	-3	1	48.19	3.21
1	-3	1	48.17	3.21
1	-3	1	48.04	3.22
-1	3	-2	120.28	7.84
1	-3	2	112.39	7.83
1	-3	2	115.77	7.87
1	-3	2	119.24	7.85
1	-3	2	120.65	7.85
-1	3	-3	37.33	2.44
-1	3	-3	34.76	2.47
1	-3	3	38.99	2.46
1	-3	3	37.68	2.44
1	-3	3	32.32	2.46
-1	3	-4	138.17	9.80
-1	3	-4	150.91	9.68
1	-3	4	147.48	9.69
1	-3	4	144.26	9.67
-1	3	-5	12.69	1.26
-1	3	-5	11.69	0.83
1	-3	5	11.90	0.83
1	-3	5	11.08	0.86
-1	3	-6	38.91	3.09
-1	3	-6	42.40	2.78
1	-3	6	41.40	2.80
-1	3	-7	1.69	0.21
-1	3	-7	1.50	0.83
1	-3	7	1.82	0.24
-1	3	-8	19.26	1.35
-1	3	-8	20.16	2.14
-1	3	-9	0.52	0.16

10	-6	-4	0.81	1.52
-6	-4	-4	2.41	0.87
4	-10	4	1.37	0.35
-6	-3	-4	2.91	0.77
9	-6	-4	2.99	1.66
6	3	4	3.44	0.62
8	-6	-4	3.62	1.88
7	-6	-4	38.98	4.62
1	-7	4	39.82	3.45
6	1	4	41.27	3.71
6	-6	-4	122.97	10.64
6	-6	-4	131.09	10.03
0	6	-4	110.97	10.06
0	-6	4	116.16	9.97
-6	6	4	123.93	10.00
6	0	4	106.53	10.14
1	5	-4	55.11	4.80
5	-6	-4	56.67	5.23
5	-6	-4	54.32	4.75
6	-1	4	54.40	4.91
-5	6	4	57.29	4.70
-1	-5	4	53.93	4.70
6	-1	4	55.75	4.84
-6	2	-4	10.08	1.51
4	-6	-4	8.31	1.51
2	4	-4	9.63	1.06
4	-6	-4	7.93	0.92
6	-2	4	9.06	1.21
6	-2	4	9.17	1.08
-4	6	4	9.62	0.85
3	3	-4	10.61	1.09
3	-6	-4	8.36	0.84
3	-6	-4	6.62	1.32
6	-3	4	8.07	1.10
6	-3	4	8.35	1.04
4	2	-4	11.81	1.54
2	-6	-4	12.73	1.13
6	-4	4	13.48	1.34
6	-4	4	13.01	1.36
1	-6	-4	61.67	6.21
5	1	-4	66.55	6.52
1	-6	-4	74.63	6.03
6	-5	4	82.50	6.18
0	-6	-4	155.25	12.21
6	0	-4	127.99	12.82
0	-6	-4	135.19	12.46
6	-6	4	161.17	12.32
6	-7	4	53.44	4.64
-2	-6	-4	4.85	1.16
2	6	4	8.02	0.97
6	-8	4	7.80	1.00
-3	-6	-4	2.27	0.96
9	-3	-4	2.46	1.15
3	6	4	4.40	0.96
6	-9	4	6.34	0.95
-4	-6	-4	1.81	0.92
4	6	4	1.83	0.91
6	-10	4	2.00	0.41
10	-7	-4	1.81	1.85
7	3	4	3.34	0.75
-7	-2	-4	3.76	0.77
9	-7	-4	-0.33	1.52
7	2	4	2.90	0.71
-7	-1	-4	0.40	0.53
8	-7	-4	-1.32	1.04
8	-7	-4	-0.47	2.34
7	1	4	1.25	0.43
7	-7	-4	13.90	3.20
0	7	-4	17.78	1.89
7	0	4	19.22	2.06
0	-7	4	19.22	1.66
6	-7	-4	40.41	3.35
6	-7	-4	34.94	4.17
1	6	-4	39.87	3.48
-6	7	4	41.25	3.33
7	-1	4	37.24	3.62
7	-1	4	34.51	3.48
-1	-6	4	40.00	3.33
5	-7	-4	20.87	2.68
5	-7	-4	21.92	2.08
7	-2	4	25.40	2.30
7	-2	4	25.62	2.48
-5	7	4	23.42	2.11
3	4	-4	30.25	2.79
4	-7	-4	24.70	2.58
-4	7	4	32.26	2.55
7	-3	4	31.59	2.80
7	-3	4	28.65	2.91
4	3	-4	32.38	3.16
7	-4	4	31.94	3.00
2	-7	-4	36.04	3.21
5	2	-4	31.46	4.41
7	-5	4	38.17	3.55
7	-5	4	42.54	3.45
1	-7	-4	56.93	4.54
6	1	-4	48.13	5.36
0	-7	-4	21.95	2.31
7	0	-4	17.63	3.10
-1	-7	-4	2.07	0.92
8	-1	-4	1.30	1.09
-2	-7	-4	3.78	1.07
-3	-7	-4	4.66	1.11
10	-3	-4	4.58	1.54
10	-8	-4	6.33	2.52
2	-10	4	11.52	1.15
9	-8	-4	3.35	1.81
8	1	4	4.22	0.80
8	-8	-4	-0.55	1.48
-8	0	-4	1.46	0.58
8	-8	-4	-0.96	0.98
8	0	4	1.25	0.64
8	0	4	0.91	0.44
0	-8	4	1.68	0.28
7	-8	-4	0.54	1.21
7	-8	-4	-1.15	0.77
-8	1	-4	-0.33	0.51
8	-1	4	2.23	0.67
-1	3	-9	0.37	1.24
-1	3	-10	5.56	1.75
0	-2	-10	9.09	0.72
0	-2	-10	9.15	1.43
0	-2	-9	2.50	0.34
0	-2	-9	3.68	0.92
0	-2	-8	7.86	0.98
0	-2	-8	7.99	0.64
0	-2	-7	23.04	1.58
0	-2	-7	21.94	1.80
0	2	6	118.33	8.49
0	-2	-6	138.31	8.47
0	-2	-6	123.12	8.57
0	2	5	103.45	6.65
0	-2	-5	102.19	6.67
0	-2	-5	97.95	6.64
0	-2	-5	95.08	6.76
0	2	4	13.94	0.96
0	-2	-4	13.97	0.99
0	2	3	263.97	18.07
0	-2	-3	279.99	18.08
0	2	2	536.24	36.55
0	2	2	548.22	36.54
0	2	2	561.40	36.52
0	-2	-2	581.81	36.51
0	-2	-2	519.43	36.50
0	2	1	535.72	35.72
0	2	1	530.72	35.69
0	2	1	541.53	35.78
0	2	1	488.75	35.66
0	2	1	561.08	35.66
0	2	1	554.62	35.63
0	-2	-1	567.62	35.64
0	-2	-1	565.49	35.65
0	-2	-1	538.84	35.65
0	-2	-1	490.85	35.66
0	-2	-1	520.63	35.66
0	2	0	3.93	0.30
0	2	0	3.92	0.32
0	2	0	3.86	0.30
0	-2	0	4.01	0.30
0	-2	0	4.02	0.31
0	-2	0	3.66	0.37
0	-2	0	3.64	0.30
0	2	-1	529.30	34.40
0	2	-1	511.22	34.35
0	2	-1	559.82	34.39
0	-2	1	519.75	34.40
0	-2	1	482.06	34.34
0	-2	1	498.47	34.38
0	2	-2	549.52	37.21
0	2	-2	498.15	37.19
0	-2	2	633.06	37.27
0	2	-3	280.96	18.17
0	2	-3	259.63	18.07
0	-2	3	259.09	18.10
0	-2	3	288.89	18.08
0	2	-4	14.58	1.00
0	2	-4	14.27	1.23
0	-2	4	14.65	1.04
0	-2	4	14.78	0.99
0	2	-5	99.80	6.65
0	2	-5	95.86	6.48
0	-2	5	94.36	6.52
0	-2	5	99.84	6.49
0	2	-6	128.60	8.83
0	2	-6	140.61	9.04
0	-2	6	130.00	8.88
0	2	-7	25.04	2.10
0	2	-7	23.38	1.64
0	-2	7	25.05	1.71
0	2	-8	7.67	0.56
0	2	-8	8.43	1.16
0	2	-9	2.10	1.13
0	2	-9	2.77	0.26
0	2	-10	9.52	1.72
0	2	-10	8.50	0.63
-1	-1	-10	8.87	1.49
-1	-1	-10	9.59	0.87
-1	-1	-9	-0.13	0.81
-1	-1	-9	-0.11	0.26
-1	-1	-8	9.68	0.97
-1	-1	-8	12.56	1.35
-1	-1	-7	0.36	0.39
-1	-1	-7	0.03	0.47
-1	-1	-6	105.75	6.67
-1	-1	-6	89.96	6.62
-1	-1	-5	1.09	0.38
-1	-1	-5	-0.37	0.27
1	1	4	29.00	1.75
-1	-1	-4	21.53	1.85
1	1	3	0.01	0.08
1	1	3	0.02	0.08
-1	-1	-3	0.67	0.26
1	1	2	457.89	31.52
1	1	2	518.06	31.51
1	1	2	498.33	31.57
1	1	2	534.94	31.54
-1	-1	-2	427.23	31.53
-1	-1	-2	407.44	31.52
1	1	1	0.18	0.17
1	1	1	0.85	0.19
1	1	1	0.45	0.09
1	1	1	0.12	0.10
1	1	1	0.25	0.07
-1	-1	-1	0.06	0.09
-1	-1	-1	0.11	0.14
-1	-1	-1	0.26	0.07
-1	-1	-1	0.87	0.27
-1	-1	-1	0.25	0.16
1	1	0	83.88	5.68
1	1	0	85.94	5.68
1	1	0	85.77	5.68
1	1	0	85.32	5.70
1	1	0	85.05	5.68
1	1	0	83.64	5.68
-1	-1	0	83.88	5.68
2	6	-4	8.01	1.23
6	-8	-4	9.11	1.44
6	-8	-4	6.52	0.79
8	-2	4	5.25	1.04
8	-2	4	5.96	1.09
5	-8	-4	27.12	2.62
3	5	-4	34.27	2.98
-8	3	-4	28.61	2.91
8	-3	4	28.54	3.12
8	-3	4	31.45	3.10
4	4	-4	72.35	6.18
8	-4	4	63.03	6.51
5	3	-4	40.37	4.52
3	-8	-4	49.01	3.91
8	-5	4	45.31	4.20
2	-8	-4	6.30	1.05
6	2	-4	6.54	1.59
7	1	-4	1.13	1.32
1	-8	-4	2.88	0.77
8	-7	4	3.24	0.61
8	0	-4	0.74	1.18
0	-8	-4	1.99	0.82
8	-8	4	3.51	0.62
-1	-8	-4	6.97	1.36
-2	-8	-4	7.96	1.37
9	-9	-4	12.24	2.50
0	-9	4	8.19	0.96
-9	1	-4	4.45	0.81
9	-1	4	3.63	0.70
9	-1	4	5.19	1.05
7	-9	-4	4.73	0.71
2	7	-4	4.26	0.88
-2	-7	4	4.40	0.64
9	-2	4	4.34	1.01
9	-2	4	3.17	0.74
6	-9	-4	3.80	0.65
-9	3	-4	3.95	0.81
3	6	-4	4.23	0.89
9	-3	4	5.07	0.97
9	-3	4	2.72	1.01
5	-9	-4	10.68	1.20
9	-4	4	14.42	1.86
9	-4	4	11.92	2.20
4	-9	-4	11.24	1.33
5	4	-4	15.09	2.54
9	-5	4	15.99	2.03
6	3	-4	6.45	1.72
9	-6	4	10.27	1.72
9	-6	4	9.21	1.77
2	-9	-4	4.30	0.82
7	2	-4	4.96	1.67
9	-8	4	15.86	2.01
0	-9	-4	7.00	1.22
9	0	-4	3.98	1.73
2	8	-4	16.38	2.50
10	-2	4	8.65	1.64
-2	-8	4	12.62	1.26
10	-2	4	7.64	1.76
7	-10	-4	6.56	0.81
3	7	-4	9.93	2.09
10	-3	4	4.19	1.02
10	-3	4	4.54	1.34
4	6	-4	1.93	1.13
10	-4	4	0.32	0.80
10	-4	4	-0.65	1.12
5	5	-4	1.05	1.28
5	-10	-4	1.04	0.40
10	-5	4	-0.37	1.03
10	-5	4	0.18	0.78
4	-10	-4	1.33	0.49
6	4	-4	2.18	1.56
10	-6	4	1.69	0.95
10	-6	4	3.58	1.03
3	-10	-4	7.94	1.16
7	3	-4	7.87	2.32
8	2	-4	8.25	2.48
2	-10	-4	12.79	1.60
0	0	-5	-0.07	0.14
0	0	5	-0.05	0.15
0	0	5	-0.02	0.12
0	0	5	-0.02	0.07
1	-1	-5	18.31	1.58
0	1	-5	16.35	1.61
1	-1	-5	17.93	1.66
-1	0	-5	19.89	1.67
0	-1	5	17.79	1.63
0	-1	5	18.74	1.63
1	0	5	18.68	1.59
1	0	5	16.06	1.62
0	-1	5	19.54	1.64
1	0	5	22.36	1.67
-1	1	-5	18.39	1.60
1	0	-5	17.58	1.58
1	0	-5	17.57	1.62
0	-1	-5	19.13	1.65
1	-1	5	19.13	1.59
-1	0	5	19.37	1.62
1	-1	5	18.10	1.61
-1	0	5	17.45	1.58
1	-1	5	19.08	1.60
0	1	5	15.96	1.57
-1	-1	-5	0.25	0.23
-2	1	5	0.07	0.18
1	-2	5	-0.10	0.14
1	-2	5	0.02	0.14
-2	1	5	0.14	0.20
1	-2	5	0.11	0.11
1	1	5	0.09	0.21
3	-2	-5	16.45	1.50
-2	-1	-5	18.14	1.46
-2	-1	-5	17.96	1.68
3	-2	-5	13.16	1.47
1	-3	5	16.13	1.43
1	-3	5	15.36	1.45
-3	2	5	15.76	1.46
1	-3	5	15.98	1.44
0	2	-5	118.76	11.22
-1	-1	0	77.88	5.69
-1	-1	0	81.46	5.68
1	1	-1	0.09	0.11
1	1	-1	0.17	0.18
1	1	-1	0.00	0.08
1	1	-1	0.04	0.07
-1	-1	1	0.45	0.13
-1	-1	1	0.03	0.07
-1	-1	1	0.03	0.09
1	1	-2	431.22	33.12
1	1	-2	497.77	33.07
1	1	-2	539.20	33.10
1	1	-2	473.47	33.09
1	1	-2	498.23	33.08
-1	-1	2	545.05	33.10
1	1	-3	0.02	0.06
1	1	-3	0.04	0.05
1	1	-3	-0.09	0.14
-1	-1	3	0.08	0.14
1	1	-4	29.81	1.84
1	1	-4	22.97	1.95
-1	-1	4	28.88	1.93
1	1	-5	-0.02	0.38
1	1	-5	0.01	0.06
-1	-1	5	-0.22	0.18
-1	-1	5	0.02	0.05
1	1	-6	100.71	6.96
-1	-1	6	103.15	6.86
1	1	-7	-0.01	0.06
1	1	-7	-0.17	0.51
-1	-1	7	0.01	0.21
1	1	-8	12.60	1.36
1	1	-9	0.25	0.96
1	1	-10	10.25	1.66
-2	0	-10	8.09	1.37
-2	0	-10	4.02	0.90
-2	0	-10	6.25	0.81
-2	0	-9	2.01	0.53
-2	0	-9	2.75	0.91
-2	0	-8	6.95	0.92
-2	0	-8	5.46	0.67
-2	0	-7	18.84	1.57
-2	0	-7	18.03	1.43
-2	0	-6	135.74	8.84
-2	0	-6	127.18	8.90
2	0	5	102.75	6.50
-2	0	-5	91.07	6.57
2	0	4	13.71	0.94
2	0	4	12.49	1.01
-2	0	-4	14.78	1.06
2	0	3	243.48	16.17
2	0	3	248.18	16.22
2	0	3	238.02	16.17
2	0	2	645.84	39.47
2	0	2	678.31	39.48
2	0	2	581.05	39.46
-2	0	-2	520.36	39.44
-2	0	-2	638.41	39.45
-2	0	-2	557.17	39.44
-2	0	-2	535.25	39.45
2	0	1	552.24	39.16
2	0	1	603.91	39.12
2	0	1	639.74	39.16
-2	0	-1	605.57	39.10
-2	0	-1	555.49	39.10
-2	0	-1	572.03	39.15
2	0	0	3.05	0.45
2	0	0	3.00	0.48
2	0	0	3.77	0.56
2	0	0	4.19	0.46
2	0	0	3.85	0.46
-2	0	0	4.23	0.48
-2	0	0	3.43	0.48
2	0	-1	565.84	36.23
2	0	-1	498.96	36.17
2	0	-1	480.42	36.14
2	0	-1	550.37	36.20
2	0	-1	560.38	36.16
-2	0	1	545.73	36.15
-2	0	1	605.81	36.22
2	0	-2	584.46	39.63
2	0	-2	600.82	39.57
2	0	-2	648.61	39.57
2	0	-2	547.32	39.58
2	0	-2	541.27	39.59
-2	0	2	651.45	39.63
2	0	-3	280.23	17.62
2	0	-3	265.64	17.62
2	0	-3	260.90	17.58
2	0	-3	287.21	17.57
-2	0	3	226.70	17.65
2	0	-4	12.89	0.93
2	0	-4	15.10	0.98
2	0	-4	13.96	1.00
2	0	-4	13.16	0.93
2	0	-4	14.14	1.08
-2	0	4	11.42	1.09
2	0	-5	99.44	6.54
2	0	-5	99.89	6.45
2	0	-5	97.53	6.42
-2	0	5	88.70	6.53
2	0	-6	137.29	8.90
-2	0	6	129.26	8.99
2	0	-7	20.23	1.56
2	0	-7	21.87	1.76
2	0	-7	23.02	1.52
-2	0	7	24.02	1.68
2	0	-8	8.95	1.10
-2	0	8	9.73	1.18
2	0	-9	2.26	0.68
2	0	-10	10.40	1.49
-3	1	-10	2.52	0.71
-3	1	-10	3.64	0.78
-3	1	-10	5.77	1.31
-3	1	-9	0.84	0.84
-3	1	-9	0.21	0.62
-3	1	-9	0.46	0.32

2	-2	-5	126.88	11.20
2	-2	-5	123.10	11.26
-2	0	-5	152.03	11.30
-2	2	5	146.22	11.25
0	-2	5	133.94	11.24
2	0	5	124.71	11.20
2	0	5	145.68	11.26
0	-2	5	138.92	11.23
0	-2	5	127.54	11.23
-2	1	-5	0.13	0.18
1	-2	-5	0.30	0.20
1	1	-5	0.07	0.12
-2	1	-5	0.11	0.14
-1	-1	5	-0.06	0.09
2	-1	5	0.05	0.14
2	-1	5	0.41	0.16
2	-1	5	0.13	0.11
-1	2	5	0.02	0.09
-1	-1	5	0.29	0.19
-1	2	5	0.16	0.23
-2	2	-5	133.62	11.23
0	-2	-5	130.51	11.32
-2	2	-5	139.26	11.31
2	0	-5	126.14	11.26
-2	0	5	137.26	11.27
2	-2	5	130.79	11.25
2	-2	5	128.08	11.27
-2	0	5	138.47	11.28
2	-2	5	141.90	11.25
-1	-2	-5	15.39	1.54
3	-1	-5	15.12	1.40
2	-3	5	14.92	1.34
-3	1	5	14.66	1.36
2	-3	5	14.16	1.32
2	-3	5	15.18	1.37
1	2	5	15.02	1.32
-2	-2	-5	0.21	0.42
4	-2	-5	0.10	0.32
-4	2	5	0.46	0.31
2	2	5	0.35	0.24
2	-4	5	-0.05	0.14
2	-4	5	0.21	0.16
2	-4	5	-0.06	0.16
-4	2	5	0.00	0.26
5	-3	-5	1.31	0.59
-3	-2	-5	0.24	0.66
-3	-2	-5	0.78	0.30
2	-5	5	0.57	0.19
2	-5	5	0.70	0.20
3	2	5	0.61	0.19
3	2	5	1.09	0.35
4	-3	-5	10.16	1.39
4	-3	-5	11.65	1.12
-3	-1	-5	9.66	1.16
-3	-1	-5	14.83	1.59
1	-4	5	12.04	1.07
1	-4	5	11.96	1.06
3	1	5	9.31	1.05
1	-4	5	11.73	1.06
3	1	5	10.38	1.10
3	-3	-5	1.08	0.29
-3	0	-5	0.39	0.27
0	3	-5	0.80	0.21
3	-3	-5	1.30	0.31
3	0	5	0.80	0.22
3	0	5	0.88	0.24
0	-3	5	0.93	0.20
0	-3	5	1.06	0.20
0	-3	5	0.90	0.26
2	-3	-5	14.05	1.40
1	2	-5	17.25	1.38
-3	1	-5	14.45	1.45
2	-3	-5	15.68	1.29
3	-1	5	13.68	1.38
-1	-2	5	15.59	1.35
-1	-2	5	13.56	1.37
-3	2	-5	15.07	1.40
1	-3	-5	13.23	1.37
2	1	-5	13.56	1.31
-2	-1	5	14.25	1.31
3	-2	5	13.48	1.32
-2	-1	5	15.19	1.29
3	-2	5	14.30	1.26
3	0	-5	0.76	0.25
0	-3	-5	0.87	0.32
-3	3	-5	0.67	0.25
-3	0	5	1.01	0.24
3	-3	5	1.22	0.22
3	-3	5	0.76	0.21
3	-3	5	0.98	0.21
0	3	5	1.67	0.44
-3	0	5	0.87	0.32
4	-1	-5	11.60	1.37
-1	-3	-5	12.27	1.44
3	-4	5	12.75	1.14
3	-4	5	10.93	1.04
5	-2	-5	0.96	0.57
-2	-3	-5	1.45	0.64
-2	-3	-5	0.68	0.21
3	-5	5	0.89	0.24
3	-5	5	0.70	0.16
-3	-3	-5	0.05	0.25
-3	-3	-5	-0.79	0.73
6	-3	-5	0.26	0.79
3	3	5	-0.03	0.21
3	-6	5	0.06	0.19
3	-6	5	0.09	0.14
-4	-3	-5	0.64	0.27
7	-4	-5	-0.04	0.87
-4	-3	-5	-0.10	0.75
3	-7	5	1.09	0.21
4	3	5	0.98	0.27
3	-7	5	1.18	0.27
2	-6	5	22.78	1.95
2	-6	5	19.86	1.91
4	2	5	23.07	2.13
5	-4	-5	-0.05	0.60

-3	1	-8	10.66	1.13
-3	1	-7	1.20	0.54
-3	1	-7	1.78	0.54
3	-1	6	31.21	2.56
-3	1	-6	41.46	2.66
-3	1	-6	37.90	2.69
3	-1	5	13.01	1.02
-3	1	-5	11.20	1.04
3	-1	4	142.96	9.54
3	-1	4	136.57	9.57
3	-1	4	129.19	9.62
-3	1	-4	166.23	9.68
-3	1	-4	143.48	9.61
3	-1	3	36.74	2.55
3	-1	3	33.87	2.55
3	-1	3	38.19	2.54
3	-1	3	37.48	2.54
-3	1	-3	37.79	2.57
-3	1	-3	35.86	2.66
-3	1	-3	42.35	2.53
3	-1	2	112.95	7.33
3	-1	2	107.44	7.36
3	-1	2	112.01	7.32
-3	1	-2	113.28	7.33
-3	1	-2	113.12	7.30
-3	1	-2	97.08	7.38
3	-1	1	45.56	3.23
3	-1	1	47.42	3.19
-3	1	-1	43.85	3.21
-3	1	-1	44.63	3.17
-3	1	-1	50.64	3.22
3	-1	0	268.56	17.19
3	-1	0	279.08	17.22
3	-1	0	253.68	17.18
3	-1	0	246.77	17.24
-3	1	0	243.38	17.21
-3	1	0	252.75	17.18
-3	1	0	259.99	17.19
3	-1	-1	40.49	3.18
3	-1	-1	48.74	3.13
3	-1	-1	46.83	3.21
3	-1	-1	41.54	3.09
3	-1	-1	48.22	3.11
3	-1	-1	47.33	3.10
-3	1	1	44.88	3.09
-3	1	1	44.61	3.09
3	-1	-2	122.78	7.78
3	-1	-2	112.94	7.86
3	-1	-2	108.73	7.79
3	-1	-2	100.32	7.79
3	-1	-2	112.00	7.81
-3	1	2	125.96	7.75
-3	1	2	130.48	7.89
3	-1	-3	37.23	2.49
3	-1	-3	29.44	2.48
3	-1	-3	39.72	2.52
3	-1	-3	36.06	2.53
-3	1	3	37.86	2.60
3	-1	-4	143.89	10.24
3	-1	-4	142.99	10.20
3	-1	-4	168.06	10.24
3	-1	-4	166.30	10.25
3	-1	-4	146.88	10.26
-3	1	4	163.48	10.36
-3	1	4	139.95	10.33
3	-1	-5	10.86	0.81
3	-1	-5	10.39	0.98
3	-1	-5	10.10	0.88
-3	1	5	13.34	1.15
-3	1	5	10.62	1.15
3	-1	-6	45.06	2.93
3	-1	-6	43.13	3.00
3	-1	-6	39.72	2.84
-3	1	6	37.79	3.03
-3	1	6	44.24	3.12
3	-1	-7	2.62	0.52
3	-1	-7	2.25	0.32
-3	1	7	1.03	0.42
-3	1	7	2.14	0.61
3	-1	-8	20.35	1.78
3	-1	-8	19.58	1.48
-3	1	8	25.18	1.96
-3	1	8	20.03	1.66
3	-1	-9	1.67	0.81
3	-1	-10	4.69	1.20
-4	2	-10	1.77	1.30
-4	2	-9	0.83	0.91
-4	2	-9	0.27	0.56
-4	2	-8	40.15	3.12
-4	2	-8	40.74	3.06
-4	2	-7	-0.53	0.47
-4	2	-7	-0.33	0.49
4	-2	6	0.85	0.23
4	-2	6	1.36	0.36
4	-2	6	1.24	0.22
-4	2	-6	1.41	0.45
-4	2	-6	0.42	0.49
4	-2	5	-0.06	0.27
4	-2	5	-0.07	0.20
-4	2	-5	0.01	0.42
-4	2	-5	0.04	0.31
4	-2	4	373.29	24.53
4	-2	4	388.86	24.58
-4	2	-4	396.05	24.55
-4	2	-4	330.73	24.49
-4	2	-4	355.03	24.64
4	-2	3	0.13	0.29
4	-2	3	-0.14	0.20
4	-2	3	0.04	0.17
-4	2	-3	0.12	0.14
-4	2	-3	0.19	0.31
-4	2	-3	0.27	0.12
-4	2	-3	0.02	0.18
4	-2	2	46.05	2.96
4	-2	2	39.00	2.92
-4	2	-2	41.14	3.01
-4	2	-2	39.40	2.90

-4	-1	-5	0.81	0.35
-4	-1	-5	0.64	0.69
4	1	5	0.27	0.21
1	-5	5	0.51	0.19
1	-5	5	0.76	0.26
4	1	5	0.13	0.29
4	-4	-5	53.88	4.32
0	4	-5	51.03	4.31
0	-4	5	51.51	4.27
0	-4	5	41.52	4.29
4	0	5	48.64	4.31
4	0	5	54.02	4.35
1	3	-5	10.93	1.07
3	-4	-5	11.58	1.08
3	-4	-5	11.02	1.15
-4	1	-5	9.05	1.26
4	-1	5	10.88	1.10
-1	-3	5	12.65	1.05
4	-1	5	10.53	1.05
-3	4	5	10.67	0.95
-1	-3	5	9.15	1.06
-4	2	-5	-0.20	0.29
-4	2	-5	0.47	0.25
2	2	-5	-0.02	0.18
-2	-2	5	-0.25	0.23
-2	-2	5	-0.07	0.13
4	-2	5	0.20	0.19
3	1	-5	11.73	1.21
1	-4	-5	10.13	1.19
-4	3	-5	9.71	1.34
-4	3	-5	13.58	1.03
4	-3	5	10.70	1.15
-3	-1	5	12.33	1.13
0	-4	-5	52.97	4.97
4	0	-5	56.48	5.00
4	-4	5	59.20	4.87
4	-4	5	56.80	4.85
-1	-4	-5	0.33	0.61
5	-1	-5	0.16	0.54
4	-5	5	0.65	0.25
-2	-4	-5	19.47	1.89
2	4	5	23.91	1.91
-3	-4	-5	0.37	0.77
-3	-4	-5	1.27	0.30
7	-3	-5	0.78	1.03
3	4	5	1.03	0.26
4	-7	5	1.64	0.34
8	-4	-5	-0.77	1.11
-4	-4	-5	0.50	0.68
4	-8	5	-0.03	0.23
4	4	5	-0.28	0.27
9	-5	-5	-0.74	1.31
-5	-4	-5	0.58	0.79
4	-9	5	0.07	0.25
5	4	5	-0.52	0.43
-5	-3	-5	-0.31	0.63
8	-5	-5	-0.05	1.16
5	3	5	1.27	0.35
-5	-2	-5	-0.31	0.97
7	-5	-5	-0.81	0.87
2	-7	5	0.25	0.18
5	2	5	0.42	0.26
6	-5	-5	3.28	0.97
-5	-1	-5	4.73	0.91
5	1	5	5.39	0.71
5	1	5	5.12	0.91
5	-5	-5	1.00	0.29
5	-5	-5	0.24	0.69
-5	0	-5	1.02	0.74
-5	0	-5	0.77	0.51
0	5	-5	0.89	0.29
-5	5	5	1.05	0.21
5	0	5	0.54	0.28
5	0	5	0.83	0.33
0	-5	5	0.75	0.19
-5	1	-5	1.23	0.52
4	-5	-5	0.65	0.25
4	-5	-5	0.55	0.56
1	4	-5	0.43	0.23
-5	1	-5	0.59	0.69
5	-1	5	0.45	0.22
-1	-4	5	0.36	0.16
5	-1	5	0.00	0.24
3	-5	-5	0.77	0.38
-5	2	-5	1.61	0.44
2	3	-5	0.67	0.25
-5	2	-5	2.21	0.89
3	-5	5	0.71	0.28
5	-2	5	0.42	0.28
5	-2	5	1.20	0.28
2	-5	-5	0.79	0.35
2	-5	-5	1.51	0.18
-5	3	-5	1.62	0.38
5	-3	5	1.07	0.33
1	-5	-5	0.40	0.40
4	1	-5	0.74	0.38
5	-4	5	0.31	0.24
0	-5	-5	0.39	0.63
5	0	-5	1.21	0.60
5	-5	5	0.67	0.27
6	-1	-5	5.61	1.24
5	-6	5	5.96	1.18
-2	-5	-5	0.11	0.80
7	-2	-5	-0.79	1.60
5	-7	5	0.35	0.28
-3	-5	-5	0.46	0.77
8	-3	-5	1.21	1.33
5	-8	5	2.22	0.40
3	5	5	2.23	0.40
9	-4	-5	-0.54	1.28
-4	-5	-5	-0.48	0.74
5	-9	5	0.19	0.26
-5	-5	-5	0.91	1.03
10	-5	-5	-1.24	1.57
5	-10	5	-0.03	0.32
10	-6	-5	-1.08	1.82
-6	-4	-5	-0.93	0.99

-4	2	-2	44.96	2.87
-4	2	-2	44.35	2.91
-4	2	-2	43.38	2.94
4	-2	1	-0.04	0.33
-4	2	-1	0.95	0.28
4	-2	0	913.02	63.24
4	-2	0	886.36	63.32
4	-2	0	969.68	63.30
-4	2	0	1000.00	63.24
-4	2	0	996.37	63.29
-4	2	0	959.47	63.42
-4	2	0	938.48	63.34
4	-2	-1	0.14	0.19
4	-2	-1	0.11	0.16
4	-2	-1	-0.01	0.33
4	-2	-1	0.07	0.18
-4	2	1	0.03	0.10
-4	2	1	-0.17	0.24
-4	2	1	-0.07	0.17
4	-2	-2	35.23	2.69
4	-2	-2	40.72	2.65
-4	2	2	35.61	2.79
-4	2	2	42.22	2.64
4	-2	-3	0.14	0.12
4	-2	-3	0.09	0.35
4	-2	-3	-0.06	0.20
4	-2	-3	-0.10	0.20
-4	2	3	0.40	0.20
-4	2	3	-0.24	0.39
4	-2	-4	383.47	25.38
4	-2	-4	373.58	25.45
4	-2	-4	387.12	25.35
4	-2	-4	380.07	25.40
-4	2	4	382.03	25.55
4	-2	-5	0.59	0.26
4	-2	-5	0.40	0.18
4	-2	-5	-0.04	0.32
4	-2	-5	0.15	0.26
-4	2	5	0.32	0.46
-4	2	5	-0.14	0.52
4	-2	-6	0.57	0.38
4	-2	-6	1.12	0.22
4	-2	-6	1.22	0.30
-4	2	6	1.35	0.64
-4	2	6	1.08	0.50
4	-2	-7	0.20	0.17
4	-2	-7	0.44	0.38
-4	2	7	-0.70	0.60
-4	2	7	-0.28	0.44
4	-2	-8	41.16	3.03
4	-2	-8	41.11	2.81
-4	2	8	42.64	3.04
-4	2	8	36.44	3.94
4	-2	-9	0.27	0.77
4	-2	-9	0.31	0.30
4	-2	-10	2.15	1.11
-5	3	-9	-0.50	0.51
-5	3	-8	14.43	1.48
5	-3	7	0.00	0.36
-5	3	-7	0.42	0.50
5	-3	6	16.06	1.43
5	-3	6	17.17	1.42
-5	3	-6	19.24	1.62
-5	3	-6	19.78	1.76
5	-3	5	0.74	0.31
5	-3	5	1.19	0.46
-5	3	-5	0.96	0.47
-5	3	-5	0.83	0.40
5	-3	4	92.07	6.58
-5	3	-4	98.72	6.55
-5	3	-4	101.02	6.68
5	-3	3	2.59	0.39
5	-3	3	3.15	0.46
-5	3	-3	3.47	0.45
-5	3	-3	3.66	0.46
-5	3	-3	2.99	0.51
-5	3	-3	3.80	0.46
5	-3	2	52.98	3.77
-5	3	-2	55.18	3.79
-5	3	-2	56.94	3.83
5	-3	1	5.88	0.56
5	-3	1	4.63	0.68
5	-3	1	4.64	0.70
-5	3	-1	5.22	0.60
-5	3	-1	5.30	0.67
-5	3	-1	5.50	0.66
5	-3	0	155.04	11.94
5	-3	0	190.67	11.90
5	-3	0	181.66	11.90
5	-3	0	182.64	12.01
-5	3	0	186.26	11.96
-5	3	0	177.92	11.90
-5	3	0	169.03	11.97
-5	3	0	178.23	11.92
5	-3	-1	5.78	0.57
5	-3	-1	5.64	0.88
5	-3	-1	5.29	0.50
5	-3	-1	4.85	0.63
5	-3	-1	5.13	0.54
-5	3	1	5.55	0.73
-5	3	1	5.21	0.56
5	-3	-2	57.24	3.78
5	-3	-2	52.73	3.79
-5	3	2	56.44	3.95
5	-3	-3	1.72	0.60
5	-3	-3	2.14	0.32
5	-3	-3	3.32	0.45
5	-3	-3	3.61	0.47
-5	3	3	3.04	0.62
-5	3	3	4.35	0.57
5	-3	-4	89.36	5.82
5	-3	-4	90.12	5.87
5	-3	-4	80.44	6.00
5	-3	-4	90.86	5.84
-5	3	4	79.51	6.03
-5	3	4	87.51	5.81
5	-3	-5	1.03	0.23

4	-10	5	0.85	0.34
9	-6	-5	-0.82	1.35
-6	-3	-5	-0.35	0.72
3	-9	5	-0.02	0.22
6	3	5	-0.38	0.38
8	-6	-5	1.39	1.58
6	2	5	2.99	0.53
7	-6	-5	0.18	1.00
-6	-1	-5	0.32	0.64
1	-7	5	0.84	0.21
6	1	5	1.16	0.47
6	1	5	0.63	0.27
-6	0	-5	-0.04	0.67
6	-6	-5	-0.97	0.83
0	6	-5	0.19	0.27
6	-6	-5	0.25	0.32
6	0	5	-0.21	0.34
6	0	5	-0.21	0.22
0	-6	5	0.21	0.16
-6	1	-5	8.82	1.48
5	-6	-5	4.89	0.77
1	5	-5	7.40	0.89
6	-1	5	5.59	1.03
6	-1	5	6.15	0.87
-5	6	5	6.62	0.63
2	4	-5	21.84	1.98
4	-6	-5	18.02	1.87
6	-2	5	21.94	1.96
6	-2	5	19.95	2.05
-4	6	5	20.23	1.76
3	-6	-5	0.05	0.11
3	-6	-5	-0.18	0.27
3	-6	-5	0.19	0.52
3	3	-5	0.06	0.32
-6	3	-5	0.78	0.32
6	-3	5	0.22	0.36
6	-3	5	0.18	0.26
2	-6	-5	28.67	2.30
4	2	-5	22.45	2.67
6	-4	5	29.23	2.53
1	-6	-5	6.51	1.19
5	1	-5	5.37	1.11
6	-5	5	8.09	0.94
0	-6	-5	-0.54	0.62
0	-6	-5	0.61	0.17
6	-6	5	0.31	0.31
7	-1	-5	0.79	1.17
-1	-6	-5	1.13	0.83
6	-7	5	1.11	0.36
8	-2	-5	2.58	1.35
-2	-6	-5	3.05	1.06
-3	-6	-5	-0.68	0.84
9	-3	-5	-0.27	1.21
6	-9	5	0.43	0.34
-4	-6	-5	-0.42	0.93
10	-4	-5	-0.97	1.68
6	-10	5	1.05	0.42
-7	-2	-5	-0.74	0.67
9	-7	-5	-1.07	1.57
2	-9	5	0.26	0.23
7	2	5	-1.06	0.69
-7	-1	-5	-0.22	0.64
8	-7	-5	0.24	1.56
7	1	5	0.05	0.56
7	1	5	-0.12	0.40
1	-8	5	0.23	0.18
7	-7	-5	-0.88	1.19
0	7	-5	0.95	0.39
-7	0	-5	-0.23	0.64
7	0	5	0.12	0.32
7	0	5	0.12	0.47
0	-7	5	0.29	0.19
-7	1	-5	1.66	0.72
6	-7	-5	1.12	0.33
6	-7	-5	-0.87	0.93
1	6	-5	1.37	0.41
-1	-6	5	0.59	0.20
7	-1	5	0.62	0.52
7	-1	5	0.59	0.38
2	5	-5	0.26	0.33
-7	2	-5	1.81	0.81
5	-7	-5	-1.02	0.79
7	-2	5	-0.34	0.48
7	-2	5	0.18	0.36
4	-7	-5	0.16	0.62
3	4	-5	1.57	0.45
7	-3	5	1.16	0.44
7	-3	5	0.60	0.50
4	3	-5	1.30	0.55
-7	4	-5	2.49	0.36
3	-7	-5	1.96	0.29
3	-7	-5	1.24	0.42
7	-4	5	2.22	0.73
7	-4	5	1.06	0.46
5	2	-5	0.40	0.71
2	-7	-5	0.41	0.41
7	-5	5	0.65	0.41
7	-5	5	0.84	0.37
6	1	-5	0.47	1.01
1	-7	-5	1.38	0.70
7	-6	5	1.60	0.41
7	0	-5	0.40	1.18
0	-7	-5	1.02	0.79
7	-7	5	0.43	0.40
-1	-7	-5	-0.75	0.82
8	-1	-5	-1.19	1.21
7	-8	5	-0.21	0.32
-2	-7	-5	-0.58	0.93
9	-2	-5	-1.90	1.33
7	-9	5	0.14	0.40
-8	-1	-5	0.46	0.80
9	-8	-5	-2.67	1.88
8	1	5	0.54	0.52
1	-9	5	0.65	0.29
8	-8	-5	-2.62	1.51
-8	0	-5	0.94	0.65
8	0	5	0.52	0.45
5	-3	-5	1.05	0.36
5	-3	-5	1.03	0.39
5	-3	-5	0.71	0.29
-5	3	5	0.43	0.60
-5	3	5	1.10	0.27
5	-3	-6	17.58	1.31
5	-3	-6	18.27	1.45
5	-3	-6	18.07	1.40
-5	3	6	17.18	1.75
5	-3	-7	0.61	0.30
5	-3	-7	0.03	0.37
5	-3	-7	0.32	0.20
-5	3	7	-0.02	0.50
-5	3	7	-0.39	0.67
5	-3	-8	13.23	1.41
5	-3	-8	14.24	1.08
5	-3	-9	0.03	0.31
5	-3	-9	-0.48	0.77
-6	4	-9	-0.18	0.58
6	-4	8	4.59	0.81
-6	4	-8	3.27	0.70
6	-4	7	5.55	0.82
-6	4	-7	3.90	0.73
6	-4	6	41.16	2.93
-6	4	-6	40.38	3.04
6	-4	5	18.32	1.46
-6	4	-5	18.85	1.61
6	-4	4	8.58	0.82
-6	4	-4	7.58	1.00
6	-4	3	52.02	3.60
-6	4	-3	52.18	3.67
-6	4	-3	51.19	3.59
6	-4	2	170.98	11.86
-6	4	-2	179.52	11.91
-6	4	-2	179.69	11.86
-6	4	-2	177.11	11.90
6	-4	1	98.29	6.66
6	-4	1	97.95	6.56
6	-4	1	94.13	6.70
-6	4	-1	90.96	6.58
-6	4	-1	102.02	6.64
-6	4	-1	97.71	6.59
6	-4	0	7.95	1.00
6	-4	0	6.59	0.68
6	-4	0	6.66	0.84
-6	4	0	7.43	1.03
-6	4	0	7.25	0.92
-6	4	0	7.58	0.87
6	-4	-1	98.17	6.38
6	-4	-1	91.27	6.47
6	-4	-1	91.93	6.47
6	-4	-1	94.45	6.45
6	-4	-1	96.60	6.67
-6	4	1	99.16	6.55
6	-4	-2	168.01	11.55
6	-4	-2	181.77	11.57
-6	4	2	179.81	11.71
-6	4	2	177.79	11.72
-6	4	2	155.29	11.59
6	-4	-3	49.48	3.35
6	-4	-3	51.57	3.29
6	-4	-3	50.14	3.32
-6	4	3	40.43	3.39
-6	4	3	48.85	3.56
6	-4	-4	7.85	0.76
6	-4	-4	8.73	0.80
6	-4	-4	8.28	0.73
-6	4	4	8.13	1.27
-6	4	4	7.49	0.94
6	-4	-5	18.68	1.49
6	-4	-5	24.17	1.49
6	-4	-5	15.85	1.67
6	-4	-5	19.06	1.43
-6	4	5	18.36	1.85
6	-4	-6	39.09	2.72
6	-4	-6	39.21	2.76
6	-4	-6	35.51	2.89
-6	4	6	43.28	3.17
6	-4	-7	4.81	0.67
6	-4	-7	4.87	0.60
-6	4	7	3.94	0.93
-6	4	7	3.39	0.77
6	-4	-8	2.77	0.72
6	-4	-9	0.69	0.84
7	-5	8	10.25	1.40
7	-5	7	-0.44	0.86
7	-5	6	12.59	1.17
7	-5	5	0.94	0.92
7	-5	5	0.69	0.34
7	-5	4	20.86	1.49
-7	5	-4	17.05	1.37
7	-5	3	0.30	0.66
7	-5	3	0.62	0.30
-7	5	-3	0.34	0.31
7	-5	2	43.62	3.23
7	-5	2	43.52	3.14
7	-5	2	51.39	3.50
-7	5	-2	42.39	3.21
-7	5	-2	45.27	3.19
7	-5	1	2.74	0.81
7	-5	1	0.52	0.51
7	-5	1	0.53	0.29
-7	5	-1	0.77	0.45
-7	5	-1	0.37	0.38
7	-5	0	42.03	3.20
7	-5	0	40.86	3.05
7	-5	0	47.33	3.31
-7	5	0	44.72	3.17
7	-5	-1	0.88	0.30
7	-5	-1	0.91	0.46
7	-5	-1	0.71	0.65
-7	5	1	0.97	0.47
7	-5	-2	40.63	2.96
-7	5	2	40.85	3.05
-7	5	2	45.60	3.22
7	-5	-3	1.75	0.71
7	-5	-3	0.37	0.27
8	0	5	0.91	0.76
7	-8	-5	-0.19	0.36
7	-8	-5	-1.31	1.21
1	7	-5	0.35	0.44
-8	1	-5	-0.19	0.66
8	-1	5	0.00	0.56
-1	-7	5	0.12	0.22
6	-8	-5	4.56	0.70
6	-8	-5	2.21	1.22
2	6	-5	6.47	1.17
-8	2	-5	4.55	1.03
-2	-6	5	5.53	0.68
8	-2	5	3.63	0.90
5	-8	-5	2.08	0.46
3	5	-5	2.36	0.62
8	-3	5	1.69	0.72
8	-3	5	1.50	0.63
4	-8	-5	-0.20	0.34
4	4	-5	0.60	0.62
4	-8	-5	-0.39	0.60
8	-4	5	-0.71	0.59
3	-8	-5	2.04	0.56
5	3	-5	1.87	0.86
3	-8	-5	2.60	0.64
8	-5	5	2.97	0.68
8	-5	5	3.90	0.76
8	-6	5	9.52	1.48
7	1	-5	-1.06	1.30
1	-8	-5	0.23	0.71
8	-7	5	0.14	0.44
0	-8	-5	1.32	0.89
8	0	-5	1.56	1.51
8	-8	5	2.13	0.55
9	-1	-5	-2.07	1.57
-1	-8	-5	-0.08	1.01
1	8	-5	0.69	0.70
-9	1	-5	0.13	0.73
8	-9	-5	-1.99	1.64
-1	-8	5	0.30	0.30
9	-1	5	0.50	0.50
9	-1	5	1.51	0.76
2	7	-5	-0.01	0.63
-9	2	-5	-0.17	0.89
7	-9	-5	-0.01	0.36
9	-2	5	-0.50	0.78
-2	-7	5	-0.10	0.26
9	-2	5	-0.06	0.63
3	6	-5	0.38	0.62
6	-9	-5	-0.46	0.32
6	-9	-5	0.30	1.07
9	-3	5	-0.50	0.59
9	-3	5	-0.11	0.82
4	5	-5	-0.17	0.83
5	-9	-5	-0.52	0.88
5	-9	-5	-0.19	0.36
9	-4	5	-0.42	0.77
9	-4	5	-0.84	0.62
4	-9	-5	-0.28	0.43
5	4	-5	0.06	1.00
9	-5	5	-0.58	0.61
9	-5	5	-0.04	0.79
3	-9	-5	0.04	0.47
6	3	-5	-0.94	1.27
9	-6	5	0.36	0.80
9	-6	5	-0.26	0.65
2	-9	-5	-0.02	0.62
7	2	-5	-0.26	1.74
9	-7	5	-0.41	0.60
9	-7	5	0.92	0.66
1	-9	-5	0.54	0.87
8	1	-5	0.06	2.08
9	-8	5	0.97	0.66
9	-8	5	1.93	0.67
4	6	-5	0.96	1.37
6	-10	-5	0.55	0.41
10	-4	5	-0.43	1.12
10	-4	5	0.62	0.82
5	5	-5	-1.08	1.52
5	-10	-5	0.01	0.44
10	-5	5	-0.16	1.09
4	-10	-5	0.01	0.56
6	4	-5	2.33	2.05
10	-6	5	0.22	1.00
10	-6	5	0.55	1.35
0	0	-6	4.49	0.57
0	0	6	4.08	0.45
0	0	6	4.19	0.57
0	0	6	4.27	0.52
1	-1	-6	58.86	5.45
-1	0	-6	66.37	5.54
1	-1	-6	59.19	5.53
0	-1	6	63.09	5.48
-1	1	6	71.09	5.51
0	-1	6	62.36	5.51
-1	1	6	69.35	5.54
1	0	6	62.22	5.46
0	-1	6	64.30	5.51
1	0	6	74.37	5.55
-1	1	6	62.84	5.49
1	0	-6	59.24	5.20
-1	1	-6	62.09	5.23
0	-1	-6	62.91	5.29
0	1	6	54.32	5.20
0	1	6	60.14	5.18
1	-1	6	61.70	5.24
-1	0	6	58.00	5.22
1	-1	6	66.66	5.22
-1	0	6	64.52	5.26
-1	0	6	59.90	5.23
1	-1	6	68.81	5.26
2	-1	-6	139.30	12.59
-1	-1	-6	162.38	12.66
2	-1	-6	142.01	12.53
1	-2	6	159.58	12.56
1	-2	6	139.94	12.55
1	-2	6	153.58	12.58
-2	-1	-6	63.85	4.95
-7	5	3	0.02	0.49
-7	5	3	1.07	0.63
7	-5	-4	21.53	1.63
7	-5	-4	23.47	1.59
-7	5	4	17.23	1.81
-7	5	4	22.75	2.02
7	-5	-5	0.42	0.46
7	-5	-5	0.37	0.30
7	-5	-5	1.08	0.36
-7	5	5	-0.60	0.54
-7	5	5	0.38	0.73
7	-5	-6	13.51	1.19
-7	5	6	15.44	1.46
-7	5	6	14.70	1.75
7	-5	-7	0.41	0.41
-7	5	7	0.20	0.80
7	-5	-8	4.97	0.86
8	-6	7	1.63	1.29
8	-6	6	12.05	1.20
8	-6	5	5.24	0.78
8	-6	4	4.57	0.60
-8	6	-4	4.99	0.80
8	-6	3	12.53	1.16
8	-6	3	18.86	1.97
-8	6	-3	13.40	1.26
8	-6	2	47.70	3.26
8	-6	2	43.77	3.34
-8	6	-2	46.65	3.34
8	-6	1	23.48	2.16
8	-6	1	24.07	1.86
8	-6	1	24.58	2.07
-8	6	-1	27.27	2.02
8	-6	0	8.64	1.25
8	-6	0	7.31	0.81
8	-6	0	8.08	0.97
-8	6	0	7.20	1.01
8	-6	-1	22.73	1.75
8	-6	-1	25.07	2.13
-8	6	1	22.64	1.90
8	-6	-2	44.84	3.22
-8	6	2	46.82	3.36
8	-6	-3	13.75	1.18
-8	6	3	14.25	1.46
8	-6	-4	5.10	0.69
-8	6	4	4.65	0.86
-8	6	4	4.48	0.99
8	-6	-5	4.57	0.66
-8	6	5	4.54	1.04
-8	6	5	4.19	0.85
-8	6	6	10.20	1.20
-8	6	7	0.53	0.75
9	-7	6	5.41	0.76
9	-7	5	0.47	0.40
9	-7	5	0.24	1.10
9	-7	4	6.04	1.50
9	-7	4	4.04	0.58
9	-7	3	0.19	0.34
9	-7	3	0.81	0.52
9	-7	3	-0.91	1.15
-9	7	-3	0.52	0.39
9	-7	2	18.83	1.57
9	-7	2	18.34	1.75
9	-7	2	22.67	2.15
9	-7	1	-0.59	0.59
9	-7	1	0.03	0.53
9	-7	1	0.73	0.35
-9	7	-1	0.30	0.48
9	-7	0	9.60	1.31
9	-7	0	7.95	0.86
-9	7	0	7.97	1.08
9	-7	-1	-0.24	0.62
9	-7	-1	0.48	0.32
-9	7	1	0.33	0.56
9	-7	-2	17.41	1.45
-9	7	2	19.15	1.85
9	-7	-3	0.21	0.30
9	-7	-3	0.10	0.80
-9	7	3	-0.60	0.52
9	-7	-4	4.77	0.71
-9	7	4	4.68	1.01
9	-7	-5	0.07	0.33
-9	7	5	-1.24	0.79
-9	7	6	5.84	1.16
10	-8	4	12.53	1.27
10	-8	4	20.90	2.92
10	-8	3	0.27	0.40
10	-8	3	0.55	0.57
10	-8	3	-1.60	1.18
10	-8	2	-1.24	0.95
10	-8	2	0.12	0.35
10	-8	2	0.26	0.57
10	-8	1	0.33	0.62
10	-8	1	0.05	0.37
10	-8	0	29.21	2.40
10	-8	0	24.41	2.02
-10	8	0	26.54	2.27
10	-8	-1	0.42	0.37
10	-8	-1	0.12	0.71
-10	8	1	0.42	0.74
10	-8	-2	0.62	0.37
10	-8	-2	1.14	0.78
-10	8	2	0.16	0.84
10	-8	-3	-0.11	0.35
-10	8	3	-1.31	0.91
10	-8	-4	12.69	1.19
-10	8	4	11.95	1.61
-8	11	2	2.10	0.72
8	-11	-2	1.74	0.44
-8	11	1	0.08	0.70
8	-11	-1	0.35	0.36
8	-11	0	8.51	0.94
8	-11	1	0.35	0.36
8	-11	2	2.50	0.97
8	-11	2	1.37	0.42
-7	10	5	0.93	0.53
7	-10	-5	0.82	0.34
-7	10	4	7.50	1.08

3	-2	-6	56.20	4.77
3	-2	-6	49.55	4.82
1	-3	6	54.78	4.77
1	-3	6	61.47	4.79
2	1	6	50.18	4.75
1	-3	6	57.20	4.79
2	-2	-6	178.40	15.58
-2	0	-6	210.68	15.71
0	2	-6	157.88	15.61
2	-2	-6	170.17	15.66
0	-2	6	187.88	15.65
2	0	6	191.88	15.59
0	-2	6	180.92	15.63
-2	2	6	188.31	15.60
2	0	6	201.47	15.66
0	-2	6	195.09	15.63
-2	1	-6	161.89	12.27
1	-2	-6	144.76	12.31
1	1	-6	138.17	12.24
-1	-1	6	153.03	12.29
2	-1	6	127.31	12.25
-1	-1	6	135.52	12.25
-1	-1	6	148.29	12.25
2	-1	6	161.47	12.27
-1	2	6	132.20	12.21
2	-1	6	155.81	12.24
2	0	-6	167.79	15.14
-2	2	-6	188.75	15.13
0	-2	-6	185.81	15.20
2	-2	6	185.91	15.13
2	-2	6	177.38	15.12
2	-2	6	175.75	15.18
-2	0	6	178.03	15.17
-1	-2	-6	58.81	4.93
3	-1	-6	53.49	4.77
2	-3	6	53.04	4.74
1	2	6	52.14	4.71
2	-3	6	59.27	4.80
2	-3	6	59.12	4.77
4	-2	-6	1.23	0.34
-2	-2	-6	1.78	0.61
2	2	6	1.12	0.27
-4	2	6	1.76	0.37
-4	2	6	1.89	0.37
2	-4	6	1.27	0.23
2	-4	6	0.78	0.26
-3	-2	-6	22.09	2.59
5	-3	-6	20.16	2.33
-3	-2	-6	26.67	2.09
2	-5	6	24.62	2.08
3	2	6	20.75	2.09
2	-5	6	22.13	2.01
-3	-1	-6	34.53	3.06
4	-3	-6	29.83	2.72
1	-4	6	29.77	2.65
-4	3	6	33.27	2.64
1	-4	6	29.94	2.64
3	1	6	25.87	2.64
3	-3	-6	85.28	7.56
-3	0	-6	102.46	7.60
0	3	-6	79.38	7.51
3	-3	-6	89.69	7.48
0	-3	6	89.73	7.53
3	0	6	79.88	7.50
0	-3	6	86.59	7.50
0	-3	6	97.27	7.52
2	-3	-6	52.81	4.74
1	2	-6	52.09	4.70
-3	1	-6	60.96	4.79
3	-1	6	53.85	4.68
-1	-2	6	62.11	4.70
-1	-2	6	52.10	4.68
-1	-2	6	52.75	4.73
3	-1	6	53.10	4.70
-2	3	6	55.15	4.64
1	-3	-6	52.85	4.86
-3	2	-6	62.51	4.76
2	1	-6	52.98	4.79
-3	2	-6	59.42	4.88
-2	-1	6	58.70	4.79
3	-2	6	51.83	4.79
3	-2	6	56.14	4.78
3	-2	6	53.72	4.75
3	0	-6	87.89	7.48
0	-3	-6	86.10	7.57
3	-3	6	88.54	7.47
3	-3	6	87.05	7.41
3	-3	6	90.05	7.46
4	-1	-6	28.51	2.67
-1	-3	-6	29.80	2.82
3	-4	6	27.13	2.49
3	-4	6	31.28	2.59
5	-2	-6	20.78	2.31
-2	-3	-6	20.92	2.47
3	-5	6	20.49	1.91
3	-5	6	26.02	2.05
-3	-3	-6	58.81	4.91
3	-6	6	56.07	4.95
4	3	6	12.77	1.38
3	-7	6	17.54	1.46
4	2	6	31.09	2.69
5	-4	-6	37.68	4.00
-4	-1	-6	50.11	4.35
-4	-1	-6	40.26	3.93
1	-5	6	49.58	3.84
4	1	6	45.35	3.88
0	4	-6	76.45	6.57
-4	0	-6	72.05	6.67
4	-4	-6	75.28	6.57
4	-4	-6	80.37	6.59
0	-4	6	78.41	6.57
4	0	6	68.09	6.52
0	-4	6	83.71	6.53
4	0	6	81.17	6.58
1	3	-6	26.29	2.44
-4	1	-6	27.56	2.59
3	-4	-6	31.73	2.36

7	-10	-4	7.46	0.83
-7	10	3	2.04	0.64
7	-10	-3	2.19	0.41
-7	10	2	3.62	0.72
7	-10	-2	4.57	0.63
-7	10	1	3.98	0.70
7	-10	-1	4.33	0.63
-7	10	0	14.87	1.37
7	-10	0	14.09	1.20
7	-10	1	2.92	0.46
7	-10	1	5.46	0.80
7	-10	2	4.87	0.66
7	-10	2	6.23	0.99
7	-10	3	1.96	0.42
7	-10	3	3.40	0.93
7	-10	4	6.95	0.79
7	-10	4	8.10	1.23
7	-10	5	1.40	0.84
7	-10	5	1.02	0.40
-6	9	6	7.01	0.91
6	-9	-6	8.19	1.09
-6	9	5	0.13	0.53
6	-9	-5	0.33	0.36
6	-9	-5	0.23	0.26
-6	9	4	6.00	0.83
6	-9	-4	4.77	0.63
6	-9	-4	6.41	0.70
-6	9	3	0.24	0.41
6	-9	-3	0.21	0.25
-6	9	2	25.67	1.93
6	-9	-2	24.30	1.82
-6	9	1	1.53	0.42
-6	9	0	6.54	0.85
6	-9	0	8.53	0.73
6	-9	0	6.32	0.77
6	-9	0	8.09	1.13
-6	9	-1	0.20	0.43
6	-9	1	0.39	0.26
6	-9	1	0.46	0.52
6	-9	1	0.32	0.29
-6	9	-2	25.99	2.07
6	-9	2	27.33	2.19
6	-9	2	27.10	1.96
6	-9	2	27.27	1.98
6	-9	3	1.50	0.70
6	-9	4	5.38	0.66
6	-9	4	5.62	0.87
6	-9	5	0.30	0.30
6	-9	5	0.49	0.62
6	-9	6	8.38	1.27
-5	8	7	1.01	0.49
5	-8	-7	0.65	0.65
-5	8	6	4.53	0.65
5	-8	-6	4.71	0.71
-5	8	5	2.62	0.51
5	-8	-5	2.32	0.35
5	-8	-5	2.69	0.48
-5	8	4	31.14	2.34
5	-8	-4	31.99	2.23
-5	8	3	8.36	0.82
5	-8	-3	7.47	0.71
-5	8	2	8.49	0.86
5	-8	-2	9.51	0.78
-5	8	1	11.74	1.02
5	-8	-1	13.05	1.35
5	-8	-1	11.35	1.03
5	-8	-1	11.79	0.94
-5	8	0	65.13	4.42
5	-8	0	60.63	4.36
5	-8	0	71.67	4.57
5	-8	0	60.21	4.40
-5	8	-1	12.56	1.13
5	-8	1	12.33	1.01
5	-8	1	11.78	0.98
5	-8	1	13.00	1.27
5	-8	2	8.94	0.78
5	-8	2	8.66	1.06
5	-8	2	9.17	0.79
5	-8	3	6.92	0.66
5	-8	3	7.82	0.99
5	-8	3	6.75	0.69
5	-8	4	31.08	2.30
5	-8	4	33.25	2.30
5	-8	4	33.68	2.53
5	-8	5	2.68	0.39
5	-8	5	2.78	0.38
5	-8	5	2.74	0.79
5	-8	6	5.12	0.97
5	-8	6	5.68	0.77
5	-8	7	1.09	0.89
4	-7	-8	5.77	1.01
-4	7	7	0.46	0.40
4	-7	-7	0.38	0.62
-4	7	6	16.57	1.36
4	-7	-6	16.49	1.43
-4	7	5	1.66	0.38
4	-7	-5	1.34	0.26
-4	7	4	27.45	2.01
4	-7	-4	27.70	1.94
-4	7	3	4.59	0.62
4	-7	-3	3.90	0.44
4	-7	-2	49.63	3.31
4	-7	-2	40.71	2.87
4	-7	-2	39.32	2.93
4	-7	-2	39.97	2.97
-4	7	1	5.17	0.66
4	-7	-1	5.50	0.60
4	-7	-1	5.49	0.51
4	-7	-1	5.81	0.67
4	-7	-1	5.97	0.77
-4	7	0	56.56	3.83
-4	7	0	55.68	3.84
4	-7	0	55.88	3.94
4	-7	0	54.74	3.81
4	-7	0	55.50	3.78
-4	7	-1	6.01	0.72
-4	7	-1	5.90	0.66

3	-4	-6	28.04	2.47
-1	-3	6	26.79	2.39
-3	4	6	26.39	2.32
4	-1	6	26.42	2.40
4	-1	6	25.30	2.43
-4	2	-6	1.72	0.32
2	2	-6	1.10	0.30
2	-4	-6	0.81	0.38
-4	2	-6	1.05	0.43
4	-2	6	1.09	0.27
3	1	-6	27.95	2.58
1	-4	-6	27.23	2.65
4	-3	6	29.11	2.55
4	-3	6	29.66	2.56
0	-4	-6	77.61	7.32
4	0	-6	85.58	7.31
4	-4	6	90.26	7.22
-1	-4	-6	48.69	4.85
5	-1	-6	56.31	4.88
4	-5	6	61.19	4.62
4	-5	6	46.75	4.46
6	-2	-6	38.82	3.80
-2	-4	-6	32.50	3.54
7	-3	-6	7.71	1.43
-3	-4	-6	4.70	1.09
-4	-4	-6	-0.26	0.83
8	-4	-6	-0.22	1.16
-5	-4	-6	-0.98	0.93
9	-5	-6	-0.18	1.47
-5	-3	-6	0.28	0.83
8	-5	-6	0.00	1.14
5	2	6	7.77	0.91
-5	-1	-6	8.04	1.41
5	1	6	8.59	0.97
1	-6	6	11.20	1.07
5	-5	-6	26.11	2.63
0	5	-6	26.40	2.55
5	0	6	29.08	2.59
0	-5	6	29.75	2.46
1	4	-6	50.36	4.33
4	-5	-6	45.22	4.32
4	-5	-6	49.60	4.32
-1	-4	6	51.55	4.26
5	-1	6	54.07	4.36
5	-1	6	47.23	4.29
3	-5	-6	20.95	2.02
2	3	-6	21.98	2.03
5	-2	6	22.44	2.03
5	-2	6	20.62	1.97
3	2	-6	23.00	2.39
5	-3	6	28.79	2.38
4	1	-6	57.55	5.54
5	-4	6	68.05	5.44
5	0	-6	31.95	3.01
0	-5	-6	25.53	2.80
-1	-5	-6	11.43	1.97
6	-1	-6	13.52	2.32
5	-6	6	17.49	1.55
7	-2	-6	7.43	1.57
-2	-5	-6	7.14	1.22
-3	-5	-6	2.09	0.95
8	-3	-6	2.17	1.24
9	-4	-6	-0.33	1.48
-4	-5	-6	-0.52	0.97
9	-6	-6	0.85	1.63
-6	-1	-6	0.97	0.96
7	-6	-6	1.26	0.98
6	1	6	4.54	0.69
6	1	6	2.80	0.54
6	-6	-6	0.47	0.70
6	-6	-6	0.74	0.42
-6	0	-6	1.46	0.94
6	0	6	1.11	0.44
6	0	6	0.66	0.29
0	-6	6	0.59	0.21
1	5	-6	14.25	1.49
5	-6	-6	14.53	1.40
6	-1	6	13.48	1.58
6	-1	6	12.92	1.40
-1	-5	6	13.36	1.31
4	-6	-6	41.90	3.94
2	4	-6	44.96	4.05
6	-2	6	46.18	4.06
6	-2	6	47.72	4.00
3	-6	-6	43.53	4.08
3	3	-6	45.52	4.58
6	-3	6	49.81	4.11
2	-6	-6	41.49	3.89
4	2	-6	45.77	4.56
1	-6	-6	12.57	1.80
5	1	-6	16.50	2.18
6	-5	6	19.22	1.70
0	-6	-6	1.84	0.76
6	0	-6	0.98	1.01
6	-6	6	0.82	0.36
-1	-6	-6	4.28	1.09
7	-1	-6	2.85	1.38
-2	-6	-6	6.40	1.22
8	-2	-6	7.07	1.80
-3	-6	-6	3.61	1.20
9	-3	-6	0.34	1.63
9	-7	-6	0.50	1.49
-7	-2	-6	1.58	0.91
7	1	6	7.25	1.00
-7	0	-6	3.33	0.98
0	-7	6	6.72	0.71
7	0	6	5.34	1.02
7	0	6	4.87	0.93
6	-7	-6	5.00	0.82
1	6	-6	6.19	1.09
-7	1	-6	6.40	1.20
7	-1	6	3.45	1.20
7	-1	6	5.30	1.11
-1	-6	6	5.78	0.71
2	5	-6	14.44	1.56
5	-7	-6	12.26	1.36
7	-2	6	11.02	2.01

4	-7	1	5.55	0.57
4	-7	1	5.53	0.52
4	-7	1	5.88	0.73
4	-7	2	41.65	2.71
4	-7	2	35.24	2.78
4	-7	2	39.86	2.69
4	-7	3	3.43	0.45
4	-7	3	3.99	0.58
4	-7	3	4.08	0.48
4	-7	4	28.99	2.04
4	-7	4	30.23	2.27
4	-7	4	28.32	2.05
4	-7	5	1.81	0.62
4	-7	5	1.98	0.30
4	-7	5	1.29	0.28
4	-7	6	15.69	1.37
4	-7	6	17.14	1.70
4	-7	6	15.51	1.21
4	-7	7	0.88	0.73
4	-7	8	6.28	1.07
3	-6	-9	-0.76	0.94
3	-6	-8	4.41	0.95
3	-6	-7	-0.05	0.56
-3	6	6	47.05	3.21
3	-6	-6	45.79	3.34
-3	6	4	6.59	0.68
3	-6	-4	7.32	0.58
-3	6	3	0.02	0.24
-3	6	3	0.25	0.24
3	-6	-3	-0.02	0.14
3	-6	-3	-0.02	0.22
3	-6	-3	-0.06	0.24
-3	6	2	204.99	13.00
-3	6	2	192.75	12.97
3	-6	-2	195.12	12.93
3	-6	-2	185.30	12.98
3	-6	-2	199.13	13.00
3	-6	-2	186.48	12.95
-3	6	1	0.15	0.20
-3	6	1	0.15	0.22
-3	6	1	-0.11	0.22
3	-6	-1	0.11	0.22
3	-6	-1	-0.03	0.14
3	-6	-1	-0.02	0.18
3	-6	-1	-0.12	0.13
3	-6	-1	-0.25	0.40
-3	6	0	5.85	0.58
-3	6	0	6.11	0.52
3	-6	0	6.78	0.60
3	-6	0	5.50	0.87
3	-6	0	5.85	0.57
-3	6	-1	0.10	0.19
-3	6	-1	0.06	0.15
-3	6	-1	0.03	0.26
3	-6	1	-0.16	0.34
3	-6	1	-0.02	0.18
3	-6	1	-0.02	0.21
3	-6	1	0.21	0.15
-3	6	-2	204.24	13.30
-3	6	-2	175.61	13.31
3	-6	2	205.73	13.29
3	-6	2	213.90	13.32
3	-6	2	197.73	13.32
-3	6	-3	0.06	0.16
3	-6	3	0.58	0.20
3	-6	3	-0.04	0.37
3	-6	3	-0.02	0.15
3	-6	3	0.11	0.19
-3	6	-4	7.15	0.63
3	-6	4	7.20	0.94
3	-6	4	7.85	0.65
3	-6	4	7.01	0.61
-3	6	-5	0.14	0.19
3	-6	5	-0.07	0.18
3	-6	5	0.64	0.22
3	-6	5	0.19	0.47
-3	6	-6	45.76	3.19
3	-6	6	46.87	3.20
3	-6	6	47.37	3.36
-3	6	-7	0.04	0.21
3	-6	7	0.27	0.54
-3	6	-8	4.25	0.53
-3	6	-9	-0.09	0.25
2	-5	-9	0.25	0.92
2	-5	-8	14.56	1.07
2	-5	-8	14.27	1.66
2	-5	-7	-0.34	0.44
2	-5	-7	-0.17	0.15
-2	5	6	20.74	1.42
2	-5	-6	19.68	1.44
2	-5	-6	19.70	1.40
2	-5	-6	18.48	1.61
-2	5	5	1.03	0.23
-2	5	5	0.78	0.38
2	-5	-5	1.07	0.17
2	-5	-5	0.81	0.19
-2	5	4	90.85	6.12
-2	5	4	90.97	6.00
2	-5	-4	96.08	5.99
2	-5	-4	78.52	6.27
-2	5	3	3.81	0.59
-2	5	3	3.92	0.43
-2	5	3	3.98	0.41
2	-5	-3	3.53	0.50
2	-5	-3	3.84	0.38
2	-5	-3	3.87	0.36
-2	5	2	57.79	3.74
-2	5	2	53.65	3.81
-2	5	2	53.99	3.73
2	-5	-2	56.33	3.72
2	-5	-2	55.84	3.72
2	-5	-2	53.09	3.76
2	-5	-2	55.55	3.76
-2	5	1	5.65	0.51
-2	5	1	5.72	0.50
-2	5	1	5.83	0.48
-2	5	1	5.66	0.59

-2	-5	6	13.09	1.29
7	-2	6	13.39	1.51
3	4	-6	17.76	1.92
4	-7	-6	15.91	1.70
7	-3	6	17.34	1.99
7	-3	6	17.36	1.86
4	3	-6	18.28	2.17
7	-4	6	19.31	2.03
7	-5	6	23.66	2.29
7	-5	6	22.90	2.32
6	1	-6	3.25	1.16
1	-7	-6	5.50	0.99
0	-7	-6	7.22	1.34
7	0	-6	4.86	1.64
-1	-7	-6	7.05	1.28
8	-1	-6	6.15	1.95
-2	-7	-6	1.65	1.15
9	-2	-6	2.04	2.12
8	-8	-6	1.88	1.90
-8	0	-6	3.10	1.01
8	0	6	4.77	1.01
1	7	-6	13.86	1.77
7	-8	-6	13.27	1.46
8	-1	6	11.74	1.96
-1	-7	6	12.78	1.31
2	6	-6	13.33	1.70
6	-8	-6	12.74	1.33
8	-2	6	10.59	1.51
-2	-6	6	12.32	1.24
8	-2	6	10.26	1.94
3	5	-6	3.44	0.71
5	-8	-6	4.09	0.65
8	-3	6	2.86	0.71
8	-3	6	2.32	0.80
4	-8	-6	0.88	0.53
4	4	-6	1.18	0.68
8	-4	6	1.26	0.61
8	-4	6	1.60	0.72
3	-8	-6	3.54	0.77
5	3	-6	3.30	1.01
8	-5	6	6.68	1.42
8	-6	6	22.85	2.51
8	-6	6	25.18	2.53
7	1	-6	11.07	2.32
1	-8	-6	10.44	1.81
8	0	-6	7.10	2.43
0	-8	-6	5.51	1.26
2	7	-6	4.57	1.08
-2	-7	6	5.83	0.91
9	-2	6	3.64	0.81
9	-2	6	3.81	1.03
6	-9	-6	6.14	1.16
-3	-6	6	7.24	0.97
9	-3	6	4.98	1.14
9	-3	6	4.06	0.98
4	5	-6	3.33	1.13
5	-9	-6	3.25	0.64
9	-4	6	3.69	0.99
9	-4	6	3.72	0.90
5	4	-6	3.01	1.38
4	-9	-6	3.04	0.70
9	-5	6	2.94	0.88
9	-6	6	12.56	2.19
9	-6	6	15.23	2.24
9	-7	6	10.86	2.05
0	0	-7	0.23	0.26
0	0	7	0.09	0.16
0	0	7	-0.10	0.12
1	-1	-7	2.20	0.42
-1	0	-7	2.63	0.46
0	-1	7	1.97	0.39
-1	1	7	2.53	0.45
1	0	7	2.04	0.43
0	-1	7	2.14	0.45
1	0	-7	2.31	0.41
0	-1	-7	2.34	0.46
-1	1	-7	2.56	0.42
1	-1	7	2.36	0.39
0	1	7	3.05	0.40
1	-1	7	2.34	0.45
-1	0	7	1.67	0.39
-1	0	7	2.37	0.43
1	-1	7	3.15	0.56
-1	-1	-7	0.36	0.41
2	-1	-7	0.11	0.14
-2	1	7	0.21	0.24
1	1	7	0.06	0.14
-2	1	7	0.04	0.16
1	-2	7	0.10	0.24
1	-2	7	-0.10	0.18
3	-2	-7	2.24	0.43
-2	-1	-7	3.00	0.71
2	1	7	2.45	0.39
1	-3	7	1.81	0.35
-3	2	7	2.83	0.43
-3	2	7	3.00	0.47
1	-3	7	2.55	0.51
1	-3	7	2.54	0.41
-2	0	-7	36.32	2.90
2	-2	-7	32.85	2.68
2	-2	-7	28.11	2.82
0	-2	7	31.52	2.76
2	0	7	32.26	2.70
-2	2	7	30.42	2.73
0	-2	7	33.96	2.76
-2	2	7	27.33	2.69
1	1	-7	0.10	0.17
-2	1	-7	-0.05	0.25
1	-2	-7	-0.17	0.32
2	-1	7	0.40	0.20
2	-1	7	0.36	0.29
-1	-1	7	-0.10	0.15
-1	-1	7	0.06	0.15
2	-1	7	-0.09	0.17
0	-2	-7	32.30	2.96
2	0	-7	31.07	2.79
2	-2	7	30.46	2.82

2	-5	-1	5.97	0.55
2	-5	-1	5.77	0.47
2	-5	-1	5.43	0.71
2	-5	-1	5.12	0.51
2	-5	-1	6.18	0.46
-2	5	0	170.78	11.69
-2	5	0	164.71	11.77
-2	5	0	180.32	11.73
-2	5	0	192.29	11.72
2	-5	0	183.63	11.77
2	-5	0	173.08	11.70
2	-5	0	164.10	11.69
2	-5	0	176.76	11.72
2	-5	0	174.33	11.71
-2	5	-1	6.04	0.47
-2	5	-1	5.56	0.55
2	-5	1	6.14	0.46
2	-5	1	5.97	0.51
2	-5	1	5.75	0.53
2	-5	1	4.43	0.62
-2	5	-2	55.19	3.72
-2	5	-2	46.43	3.73
2	-5	2	53.47	3.77
2	-5	2	59.53	3.75
2	-5	2	57.75	3.74
2	-5	2	58.94	3.72
-2	5	-3	3.88	0.38
2	-5	3	3.44	0.49
2	-5	3	3.91	0.42
2	-5	3	3.76	0.36
2	-5	3	4.00	0.60
-2	5	-4	94.45	6.32
2	-5	4	96.00	6.32
2	-5	4	90.88	6.39
2	-5	4	96.21	6.34
-2	5	-5	0.72	0.18
2	-5	5	0.69	0.19
2	-5	5	0.97	0.21
2	-5	5	1.03	0.38
-2	5	-6	18.92	1.42
2	-5	6	20.63	1.45
2	-5	6	20.96	1.57
-2	5	-7	0.20	0.18
2	-5	7	0.64	0.21
-2	5	-8	14.36	1.07
-2	5	-9	0.08	0.20
1	-4	-10	3.11	0.42
1	-4	-10	3.53	0.88
1	-4	-9	-0.03	0.58
1	-4	-9	0.23	0.16
1	-4	-8	19.23	1.37
1	-4	-8	19.33	1.71
1	-4	-7	1.05	0.47
1	-4	-7	1.46	0.22
1	-4	-6	22.32	1.56
1	-4	-6	23.41	1.58
1	-4	-6	20.90	1.77
-1	4	5	10.13	0.72
1	-4	-5	9.68	0.71
1	-4	-5	9.29	0.73
-1	4	4	132.04	8.56
-1	4	4	118.47	8.76
1	-4	-4	128.76	8.57
1	-4	-4	133.99	8.56
-1	4	3	31.82	2.09
-1	4	3	28.15	2.26
-1	4	3	32.60	2.08
1	-4	-3	30.25	2.13
1	-4	-3	32.52	2.08
1	-4	-3	27.49	2.14
-1	4	2	58.42	3.82
-1	4	2	53.82	3.94
-1	4	2	58.47	3.83
1	-4	-2	55.37	3.84
1	-4	-2	58.34	3.85
1	-4	-2	56.09	3.81
1	-4	-2	56.93	3.81
-1	4	1	41.92	3.08
-1	4	1	46.35	3.10
-1	4	1	43.93	3.20
1	-4	-1	48.01	3.08
1	-4	-1	46.81	3.10
1	-4	-1	47.49	3.08
1	-4	-1	45.47	3.11
-1	4	0	260.23	18.23
-1	4	0	273.06	18.14
-1	4	0	278.32	18.13
-1	4	0	281.02	18.16
1	-4	0	263.39	18.13
1	-4	0	283.10	18.18
1	-4	0	256.27	18.14
1	-4	0	278.95	18.16
1	-4	0	280.19	18.13
-1	4	-1	45.05	3.14
-1	4	-1	46.55	3.23
1	-4	1	46.81	3.13
1	-4	1	45.72	3.12
1	-4	1	47.25	3.15
-1	4	-2	58.46	3.88
1	-4	2	60.00	3.88
1	-4	2	57.08	3.91
1	-4	2	58.78	3.90
1	-4	2	54.66	3.90
-1	4	-3	29.20	2.12
1	-4	3	35.35	2.15
1	-4	3	30.56	2.12
1	-4	3	30.04	2.18
-1	4	-4	148.12	9.50
1	-4	4	140.15	9.50
1	-4	4	135.50	9.53
1	-4	4	146.46	9.52
-1	4	-5	9.19	0.70
1	-4	5	9.43	0.76
1	-4	5	9.87	0.70
-1	4	-6	22.89	1.62
1	-4	6	24.21	1.67
-1	4	-7	1.59	0.21

2	-2	7	36.19	2.82
-2	0	7	28.17	2.79
2	-2	7	33.02	2.81
-2	0	7	34.04	2.83
3	-1	-7	2.74	0.46
-1	-2	-7	2.57	0.71
1	2	7	1.98	0.34
2	-3	7	2.77	0.55
2	-3	7	2.08	0.36
-2	-2	-7	-0.53	0.66
4	-2	-7	0.15	0.21
2	2	7	-0.05	0.22
-4	2	7	0.41	0.27
2	-4	7	-0.08	0.22
5	-3	-7	0.42	0.35
-3	-2	-7	0.60	1.37
3	2	7	-0.06	0.26
-5	3	7	0.33	0.26
2	-5	7	0.45	0.23
2	-5	7	0.05	0.23
-5	3	7	0.70	0.45
4	-3	-7	1.63	0.30
-3	-1	-7	1.77	0.72
3	1	7	1.38	0.28
1	-4	7	1.28	0.36
1	-4	7	1.26	0.29
-4	3	7	1.42	0.24
-3	0	-7	0.28	0.51
3	-3	-7	0.28	0.34
0	-3	7	0.20	0.19
3	0	7	0.17	0.17
3	0	7	0.36	0.19
-3	3	7	0.83	0.34
-3	1	-7	3.44	0.63
1	2	-7	2.64	0.53
-3	1	-7	3.19	0.50
2	-3	-7	1.95	0.48
3	-1	7	1.95	0.42
-1	-2	7	3.10	0.50
-2	3	7	2.34	0.36
3	-1	7	2.30	0.41
3	-1	7	2.04	0.36
-1	-2	7	2.43	0.46
-3	2	-7	2.73	0.48
1	-3	-7	2.45	0.58
3	-2	7	2.53	0.48
-2	-1	7	2.70	0.53
3	-2	7	2.56	0.46
3	-2	7	2.03	0.37
0	-3	-7	1.12	0.58
3	0	-7	0.49	0.23
-3	0	7	0.55	0.29
3	-3	7	0.19	0.24
3	-3	7	0.33	0.23
3	-3	7	0.37	0.22
4	-1	-7	3.08	0.64
-1	-3	-7	0.99	0.74
3	-4	7	1.89	0.37
-4	1	7	2.28	0.47
3	-4	7	1.62	0.38
-2	-3	-7	0.03	0.71
5	-2	-7	-0.30	0.47
3	-5	7	-0.04	0.25
3	-5	7	0.36	0.18
6	-3	-7	0.22	0.58
-3	-3	-7	0.48	0.91
3	-6	7	0.01	0.26
7	-4	-7	-0.52	0.83
-4	-3	-7	-0.03	0.84
3	-7	7	0.54	0.28
-4	-2	-7	3.99	1.11
6	-4	-7	2.32	0.68
4	2	7	3.31	0.52
-4	-1	-7	0.53	0.82
5	-4	-7	0.28	0.31
4	1	7	0.29	0.28
1	-5	7	0.08	0.20
4	-4	-7	13.75	1.34
4	-4	-7	13.11	1.17
4	0	7	12.71	1.25
4	0	7	11.25	1.18
0	-4	7	10.80	1.27
3	-4	-7	1.93	0.47
-4	1	-7	1.71	0.55
4	-1	7	1.61	0.30
-1	-3	7	1.20	0.26
4	-1	7	1.18	0.25
4	-1	7	1.17	0.30
2	2	-7	0.20	0.25
2	-4	-7	-0.21	0.41
-2	-2	7	0.11	0.20
4	-2	7	0.53	0.24
4	-2	7	-0.06	0.23
1	-4	-7	1.78	0.61
3	1	-7	1.57	0.37
-4	3	-7	2.67	0.44
4	-3	7	1.91	0.36
4	-3	7	1.27	0.33
4	0	-7	12.38	1.41
0	-4	-7	11.39	1.67
4	-4	7	14.25	1.35
5	-1	-7	0.45	0.44
-1	-4	-7	-0.21	0.64
4	-5	7	0.26	0.30
-2	-4	-7	2.35	0.88
6	-2	-7	3.26	0.84
-3	-4	-7	-1.28	0.88
7	-3	-7	0.49	0.88
4	-7	7	0.37	0.32
8	-4	-7	-1.60	1.17
-4	-4	-7	-0.66	0.95
4	-8	7	0.21	0.35
8	-5	-7	-0.71	1.07
-5	-3	-7	0.81	0.88
3	-8	7	0.47	0.31
-5	-2	-7	0.99	0.85
7	-5	-7	-0.32	0.72

1	-4	7	1.46	0.26
-1	4	-8	16.54	1.18
-1	4	-9	0.20	0.17
0	-3	-10	6.56	0.61
0	-3	-10	6.37	1.45
0	-3	-9	0.10	0.96
0	-3	-9	0.09	0.16
0	-3	-8	10.99	0.84
0	-3	-8	10.45	1.32
0	-3	-7	0.38	0.49
0	-3	-7	0.29	0.19
0	-3	-6	66.17	4.58
0	-3	-6	67.87	4.76
0	-3	-6	70.17	4.58
0	3	5	1.20	0.14
0	-3	-5	0.74	0.19
0	3	4	41.40	2.71
0	3	4	40.28	2.72
0	-3	-4	39.63	2.75
0	3	3	0.93	0.16
0	3	3	1.43	0.19
0	3	3	1.06	0.46
0	3	3	1.45	0.17
0	-3	-3	1.27	0.19
0	-3	-3	1.92	0.24
0	-3	-3	1.21	0.19
0	3	2	282.14	19.77
0	3	2	301.81	19.61
0	3	2	312.89	19.60
0	3	2	291.00	19.62
0	-3	-2	288.60	19.62
0	-3	-2	313.59	19.62
0	-3	-2	274.39	19.62
0	3	1	1.59	0.19
0	3	1	1.81	0.22
0	3	1	1.93	0.38
0	3	1	1.91	0.23
0	-3	-1	1.58	0.22
0	-3	-1	2.16	0.22
0	-3	-1	1.89	0.32
0	-3	-1	1.66	0.25
0	3	0	86.67	6.04
0	3	0	91.75	5.90
0	3	0	86.26	5.90
0	-3	0	87.75	5.91
0	-3	0	90.27	5.90
0	-3	0	85.72	5.93
0	3	-1	2.03	0.25
0	3	-1	1.86	0.40
0	3	-1	2.00	0.20
0	3	-1	1.56	0.25
0	-3	1	1.72	0.24
0	-3	1	1.79	0.23
0	3	-2	301.74	18.93
0	-3	2	264.38	18.94
0	-3	2	287.86	18.95
0	3	-3	1.44	0.18
0	-3	3	1.39	0.20
0	-3	3	1.51	0.21
0	3	-4	40.77	2.72
0	-3	4	40.55	2.72
0	-3	4	40.19	2.77
0	3	-5	0.95	0.13
0	3	-5	2.94	0.74
0	-3	5	0.80	0.13
0	-3	5	0.70	0.20
0	3	-6	66.71	4.41
0	3	-6	66.09	4.73
0	-3	6	64.94	4.46
0	3	-7	-0.14	0.86
0	3	-7	0.33	0.12
0	-3	7	0.39	0.20
0	3	-8	11.11	0.82
0	3	-8	13.76	1.88
0	3	-9	0.70	1.30
0	3	-10	6.66	1.86
-1	-2	-10	3.70	1.38
-1	-2	-10	4.20	0.58
-1	-2	-9	1.57	1.00
-1	-2	-8	20.36	1.47
-1	-2	-8	18.05	1.88
-1	-2	-7	1.70	0.63
-1	-2	-7	1.73	0.35
-1	-2	-6	41.63	2.84
-1	-2	-6	39.40	2.94
-1	-2	-5	11.56	0.96
1	2	4	157.57	9.83
-1	-2	-4	137.89	9.89
1	2	3	36.26	2.48
1	2	3	35.30	2.54
1	2	3	38.24	2.48
-1	-2	-3	37.14	2.50
-1	-2	-3	38.32	2.55
1	2	2	118.11	7.96
1	2	2	119.78	7.98
1	2	2	122.97	7.96
-1	-2	-2	116.99	7.97
-1	-2	-2	117.82	7.97
-1	-2	-2	121.86	8.00
1	2	1	43.23	3.04
1	2	1	46.89	3.03
1	2	1	45.31	3.04
1	2	1	40.32	3.24
1	2	1	45.68	3.02
-1	-2	-1	38.45	3.07
-1	-2	-1	52.12	3.06
-1	-2	-1	44.62	3.02
-1	-2	-1	45.83	3.04
1	2	0	275.48	19.28
1	2	0	300.13	19.14
1	2	0	300.17	19.13
1	2	0	290.73	19.14
1	2	0	277.00	19.13
-1	-2	0	265.38	19.13
-1	-2	0	305.82	19.13
1	2	-1	48.41	3.23
1	2	-1	47.76	3.36

2	-7	7	0.13	0.25
-5	-1	-7	1.77	1.21
6	-5	-7	0.44	0.51
5	1	7	0.92	0.42
5	1	7	0.57	0.27
1	-6	7	0.81	0.24
5	-5	-7	0.24	0.33
-5	0	-7	-0.42	0.75
5	-5	-7	-0.07	0.39
5	0	7	0.27	0.24
0	-5	7	0.09	0.20
5	0	7	0.75	0.36
-5	1	-7	0.53	0.54
4	-5	-7	0.10	0.36
-1	-4	7	0.27	0.20
5	-1	7	0.02	0.21
5	-1	7	0.34	0.32
3	-5	-7	1.52	0.52
2	3	-7	0.22	0.31
-5	2	-7	0.31	0.34
5	-2	7	0.15	0.24
5	-2	7	0.20	0.31
-2	-3	7	0.00	0.21
3	2	-7	0.07	0.38
2	-5	-7	0.16	0.48
-5	3	-7	0.27	0.26
5	-3	7	0.18	0.29
4	1	-7	0.04	0.36
1	-5	-7	0.44	0.57
5	-4	7	0.51	0.47
0	-5	-7	0.06	0.64
5	0	-7	0.99	0.52
5	-5	7	0.30	0.34
-1	-5	-7	-0.01	0.82
6	-1	-7	0.78	0.80
5	-6	7	0.86	0.37
7	-2	-7	-0.13	1.03
-2	-5	-7	-0.04	0.87
5	-7	7	0.25	0.34
-3	-5	-7	0.40	1.00
8	-3	-7	-1.09	1.32
5	-8	7	0.49	0.40
-6	-2	-7	-0.79	0.81
8	-6	-7	-0.28	0.98
-6	-1	-7	0.10	0.90
7	-6	-7	-0.02	0.68
6	1	7	0.13	0.42
1	-7	7	0.46	0.26
6	-6	-7	0.45	0.50
0	-6	7	-0.07	0.18
6	0	7	0.08	0.42
6	0	7	0.04	0.23
1	5	-7	0.69	0.38
5	-6	-7	1.83	0.51
-6	1	-7	3.01	0.87
6	-1	7	0.79	0.37
6	-1	7	0.58	0.49
-1	-5	7	0.84	0.27
4	-6	-7	3.93	0.66
2	4	-7	4.98	0.99
6	-2	7	4.37	0.78
6	-2	7	3.85	0.70
-2	-4	7	3.95	0.66
3	-6	-7	0.17	0.48
-6	3	-7	-0.24	0.29
3	3	-7	0.07	0.38
6	-3	7	-0.23	0.32
6	-3	7	0.00	0.49
4	2	-7	6.27	1.24
2	-6	-7	5.11	0.98
6	-4	7	7.79	1.07
6	-4	7	6.92	1.01
5	1	-7	1.12	0.66
1	-6	-7	1.15	0.78
6	-5	7	1.37	0.42
6	0	-7	-0.24	0.80
0	-6	-7	-0.55	0.70
6	-6	7	0.25	0.37
7	-1	-7	-0.44	1.11
-1	-6	-7	-0.10	0.94
6	-7	7	0.48	0.46
-2	-6	-7	-0.17	1.07
8	-2	-7	0.34	1.47
6	-8	7	1.30	0.52
-7	-1	-7	-0.40	0.94
1	-8	7	0.40	0.31
-7	0	-7	1.51	1.01
0	-7	7	0.08	0.24
7	0	7	0.62	0.58
6	-7	-7	0.37	0.45
-7	1	-7	0.62	1.03
1	6	-7	0.11	0.43
7	-1	7	0.37	0.33
7	-1	7	-0.06	0.55
-1	-6	7	0.25	0.26
5	-7	-7	-0.21	0.46
-7	2	-7	0.41	0.71
2	5	-7	0.87	0.46
-2	-5	7	0.13	0.27
7	-2	7	0.40	0.42
7	-2	7	-0.42	0.54
4	-7	-7	-0.27	0.51
3	4	-7	0.71	0.49
7	-3	7	0.01	0.40
7	-3	7	0.39	0.57
4	3	-7	0.26	0.57
3	-7	-7	0.42	0.55
7	-4	7	0.37	0.47
7	-4	7	0.36	0.49
2	-7	-7	-0.75	0.76
5	2	-7	0.56	0.70
7	-5	7	0.16	0.42
7	-5	7	0.87	0.50
1	-7	-7	-0.50	0.84
6	1	-7	1.28	0.98
7	-6	7	0.38	0.45
7	0	-7	-0.50	1.28
1	2	-1	51.42	3.21
-1	-2	1	49.12	3.23
-1	-2	1	46.10	3.23
-1	-2	1	42.34	3.25
1	2	-2	109.96	7.80
1	2	-2	122.78	7.77
1	2	-2	115.42	7.91
1	2	-2	120.71	7.81
-1	-2	2	112.26	7.77
-1	-2	2	118.94	7.81
1	2	-3	38.68	2.59
1	2	-3	40.35	2.76
-1	-2	3	36.47	2.64
1	2	-4	140.17	9.92
1	2	-4	158.66	9.79
-1	-2	4	141.87	9.84
1	2	-5	11.32	1.20
1	2	-5	11.69	0.78
-1	-2	5	10.05	0.87
1	2	-6	40.00	3.00
1	2	-6	39.55	2.70
-1	-2	6	40.98	2.79
1	2	-7	1.50	0.73
1	2	-7	2.03	0.24
-1	-2	7	1.70	0.30
1	2	-8	19.47	2.03
1	2	-8	18.97	1.32
-1	-2	8	19.49	1.56
1	2	-9	0.47	0.15
1	2	-9	-0.35	1.11
1	2	-10	6.29	1.68
-2	-1	-10	6.02	1.45
-2	-1	-10	4.59	0.65
-2	-1	-9	1.20	0.97
-2	-1	-8	18.17	1.58
-2	-1	-8	19.48	1.85
-2	-1	-7	2.51	0.58
-2	-1	-7	1.50	0.61
2	1	6	45.84	2.95
-2	-1	-6	39.66	3.10
2	1	5	11.09	0.82
-2	-1	-5	10.93	1.03
2	1	4	159.36	9.70
2	1	4	133.30	9.65
-2	-1	-4	143.39	9.79
2	1	3	35.24	2.52
2	1	3	36.95	2.48
2	1	3	33.57	2.55
-2	-1	-3	42.73	2.63
2	1	2	121.21	7.58
2	1	2	102.99	7.65
2	1	2	115.07	7.58
2	1	2	117.06	7.67
2	1	2	116.32	7.62
-2	-1	-2	112.56	7.57
-2	-1	-2	113.80	7.66
-2	-1	-2	110.89	7.59
2	1	1	46.63	2.98
2	1	1	44.84	2.93
2	1	1	40.52	2.90
2	1	1	46.97	3.12
2	1	1	36.60	2.95
-2	-1	-1	39.70	2.95
2	1	0	265.93	18.58
2	1	0	289.17	18.44
2	1	0	284.04	18.42
2	1	0	252.58	18.46
2	1	0	268.17	18.42
2	1	0	274.72	18.43
-2	-1	0	277.97	18.43
-2	-1	0	301.88	18.43
2	1	-1	44.46	3.14
2	1	-1	46.35	3.11
2	1	-1	49.05	3.30
2	1	-1	42.77	3.11
2	1	-1	47.46	3.15
-2	-1	1	47.81	3.11
-2	-1	1	42.24	3.14
2	1	-2	114.52	7.64
2	1	-2	120.42	7.70
2	1	-2	115.10	7.65
2	1	-2	118.22	7.79
-2	-1	2	107.87	7.70
-2	-1	2	111.23	7.63
2	1	-3	34.49	2.45
2	1	-3	37.06	2.59
2	1	-3	38.98	2.45
2	1	-3	34.95	2.50
2	1	-3	37.54	2.60
-2	-1	3	34.09	2.53
2	1	-4	146.74	9.84
2	1	-4	140.94	9.80
2	1	-4	149.91	9.74
-2	-1	4	147.35	9.83
2	1	-5	10.75	0.79
-2	-1	5	11.68	0.97
2	1	-6	40.20	2.74
-2	-1	6	41.16	2.87
2	1	-7	1.60	0.21
2	1	-7	2.50	0.62
-2	-1	7	1.86	0.33
2	1	-8	17.90	1.36
2	1	-8	21.80	1.80
-2	-1	8	20.49	1.51
2	1	-9	0.18	0.71
2	1	-9	-0.04	0.20
2	1	-10	5.29	1.10
-3	0	-10	9.31	1.29
-3	0	-10	5.91	1.27
-3	0	-10	5.58	1.38
-3	0	-9	0.48	0.52
-3	0	-9	1.08	0.68
-3	0	-9	-0.14	0.85
-3	0	-8	7.69	1.09
-3	0	-8	12.08	1.39
-3	0	-7	-0.18	0.46
-3	0	-7	0.23	0.53
0	-7	-7	0.54	1.02
7	-7	7	0.04	0.44
-1	-7	-7	0.27	1.24
7	-8	7	0.44	0.53
8	-1	7	0.08	0.75
-1	-7	7	-0.05	0.33
2	6	-7	1.24	0.70
6	-8	-7	1.59	0.55
8	-2	7	1.26	0.78
8	-2	7	0.74	0.76
-2	-6	7	0.98	0.39
5	-8	-7	0.05	0.50
3	5	-7	0.43	0.67
8	-3	7	0.65	0.60
8	-3	7	-0.05	0.72
-3	-5	7	-0.08	0.31
4	-8	-7	-0.25	0.65
4	4	-7	-0.58	0.77
8	-4	7	-0.51	0.59
8	-4	7	-0.49	0.67
3	-8	-7	-0.08	0.75
5	3	-7	0.30	0.95
8	-5	7	0.39	0.64
8	-5	7	1.23	0.72
2	-8	-7	1.02	0.96
6	2	-7	1.16	1.23
8	-6	7	1.32	0.64
8	-6	7	2.20	0.66
7	1	-7	1.52	1.82
1	-8	-7	0.68	1.15
8	-7	7	0.26	0.65
0	0	-8	109.60	8.53
0	0	8	105.46	8.49
0	0	8	85.37	8.40
-1	0	-8	45.85	3.67
1	-1	-8	39.94	3.67
0	-1	8	41.70	3.56
1	0	8	34.82	3.56
-1	1	8	41.42	3.57
0	-1	8	43.99	3.61
0	-1	-8	42.29	3.62
-1	1	-8	44.34	3.53
-1	0	8	41.52	3.49
-1	0	8	38.03	3.45
0	1	8	34.07	3.44
1	-1	8	39.70	3.51
1	-1	8	41.53	3.46
2	-1	-8	19.10	1.69
-1	-1	-8	21.18	2.02
1	-2	8	18.23	1.90
-2	1	8	16.85	1.72
-2	1	8	20.71	1.73
1	-2	8	17.66	1.78
3	-2	-8	27.10	2.22
2	1	8	22.68	2.21
1	-3	8	27.45	2.33
-3	2	8	24.04	2.25
2	-2	-8	9.86	1.19
-2	2	8	11.24	0.97
2	0	8	8.94	0.85
2	0	8	9.63	1.00
0	-2	8	11.36	1.04
-2	2	8	8.17	0.82
2	0	8	8.18	0.84
-2	1	-8	19.40	1.66
1	-2	-8	19.18	1.80
2	-1	8	17.61	1.55
-1	-1	8	16.94	1.60
2	-1	8	15.61	1.59
-1	-1	8	17.19	1.69
-1	2	8	14.49	1.51
2	0	-8	11.31	1.13
0	-2	-8	10.59	1.52
-2	0	8	11.83	1.18
2	-2	8	11.64	1.23
2	-2	8	12.81	1.17
-2	0	8	10.67	1.16
3	-1	-8	26.11	2.31
-1	-2	-8	30.45	2.72
-3	1	8	22.68	2.31
2	-3	8	28.02	2.40
1	2	8	23.13	2.23
2	-3	8	28.22	2.40
4	-2	-8	52.55	4.71
-2	-2	-8	54.87	5.16
2	-4	8	57.27	4.78
5	-3	-8	18.79	1.85
-3	-2	-8	16.61	2.62
2	-5	8	20.33	1.86
-3	-1	-8	29.81	3.44
4	-3	-8	24.93	2.26
1	-4	8	25.35	2.35
3	-3	8	15.74	1.69
-3	0	-8	19.18	1.80
-3	3	8	14.91	1.46
3	0	8	13.66	1.42
2	-3	-8	25.20	2.29
1	2	-8	23.92	2.13
3	-1	8	19.91	2.04
-1	-2	8	24.65	2.11
3	-1	8	25.07	2.12
-2	3	8	21.74	2.01
1	-3	-8	24.40	2.61
-3	2	-8	30.13	2.42
2	1	-8	25.70	2.43
3	-2	8	26.21	2.46
-2	-1	8	24.00	2.41
3	-2	8	30.90	2.42
3	0	-8	15.36	1.55
0	-3	-8	16.89	1.94
3	-3	8	16.51	1.48
3	-3	8	16.87	1.65
4	-1	-8	23.71	2.34
-1	-3	-8	26.33	2.85
3	-4	8	27.41	2.41
5	-2	-8	18.04	1.86
-2	-3	-8	16.90	2.40
3	0	6	68.95	4.33
-3	0	-6	57.35	4.41
-3	0	-6	64.21	4.45
3	0	5	0.33	0.26
3	0	5	0.42	0.23
-3	0	-5	0.70	0.36
-3	0	-5	0.19	0.37
3	0	4	36.85	2.77
3	0	4	41.45	2.67
3	0	4	40.45	2.71
3	0	3	1.82	0.33
3	0	3	1.12	0.23
3	0	3	1.58	0.25
-3	0	-3	1.09	0.35
3	0	2	247.72	18.37
3	0	2	320.15	18.35
3	0	2	279.67	18.41
-3	0	-2	264.88	18.35
-3	0	-2	286.23	18.40
-3	0	-2	273.87	18.34
-3	0	-2	256.81	18.41
-3	0	-2	275.01	18.39
3	0	1	1.61	0.37
-3	0	-1	1.24	0.28
-3	0	-1	1.96	0.36
-3	0	-1	1.26	0.33
3	0	0	115.22	8.01
3	0	0	126.52	7.99
3	0	0	103.15	7.95
3	0	0	106.96	7.95
3	0	0	120.04	8.09
-3	0	0	118.27	7.98
-3	0	0	125.90	7.98
-3	0	0	125.57	8.00
3	0	-1	2.17	0.33
3	0	-1	1.96	0.33
3	0	-1	1.13	0.33
3	0	-1	0.98	0.38
3	0	-1	1.28	0.26
-3	0	1	1.07	0.30
-3	0	1	1.02	0.30
-3	0	1	2.02	0.35
-3	0	1	1.84	0.27
3	0	-2	290.72	19.42
3	0	-2	275.51	19.40
3	0	-2	304.68	19.47
3	0	-2	286.80	19.51
-3	0	2	297.18	19.36
3	0	-3	1.67	0.38
3	0	-3	2.05	0.28
3	0	-3	1.31	0.33
3	0	-3	1.55	0.31
3	0	-3	1.07	0.27
-3	0	3	1.52	0.37
3	0	-4	36.80	2.64
3	0	-4	39.18	2.70
3	0	-4	39.68	2.72
3	0	-4	38.17	2.63
3	0	-5	0.75	0.26
3	0	-5	1.42	0.30
3	0	-5	0.86	0.16
3	0	-5	0.76	0.46
-3	0	5	0.12	0.36
3	0	-6	68.00	4.37
3	0	-6	61.03	4.23
-3	0	6	58.57	4.34
3	0	-7	0.42	0.32
3	0	-7	0.32	0.47
-3	0	7	0.16	0.32
3	0	-8	12.83	1.41
3	0	-8	9.54	1.06
-3	0	8	11.06	1.41
-3	0	8	12.83	1.10
3	0	-9	0.14	0.85
3	0	-10	7.45	1.23
-4	1	-10	4.93	1.37
-4	1	-9	0.69	0.90
-4	1	-9	-0.69	0.45
-4	1	-9	-0.88	0.67
-4	1	-8	15.33	1.41
-4	1	-8	20.80	1.81
-4	1	-8	16.06	1.70
-4	1	-7	2.35	0.64
-4	1	-7	0.93	0.52
-4	1	-7	1.39	0.42
4	-1	6	24.02	1.73
4	-1	6	22.22	1.58
-4	1	-6	21.71	1.76
-4	1	-6	23.72	1.80
4	-1	5	12.65	0.97
4	-1	5	14.05	1.15
-4	1	-5	13.38	1.27
4	-1	4	122.65	8.69
4	-1	4	132.44	8.68
4	-1	4	128.17	8.68
-4	1	-4	136.44	8.73
4	-1	3	27.52	2.03
4	-1	3	29.77	2.11
4	-1	3	31.17	2.04
-4	1	-3	29.25	1.99
-4	1	-3	27.60	2.00
4	-1	2	55.81	3.73
4	-1	2	55.88	3.74
4	-1	2	53.37	3.76
4	-1	2	49.63	3.76
-4	1	-2	53.87	3.75
-4	1	-2	54.57	3.70
-4	1	-2	57.28	3.75
4	-1	1	45.69	3.15
-4	1	-1	43.32	3.07
-4	1	-1	44.84	3.13
-4	1	-1	43.51	3.12
-4	1	-1	45.58	3.13
-4	1	-1	42.74	3.08
4	-1	0	252.52	15.39
-4	1	0	218.85	15.38
-4	1	0	213.33	15.35

3	-5	8	19.60	1.84
6	-3	-8	2.43	0.60
-3	-3	-8	2.55	1.01
-4	-3	-8	1.28	0.97
7	-4	-8	1.18	1.49
6	-4	-8	2.76	0.59
-4	-2	-8	1.71	1.03
2	-6	8	4.15	0.78
-4	-1	-8	6.76	1.32
5	-4	-8	8.45	0.95
1	-5	8	7.37	0.95
4	-4	-8	7.82	1.20
-4	0	-8	9.87	1.52
-4	4	8	7.48	0.83
4	0	8	7.00	0.92
0	-4	8	6.99	1.06
1	3	-8	22.71	2.02
3	-4	-8	22.42	2.12
-1	-3	8	21.85	1.94
4	-1	8	20.41	1.96
4	-1	8	18.15	1.88
2	-4	-8	53.05	4.68
2	2	-8	49.91	4.55
-2	-2	8	47.87	4.50
4	-2	8	58.47	4.58
1	-4	-8	23.95	2.60
3	1	-8	24.09	2.34
4	-3	8	27.29	2.38
0	-4	-8	8.56	1.64
4	0	-8	8.24	1.12
4	-4	8	8.07	1.09
-1	-4	-8	7.40	1.34
5	-1	-8	8.18	1.62
4	-5	8	8.41	1.11
-2	-4	-8	2.51	1.12
4	-6	8	5.46	0.95
7	-3	-8	1.33	0.85
-3	-4	-8	0.39	1.04
-5	-2	-8	1.85	1.01
-5	-1	-8	5.57	1.34
1	-6	8	9.22	1.02
5	-5	-8	8.53	1.30
-5	0	-8	12.04	1.83
0	-5	8	8.03	0.98
5	0	8	8.07	1.19
1	4	-8	6.97	1.00
4	-5	-8	7.53	1.15
-1	-4	8	7.27	0.85
5	-1	8	5.23	0.80
5	-1	8	6.77	1.04
3	-5	-8	17.46	1.83
2	3	-8	16.22	1.74
5	-2	8	17.42	1.76
-2	-3	8	17.25	1.62
5	-2	8	15.29	1.66
3	2	-8	17.93	1.76
2	-5	-8	15.81	1.93
5	-3	8	15.59	1.62
1	-5	-8	5.99	1.08
4	1	-8	7.12	1.14
0	-5	-8	5.42	1.36
5	0	-8	8.16	1.41
5	-5	8	9.65	1.22
6	-1	-8	4.83	0.94
-1	-5	-8	6.82	1.32
-2	-5	-8	1.26	1.16
7	-2	-8	2.56	0.99
-6	-1	-8	2.13	1.11
-6	0	-8	18.06	2.41
6	0	8	12.63	2.09
0	-6	8	14.82	1.49
1	5	-8	8.59	1.24
5	-6	-8	10.08	1.33
-1	-5	8	9.17	1.04
6	-1	8	6.92	1.44
4	-6	-8	3.03	0.72
2	4	-8	5.63	1.11
-2	-4	8	3.77	0.74
6	-2	8	3.36	0.70
6	-2	8	2.83	0.52
3	-6	-8	3.41	0.80
3	3	-8	3.53	0.68
-3	-3	8	3.85	0.73
6	-3	8	4.83	1.00
4	2	-8	3.61	0.74
2	-6	-8	3.60	0.91
6	-4	8	6.25	1.35
5	1	-8	8.33	1.56
1	-6	-8	7.72	1.41
0	-6	-8	8.82	1.63
6	0	-8	10.86	2.09
7	-1	-8	2.64	1.20
-1	-6	-8	3.25	1.34
6	-7	-8	7.04	1.07
7	-1	8	4.28	0.94
-1	-6	8	6.14	0.96
5	-7	-8	5.36	0.88
2	5	-8	3.08	0.77
-2	-5	8	4.90	0.89
7	-2	8	4.10	0.95
4	-7	-8	4.53	0.81
3	4	-8	3.13	0.74
7	-3	8	4.25	0.87
-3	-4	8	4.88	0.91
4	3	-8	4.66	0.89
3	-7	-8	6.22	1.17
7	-4	8	4.68	0.87
7	-4	8	7.32	1.45
7	-5	8	11.15	2.08
7	-5	8	10.45	1.62
6	1	-8	3.31	1.10
1	-7	-8	4.56	1.50
0	0	9	0.03	0.25
-1	0	-9	0.88	0.47
1	-1	-9	0.33	0.50
1	0	9	0.35	0.30
0	-1	9	0.91	0.34

-4	1	0	219.34	15.42
-4	1	0	245.43	15.38
4	-1	-1	42.00	2.94
4	-1	-1	45.16	2.94
-4	1	1	37.52	2.98
-4	1	1	42.57	2.91
4	-1	-2	58.13	3.48
4	-1	-2	54.09	3.49
4	-1	-2	44.87	3.58
4	-1	-2	47.96	3.57
-4	1	2	53.33	3.43
-4	1	2	42.69	3.54
4	-1	-3	30.56	2.15
4	-1	-3	27.31	2.20
4	-1	-3	27.84	2.09
-4	1	3	28.56	2.01
-4	1	3	33.47	2.27
4	-1	-4	139.15	11.55
4	-1	-4	192.47	11.60
-4	1	4	193.10	11.72
-4	1	4	167.74	11.68
4	-1	-5	9.70	1.00
4	-1	-5	8.42	0.91
-4	1	5	11.48	1.14
4	-1	-6	23.72	1.80
4	-1	-6	21.63	1.83
4	-1	-6	23.87	1.89
-4	1	6	30.46	2.11
4	-1	-7	1.13	0.34
4	-1	-7	1.14	0.50
-4	1	7	1.77	0.49
-4	1	7	2.22	0.66
4	-1	-8	12.79	1.21
-4	1	8	15.49	1.33
4	-1	-9	0.38	0.93
4	-1	-10	2.93	1.26
-5	2	-9	0.13	0.66
-5	2	-8	10.48	1.48
5	-2	7	0.08	0.30
-5	2	-7	-0.03	0.51
-5	2	-7	-0.35	0.58
5	-2	6	16.80	1.28
-5	2	-6	18.15	1.42
-5	2	-6	13.14	1.61
5	-2	5	0.47	0.34
5	-2	5	0.75	0.36
-5	2	-5	0.89	0.35
-5	2	-5	0.44	0.50
5	-2	4	89.93	5.97
5	-2	4	83.03	5.99
-5	2	-4	83.60	6.08
-5	2	-4	92.29	5.93
-5	2	-4	89.33	5.96
5	-2	3	4.33	0.56
5	-2	3	3.06	0.47
-5	2	-3	3.59	0.51
-5	2	-3	2.84	0.61
-5	2	-3	3.89	0.44
5	-2	2	50.67	3.67
-5	2	-2	54.28	3.67
-5	2	-2	53.15	3.60
-5	2	-2	51.46	3.72
5	-2	1	5.04	0.74
5	-2	1	5.71	0.62
-5	2	-1	5.21	0.82
-5	2	-1	4.05	0.58
-5	2	-1	5.50	0.76
-5	2	-1	5.36	0.71
5	-2	0	158.82	11.20
5	-2	0	174.73	11.21
5	-2	0	164.66	11.24
5	-2	0	175.33	11.32
-5	2	0	172.79	11.21
-5	2	0	164.55	11.28
-5	2	0	171.00	11.22
-5	2	0	153.37	11.20
5	-2	-1	5.01	0.68
5	-2	-1	5.40	0.61
5	-2	-1	5.54	0.64
-5	2	1	5.49	0.68
-5	2	1	4.82	0.55
-5	2	1	4.72	0.69
5	-2	-2	35.73	2.50
5	-2	-2	29.53	2.32
-5	2	2	34.20	2.50
5	-2	-3	2.32	0.66
5	-2	-3	3.75	0.62
5	-2	-3	4.56	0.61
5	-2	-3	3.33	0.47
-5	2	3	4.81	0.72
-5	2	3	3.25	0.53
5	-2	-4	98.23	5.95
5	-2	-4	92.08	5.98
5	-2	-4	89.07	5.94
5	-2	-4	84.38	6.09
-5	2	4	81.88	5.89
-5	2	4	80.00	6.09
5	-2	-5	0.89	0.44
5	-2	-5	0.77	0.41
5	-2	-5	1.12	0.32
5	-2	-5	1.54	0.37
-5	2	5	0.87	0.22
-5	2	5	1.36	0.63
-5	2	5	0.95	0.56
5	-2	-6	19.04	1.49
5	-2	-6	20.73	1.60
5	-2	-6	18.17	1.57
5	-2	-7	0.05	0.32
5	-2	-7	0.24	0.27
5	-2	-7	0.27	0.45
-5	2	7	0.59	0.77
-5	2	7	0.68	0.51
5	-2	-8	17.07	1.62
5	-2	-8	14.19	1.23
5	-2	-9	-0.14	0.44
5	-2	-9	0.14	0.90
-6	3	-9	-0.65	0.64

-1	1	9	0.19	0.29
0	-1	-9	1.05	0.53
0	1	9	0.51	0.27
-1	0	9	0.35	0.28
1	-1	9	0.45	0.30
-1	-1	-9	0.72	0.57
1	1	9	-0.07	0.16
-2	1	9	0.00	0.28
1	-2	9	0.03	0.32
-2	-1	-9	0.89	0.77
-3	2	9	0.46	0.30
1	-3	9	0.45	0.35
-2	0	-9	4.25	0.74
2	-2	-9	2.78	0.69
2	0	9	2.54	0.51
-2	1	-9	0.37	0.44
1	-2	-9	0.15	0.53
-1	-1	9	0.04	0.31
2	-1	9	0.17	0.29
2	0	-9	3.63	0.61
0	-2	-9	3.74	0.86
2	-2	9	2.67	0.73
-2	0	9	3.73	0.68
-1	-2	-9	-0.41	0.73
3	-1	-9	0.65	0.27
-3	1	9	0.34	0.34
2	-3	9	0.44	0.37
-2	-2	-9	0.81	1.01
2	-4	9	0.20	0.37
-4	2	9	0.00	0.32
-3	-2	-9	0.55	1.15
2	-5	9	0.47	0.37
-3	-1	-9	1.29	0.90
1	-4	9	0.53	0.34
3	-3	-9	-0.24	0.55
-3	0	-9	0.73	0.68
3	0	9	0.28	0.26
0	-3	9	-0.02	0.29
2	-3	-9	1.24	0.56
-3	1	-9	0.72	0.45
3	-1	9	0.89	0.34
-1	-2	9	0.31	0.30
2	1	-9	0.67	0.32
1	-3	-9	0.97	0.70
3	-2	9	0.59	0.35
-2	-1	9	0.75	0.36
3	0	-9	0.22	0.30
0	-3	-9	0.01	0.77
-3	0	9	0.09	0.31
3	-3	9	0.13	0.34
4	-1	-9	0.31	0.35
-1	-3	-9	0.78	1.07
3	-4	9	0.73	0.39
-2	-3	-9	-0.89	1.12
3	-5	9	0.25	0.42
-3	-3	-9	-1.62	1.40
3	-6	9	-0.16	0.41
-4	-2	-9	-0.08	1.24
2	-6	9	1.04	0.45
-4	-1	-9	-1.72	1.12
1	-5	9	0.35	0.33
-4	0	-9	3.99	1.03
-4	1	-9	0.13	0.56
3	-4	-9	0.11	0.67
-1	-3	9	0.19	0.29
4	-1	9	0.37	0.37
2	-4	-9	0.35	0.68
-4	2	-9	-0.52	0.44
4	-2	9	0.53	0.40
-2	-2	9	-0.12	0.30
3	1	-9	0.36	0.36
1	-4	-9	-0.19	0.77
-3	-1	9	0.30	0.31
4	0	-9	1.12	0.43
0	-4	-9	1.30	1.03
4	-4	9	1.72	0.50
-1	-4	-9	0.02	1.26
4	-5	9	0.61	0.44
-2	-4	-9	-1.00	1.44
4	-6	9	0.75	0.48
-5	0	-9	0.32	1.08
0	-5	9	-0.12	0.32
4	-5	-9	0.39	0.70
-5	1	-9	0.32	0.74
-1	-4	9	0.13	0.27
5	-1	9	0.37	0.47
3	-5	-9	-0.15	0.66
-5	2	-9	0.56	0.54
2	3	-9	0.60	0.39
5	-2	9	0.17	0.44
-2	-3	9	0.10	0.32
3	2	-9	0.34	0.44
2	-5	-9	-0.58	0.81
5	-3	9	-0.20	0.46
5	-3	9	-0.13	0.61
-3	-2	9	0.45	0.35
4	1	-9	-0.03	0.42
1	-5	-9	0.36	1.06
5	-4	9	0.69	0.50
0	-5	-9	-0.09	1.31
5	0	-9	-0.07	0.51
5	-5	9	0.13	0.49
4	-6	-9	1.54	0.90
2	4	-9	0.86	0.51
6	-2	-9	-0.54	0.78
-2	-4	9	0.93	0.39
3	-6	-9	-0.64	0.88
3	3	-9	1.26	0.54
-3	-3	9	0.19	0.38
6	-3	9	0.19	0.58
2	-6	-9	-0.09	1.17
4	2	-9	0.24	0.56
6	-4	9	0.38	0.75
6	-4	9	1.32	0.63
-4	-2	9	0.58	0.43
0	0	10	2.34	0.49
-1	0	-10	11.24	1.45

6	-3	8	2.96	0.65
-6	3	-8	3.01	0.78
6	-3	7	-0.36	0.49
-6	3	-7	-0.58	0.55
6	-3	6	41.69	2.83
6	-3	6	36.96	2.84
-6	3	-6	37.71	2.98
6	-3	5	-0.03	0.31
6	-3	5	-0.17	0.49
-6	3	-5	0.54	0.49
6	-3	4	5.93	0.71
-6	3	-4	4.28	0.67
6	-3	3	0.32	0.49
6	-3	3	0.20	0.30
-6	3	-3	-0.35	0.38
-6	3	-3	0.13	0.26
6	-3	2	206.10	13.14
6	-3	2	176.71	13.16
-6	3	-2	187.47	13.11
-6	3	-2	209.25	13.20
-6	3	-2	199.43	13.18
6	-3	1	-0.43	0.56
6	-3	1	-0.18	0.26
6	-3	1	-0.05	0.42
-6	3	-1	0.42	0.36
-6	3	-1	-0.12	0.37
-6	3	-1	0.06	0.42
-6	3	-1	-0.67	0.47
6	-3	0	7.35	0.84
6	-3	0	7.60	1.01
6	-3	0	5.91	0.74
6	-3	0	4.63	0.67
-6	3	0	5.18	0.69
-6	3	0	5.07	0.71
6	-3	-1	0.09	0.27
6	-3	-1	-0.17	0.31
6	-3	-1	0.12	0.50
6	-3	-1	0.83	0.34
-6	3	1	0.71	0.46
-6	3	1	0.14	0.42
-6	3	1	0.15	0.17
6	-3	-2	202.12	13.69
6	-3	-2	188.62	13.69
6	-3	-2	221.99	13.81
6	-3	-3	-0.17	0.28
6	-3	-3	-0.06	0.25
6	-3	-3	-0.02	0.33
6	-3	-3	-0.18	0.57
-6	3	3	-0.09	0.37
-6	3	3	-0.07	0.53
6	-3	-4	6.68	0.76
6	-3	-4	6.76	0.73
6	-3	-4	6.45	0.73
6	-3	-4	4.34	0.86
-6	3	4	7.36	0.98
-6	3	4	7.53	0.70
6	-3	-5	0.09	0.32
6	-3	-5	0.39	0.39
6	-3	-5	0.27	0.43
6	-3	-5	0.03	0.28
-6	3	5	0.39	0.63
-6	3	5	0.13	0.28
-6	3	5	-0.61	0.48
6	-3	-6	42.12	2.99
6	-3	-6	38.56	3.17
6	-3	-6	40.83	3.01
-6	3	6	49.97	3.30
6	-3	-7	-0.18	0.38
6	-3	-7	0.21	0.28
6	-3	-7	0.28	0.43
-6	3	7	0.48	0.58
-6	3	7	-0.19	0.79
6	-3	-8	4.51	0.90
6	-3	-8	3.60	0.52
-6	3	8	5.70	1.05
6	-3	-9	0.74	0.91
7	-4	8	7.43	1.14
7	-4	7	0.86	0.77
7	-4	6	13.15	1.23
7	-4	5	2.65	0.83
7	-4	4	24.87	1.72
-7	4	-4	19.80	1.58
7	-4	3	2.87	0.54
7	-4	3	3.03	0.47
-7	4	-3	3.40	0.50
7	-4	2	35.94	2.84
7	-4	2	38.89	2.76
-7	4	-2	39.38	2.83
-7	4	-2	40.04	2.80
7	-4	1	4.98	0.68
7	-4	1	7.04	0.97
7	-4	1	5.50	0.95
-7	4	-1	5.70	0.76
-7	4	-1	6.08	0.94
7	-4	0	49.40	3.77
7	-4	0	53.93	3.64
7	-4	0	53.51	3.83
-7	4	0	53.75	3.74
-7	4	0	48.78	3.85
7	-4	-1	6.36	1.08
7	-4	-1	5.22	0.68
7	-4	-1	5.45	0.70
7	-4	-2	36.35	2.44
-7	4	2	30.43	2.61
-7	4	2	32.99	2.50
7	-4	-3	3.45	0.45
7	-4	-3	3.48	0.50
-7	4	3	4.82	0.69
-7	4	3	2.70	0.72
7	-4	-4	26.41	2.00
7	-4	-4	27.66	1.95
-7	4	4	25.44	2.34
7	-4	-5	1.47	0.41
7	-4	-5	1.68	0.44
7	-4	-5	0.58	0.57
-7	4	5	0.83	0.79
-7	4	5	1.92	0.50

0	-1	10	8.69	1.37
0	-1	-10	8.02	1.50
1	-1	10	7.68	1.22
-1	0	10	7.87	1.18
-1	-1	-10	13.49	1.91
1	-2	10	13.16	1.56
-2	-1	-10	6.73	1.24
1	-3	10	7.72	1.25
-2	0	-10	14.30	1.78
0	-2	10	13.29	1.58
1	-2	-10	14.11	1.93
-1	-1	10	12.51	1.51
2	-1	10	13.12	1.50
0	-2	-10	13.15	1.94
-2	0	10	13.42	1.67
2	-2	10	11.74	1.55
-1	-2	-10	5.70	1.26
2	-3	10	7.96	1.57
-2	-2	-10	1.40	1.34
2	-4	10	1.56	0.54
-3	-1	-10	4.75	1.35
1	-4	10	3.54	0.69
-3	0	-10	12.82	1.78
0	-3	10	10.45	1.41
2	-3	-10	5.98	1.24
-3	1	-10	9.08	1.38
-1	-2	10	7.14	1.20
1	-3	-10	4.50	1.31
-2	-1	10	6.48	1.09
3	-2	10	8.92	1.32
0	-3	-10	7.28	1.57
3	-3	10	10.35	1.39
-3	0	10	7.87	1.23
-1	-3	-10	4.75	1.67
3	-4	10	3.52	0.70
-4	1	10	2.14	0.88
3	-4	-10	4.03	1.19
-1	-3	10	2.49	0.54
2	-4	-10	1.62	1.11
4	-2	10	1.77	0.55
-2	-2	10	2.03	0.48
1	-4	-10	4.06	1.36
-3	-1	10	2.18	0.54
0	0	0	0.00	0.00

;

_shelx_hkl_checksum 21257

7	-4	-6	12.91	1.52
7	-4	-6	15.89	1.28
-7	4	6	14.98	1.92
7	-4	-7	0.43	0.49
-7	4	7	0.62	0.86
7	-4	-8	5.99	0.96
8	-5	7	0.89	1.04
8	-5	6	3.80	0.62
8	-5	5	6.63	1.20
8	-5	4	25.68	1.99
8	-5	4	30.94	2.10
-8	5	-4	22.27	1.91
8	-5	3	6.05	0.76
8	-5	3	5.48	0.89
-8	5	-3	6.50	0.87
8	-5	2	8.75	1.06
8	-5	2	7.75	0.87
-8	5	-2	7.96	1.02
-8	5	-2	9.65	1.07
8	-5	1	11.82	1.09
8	-5	1	16.25	1.66
8	-5	1	11.85	1.33
-8	5	-1	12.09	1.34
8	-5	0	62.94	4.40
8	-5	0	52.52	4.15
8	-5	0	64.24	4.32
-8	5	0	60.86	4.27
8	-5	-1	11.64	1.04
8	-5	-1	13.60	1.47
-8	5	1	10.52	1.35
-8	5	1	10.70	1.26
8	-5	-2	8.07	0.82
-8	5	2	6.63	1.02
-8	5	2	7.91	1.22
8	-5	-3	5.86	0.76
-8	5	3	6.19	1.03
-8	5	3	6.41	0.88
8	-5	-4	26.99	2.29
-8	5	4	30.29	2.72
-8	5	4	37.23	2.57
8	-5	-5	1.32	0.65
8	-5	-5	2.13	0.45
-8	5	5	3.39	0.76
-8	5	5	2.41	0.90
-8	5	6	6.76	0.95
8	-5	-7	0.53	0.71
-8	5	7	0.55	0.67
9	-6	6	21.27	2.67
9	-6	5	0.48	0.52
9	-6	5	0.11	0.39
9	-6	5	0.56	0.97
9	-6	4	7.23	0.90
9	-6	4	5.65	0.96
9	-6	3	0.03	0.36
9	-6	3	-0.24	1.06
9	-6	3	0.05	0.50
-9	6	-3	0.56	0.42
9	-6	2	23.01	1.89
9	-6	2	25.66	2.07
9	-6	1	-0.08	0.65
9	-6	1	0.12	0.58
9	-6	1	0.24	0.33
-9	6	-1	0.06	0.46
9	-6	0	5.99	0.76
9	-6	0	5.75	0.99
-9	6	0	6.23	0.88
9	-6	-1	0.50	0.63
9	-6	-1	0.42	0.33
-9	6	1	-0.32	0.53
9	-6	-2	23.33	1.79
-9	6	2	22.00	2.09
9	-6	-3	-0.05	0.33
9	-6	-3	0.71	0.85
-9	6	3	-0.45	0.53
9	-6	-4	3.89	0.57
-9	6	4	4.04	0.93
9	-6	-5	0.30	0.36
-9	6	5	-1.64	0.77
-9	6	6	8.79	1.22
10	-7	5	2.01	1.52
10	-7	5	0.83	0.47
10	-7	4	8.75	1.13
10	-7	4	6.37	0.99
10	-7	3	1.93	0.67
10	-7	3	5.77	1.45
10	-7	3	1.49	0.47
10	-7	2	5.98	1.21
10	-7	2	4.25	0.86
10	-7	1	3.15	0.58
10	-7	1	4.35	0.82
10	-7	0	13.64	1.62
10	-7	0	12.18	1.21
-10	7	0	12.62	1.47
10	-7	-1	5.37	0.97
10	-7	-1	3.56	0.58
-10	7	1	2.95	0.90
10	-7	-2	3.57	0.57
-10	7	2	2.88	0.98
10	-7	-3	1.96	0.47
-10	7	3	1.03	0.96
10	-7	-4	7.60	0.93
-10	7	4	6.82	1.08
10	-7	-5	1.24	0.51
-10	7	5	-0.57	0.95
11	-8	2	4.54	1.55
11	-8	1	0.18	0.40
11	-8	1	0.19	0.68
11	-8	0	9.68	1.32
11	-8	0	7.40	1.00
-11	8	0	6.67	1.17
11	-8	-1	0.76	0.74
11	-8	-1	0.67	0.42
-11	8	1	-0.15	0.85
11	-8	-2	3.81	1.02
11	-8	-2	1.61	0.50
-11	8	2	0.38	0.97
-7	11	3	0.56	0.62

7	-11	-3	0.11	0.32
-7	11	2	3.00	0.75
7	-11	-2	2.50	0.47
-7	11	1	-0.39	0.63
7	-11	-1	-0.21	0.33
7	-11	0	6.62	0.83
7	-11	1	0.17	0.35
7	-11	2	3.62	0.93
7	-11	2	2.65	0.47
7	-11	3	0.16	0.86
7	-11	3	0.02	0.36
-6	10	5	1.28	0.50
6	-10	-5	0.86	0.35
-6	10	4	2.19	0.54
6	-10	-4	2.01	0.40
-6	10	3	3.00	0.64
6	-10	-3	3.11	0.45
-6	10	2	20.19	1.73
6	-10	-2	19.88	1.54
-6	10	1	6.32	0.93
6	-10	-1	5.32	0.70
-6	10	0	3.00	0.53
6	-10	0	2.04	0.40
6	-10	1	5.36	0.81
6	-10	1	5.27	0.65
6	-10	2	23.17	2.01
6	-10	2	18.42	1.53
6	-10	3	3.36	0.86
6	-10	3	3.03	0.46
6	-10	4	1.98	0.85
6	-10	4	1.84	0.40
6	-10	5	0.93	0.66
6	-10	5	1.09	0.38
-5	9	6	3.78	0.68
5	-9	-6	4.23	0.84
-5	9	5	0.30	0.47
5	-9	-5	-0.22	0.25
-5	9	4	13.80	1.19
5	-9	-4	13.31	1.10
-5	9	3	0.26	0.37
5	-9	-3	0.12	0.24
-5	9	2	11.34	1.08
5	-9	-2	12.27	1.09
5	-9	-2	11.77	0.97
-5	9	1	-0.24	0.32
5	-9	-1	-0.04	0.28
5	-9	-1	0.69	0.26
-5	9	0	23.35	1.75
5	-9	0	20.91	1.71
5	-9	0	22.24	1.67
5	-9	0	25.77	1.93
-5	9	-1	0.12	0.43
5	-9	1	-0.03	0.24
5	-9	1	-0.16	0.47
5	-9	1	0.01	0.24
5	-9	2	12.76	1.30
5	-9	2	11.28	0.97
5	-9	2	11.88	1.02
5	-9	3	0.27	0.56
5	-9	3	-0.10	0.26
5	-9	4	13.39	1.12
5	-9	4	14.57	1.41
5	-9	5	-0.07	0.28
5	-9	5	-0.09	0.66
5	-9	6	4.75	0.97
-4	8	7	-0.01	0.43
4	-8	-7	0.28	0.82
-4	8	6	1.15	0.44
4	-8	-6	2.10	0.68
-4	8	5	0.22	0.38
4	-8	-5	0.05	0.22
-4	8	4	63.57	4.29
4	-8	-4	60.85	4.23
-4	8	3	-0.10	0.26
4	-8	-3	-0.15	0.28
4	-8	-3	-0.08	0.20
4	-8	-2	0.60	0.29
4	-8	-2	0.58	0.33
4	-8	-2	0.25	0.20
-4	8	1	-0.21	0.27
4	-8	-1	-0.10	0.24
4	-8	-1	-0.24	0.55
4	-8	-1	0.00	0.19
-4	8	0	139.06	9.03
4	-8	0	136.92	9.13
4	-8	0	125.48	8.98
4	-8	0	134.83	9.02
-4	8	-1	0.83	0.32
4	-8	1	0.00	0.40
4	-8	2	0.73	0.23
4	-8	2	0.57	0.26
4	-8	2	0.53	0.41
4	-8	3	0.07	0.21
4	-8	3	-0.13	0.24
4	-8	3	-0.42	0.36
4	-8	4	63.39	4.30
4	-8	4	63.18	4.29
4	-8	4	61.78	4.41
4	-8	5	0.22	0.57
4	-8	5	0.25	0.25
4	-8	5	-0.11	0.25
4	-8	6	1.22	0.37
4	-8	6	1.76	0.67
4	-8	7	0.41	0.74
3	-7	-8	4.03	1.08
-3	7	7	0.66	0.35
3	-7	-7	-0.14	0.78
-3	7	6	15.61	1.24
3	-7	-6	14.61	1.16
-3	7	5	1.46	0.35
3	-7	-5	1.47	0.25
-3	7	4	27.49	2.05
3	-7	-4	29.69	2.00
-3	7	3	4.36	0.65
-3	7	3	4.38	0.53
3	-7	-3	3.71	0.50
3	-7	-3	3.40	0.41

-3	7	2	43.00	2.94
3	-7	-2	41.55	2.89
3	-7	-2	41.05	2.91
3	-7	-2	38.92	2.82
-3	7	1	5.98	0.65
-3	7	1	5.20	0.72
3	-7	-1	5.46	0.50
3	-7	-1	5.53	0.61
3	-7	-1	5.28	0.64
3	-7	-1	5.09	0.75
-3	7	0	44.88	3.61
-3	7	0	53.99	3.63
3	-7	0	58.25	3.63
3	-7	0	54.52	3.58
3	-7	0	51.73	3.73
-3	7	-1	6.26	0.73
3	-7	1	5.29	0.59
3	-7	1	5.86	0.52
3	-7	1	4.82	0.67
3	-7	2	39.23	2.77
3	-7	2	40.68	2.74
3	-7	3	3.74	0.55
3	-7	3	3.43	0.50
3	-7	3	4.45	0.43
3	-7	4	28.60	2.17
3	-7	4	27.97	2.03
3	-7	4	29.28	2.01
-3	7	-5	1.64	0.31
3	-7	5	1.59	0.29
3	-7	5	1.43	0.50
3	-7	5	1.37	0.29
-3	7	-6	15.13	1.18
3	-7	6	14.96	1.19
3	-7	6	16.82	1.50
-3	7	-7	0.44	0.25
3	-7	7	1.04	0.62
-3	7	-8	4.73	0.58
2	-6	-9	0.34	1.14
2	-6	-8	3.89	1.10
-2	6	7	4.91	0.58
2	-6	-7	3.45	0.86
-2	6	6	44.65	3.02
2	-6	-6	43.04	2.99
-2	6	5	20.47	1.46
2	-6	-5	19.71	1.42
-2	6	4	9.59	0.98
-2	6	4	8.65	0.75
2	-6	-4	8.18	0.68
-2	6	3	52.66	3.56
-2	6	3	52.12	3.70
2	-6	-3	52.63	3.54
2	-6	-3	47.61	3.59
2	-6	-3	56.70	3.63
-2	6	2	170.27	11.59
-2	6	2	175.22	11.55
2	-6	-2	175.35	11.47
2	-6	-2	178.26	11.52
2	-6	-2	160.65	11.53
-2	6	1	92.71	6.36
-2	6	1	94.70	6.26
-2	6	1	89.57	6.27
2	-6	-1	91.38	6.36
2	-6	-1	98.27	6.29
2	-6	-1	89.49	6.24
2	-6	-1	95.28	6.28
-2	6	0	7.94	0.69
-2	6	0	8.27	0.72
-2	6	0	7.70	0.80
-2	6	0	7.72	0.64
2	-6	0	8.10	0.62
2	-6	0	7.83	0.91
2	-6	0	7.65	0.70
2	-6	0	7.72	0.68
-2	6	-1	90.76	6.29
-2	6	-1	93.49	6.25
-2	6	-1	93.38	6.40
2	-6	1	92.84	6.33
2	-6	1	91.93	6.27
2	-6	1	94.51	6.29
2	-6	1	95.25	6.25
-2	6	-2	146.16	11.07
-2	6	-2	167.40	11.05
2	-6	2	168.48	11.08
2	-6	2	177.53	11.05
2	-6	2	168.34	11.08
-2	6	-3	55.66	3.67
2	-6	3	53.99	3.66
2	-6	3	51.53	3.73
2	-6	3	55.26	3.69
-2	6	-4	8.14	0.67
2	-6	4	8.26	0.86
2	-6	4	8.17	0.66
2	-6	4	8.56	0.72
-2	6	-5	19.68	1.37
2	-6	5	17.94	1.40
2	-6	5	20.18	1.53
2	-6	5	18.91	1.36
-2	6	-6	44.27	2.96
2	-6	6	46.43	3.11
2	-6	6	39.61	2.99
-2	6	-7	4.68	0.51
2	-6	7	4.97	0.57
2	-6	7	6.83	1.01
-2	6	-8	3.87	0.48
-2	6	-9	0.53	0.24
1	-5	-9	-0.37	0.60
1	-5	-8	5.79	1.01
1	-5	-7	-0.01	0.55
1	-5	-7	0.11	0.23
-1	5	6	48.12	3.11
-1	5	6	39.13	3.46
1	-5	-6	46.02	3.12
1	-5	-6	48.47	3.10
-1	5	5	0.59	0.46
-1	5	5	0.69	0.21
1	-5	-5	0.54	0.19
1	-5	-5	0.48	0.16
-1	5	4	15.76	1.23
-1	5	4	17.19	1.23
-1	5	4	17.90	1.58
1	-5	-4	18.16	1.34
1	-5	-4	17.26	1.21
-1	5	3	1.07	0.42
-1	5	3	0.91	0.20
-1	5	3	0.86	0.17
1	-5	-3	1.02	0.19
1	-5	-3	0.69	0.23
1	-5	-3	0.68	0.17
1	-5	-3	0.80	0.23
-1	5	2	199.03	13.15
-1	5	2	196.38	13.00
-1	5	2	199.01	12.99
1	-5	-2	194.40	12.98
1	-5	-2	192.61	13.02
1	-5	-2	205.21	13.02
1	-5	-2	195.77	12.98
1	-5	-2	176.52	12.96
-1	5	1	1.54	0.23
-1	5	1	1.36	0.23
-1	5	1	1.53	0.38
-1	5	1	1.33	0.21
1	-5	-1	1.53	0.26
1	-5	-1	1.35	0.23
-1	5	0	25.13	1.72
-1	5	0	24.57	1.89
-1	5	0	24.23	1.75
1	-5	0	23.32	1.75
1	-5	0	25.89	1.73
1	-5	0	25.01	1.77
1	-5	0	26.29	1.73
1	-5	0	25.11	1.84
-1	5	-1	1.32	0.19
-1	5	-1	1.29	0.35
-1	5	-1	1.28	0.28
1	-5	1	1.26	0.19
1	-5	1	1.74	0.31
1	-5	1	1.54	0.24
1	-5	1	1.19	0.22
-1	5	-2	201.12	12.80
-1	5	-2	191.19	12.62
-1	5	-2	159.79	12.62
1	-5	2	197.59	12.66
1	-5	2	200.05	12.65
1	-5	2	181.62	12.65
1	-5	2	194.77	12.62
-1	5	-3	0.77	0.15
1	-5	3	0.78	0.27
1	-5	3	0.84	0.21
1	-5	3	0.90	0.17
-1	5	-4	17.31	1.24
1	-5	4	17.22	1.32
1	-5	4	17.97	1.24
1	-5	4	17.92	1.29
-1	5	-5	0.64	0.17
1	-5	5	0.34	0.16
1	-5	5	0.40	0.20
1	-5	5	0.69	0.32
-1	5	-6	46.84	3.12
1	-5	6	43.98	3.16
1	-5	6	47.26	3.20
-1	5	-7	-0.02	0.16
1	-5	7	0.41	0.22
-1	5	-8	6.31	0.57
-1	5	-9	0.42	0.21
0	-4	-9	0.25	0.73
0	-4	-8	5.64	0.55
0	-4	-8	4.97	0.88
0	4	7	7.49	1.52
0	-4	-7	10.15	0.77
0	-4	-7	9.10	1.21
0	4	6	69.73	4.70
0	-4	-6	68.95	4.71
0	-4	-6	71.71	4.74
0	4	5	45.72	3.11
0	-4	-5	46.55	3.14
0	4	4	14.08	0.96
0	4	4	13.14	0.97
0	4	4	12.84	1.08
0	-4	-4	14.57	1.07
0	4	3	121.17	7.62
0	4	3	99.25	7.82
0	4	3	115.55	7.60
0	-4	-3	105.40	7.61
0	-4	-3	121.27	7.65
0	-4	-3	120.26	7.62
0	4	2	300.60	20.06
0	4	2	322.33	20.08
0	4	2	290.75	20.26
0	-4	-2	290.17	20.09
0	-4	-2	298.27	20.08
0	-4	-2	326.16	20.08
0	-4	-2	283.75	20.08
0	4	1	227.86	15.00
0	4	1	223.97	14.82
0	4	1	220.05	14.82
0	-4	-1	226.51	14.82
0	-4	-1	227.90	14.84
0	-4	-1	217.11	14.83
0	-4	-1	214.33	14.88
0	4	0	8.35	0.88
0	4	0	7.84	0.66
0	4	0	8.44	0.62
0	-4	0	8.25	0.64
0	-4	0	8.24	0.62
0	-4	0	8.12	0.74
0	4	-1	237.30	15.53
0	4	-1	213.17	15.36
0	-4	1	245.33	15.40
0	-4	1	227.64	15.38
0	4	-2	300.08	19.71
0	4	-2	287.90	19.53
0	-4	2	292.89	19.55
0	-4	2	294.07	19.55
0	4	-3	114.68	7.65

0	-4	3	122.83	7.69
0	-4	3	106.62	7.67
0	4	-4	12.65	0.97
0	-4	4	15.67	0.97
0	-4	4	12.76	1.03
0	-4	4	13.35	1.02
0	4	-5	42.33	2.96
0	-4	5	43.15	3.00
0	-4	5	47.77	2.97
0	-4	5	42.42	3.02
0	4	-6	63.94	4.41
0	-4	6	67.68	4.47
0	4	-7	9.84	0.77
0	-4	7	10.89	0.86
0	4	-8	5.78	0.51
0	4	-9	1.30	0.23
-1	-3	-10	2.20	1.53
-1	-3	-10	3.21	0.52
-1	-3	-9	0.29	0.25
-1	-3	-9	0.08	1.06
-1	-3	-8	18.46	2.01
-1	-3	-8	19.11	1.42
-1	-3	-7	1.14	0.73
-1	-3	-7	1.44	0.32
1	3	6	22.92	1.54
-1	-3	-6	21.95	1.64
1	3	5	9.06	0.65
-1	-3	-5	8.82	0.79
1	3	4	139.57	8.65
1	3	4	123.67	8.65
1	3	4	137.84	8.99
-1	-3	-4	118.77	8.69
1	3	3	31.75	2.06
1	3	3	30.50	2.07
1	3	3	32.81	2.09
1	3	3	28.45	2.09
-1	-3	-3	26.77	2.07
-1	-3	-3	31.45	2.12
-1	-3	-3	32.14	2.10
1	3	2	53.34	3.57
1	3	2	45.20	3.85
1	3	2	53.88	3.58
1	3	2	52.82	3.58
-1	-3	-2	54.81	3.61
-1	-3	-2	56.64	3.59
-1	-3	-2	54.94	3.60
1	3	1	39.32	2.98
1	3	1	46.55	2.99
1	3	1	39.42	3.22
1	3	1	43.06	2.97
-1	-3	-1	44.87	3.00
-1	-3	-1	47.86	3.02
-1	-3	-1	47.25	2.99
1	3	0	283.86	18.52
1	3	0	268.77	18.32
1	3	0	261.10	18.31
1	3	0	291.24	18.32
1	3	0	274.24	18.32
-1	-3	0	267.08	18.31
-1	-3	0	266.92	18.31
-1	-3	0	288.85	18.35
1	3	-1	51.02	3.12
1	3	-1	47.57	3.09
1	3	-1	43.01	3.12
1	3	-1	45.17	3.30
-1	-3	1	44.89	3.11
-1	-3	1	42.26	3.10
1	3	-2	60.30	3.93
1	3	-2	54.74	3.70
-1	-3	2	53.77	3.71
-1	-3	2	51.81	3.73
1	3	-3	30.93	2.02
1	3	-3	29.98	2.25
-1	-3	3	29.28	2.02
-1	-3	3	28.25	2.06
1	3	-4	129.57	8.65
-1	-3	4	129.22	8.65
-1	-3	4	130.71	8.71
1	3	-5	8.80	0.65
-1	-3	5	8.52	0.76
1	3	-6	21.12	1.50
-1	-3	6	22.45	1.60
1	3	-7	1.38	0.20
-1	-3	7	1.54	0.31
1	3	-8	18.39	2.30
1	3	-8	18.02	1.30
-1	-3	8	18.92	1.42
1	3	-9	0.31	0.19
1	3	-9	-2.32	1.43
-2	-2	-10	1.30	0.55
-2	-2	-10	2.08	1.45
-2	-2	-9	1.11	0.40
-2	-2	-9	0.12	1.03
-2	-2	-8	43.06	2.98
-2	-2	-8	38.75	3.26
-2	-2	-7	-0.50	0.63
-2	-2	-7	-0.09	0.39
-2	-2	-6	2.26	0.49
2	2	5	0.06	0.11
2	2	5	-0.17	0.29
-2	-2	-5	-0.04	0.35
2	2	4	357.51	24.21
2	2	4	402.88	24.25
2	2	4	357.07	24.28
2	2	4	396.25	24.20
-2	-2	-4	308.93	24.21
2	2	3	0.02	0.24
2	2	3	-0.02	0.12
2	2	3	0.11	0.15
2	2	3	0.10	0.09
-2	-2	-3	0.13	0.18
-2	-2	-3	-0.05	0.16
-2	-2	-3	0.04	0.11
-2	-2	-3	-0.10	0.26
2	2	2	41.92	2.74
2	2	2	43.29	2.76
2	2	2	42.34	2.77

2	2	2	36.96	2.84
2	2	2	35.44	2.82
-2	-2	-2	41.66	2.82
-2	-2	-2	41.23	2.78
-2	-2	-2	41.20	2.75
2	2	1	-0.34	0.44
2	2	1	0.00	0.10
2	2	1	0.31	0.20
2	2	1	0.35	0.22
2	2	1	0.16	0.15
2	2	1	-0.05	0.14
-2	-2	-1	-0.01	0.17
-2	-2	-1	0.08	0.12
-2	-2	-1	0.08	0.18
2	2	0	988.93	65.15
2	2	0	995.71	65.05
2	2	0	987.77	65.24
2	2	0	979.36	65.27
2	2	0	976.23	65.12
2	2	0	940.62	65.07
-2	-2	0	972.40	65.15
-2	-2	0	989.53	65.07
-2	-2	0	985.48	65.05
2	2	-1	-0.03	0.18
2	2	-1	0.13	0.14
2	2	-1	0.10	0.15
2	2	-1	0.47	0.39
-2	-2	1	-0.12	0.16
-2	-2	1	0.19	0.17
2	2	-2	40.87	2.78
2	2	-2	39.04	2.79
2	2	-2	40.60	2.84
2	2	-2	44.52	3.04
-2	-2	2	40.26	2.78
-2	-2	2	41.22	2.84
2	2	-3	0.03	0.13
2	2	-3	0.01	0.32
2	2	-3	0.05	0.20
-2	-2	3	0.35	0.12
-2	-2	3	-0.02	0.21
2	2	-4	398.19	25.97
-2	-2	4	383.28	26.04
2	2	-5	0.06	0.14
-2	-2	5	-0.01	0.27
2	2	-6	0.81	0.18
2	2	-6	0.46	0.62
-2	-2	6	0.76	0.31
2	2	-7	-0.36	0.66
2	2	-7	-0.01	0.17
-2	-2	7	0.06	0.25
2	2	-8	36.84	2.76
2	2	-8	44.11	3.20
-2	-2	8	42.28	2.87
2	2	-9	-0.65	0.87
2	2	-9	0.26	0.25
2	2	-10	1.55	1.17
-3	-1	-10	2.65	1.48
-3	-1	-9	0.06	1.02
-3	-1	-9	-0.45	0.46
-3	-1	-8	27.44	2.22
-3	-1	-7	1.00	0.64
-3	-1	-7	3.75	0.70
3	1	6	23.00	1.61
3	1	6	22.82	1.54
-3	-1	-6	20.93	1.90
3	1	5	9.28	0.69
3	1	5	10.54	0.80
3	1	5	8.15	0.87
3	1	4	130.80	8.94
3	1	4	131.66	8.85
3	1	4	135.68	8.91
3	1	3	28.27	2.15
3	1	3	29.44	2.10
3	1	3	31.73	2.03
-3	-1	-3	28.64	2.00
-3	-1	-3	26.02	2.05
-3	-1	-3	34.53	2.09
-3	-1	-3	28.64	2.02
3	1	2	56.51	3.58
3	1	2	49.65	3.52
3	1	2	56.43	3.52
-3	-1	-2	45.43	3.53
-3	-1	-2	51.02	3.50
-3	-1	-2	52.35	3.59
3	1	1	45.23	3.01
3	1	1	40.99	3.03
3	1	1	43.43	3.01
3	1	1	44.05	3.05
-3	-1	-1	38.97	3.03
-3	-1	-1	51.22	3.07
-3	-1	-1	42.54	3.00
3	1	0	241.22	17.85
3	1	0	276.54	17.65
3	1	0	275.52	17.67
3	1	0	252.64	17.65
3	1	0	237.54	17.70
3	1	0	280.67	17.66
-3	-1	0	247.65	17.65
-3	-1	0	296.73	17.66
-3	-1	0	272.49	17.66
3	1	-1	46.48	3.14
3	1	-1	42.73	3.06
3	1	-1	42.20	3.29
3	1	-1	44.86	3.06
-3	-1	1	39.45	3.08
-3	-1	1	50.49	3.06
3	1	-2	56.08	3.53
3	1	-2	54.23	3.61
3	1	-2	49.56	3.53
3	1	-2	53.30	3.78
-3	-1	2	51.96	3.50
-3	-1	2	45.52	3.59
3	1	-3	27.27	1.94
3	1	-3	27.52	2.03
3	1	-3	28.35	1.94
3	1	-3	29.54	2.19
-3	-1	3	25.33	2.05

-3	-1	3	28.75	1.89
3	1	-4	129.91	8.97
3	1	-4	128.99	8.76
3	1	-4	133.30	8.85
3	1	-4	132.52	8.76
-3	-1	4	130.41	8.87
3	1	-5	8.16	1.00
3	1	-5	8.51	0.82
3	1	-5	9.70	0.73
-3	-1	5	8.56	0.92
3	1	-6	22.39	1.83
3	1	-6	21.62	1.53
-3	-1	6	20.50	1.68
3	1	-7	1.79	0.65
3	1	-7	1.20	0.29
-3	-1	7	1.57	0.40
3	1	-8	15.96	1.39
3	1	-8	19.52	1.93
-3	-1	8	19.94	1.49
3	1	-9	0.63	0.43
3	1	-9	0.39	0.99
3	1	-10	2.85	0.99
-4	0	-9	2.95	0.79
-4	0	-9	1.69	1.00
-4	0	-8	6.60	1.02
4	0	7	10.64	0.97
-4	0	-7	9.28	1.31
-4	0	-7	9.57	1.29
4	0	6	69.53	4.36
4	0	6	62.61	4.44
-4	0	-6	69.37	4.57
-4	0	-6	56.61	4.56
4	0	5	43.97	2.86
4	0	5	40.75	2.96
-4	0	-5	40.84	3.08
-4	0	-5	41.96	3.03
4	0	4	13.06	1.03
4	0	4	11.50	1.10
4	0	4	15.77	1.16
4	0	4	12.25	0.99
4	0	4	13.11	0.99
4	0	3	112.81	7.43
4	0	3	113.00	7.33
-4	0	-3	110.61	7.46
-4	0	-3	106.90	7.37
-4	0	-3	105.56	7.31
-4	0	-3	107.37	7.33
4	0	2	288.82	19.00
4	0	2	269.80	19.07
-4	0	-2	300.37	19.09
-4	0	-2	281.35	19.01
-4	0	-1	288.64	19.33
4	0	0	16.96	1.24
4	0	0	15.29	1.50
4	0	0	14.15	1.28
4	0	0	14.69	1.21
4	0	0	13.58	1.19
-4	0	0	14.07	1.15
-4	0	0	14.78	1.22
-4	0	0	13.10	1.21
-4	0	0	15.67	1.31
-4	0	0	15.37	1.23
4	0	-1	202.81	14.37
-4	0	1	223.35	14.24
4	0	-2	296.79	19.73
4	0	-2	291.01	19.92
4	0	-2	286.85	19.83
-4	0	2	284.15	19.78
-4	0	2	319.04	19.69
4	0	-3	120.45	7.52
4	0	-3	102.51	7.50
4	0	-3	106.32	7.67
4	0	-3	118.74	7.61
-4	0	3	98.93	7.56
-4	0	3	120.06	7.44
4	0	-4	12.62	1.23
4	0	-4	11.21	1.10
4	0	-4	14.70	1.29
4	0	-4	11.56	1.03
4	0	-4	13.53	1.16
-4	0	4	13.24	0.91
4	0	-5	40.81	2.76
4	0	-5	36.45	2.82
4	0	-5	40.47	2.88
4	0	-5	42.50	2.94
4	0	-6	70.55	4.54
4	0	-6	56.18	4.43
4	0	-6	64.65	4.50
-4	0	6	74.89	4.57
-4	0	6	57.21	4.56
4	0	-7	10.02	1.20
4	0	-7	10.57	1.08
-4	0	7	9.49	1.08
-4	0	7	9.75	1.35
4	0	-8	5.43	1.05
-4	0	8	5.47	0.90
-4	0	8	5.27	0.71
4	0	-9	3.11	0.68
4	0	-9	1.63	1.16
-4	0	9	0.86	1.07
-5	1	-9	-0.78	0.76
-5	1	-8	6.15	1.04
-5	1	-8	10.01	1.13
5	-1	7	0.29	0.31
5	-1	7	0.11	0.29
-5	1	-7	-0.15	0.54
-5	1	-7	0.67	0.60
5	-1	6	41.37	2.95
-5	1	-6	44.78	3.12
5	-1	5	0.63	0.32
-5	1	-5	1.27	0.50
-5	1	-5	0.31	0.39
-5	1	-5	0.13	0.43
5	-1	4	16.63	1.30
5	-1	4	15.09	1.34
-5	1	-4	17.43	1.42
5	-1	3	0.98	0.42

5	-1	3	1.52	0.40
5	-1	3	1.46	0.41
-5	1	-3	0.47	0.30
-5	1	-3	0.57	0.40
-5	1	-3	2.06	0.51
5	-1	2	189.27	12.84
-5	1	-2	186.49	12.85
-5	1	-2	207.30	12.84
-5	1	-2	204.18	12.85
-5	1	-2	166.59	12.76
5	-1	1	1.22	0.47
5	-1	1	1.63	0.38
-5	1	-1	0.77	0.39
-5	1	-1	0.87	0.41
-5	1	-1	1.02	0.48
-5	1	-1	1.57	0.40
5	-1	0	36.32	2.53
5	-1	0	38.21	2.47
5	-1	0	31.26	2.36
-5	1	0	26.84	2.36
-5	1	0	27.30	2.38
-5	1	0	36.01	2.37
-5	1	0	34.62	2.50
-5	1	0	28.33	2.39
5	-1	-1	2.32	0.59
5	-1	-1	1.15	0.38
5	-1	-1	2.26	0.65
5	-1	-1	1.57	0.39
-5	1	1	2.48	0.54
-5	1	1	2.04	0.35
-5	1	1	1.68	0.47
-5	1	1	1.08	0.39
5	-1	-2	323.59	21.76
-5	1	2	312.44	21.82
-5	1	2	340.55	21.85
5	-1	-3	0.84	0.50
5	-1	-3	0.53	0.51
-5	1	3	0.87	0.31
-5	1	3	0.88	0.48
-5	1	3	1.46	0.50
5	-1	-4	14.35	1.40
5	-1	-4	16.25	1.40
5	-1	-4	15.15	1.49
5	-1	-4	18.02	1.40
-5	1	4	16.89	1.19
5	-1	-5	0.56	0.51
5	-1	-5	2.39	0.51
5	-1	-5	0.51	0.42
5	-1	-5	0.15	0.42
5	-1	-5	0.62	0.32
-5	1	5	1.04	0.55
-5	1	5	0.58	0.20
5	-1	-6	41.47	3.06
5	-1	-6	43.14	3.12
5	-1	-7	0.43	0.55
5	-1	-7	0.67	0.39
-5	1	7	0.41	0.45
-5	1	7	0.12	0.64
5	-1	-8	6.21	1.11
5	-1	-9	0.24	1.06
-6	2	-9	0.06	0.77
6	-2	8	3.21	0.61
-6	2	-8	3.10	0.88
6	-2	7	4.45	0.66
-6	2	-7	5.57	0.92
6	-2	6	72.48	4.72
-6	2	-6	61.71	4.83
6	-2	5	28.67	2.23
-6	2	-5	30.91	2.42
6	-2	4	8.58	0.95
6	-2	4	7.18	0.90
6	-2	4	8.77	1.03
-6	2	-4	11.84	1.22
6	-2	3	52.56	3.49
6	-2	3	48.26	3.49
-6	2	-3	46.82	3.45
6	-2	2	161.75	10.90
6	-2	2	161.83	10.87
-6	2	-2	161.51	10.84
-6	2	-2	165.66	10.91
-6	2	-2	154.06	10.94
6	-2	1	87.36	6.15
6	-2	1	85.38	6.07
-6	2	-1	92.87	6.19
-6	2	-1	82.11	6.07
-6	2	-1	95.89	6.17
6	-2	0	6.43	0.78
-6	2	0	6.54	0.93
6	-2	-1	90.48	7.08
-6	2	1	118.42	7.24
6	-2	-2	176.81	11.20
6	-2	-2	146.00	11.33
6	-2	-2	164.88	11.18
-6	2	2	176.98	11.30
6	-2	-3	47.08	3.94
-6	2	3	51.35	3.84
-6	2	3	58.09	3.61
-6	2	3	52.81	3.83
6	-2	-4	8.76	0.92
6	-2	-4	5.64	0.96
6	-2	-4	8.62	0.97
-6	2	4	10.59	1.21
-6	2	4	7.27	0.79
6	-2	-5	17.56	1.68
6	-2	-5	19.75	1.51
6	-2	-5	16.59	1.49
-6	2	5	17.15	1.36
6	-2	-6	38.11	2.80
6	-2	-6	35.46	2.93
6	-2	-6	40.59	2.80
6	-2	-7	4.02	0.61
6	-2	-7	4.61	0.75
6	-2	-7	4.28	0.73
-6	2	7	7.58	1.15
-6	2	7	7.17	1.08
6	-2	-8	3.26	0.96
6	-2	-9	1.50	1.10

7	-3	8	3.69	0.81
7	-3	7	0.02	0.65
7	-3	6	17.57	1.83
7	-3	6	21.22	1.69
7	-3	5	0.96	0.42
7	-3	5	1.17	0.47
7	-3	4	22.89	1.71
7	-3	4	23.94	1.72
-7	3	-4	17.66	1.53
7	-3	3	3.72	0.61
7	-3	3	3.32	0.52
-7	3	-3	3.39	0.52
7	-3	2	37.85	2.92
7	-3	2	39.96	2.86
-7	3	-2	39.09	2.88
-7	3	-2	41.05	2.97
7	-3	1	5.85	0.78
7	-3	1	5.71	0.83
-7	3	-1	4.85	0.75
-7	3	-1	6.33	0.98
-7	3	-1	4.44	0.87
7	-3	0	57.64	3.67
7	-3	0	51.45	3.79
-7	3	0	50.32	3.85
-7	3	0	47.68	3.71
7	-3	-1	5.25	0.73
7	-3	-1	5.63	0.70
-7	3	1	4.40	0.80
7	-3	-2	94.38	6.12
-7	3	2	75.71	5.80
7	-3	-3	3.50	0.56
7	-3	-3	6.57	1.04
-7	3	3	4.53	0.76
-7	3	3	2.69	0.71
-7	3	3	5.87	0.87
7	-3	-4	24.74	1.99
7	-3	-4	26.62	1.99
-7	3	4	25.67	2.21
7	-3	-5	1.35	0.49
7	-3	-5	1.25	0.46
7	-3	-5	0.71	0.61
-7	3	5	2.12	0.79
-7	3	5	1.65	0.37
-7	3	5	1.29	0.58
7	-3	-6	14.62	1.30
7	-3	-6	13.48	1.53
-7	3	6	13.37	1.12
7	-3	-7	0.52	0.47
7	-3	-7	1.13	0.60
7	-3	-8	4.87	1.02
8	-4	7	0.14	0.85
8	-4	6	2.64	0.92
8	-4	6	0.79	0.47
8	-4	6	1.23	0.50
8	-4	5	0.01	0.45
8	-4	5	0.08	0.76
8	-4	5	-0.15	0.38
8	-4	4	58.64	3.73
8	-4	4	55.21	3.75
-8	4	-4	43.62	3.61
8	-4	3	-0.38	0.45
8	-4	3	-0.22	0.34
8	-4	3	-0.58	0.66
-8	4	-3	0.08	0.36
8	-4	2	2.12	0.78
8	-4	2	0.32	0.50
8	-4	2	0.65	0.36
-8	4	-2	0.50	0.48
-8	4	-2	0.70	0.45
8	-4	1	-0.43	0.68
8	-4	1	-0.34	0.51
8	-4	1	0.06	0.32
-8	4	-1	2.08	0.60
8	-4	0	125.09	8.61
8	-4	0	124.27	8.75
-8	4	0	131.88	8.68
-8	4	0	125.81	8.80
8	-4	-1	-0.07	0.32
8	-4	-1	-0.15	0.57
-8	4	1	-0.75	0.43
-8	4	1	-0.05	0.52
8	-4	-2	0.22	0.34
8	-4	-2	1.40	0.75
-8	4	2	0.52	0.48
-8	4	2	0.36	0.54
8	-4	-3	-0.30	0.33
8	-4	-3	0.16	0.77
-8	4	3	-0.51	0.55
-8	4	3	0.07	0.57
8	-4	-4	56.10	4.21
-8	4	4	64.08	4.42
8	-4	-5	-0.39	0.37
-8	4	5	0.08	0.55
-8	4	5	0.59	0.84
8	-4	-6	0.84	0.60
8	-4	-6	0.52	0.49
-8	4	6	1.73	0.56
8	-4	-7	-0.37	0.62
-8	4	7	-0.04	0.53
9	-5	6	2.78	0.63
9	-5	6	3.40	0.67
9	-5	5	-0.06	0.51
9	-5	5	0.69	0.85
9	-5	5	-0.09	0.40
9	-5	4	11.60	1.17
9	-5	4	11.83	1.27
9	-5	3	-0.13	0.34
9	-5	3	0.14	0.50
9	-5	3	0.75	1.00
-9	5	-3	0.13	0.42
9	-5	2	10.04	1.06
9	-5	2	10.85	1.28
9	-5	1	0.02	0.35
9	-5	1	-0.01	0.72
9	-5	1	0.73	0.63
-9	5	-1	-0.81	0.45
9	-5	0	22.10	1.97

9	-5	0	19.75	1.68
-9	5	0	21.72	1.87
9	-5	-1	0.32	0.58
9	-5	-1	-0.03	0.34
-9	5	1	-0.88	0.55
-9	5	1	-1.03	0.52
9	-5	-2	9.65	1.05
-9	5	2	9.13	1.40
-9	5	2	13.65	1.44
9	-5	-3	0.36	0.80
9	-5	-3	-0.01	0.38
-9	5	3	0.06	0.76
-9	5	3	-0.35	0.52
9	-5	-4	31.19	2.76
-9	5	4	23.09	2.34
9	-5	-5	0.28	0.66
9	-5	-5	0.03	0.41
-9	5	5	-0.62	0.72
-9	5	6	6.63	1.05
10	-6	5	4.24	1.50
10	-6	4	1.29	0.63
10	-6	4	1.10	0.50
10	-6	4	5.50	1.46
10	-6	3	3.94	0.85
10	-6	3	7.27	1.45
10	-6	2	15.12	1.48
10	-6	2	18.04	1.70
10	-6	1	4.45	0.86
10	-6	1	3.92	0.62
-10	6	-1	4.59	0.77
10	-6	0	2.08	0.49
10	-6	0	2.14	0.73
-10	6	0	0.87	0.68
10	-6	-1	3.65	0.59
-10	6	1	4.13	0.95
10	-6	-2	14.49	1.43
-10	6	2	20.84	2.05
10	-6	-3	2.15	0.52
-10	6	3	4.07	0.96
10	-6	-4	5.44	1.24
-10	6	4	3.41	0.85
10	-6	-5	0.71	0.56
-10	6	5	0.52	0.95
11	-7	3	-0.48	1.28
11	-7	3	-0.06	0.41
11	-7	2	6.29	1.32
11	-7	1	0.56	0.45
11	-7	1	-0.70	0.62
11	-7	0	8.48	1.32
11	-7	0	4.40	0.71
-11	7	0	5.44	1.05
11	-7	-1	0.37	0.75
11	-7	-1	-0.27	0.39
-11	7	1	-1.29	0.85
11	-7	-2	3.03	0.60
-11	7	2	1.67	1.04
11	-7	-3	-0.38	0.41
11	-7	-3	1.56	0.99
-11	7	3	-1.18	0.99
-6	11	3	0.28	0.60
6	-11	-3	-0.15	0.33
-6	11	2	4.49	0.77
6	-11	-2	4.11	0.57
-6	11	1	0.16	0.56
6	-11	-1	-0.05	0.35
6	-11	0	4.60	0.69
6	-11	1	0.36	0.74
6	-11	1	-0.13	0.35
6	-11	2	4.79	0.71
6	-11	2	6.28	1.03
6	-11	3	0.06	0.34
6	-11	3	0.45	0.83
-5	10	5	-0.11	0.39
5	-10	-5	-0.38	0.30
-5	10	4	0.97	0.47
5	-10	-4	0.56	0.32
-5	10	3	0.13	0.47
5	-10	-3	0.00	0.30
-5	10	2	26.55	2.08
5	-10	-2	25.98	1.94
-5	10	1	0.23	0.41
5	-10	-1	0.01	0.28
-5	10	0	0.93	0.41
5	-10	0	0.16	0.54
5	-10	0	0.63	0.31
5	-10	1	-0.08	0.28
5	-10	1	0.32	0.53
5	-10	2	28.70	2.39
5	-10	2	26.93	2.01
5	-10	3	0.28	0.69
5	-10	3	-0.09	0.29
5	-10	4	1.10	0.75
5	-10	4	1.41	0.39
5	-10	5	0.77	0.57
5	-10	5	-0.17	0.32
-4	9	6	4.12	0.65
-4	9	5	-0.03	0.43
4	-9	-5	-0.06	0.25
-4	9	4	14.36	1.16
4	-9	-4	12.71	1.07
-4	9	3	0.18	0.37
4	-9	-3	-0.35	0.30
4	-9	-3	0.09	0.23
-4	9	2	11.08	1.10
4	-9	-2	12.43	1.11
4	-9	-2	13.82	1.01
-4	9	1	0.02	0.29
4	-9	-1	-0.13	0.26
4	-9	-1	-0.17	0.21
-4	9	0	23.45	1.76
4	-9	0	22.13	1.73
4	-9	0	24.14	1.94
4	-9	0	22.31	1.66
4	-9	1	0.29	0.24
4	-9	1	-0.28	0.44
4	-9	1	0.12	0.27
4	-9	2	11.77	1.00

4	-9	2	12.12	1.28
4	-9	2	12.67	1.15
4	-9	3	0.16	0.54
4	-9	3	0.03	0.25
4	-9	4	12.76	1.10
4	-9	4	15.32	1.35
4	-9	5	0.35	0.63
4	-9	5	-0.25	0.27
4	-9	6	3.33	0.83
-3	8	7	0.93	0.43
3	-8	-7	0.31	1.00
-3	8	6	5.63	0.82
3	-8	-6	4.82	0.66
-3	8	5	2.46	0.46
3	-8	-5	2.41	0.34
-3	8	4	29.29	2.11
3	-8	-4	28.68	2.06
-3	8	3	6.86	0.70
-3	8	3	7.44	0.97
3	-8	-3	6.61	0.79
3	-8	-3	6.98	0.66
3	-8	-3	7.39	0.83
-3	8	2	10.48	1.02
-3	8	2	9.09	0.83
3	-8	-2	8.90	0.78
3	-8	-2	8.11	0.85
-3	8	1	12.03	0.99
-3	8	1	11.81	1.14
3	-8	-1	11.76	1.01
3	-8	-1	10.44	1.37
-3	8	0	61.01	4.09
3	-8	0	59.75	4.08
3	-8	0	57.67	4.21
3	-8	0	57.98	4.03
-3	8	-1	12.00	1.11
3	-8	1	11.29	0.99
3	-8	1	13.71	1.19
3	-8	1	11.52	0.94
3	-8	2	9.09	0.84
3	-8	2	9.05	0.78
3	-8	2	9.72	1.01
3	-8	3	6.98	0.64
3	-8	3	7.17	0.73
3	-8	3	7.37	0.89
3	-8	4	33.00	2.18
3	-8	4	27.91	2.12
3	-8	4	29.57	2.30
3	-8	5	2.02	0.38
3	-8	5	2.86	0.37
3	-8	5	2.84	0.63
3	-8	6	5.20	0.77
3	-8	7	0.45	0.62
-2	7	8	5.44	0.77
2	-7	-8	5.02	1.36
-2	7	7	0.55	0.34
2	-7	-7	0.72	1.00
-2	7	6	13.94	1.10
2	-7	-6	12.34	1.01
-2	7	5	0.27	0.30
2	-7	-5	0.28	0.19
-2	7	4	24.47	1.96
-2	7	4	22.74	1.72
2	-7	-4	24.02	1.84
2	-7	-4	23.84	1.69
-2	7	3	0.55	0.39
-2	7	3	1.27	0.27
2	-7	-3	0.53	0.29
2	-7	-3	0.76	0.20
2	-7	-3	0.64	0.29
-2	7	2	39.79	3.12
-2	7	2	42.87	2.96
2	-7	-2	42.55	2.94
2	-7	-2	42.70	3.03
2	-7	-2	46.80	3.01
-2	7	1	0.57	0.37
-2	7	1	1.05	0.31
2	-7	-1	0.76	0.56
2	-7	-1	0.60	0.20
2	-7	-1	0.50	0.26
-2	7	0	40.39	3.02
-2	7	0	41.97	2.89
2	-7	0	39.62	2.99
2	-7	0	42.74	2.87
2	-7	0	41.04	2.83
-2	7	-1	0.75	0.36
-2	7	-1	0.36	0.37
2	-7	1	0.57	0.25
2	-7	1	0.48	0.19
2	-7	1	0.68	0.41
2	-7	2	42.38	2.83
2	-7	2	43.06	2.87
2	-7	2	37.86	2.90
2	-7	3	0.55	0.19
2	-7	3	0.74	0.36
2	-7	3	0.54	0.25
2	-7	4	22.24	1.67
2	-7	4	24.16	1.71
2	-7	4	24.11	1.81
2	-7	5	0.22	0.24
2	-7	5	0.47	0.41
2	-7	5	0.32	0.21
-2	7	-6	12.28	1.08
2	-7	6	13.78	1.10
2	-7	6	14.80	1.29
-2	7	-7	0.20	0.24
2	-7	7	0.81	0.50
-2	7	-8	4.84	0.61
1	-6	-8	9.77	1.48
-1	6	7	1.53	0.31
-1	6	6	13.38	1.04
1	-6	-6	12.86	0.99
-1	6	5	6.12	1.09
-1	6	5	5.80	0.61
1	-6	-5	5.96	0.54
-1	6	4	49.49	3.48
-1	6	4	51.59	3.78
1	-6	-4	52.42	3.59

1	-6	-4	51.37	3.48
-1	6	3	17.03	1.22
-1	6	3	17.09	1.56
1	-6	-3	14.65	1.26
1	-6	-3	17.67	1.29
1	-6	-3	16.08	1.19
-1	6	2	29.97	2.37
1	-6	-2	30.82	2.17
1	-6	-2	29.93	2.10
1	-6	-2	30.84	2.16
-1	6	1	27.51	1.94
-1	6	1	27.94	2.15
1	-6	-1	26.86	1.91
1	-6	-1	29.17	1.97
1	-6	-1	25.91	2.10
1	-6	-1	27.02	1.95
-1	6	0	103.44	6.98
-1	6	0	102.62	7.13
1	-6	0	105.31	6.95
1	-6	0	101.02	6.97
1	-6	0	106.93	7.04
1	-6	0	102.22	6.99
-1	6	-1	26.18	1.94
-1	6	-1	28.42	2.13
-1	6	-1	26.09	1.86
1	-6	1	26.81	1.98
1	-6	1	27.90	1.86
1	-6	1	25.33	1.89
-1	6	-2	30.66	2.14
-1	6	-2	32.51	2.40
1	-6	2	30.93	2.17
1	-6	2	29.91	2.13
-1	6	-3	15.59	1.11
1	-6	3	15.37	1.16
1	-6	3	14.60	1.24
1	-6	3	15.30	1.11
-1	6	-4	53.94	3.54
1	-6	4	49.15	3.57
1	-6	4	52.92	3.54
1	-6	4	52.42	3.60
-1	6	-5	5.91	0.55
1	-6	5	6.27	0.56
1	-6	5	6.99	0.76
1	-6	5	5.39	0.62
-1	6	-6	11.71	0.96
1	-6	6	13.93	1.12
1	-6	6	12.78	1.03
-1	6	-7	1.04	0.25
1	-6	7	1.38	0.31
1	-6	7	2.63	0.49
-1	6	-8	7.52	0.68
-1	6	-9	0.05	0.25
0	-5	-9	-0.13	0.78
0	-5	-8	6.14	1.02
0	5	7	0.26	0.21
0	5	7	-0.10	0.69
0	5	6	26.43	1.73
0	5	6	19.75	2.34
0	-5	-6	26.01	1.73
0	5	5	0.81	0.66
0	-5	-5	0.76	0.20
0	5	4	38.96	2.55
0	5	4	33.77	2.92
0	-5	-4	38.30	2.64
0	5	3	1.67	0.59
0	5	3	2.29	0.28
0	-5	-3	1.78	0.30
0	-5	-3	1.68	0.25
0	-5	-3	2.09	0.31
0	5	2	74.51	5.34
0	-5	-2	75.11	5.07
0	-5	-2	75.91	5.11
0	-5	-2	75.38	5.09
0	5	1	2.59	0.34
0	5	1	2.05	0.56
0	5	1	2.59	0.33
0	-5	-1	2.73	0.34
0	-5	-1	2.53	0.39
0	-5	-1	2.75	0.55
0	-5	-1	2.59	0.42
0	5	0	78.28	5.25
0	5	0	77.65	5.50
0	5	0	77.61	5.28
0	-5	0	74.69	5.27
0	-5	0	77.64	5.26
0	-5	0	86.36	5.31
0	-5	0	74.93	5.33
0	5	-1	3.10	0.42
0	5	-1	2.67	0.41
0	5	-1	2.15	0.32
0	5	-1	2.66	0.55
0	-5	1	2.16	0.30
0	-5	1	2.77	0.44
0	-5	1	2.96	0.41
0	5	-2	73.99	5.25
0	5	-2	80.06	5.51
0	-5	2	80.56	5.31
0	-5	2	77.16	5.28
0	-5	2	79.70	5.30
0	5	-3	2.71	0.32
0	5	-3	2.06	0.54
0	-5	3	2.56	0.33
0	-5	3	2.09	0.35
0	5	-4	40.53	2.76
0	-5	4	42.46	2.77
0	-5	4	40.90	2.80
0	-5	4	38.34	2.81
0	5	-5	0.94	0.19
0	-5	5	0.87	0.19
0	-5	5	1.08	0.29
0	-5	5	0.96	0.26
0	5	-6	26.23	1.89
0	-5	6	29.42	1.95
0	-5	6	26.12	1.95
0	5	-7	0.24	0.18
0	-5	7	0.19	0.25
0	5	-8	6.52	0.63

0	-5	8	7.61	0.82
0	5	-9	0.05	0.23
-1	-4	-9	-0.66	1.25
1	4	8	9.58	1.85
1	4	7	0.50	0.19
1	4	7	0.80	0.78
1	4	6	44.19	3.15
-1	-4	-6	49.30	3.26
1	4	5	0.43	0.68
1	4	5	0.51	0.18
-1	-4	-5	0.27	0.25
1	4	4	17.29	1.18
1	4	4	16.63	1.20
-1	-4	-4	16.52	1.26
1	4	3	0.47	0.61
1	4	3	0.64	0.18
1	4	3	0.85	0.14
-1	-4	-3	0.73	0.21
-1	-4	-3	0.55	0.19
-1	-4	-3	0.64	0.21
1	4	2	193.41	13.07
1	4	2	176.43	12.75
1	4	2	194.22	12.74
-1	-4	-2	189.46	12.76
-1	-4	-2	190.52	12.79
-1	-4	-2	203.66	12.77
1	4	1	1.30	0.20
1	4	1	1.67	0.25
1	4	1	0.76	0.51
1	4	1	1.18	0.23
-1	-4	-1	1.32	0.22
-1	-4	-1	1.48	0.26
-1	-4	-1	1.29	0.43
-1	-4	-1	1.43	0.23
1	4	0	26.35	1.71
1	4	0	22.25	1.67
1	4	0	25.26	2.05
-1	-4	0	24.70	1.68
-1	-4	0	20.08	1.71
-1	-4	0	24.86	1.67
-1	-4	0	24.10	1.76
1	4	-1	1.80	0.53
1	4	-1	1.42	0.22
1	4	-1	1.04	0.30
-1	-4	1	1.16	0.23
1	4	-2	208.24	13.29
1	4	-2	192.53	13.01
-1	-4	2	187.31	13.05
-1	-4	2	193.39	13.03
1	4	-3	0.71	0.47
1	4	-3	1.16	0.19
-1	-4	3	0.67	0.24
-1	-4	3	0.68	0.22
1	4	-4	16.75	1.18
-1	-4	4	16.62	1.21
-1	-4	4	16.46	1.27
1	4	-5	0.36	0.16
-1	-4	5	0.59	0.22
-1	-4	5	0.43	0.25
1	4	-6	41.83	2.86
-1	-4	6	42.13	2.94
1	4	-7	0.51	0.19
-1	-4	7	0.17	0.27
1	4	-8	6.33	0.61
-1	-4	8	7.18	0.73
1	4	-9	0.03	0.23
-2	-3	-9	-0.38	1.18
-2	-3	-8	11.50	1.52
2	3	7	0.17	0.17
-2	-3	-7	-0.97	0.80
2	3	6	17.73	1.26
-2	-3	-6	18.22	1.46
2	3	5	0.93	0.70
2	3	5	0.78	0.18
-2	-3	-5	0.89	0.35
2	3	4	92.46	6.52
2	3	4	100.07	6.53
2	3	4	111.51	6.60
-2	-3	-4	86.29	6.54
2	3	3	2.97	0.53
2	3	3	3.91	0.40
2	3	3	5.03	0.90
2	3	3	3.68	0.34
-2	-3	-3	3.07	0.39
-2	-3	-3	3.14	0.40
2	3	2	50.35	3.95
2	3	2	51.87	3.49
2	3	2	54.20	3.56
2	3	2	46.45	3.65
2	3	2	50.96	3.51
-2	-3	-2	53.44	3.56
-2	-3	-2	53.15	3.51
-2	-3	-2	53.26	3.55
2	3	1	5.03	0.53
2	3	1	4.33	0.52
2	3	1	5.45	0.53
2	3	1	5.17	0.48
-2	-3	-1	5.20	0.73
-2	-3	-1	5.18	0.57
-2	-3	-1	5.82	0.53
-2	-3	-1	6.16	0.49
2	3	0	172.93	11.62
2	3	0	177.67	11.60
2	3	0	168.73	11.61
2	3	0	175.04	11.91
-2	-3	0	158.41	11.60
-2	-3	0	185.73	11.64
-2	-3	0	177.41	11.60
2	3	-1	5.61	0.50
2	3	-1	4.44	0.96
2	3	-1	5.15	0.54
-2	-3	1	5.34	0.54
2	3	-2	51.95	3.62
2	3	-2	53.90	3.96
2	3	-2	56.11	3.68
-2	-3	2	54.12	3.67
-2	-3	2	51.31	3.62

2	3	-3	3.10	0.65
2	3	-3	3.20	0.48
-2	-3	3	3.49	0.50
-2	-3	3	3.90	0.42
2	3	-4	91.96	5.97
2	3	-4	88.56	6.31
-2	-3	4	88.28	5.96
-2	-3	4	87.03	6.04
2	3	-5	0.72	0.20
-2	-3	5	0.54	0.29
-2	-3	5	0.99	0.19
2	3	-6	17.14	1.29
-2	-3	6	19.16	1.42
2	3	-7	0.32	0.21
-2	-3	7	0.23	0.31
2	3	-8	13.90	1.11
-2	-3	8	15.53	1.24
2	3	-9	0.05	0.28
-3	-2	-9	0.22	1.20
-3	-2	-8	10.91	1.45
3	2	7	0.09	0.19
-3	-2	-7	-0.67	0.78
3	2	6	17.84	1.35
3	2	6	19.25	1.39
-3	-2	-6	21.16	1.66
3	2	5	0.82	0.37
3	2	5	0.75	0.21
3	2	5	1.09	0.26
-3	-2	-5	1.42	0.49
3	2	4	87.77	5.39
3	2	4	74.91	5.47
-3	-2	-4	80.70	5.38
-3	-2	-4	77.07	5.52
3	2	3	3.16	0.40
3	2	3	3.57	0.43
3	2	3	5.39	1.07
3	2	3	3.25	0.46
3	2	3	4.48	0.64
-3	-2	-3	3.06	0.49
-3	-2	-3	3.52	0.54
-3	-2	-3	3.13	0.41
-3	-2	-3	3.14	0.46
-3	-2	-3	3.31	0.35
3	2	2	52.05	3.49
3	2	2	50.72	3.58
3	2	2	53.28	3.53
-3	-2	-2	52.09	3.49
-3	-2	-2	51.77	3.54
-3	-2	-2	48.26	3.57
3	2	1	7.35	1.19
3	2	1	4.00	0.67
3	2	1	6.11	0.55
3	2	1	4.38	0.64
3	2	1	5.43	0.58
-3	-2	-1	4.51	0.60
-3	-2	-1	5.02	0.50
3	2	0	171.60	11.62
3	2	0	166.20	11.92
3	2	0	184.09	11.64
3	2	0	170.43	11.62
3	2	0	163.56	11.68
-3	-2	0	192.58	11.64
-3	-2	0	165.61	11.64
-3	-2	0	175.02	11.62
3	2	-1	4.52	0.51
3	2	-1	4.86	1.03
3	2	-1	4.68	0.50
3	2	-1	5.56	0.64
-3	-2	1	4.18	0.51
-3	-2	1	5.44	0.58
3	2	-2	54.26	3.55
3	2	-2	52.19	3.90
3	2	-2	53.51	3.63
3	2	-2	49.57	3.55
-3	-2	2	52.44	3.54
-3	-2	2	50.11	3.62
3	2	-3	3.70	0.72
3	2	-3	3.79	0.41
3	2	-3	3.03	0.54
3	2	-3	3.64	0.47
-3	-2	3	3.15	0.47
-3	-2	3	3.30	0.40
3	2	-4	91.05	6.03
3	2	-4	87.79	6.11
3	2	-4	89.70	6.36
-3	-2	4	91.12	6.14
-3	-2	4	88.24	5.99
3	2	-5	0.81	0.25
-3	-2	5	0.86	0.17
-3	-2	5	0.52	0.35
3	2	-6	17.31	1.35
-3	-2	6	20.11	1.52
3	2	-7	0.09	0.26
-3	-2	7	0.23	0.37
3	2	-8	13.25	1.16
-3	-2	8	16.11	1.30
3	2	-9	-0.22	0.37
-4	-1	-9	0.28	1.04
-4	-1	-8	5.38	1.06
4	1	7	0.23	0.24
-4	-1	-7	0.28	0.76
4	1	6	45.18	3.08
4	1	6	40.86	3.19
4	1	6	49.31	3.15
4	1	5	0.16	0.30
4	1	5	0.32	0.40
4	1	5	0.69	0.24
-4	-1	-5	1.46	0.62
4	1	4	16.58	1.40
4	1	4	16.94	1.36
4	1	4	17.20	1.26
4	1	3	0.97	0.28
4	1	3	0.68	0.36
4	1	3	1.01	0.40
4	1	3	0.97	0.29
-4	-1	-3	0.68	0.36
-4	-1	-3	0.67	0.18

-4	-1	-3	1.44	0.25
-4	-1	-3	0.41	0.36
-4	-1	-3	1.62	0.54
4	1	2	254.12	15.28
4	1	2	251.41	15.45
4	1	2	210.25	15.24
-4	-1	-2	194.61	15.17
4	1	1	1.99	0.66
4	1	1	1.70	0.38
4	1	1	1.36	0.39
4	1	1	2.49	0.51
4	1	1	1.23	0.35
-4	-1	-1	2.52	0.43
-4	-1	-1	2.84	0.46
-4	-1	-1	0.86	0.30
-4	-1	-1	2.02	0.45
4	1	0	25.41	1.96
4	1	0	28.97	2.10
-4	-1	0	24.44	1.95
-4	-1	0	25.09	1.97
-4	-1	0	26.84	1.97
-4	-1	0	28.50	1.91
4	1	-1	1.11	0.48
4	1	-1	1.40	0.38
4	1	-1	1.35	0.35
4	1	-1	1.19	0.62
-4	-1	1	1.38	0.32
-4	-1	1	2.48	0.46
-4	-1	1	1.30	0.38
4	1	-2	208.78	12.98
4	1	-2	191.83	12.87
4	1	-2	182.35	12.88
4	1	-2	184.03	13.17
-4	-1	2	193.68	12.83
4	1	-3	0.56	0.55
4	1	-3	0.65	0.29
4	1	-3	0.98	0.40
4	1	-3	0.48	0.33
-4	-1	3	0.87	0.25
-4	-1	3	1.15	0.42
4	1	-4	16.48	1.26
4	1	-4	15.05	1.31
4	1	-4	16.84	1.42
4	1	-4	15.42	1.67
-4	-1	4	16.69	1.19
-4	-1	4	20.46	1.49
4	1	-5	0.67	0.21
4	1	-5	0.73	0.39
4	1	-5	0.47	0.32
-4	-1	5	0.57	0.17
-4	-1	5	0.16	0.40
4	1	-6	43.48	3.13
4	1	-6	41.84	3.02
-4	-1	6	43.94	3.14
4	1	-7	-0.07	0.37
-4	-1	7	0.04	0.38
4	1	-8	6.06	1.31
4	1	-8	6.38	0.76
4	1	-9	-1.28	1.38
4	1	-9	0.86	0.57
-5	0	-9	0.91	0.79
-5	0	-9	0.05	1.23
5	0	8	6.75	0.80
5	0	8	8.21	0.82
-5	0	-8	7.11	1.12
5	0	7	0.40	0.33
5	0	7	0.19	0.55
5	0	7	0.66	0.35
-5	0	-7	0.29	0.66
5	0	6	23.91	1.93
5	0	6	25.06	1.91
5	0	6	25.98	1.84
5	0	6	25.35	1.83
-5	0	-6	25.44	2.09
5	0	5	1.21	0.40
5	0	5	0.57	0.42
5	0	5	0.82	0.32
5	0	4	39.15	2.80
5	0	4	37.35	2.68
5	0	3	2.30	0.51
5	0	3	2.13	0.40
-5	0	-3	2.03	0.46
-5	0	-3	1.49	0.51
-5	0	-3	2.26	0.32
5	0	2	65.64	4.62
-5	0	-2	70.21	4.68
-5	0	-2	63.57	4.65
-5	0	-2	65.51	4.57
5	0	1	4.98	0.69
5	0	1	5.11	0.67
5	0	1	3.82	0.76
-5	0	-1	4.43	0.81
-5	0	-1	6.52	0.72
-5	0	-1	5.40	0.73
-5	0	-1	5.65	0.67
-5	0	-1	3.87	0.63
5	0	0	161.12	11.39
5	0	0	169.45	11.33
5	0	0	187.76	11.32
5	0	0	168.61	11.55
5	0	0	150.42	11.28
-5	0	0	177.57	11.38
-5	0	0	173.86	11.33
-5	0	0	154.86	11.26
-5	0	0	166.90	11.32
5	0	-1	3.63	0.70
5	0	-1	3.88	0.54
5	0	-1	3.70	0.84
5	0	-1	5.68	0.76
-5	0	1	3.72	0.63
-5	0	1	3.83	0.64
-5	0	1	5.28	0.64
5	0	-2	66.74	4.71
5	0	-2	62.98	4.86
-5	0	2	67.24	4.49
5	0	-3	2.24	0.62
5	0	-3	2.90	0.47

5	0	-3	1.64	0.66
5	0	-3	3.22	0.47
-5	0	3	2.14	0.38
5	0	-4	41.81	3.11
5	0	-4	38.53	2.92
5	0	-4	45.74	2.97
-5	0	4	40.36	2.83
-5	0	4	40.14	3.03
5	0	-5	0.77	0.32
5	0	-5	2.72	0.58
5	0	-5	1.27	0.45
-5	0	5	1.41	0.52
-5	0	5	1.21	0.26
-5	0	5	0.34	0.50
5	0	-6	29.19	2.18
5	0	-6	27.40	2.25
-5	0	6	28.74	2.34
5	0	-7	-0.81	0.78
5	0	-7	0.88	0.50
-5	0	7	0.24	0.58
-5	0	7	0.40	0.46
5	0	-8	12.35	1.37
5	0	-9	-0.54	1.29
6	-1	9	0.12	0.85
6	-1	8	6.47	1.01
6	-1	7	0.90	0.48
6	-1	7	0.94	0.44
6	-1	6	11.54	1.17
6	-1	6	12.36	1.19
6	-1	5	4.11	0.65
6	-1	5	5.68	0.87
-6	1	-4	32.28	2.25
6	-1	3	14.62	1.33
-6	1	-3	13.96	1.33
6	-1	2	27.78	2.22
-6	1	-2	30.16	2.19
-6	1	-2	27.92	2.32
-6	1	-2	28.59	2.27
6	-1	1	31.58	2.42
-6	1	-1	32.56	2.41
-6	1	-1	31.44	2.53
6	-1	0	188.69	13.18
6	-1	0	207.17	13.40
6	-1	0	196.73	13.26
-6	1	0	183.48	13.24
-6	1	0	192.24	13.20
-6	1	0	205.71	13.17
6	-1	-1	49.98	3.95
6	-1	-1	65.13	4.35
6	-1	-1	59.14	4.15
-6	1	1	57.05	4.09
-6	1	1	50.49	4.01
6	-1	-2	47.95	3.73
-6	1	2	45.03	3.60
-6	1	2	55.82	3.65
6	-1	-3	13.61	1.62
-6	1	3	16.62	1.26
6	-1	-4	109.19	7.02
-6	1	4	104.20	7.12
-6	1	4	94.27	7.13
6	-1	-5	9.71	1.02
-6	1	5	10.30	1.43
-6	1	5	11.97	1.25
6	-1	-6	10.43	1.30
6	-1	-6	13.07	1.37
-6	1	6	12.17	1.14
6	-1	-7	0.72	0.82
6	-1	-7	1.09	0.54
-6	1	7	2.13	0.83
6	-1	-8	7.52	1.27
7	-2	8	3.92	0.83
7	-2	8	4.15	0.69
7	-2	7	-0.21	0.60
7	-2	7	0.05	0.44
7	-2	6	11.10	1.14
7	-2	6	12.31	1.38
7	-2	5	1.18	0.58
7	-2	5	0.83	0.45
7	-2	5	0.47	0.45
7	-2	4	18.83	1.53
-7	2	-4	15.88	1.32
7	-2	3	0.82	0.49
7	-2	3	1.10	0.44
7	-2	3	1.54	0.52
-7	2	-3	0.29	0.39
7	-2	2	38.95	2.91
-7	2	-2	41.67	2.97
-7	2	-2	36.21	3.04
-7	2	-2	38.16	2.86
7	-2	1	0.63	0.50
7	-2	1	0.32	0.38
7	-2	1	2.64	0.73
-7	2	-1	0.40	0.50
-7	2	-1	0.26	0.47
-7	2	-1	0.29	0.63
-7	2	-1	0.29	0.43
7	-2	0	65.99	4.76
7	-2	0	66.76	4.67
-7	2	0	69.24	4.61
-7	2	0	58.66	4.71
7	-2	-1	1.81	0.80
7	-2	-1	2.06	0.73
7	-2	-1	1.33	0.53
7	-2	-1	0.95	0.42
-7	2	1	1.89	0.50
-7	2	1	0.35	0.48
-7	2	1	1.69	0.66
7	-2	-2	66.30	5.20
7	-2	-2	74.84	5.53
-7	2	2	82.72	5.37
-7	2	2	77.30	5.17
7	-2	-3	1.21	0.44
7	-2	-3	0.88	0.77
-7	2	3	1.40	0.42
-7	2	3	0.59	0.51
-7	2	3	1.34	0.56
7	-2	-4	42.56	3.20

-7	2	4	41.39	3.33
-7	2	4	48.45	3.41
7	-2	-5	0.44	0.60
7	-2	-5	0.85	0.45
-7	2	5	0.23	0.35
-7	2	5	0.78	0.77
-7	2	5	0.47	0.59
7	-2	-6	11.95	1.46
-7	2	6	10.89	1.08
7	-2	-7	0.44	0.52
7	-2	-7	-0.70	0.76
7	-2	-8	4.92	0.86
7	-2	-8	4.40	1.15
8	-3	7	1.05	0.86
8	-3	7	0.74	0.54
8	-3	6	6.37	0.85
8	-3	6	7.18	1.05
8	-3	5	4.72	0.79
8	-3	5	2.43	0.62
8	-3	5	2.88	0.61
8	-3	4	26.48	1.99
-8	3	-4	21.59	1.79
8	-3	3	6.19	0.86
8	-3	3	5.73	0.83
-8	3	-3	5.51	0.92
8	-3	2	7.21	0.92
8	-3	2	7.33	1.06
-8	3	-2	6.30	0.96
-8	3	-2	7.46	1.09
-8	3	-2	8.43	1.13
8	-3	1	9.64	1.19
8	-3	1	10.66	1.11
-8	3	-1	9.78	1.40
-8	3	-1	12.21	1.33
-8	3	-1	10.66	1.19
8	-3	0	83.31	5.10
-8	3	0	62.92	5.08
-8	3	0	66.24	5.00
8	-3	-1	24.18	2.17
-8	3	1	23.46	1.95
8	-3	-2	22.60	2.05
-8	3	2	16.01	1.74
-8	3	2	17.54	1.60
-8	3	3	13.97	1.60
-8	3	3	14.08	1.43
8	-3	-4	50.08	4.10
8	-3	-4	50.86	3.69
-8	3	4	56.32	3.92
-8	3	4	45.84	3.84
-8	3	4	53.06	3.66
8	-3	-5	4.08	0.66
-8	3	5	3.58	1.04
-8	3	5	3.95	0.64
-8	3	6	5.85	0.70
8	-3	-7	1.03	0.62
8	-3	-7	-1.30	0.83
-8	3	7	0.79	0.42
9	-4	6	3.61	0.71
9	-4	6	4.01	0.76
9	-4	5	0.53	0.53
9	-4	5	0.33	0.71
9	-4	5	-0.02	0.47
9	-4	4	12.46	1.24
9	-4	4	11.86	1.37
9	-4	3	0.64	0.88
9	-4	3	-0.35	0.53
9	-4	3	-0.22	0.40
-9	4	-3	-0.11	0.43
9	-4	2	9.38	1.11
9	-4	2	9.43	1.28
9	-4	1	0.32	0.55
9	-4	1	-0.27	0.39
9	-4	1	0.45	0.69
-9	4	-1	-0.46	0.51
9	-4	0	20.23	1.63
-9	4	0	18.14	1.93
9	-4	-1	-0.27	0.37
9	-4	-1	-0.23	0.69
-9	4	1	0.31	0.59
-9	4	1	-0.36	0.52
-9	4	2	21.38	1.85
-9	4	2	17.79	1.97
9	-4	-3	-0.27	0.39
9	-4	-3	1.11	0.93
-9	4	3	-0.06	0.52
-9	4	3	0.78	1.05
9	-4	-4	31.59	2.94
-9	4	4	29.31	2.53
9	-4	-5	-0.46	0.61
9	-4	-5	-0.58	0.46
-9	4	5	-0.52	0.64
-9	4	6	6.88	0.95
10	-5	5	-1.01	0.43
10	-5	5	0.03	1.20
10	-5	4	3.80	1.33
10	-5	4	0.67	0.61
10	-5	4	0.35	0.49
10	-5	3	0.77	1.08
10	-5	3	0.04	0.45
10	-5	3	-0.07	0.57
10	-5	2	23.65	1.98
10	-5	2	24.99	2.16
10	-5	1	0.54	0.76
10	-5	1	-0.09	0.43
10	-5	1	-0.04	0.55
-10	5	-1	-0.36	0.53
10	-5	0	0.98	0.66
10	-5	0	0.18	0.44
-10	5	0	0.74	0.64
10	-5	-1	-0.95	0.36
10	-5	-1	1.80	0.83
-10	5	1	-1.06	0.72
10	-5	-2	67.94	4.69
-10	5	2	51.51	4.39
10	-5	-3	-0.42	0.43
10	-5	-3	-0.55	0.91
-10	5	3	-0.09	0.67

10	-5	-4	3.47	1.17
-10	5	4	2.26	0.79
10	-5	-5	0.55	0.78
10	-5	-5	-1.27	0.68
-10	5	5	0.11	0.87
11	-6	3	1.14	1.28
11	-6	3	0.20	0.48
11	-6	2	3.86	1.18
11	-6	2	3.44	0.67
11	-6	1	-0.07	0.64
11	-6	1	-0.37	0.45
11	-6	0	3.22	0.67
-11	6	0	5.51	1.05
11	-6	-1	1.09	0.82
11	-6	-1	-0.22	0.45
-11	6	1	-1.40	0.88
-11	6	2	6.98	1.24
11	-6	-3	-1.12	0.44
11	-6	-3	1.01	1.06
-11	6	3	-1.41	0.90
-5	11	3	0.25	0.57
5	-11	-3	0.12	0.33
-5	11	2	4.35	0.77
5	-11	-2	5.46	0.73
-5	11	1	-0.13	0.55
5	-11	-1	0.13	0.35
5	-11	0	4.89	0.68
5	-11	1	0.71	0.75
5	-11	1	0.34	0.35
5	-11	2	5.27	0.72
5	-11	2	5.59	1.00
5	-11	3	-0.07	0.77
5	-11	3	0.16	0.36
-4	10	5	0.68	0.42
4	-10	-5	0.99	0.35
-4	10	4	2.19	0.55
4	-10	-4	1.74	0.38
-4	10	3	2.72	0.58
4	-10	-3	2.84	0.43
-4	10	2	18.52	1.57
4	-10	-2	17.32	1.40
-4	10	1	4.50	0.63
4	-10	-1	5.02	0.60
-4	10	0	2.60	0.52
4	-10	0	2.04	0.38
4	-10	0	3.69	0.70
4	-10	1	5.71	0.85
4	-10	1	4.84	0.62
4	-10	2	18.90	1.84
4	-10	2	18.29	1.44
4	-10	3	3.69	0.81
4	-10	3	2.83	0.45
4	-10	4	3.11	0.79
4	-10	4	2.27	0.42
4	-10	5	1.11	0.40
4	-10	5	1.37	0.59
-3	9	6	7.97	1.05
-3	9	5	0.21	0.42
3	-9	-5	-0.04	0.25
-3	9	4	4.14	0.67
3	-9	-4	4.43	0.59
3	-9	-4	6.34	0.82
-3	9	3	0.72	0.40
3	-9	-3	0.24	0.26
3	-9	-3	0.38	0.34
3	-9	-3	0.43	0.33
-3	9	2	26.14	2.08
-3	9	2	26.05	1.90
3	-9	-2	24.83	1.84
3	-9	-2	24.07	1.92
-3	9	1	0.44	0.43
-3	9	1	0.30	0.31
3	-9	-1	0.57	0.32
3	-9	-1	0.16	0.24
-3	9	0	7.24	0.83
3	-9	0	6.79	0.67
3	-9	0	6.85	0.76
3	-9	0	6.32	0.86
3	-9	1	1.14	0.27
3	-9	1	0.35	0.37
3	-9	1	0.31	0.47
3	-9	2	25.08	2.01
3	-9	2	24.36	1.79
3	-9	3	0.11	0.26
3	-9	3	0.71	0.56
3	-9	4	5.00	0.68
3	-9	4	4.98	0.61
3	-9	5	0.07	0.29
3	-9	5	0.30	0.62
3	-9	6	7.03	0.96
-2	8	7	1.66	0.44
-2	8	6	13.07	1.14
2	-8	-6	11.06	0.97
-2	8	5	6.25	0.82
2	-8	-5	6.15	0.62
-2	8	4	6.05	0.71
2	-8	-4	6.79	0.84
2	-8	-4	5.65	0.62
-2	8	3	13.23	1.07
-2	8	3	13.64	1.37
2	-8	-3	13.53	1.04
2	-8	-3	13.53	1.18
2	-8	-3	12.88	1.16
-2	8	2	46.13	3.28
-2	8	2	43.75	3.05
2	-8	-2	41.40	3.10
2	-8	-2	44.37	3.03
-2	8	1	23.44	1.70
-2	8	1	22.63	1.92
2	-8	-1	22.82	1.72
2	-8	-1	23.18	1.65
2	-8	-1	22.27	2.00
-2	8	0	6.65	0.85
-2	8	0	7.32	1.03
2	-8	0	9.13	0.72
2	-8	0	7.01	0.87
2	-8	0	8.18	0.78

-2	8	-1	24.36	1.80
-2	8	-1	21.64	1.95
2	-8	1	22.46	1.71
2	-8	1	23.39	1.66
2	-8	1	23.05	1.83
2	-8	2	41.19	3.12
2	-8	2	46.41	3.08
2	-8	2	43.79	3.02
2	-8	3	14.01	1.08
2	-8	3	13.68	1.14
2	-8	3	14.20	1.24
2	-8	4	5.55	0.72
2	-8	4	6.62	0.67
2	-8	4	5.18	0.60
2	-8	5	5.20	0.58
2	-8	5	4.94	0.71
2	-8	6	13.13	1.38
2	-8	7	1.37	0.62
-1	7	8	6.63	0.82
1	-7	-8	4.73	1.56
-1	7	7	0.55	0.35
-1	7	6	5.72	0.72
1	-7	-6	5.59	0.58
-1	7	5	1.22	0.34
-1	7	5	1.63	0.60
1	-7	-5	0.79	0.24
-1	7	4	38.84	2.73
-1	7	4	41.13	3.09
1	-7	-4	38.32	2.84
1	-7	-4	38.86	2.71
-1	7	3	3.55	0.47
-1	7	3	3.15	0.69
1	-7	-3	2.69	0.44
1	-7	-3	3.43	0.43
1	-7	-3	3.39	0.46
-1	7	2	12.54	1.34
-1	7	2	12.38	0.96
1	-7	-2	11.29	0.93
1	-7	-2	11.20	1.02
1	-7	-2	12.33	1.03
-1	7	1	6.63	0.70
-1	7	1	6.87	1.04
1	-7	-1	6.62	0.85
1	-7	-1	6.12	0.68
1	-7	-1	6.01	0.58
-1	7	0	73.85	4.91
-1	7	0	69.08	5.12
1	-7	0	72.43	4.91
1	-7	0	68.24	4.86
1	-7	0	76.30	5.02
-1	7	-1	5.85	1.01
-1	7	-1	6.39	0.82
1	-7	1	6.47	0.67
1	-7	1	6.96	0.85
1	-7	1	6.24	0.59
-1	7	-2	12.16	1.30
1	-7	2	12.06	0.99
1	-7	2	11.78	1.07
1	-7	2	12.16	0.93
1	-7	3	4.02	0.43
1	-7	3	2.73	0.40
1	-7	3	2.51	0.46
1	-7	4	38.01	2.60
1	-7	4	39.45	2.72
1	-7	4	35.40	2.64
1	-7	5	0.76	0.43
1	-7	5	0.92	0.29
1	-7	5	0.96	0.24
1	-7	6	6.70	0.95
1	-7	7	0.39	0.47
0	6	8	13.70	1.12
0	-6	-8	12.59	1.75
0	6	7	0.12	0.28
0	6	6	0.89	0.30
0	6	6	1.40	0.73
0	-6	-6	0.92	0.23
0	6	5	0.30	0.26
0	6	5	0.60	0.65
0	-6	-5	0.24	0.20
0	6	4	106.85	6.97
0	6	4	104.69	7.35
0	-6	-4	99.59	7.05
0	6	3	0.34	0.56
0	6	3	0.23	0.21
0	-6	-3	0.42	0.26
0	-6	-3	0.93	0.28
0	6	2	1.87	0.31
0	6	2	2.05	0.65
0	-6	-2	2.24	0.35
0	-6	-2	2.54	0.57
0	-6	-2	2.12	0.34
0	6	1	0.43	0.54
0	6	1	0.19	0.28
0	-6	-1	0.77	0.53
0	-6	-1	0.49	0.24
0	-6	-1	0.46	0.30
0	6	0	239.76	15.97
0	6	0	249.73	16.25
0	-6	0	231.99	15.96
0	-6	0	234.89	16.02
0	6	-1	0.28	0.36
0	6	-1	1.72	0.60
0	6	-1	0.19	0.39
0	-6	1	0.34	0.40
0	-6	1	0.43	0.24
0	6	-2	2.06	0.58
0	-6	2	1.94	0.33
0	6	-3	0.26	0.18
0	6	-3	0.33	0.50
0	-6	3	0.22	0.32
0	-6	3	0.31	0.20
0	-6	3	0.40	0.23
0	6	-4	110.90	7.49
0	-6	4	111.93	7.53
0	-6	4	111.56	7.49
0	-6	4	112.36	7.54
0	6	-5	0.25	0.19

0	-6	5	0.06	0.25
0	-6	5	0.14	0.18
0	-6	5	0.61	0.35
0	6	-6	1.03	0.23
0	-6	6	0.85	0.30
0	-6	6	1.21	0.39
0	6	-7	-0.15	0.21
0	-6	7	0.15	0.37
0	6	-8	11.46	0.95
1	5	8	8.04	0.74
-1	-5	-8	4.14	1.52
1	5	7	1.24	0.29
1	5	7	1.51	0.93
1	5	6	10.49	1.84
1	5	6	12.43	0.95
1	5	5	5.02	0.54
1	5	5	4.78	1.06
1	5	4	47.96	3.10
1	5	4	40.15	3.57
-1	-5	-4	46.91	3.17
1	5	3	14.55	1.72
1	5	3	15.54	1.12
-1	-5	-3	15.33	1.17
-1	-5	-3	14.44	1.16
1	5	2	28.51	2.68
-1	-5	-2	29.70	2.24
-1	-5	-2	30.89	2.22
-1	-5	-2	36.85	2.29
1	5	1	24.39	1.81
1	5	1	26.82	2.32
-1	-5	-1	25.73	1.85
-1	-5	-1	23.84	1.80
-1	-5	-1	26.38	2.00
1	5	0	103.12	6.69
1	5	0	95.62	6.68
1	5	0	101.43	7.04
-1	-5	0	99.75	6.72
-1	-5	0	95.13	6.73
-1	-5	0	99.17	6.68
1	5	-1	26.38	1.83
1	5	-1	26.68	2.30
1	5	-1	25.41	1.93
-1	-5	1	25.82	1.87
1	5	-2	28.92	2.42
1	5	-2	30.81	2.03
-1	-5	2	27.52	2.07
1	5	-3	16.07	1.15
-1	-5	3	15.72	1.22
-1	-5	3	15.39	1.22
1	5	-4	49.67	3.43
-1	-5	4	50.55	3.46
-1	-5	4	51.15	3.50
1	5	-5	5.61	0.56
-1	-5	5	5.96	0.65
-1	-5	5	6.14	0.55
-1	-5	5	5.23	0.67
1	5	-6	13.21	1.00
-1	-5	6	13.82	1.06
-1	-5	6	11.99	1.10
1	5	-7	1.24	0.26
-1	-5	7	1.20	0.42
1	5	-8	7.04	0.68
2	4	9	0.42	1.12
-2	-4	-9	-1.58	1.60
2	4	8	5.91	1.30
2	4	8	3.38	0.54
-2	-4	-8	3.09	1.39
2	4	7	4.38	0.54
2	4	7	6.02	1.24
2	4	6	38.37	2.64
2	4	5	16.71	1.23
2	4	4	10.55	1.57
2	4	4	7.88	0.69
-2	-4	-4	6.87	0.70
2	4	3	48.56	3.33
2	4	3	51.27	3.87
2	4	3	48.01	3.32
-2	-4	-3	46.53	3.35
-2	-4	-3	49.80	3.34
2	4	2	178.30	10.55
2	4	2	141.23	10.91
-2	-4	-2	155.50	10.57
-2	-4	-2	154.00	10.54
2	4	1	92.14	5.92
2	4	1	90.02	5.91
2	4	1	85.60	6.32
-2	-4	-1	80.27	5.98
-2	-4	-1	86.94	5.96
-2	-4	-1	89.68	5.91
2	4	0	6.96	0.68
2	4	0	8.09	1.34
2	4	0	7.86	0.82
2	4	0	7.37	0.68
-2	-4	0	8.49	0.70
-2	-4	0	7.57	0.80
-2	-4	0	7.79	0.90
2	4	-1	92.23	6.14
2	4	-1	84.45	6.10
2	4	-1	95.43	6.52
2	4	-1	89.56	6.08
-2	-4	1	90.05	6.11
2	4	-2	154.90	10.82
2	4	-2	172.24	11.19
2	4	-2	161.27	10.77
-2	-4	2	150.46	10.81
-2	-4	2	167.12	10.79
2	4	-3	50.44	3.47
2	4	-3	51.81	3.90
-2	-4	3	52.49	3.54
-2	-4	3	49.26	3.48
2	4	-4	7.95	0.69
2	4	-4	7.59	1.33
-2	-4	4	9.05	0.81
-2	-4	4	7.33	0.72
2	4	-5	17.92	1.36
-2	-4	5	18.58	1.46
-2	-4	5	19.65	1.36

2	4	-6	37.92	2.77
-2	-4	6	41.27	2.88
-2	-4	6	41.63	2.77
2	4	-7	4.53	0.55
-2	-4	7	5.05	0.70
2	4	-8	3.78	0.56
2	4	-9	0.65	0.38
-3	-3	-9	-0.30	1.42
3	3	8	4.36	0.60
-3	-3	-8	4.04	1.40
3	3	7	-0.14	0.25
3	3	6	53.99	3.90
3	3	6	43.54	3.28
3	3	5	0.39	0.70
3	3	5	-0.03	0.24
3	3	4	6.78	0.67
3	3	4	6.66	0.65
3	3	3	0.98	0.39
3	3	3	0.34	0.24
3	3	3	-0.14	0.22
3	3	3	-0.16	0.63
-3	-3	-3	-0.07	0.19
-3	-3	-3	0.26	0.33
-3	-3	-3	-0.08	0.20
3	3	2	183.74	11.81
3	3	2	180.36	11.86
-3	-3	-2	168.88	11.83
-3	-3	-2	172.10	11.78
3	3	1	0.63	0.31
3	3	1	-0.21	0.43
3	3	1	-0.09	0.28
3	3	1	0.10	0.22
3	3	1	-0.26	0.64
-3	-3	-1	0.72	0.24
-3	-3	-1	0.31	0.35
3	3	0	4.60	0.61
3	3	0	5.70	0.60
3	3	0	3.45	0.91
3	3	0	5.11	0.54
-3	-3	0	5.22	0.78
-3	-3	0	5.34	0.58
3	3	-1	0.29	0.40
3	3	-1	-0.24	0.22
3	3	-1	0.22	0.30
3	3	-1	0.16	0.22
3	3	-1	0.21	0.68
-3	-3	1	0.04	0.26
-3	-3	1	0.04	0.18
3	3	-2	175.08	12.27
3	3	-2	178.36	12.20
3	3	-2	193.71	12.64
-3	-3	2	191.29	12.27
-3	-3	2	174.79	12.20
3	3	-3	0.07	0.32
3	3	-3	-0.04	0.60
3	3	-3	-0.17	0.24
-3	-3	3	0.08	0.24
-3	-3	3	0.05	0.30
3	3	-4	6.70	1.32
3	3	-4	6.48	0.63
3	3	-4	5.78	0.74
-3	-3	4	6.43	0.76
-3	-3	4	6.01	0.61
3	3	-5	-0.08	0.25
-3	-3	5	0.43	0.22
-3	-3	5	0.20	0.33
3	3	-6	40.45	2.93
-3	-3	6	44.30	3.05
3	3	-7	-0.03	0.28
-3	-3	7	-0.01	0.35
3	3	-8	3.18	0.45
3	3	-9	0.07	0.40
-4	-2	-9	-0.15	1.28
4	2	8	3.58	0.47
-4	-2	-8	3.67	1.26
4	2	7	4.25	0.60
-4	-2	-7	2.56	1.04
4	2	6	38.53	2.72
4	2	6	39.84	2.76
4	2	5	18.25	1.38
4	2	5	19.65	1.44
4	2	5	17.54	1.51
4	2	4	10.47	1.47
4	2	4	8.34	0.79
4	2	4	7.51	0.84
4	2	3	37.50	2.87
-4	-2	-3	40.91	2.90
-4	-2	-3	46.16	2.85
4	2	2	287.11	21.02
4	2	2	342.49	21.33
-4	-2	-2	347.60	21.01
-4	-2	-2	279.12	20.96
4	2	1	106.88	6.50
4	2	1	94.28	6.37
4	2	1	90.86	6.37
-4	-2	-1	89.18	6.35
-4	-2	-1	88.03	6.38
4	2	0	7.63	0.76
4	2	0	6.19	0.82
4	2	0	8.33	1.48
4	2	0	6.38	0.91
4	2	0	9.52	0.94
-4	-2	0	8.61	0.85
-4	-2	0	6.11	0.79
4	2	-1	83.84	5.78
4	2	-1	77.36	6.19
4	2	-1	92.62	5.89
4	2	-1	85.94	5.79
-4	-2	1	84.39	5.83
4	2	-2	158.55	10.58
4	2	-2	168.02	10.71
4	2	-2	155.38	10.59
4	2	-2	156.13	11.02
-4	-2	2	157.45	10.66
-4	-2	2	150.29	10.56
4	2	-3	46.90	3.93
4	2	-3	53.10	3.45

4	2	-3	47.44	3.44
4	2	-3	50.23	3.58
-4	-2	3	50.86	3.41
-4	-2	3	49.41	3.56
4	2	-4	6.80	0.87
4	2	-4	7.11	0.70
4	2	-4	7.85	0.77
4	2	-4	7.08	1.41
-4	-2	4	7.88	0.68
-4	-2	4	7.17	0.94
4	2	-5	15.09	1.88
4	2	-5	17.95	1.35
4	2	-5	17.72	1.49
-4	-2	5	18.63	1.27
-4	-2	5	15.94	1.48
4	2	-6	39.04	2.75
-4	-2	6	38.53	2.88
4	2	-7	4.30	0.56
-4	-2	7	3.93	0.92
4	2	-8	3.24	0.57
5	1	8	7.16	0.85
-5	-1	-8	6.41	1.26
5	1	7	1.47	0.40
5	1	7	1.34	0.42
-5	-1	-7	0.50	0.87
5	1	6	12.27	1.10
5	1	6	11.69	1.08
5	1	6	12.66	1.10
5	1	5	4.88	0.86
5	1	5	5.74	0.67
5	1	5	5.69	0.74
5	1	5	6.23	0.76
-5	-1	-4	30.35	2.09
-5	-1	-4	29.02	2.04
-5	-1	-3	12.16	1.08
-5	-1	-3	13.30	0.99
-5	-1	-3	12.94	1.31
-5	-1	-3	12.06	1.28
5	1	2	54.76	3.64
5	1	2	45.36	3.58
5	1	2	50.70	3.86
5	1	1	54.47	4.13
5	1	1	54.83	3.85
5	1	1	43.09	3.73
-5	-1	-1	57.35	3.83
-5	-1	-1	56.33	3.75
5	1	0	177.84	11.66
5	1	0	199.40	11.94
5	1	0	157.66	11.54
-5	-1	0	168.57	11.55
-5	-1	0	159.65	11.47
-5	-1	0	165.05	11.56
5	1	-1	29.02	2.12
5	1	-1	26.92	2.29
5	1	-1	27.75	2.67
-5	-1	1	28.85	2.18
5	1	-2	26.26	2.06
5	1	-2	28.36	2.26
5	1	-2	23.63	2.58
5	1	-2	30.71	2.09
-5	-1	2	27.41	2.02
-5	-1	2	28.06	2.14
5	1	-3	15.91	1.30
5	1	-3	14.98	1.45
5	1	-3	13.95	1.25
5	1	-3	13.44	1.86
-5	-1	3	15.52	1.20
5	1	-4	49.98	3.23
5	1	-4	45.76	3.18
5	1	-4	37.57	3.56
5	1	-4	45.40	3.36
-5	-1	4	46.87	3.12
5	1	-5	4.15	0.95
5	1	-5	7.36	0.68
5	1	-5	5.19	0.91
5	1	-5	6.01	0.85
-5	-1	5	5.58	0.62
5	1	-6	11.00	1.22
5	1	-6	10.53	1.18
-5	-1	6	12.28	0.94
5	1	-7	1.86	0.51
-5	-1	7	-0.07	0.88
5	1	-8	9.27	1.22
6	0	8	12.10	1.29
6	0	7	0.06	0.49
6	0	6	0.66	0.48
6	0	6	1.50	0.46
6	0	6	1.08	0.51
6	0	5	0.07	0.45
6	0	5	0.07	0.41
6	0	5	0.79	0.43
6	0	4	103.65	7.29
6	0	4	108.56	7.21
6	0	3	0.08	0.40
6	0	3	0.96	0.47
6	0	3	-0.14	0.35
-6	0	-3	0.18	0.43
-6	0	-3	-0.36	0.51
6	0	2	5.31	0.67
6	0	2	2.82	0.78
6	0	2	3.62	0.62
6	0	2	4.15	0.64
-6	0	-2	2.35	0.58
-6	0	-2	3.43	0.68
-6	0	-2	4.47	0.74
-6	0	-2	4.83	0.66
6	0	1	1.15	0.49
6	0	1	1.05	0.55
6	0	1	0.89	0.55
6	0	1	0.71	0.68
-6	0	-1	1.20	0.39
-6	0	-1	1.66	0.60
-6	0	-1	0.57	0.49
6	0	0	601.47	37.53
6	0	0	543.19	37.28
6	0	0	568.25	37.21
-6	0	0	537.31	37.24

-6	0	0	554.96	37.19
-6	0	0	541.05	37.14
6	0	-1	0.38	0.63
6	0	-1	0.92	0.50
6	0	-1	0.62	0.45
6	0	-1	0.29	0.79
-6	0	1	0.42	0.53
-6	0	1	0.57	0.46
6	0	-2	4.10	0.58
6	0	-2	2.40	0.78
6	0	-2	1.19	0.84
-6	0	2	2.75	0.43
-6	0	2	4.59	0.72
-6	0	2	4.63	0.66
6	0	-3	0.83	0.43
6	0	-3	0.23	0.68
6	0	-3	-0.48	0.56
6	0	-3	0.78	0.41
-6	0	3	0.50	0.48
-6	0	3	0.14	0.44
-6	0	3	0.18	0.38
6	0	-4	162.60	10.49
6	0	-4	160.20	10.50
-6	0	4	142.69	10.55
6	0	-5	-0.01	0.34
6	0	-5	-0.26	0.49
6	0	-5	0.66	0.44
-6	0	5	0.07	0.34
-6	0	5	0.14	0.53
-6	0	5	0.55	0.48
6	0	-6	1.14	0.58
6	0	-6	2.29	0.54
-6	0	6	1.03	0.36
-6	0	6	0.88	0.66
6	0	-7	0.48	0.53
6	0	-7	-0.78	0.47
7	-1	8	5.05	0.87
7	-1	7	0.13	0.50
7	-1	7	-0.08	0.62
7	-1	6	5.08	0.78
7	-1	6	5.54	0.82
7	-1	5	0.91	0.53
7	-1	5	2.28	0.55
7	-1	5	1.02	0.58
7	-1	4	33.02	2.28
-7	1	-4	24.85	2.08
7	-1	3	3.29	0.62
-7	1	-3	1.28	0.72
-7	1	-3	3.04	0.61
7	-1	2	13.97	1.34
-7	1	-2	16.30	1.53
-7	1	-2	14.76	1.61
-7	1	-2	12.85	1.38
7	-1	1	9.04	1.48
7	-1	1	11.24	1.27
7	-1	1	12.46	1.19
-7	1	-1	13.56	1.50
-7	1	-1	13.91	1.19
-7	1	-1	11.93	1.30
7	-1	0	137.49	10.63
7	-1	0	157.35	10.76
7	-1	0	165.68	10.96
-7	1	0	153.64	10.74
-7	1	0	149.09	10.66
-7	1	0	178.79	10.65
7	-1	-1	15.57	1.39
7	-1	-1	15.00	1.68
7	-1	-1	15.92	1.94
-7	1	1	14.98	1.46
-7	1	1	14.21	1.57
-7	1	1	14.78	1.31
7	-1	-2	26.52	2.00
7	-1	-2	27.71	2.32
7	-1	-2	21.00	2.47
-7	1	2	27.29	2.10
-7	1	2	20.43	1.82
-7	1	2	26.46	2.08
7	-1	-3	5.94	1.09
-7	1	3	4.42	0.70
-7	1	3	8.55	1.10
7	-1	-4	86.52	5.69
-7	1	4	78.89	5.76
-7	1	4	79.68	5.80
7	-1	-5	2.21	0.50
7	-1	-5	0.87	0.71
-7	1	5	1.73	0.73
-7	1	5	2.35	0.87
-7	1	5	0.62	0.45
7	-1	-6	4.84	0.98
-7	1	6	5.55	0.67
7	-1	-7	-0.69	0.60
7	-1	-8	5.03	0.95
8	-2	7	0.75	0.57
8	-2	7	1.35	0.80
8	-2	6	14.66	1.63
8	-2	6	10.26	1.30
8	-2	5	10.53	1.22
8	-2	5	9.66	1.26
8	-2	4	5.14	0.88
-8	2	-4	4.35	0.70
8	-2	3	25.41	2.05
8	-2	2	42.96	3.25
-8	2	-2	42.45	3.43
-8	2	-2	43.66	3.92
-8	2	-2	47.00	3.31
8	-2	1	32.25	2.76
8	-2	1	36.48	2.74
8	-2	1	30.25	2.86
-8	2	-1	38.18	2.94
-8	2	-1	42.86	2.81
-8	2	-1	41.48	2.76
8	-2	0	16.86	1.87
8	-2	0	12.40	1.38
8	-2	0	15.22	1.57
-8	2	0	13.52	1.42
-8	2	0	16.73	1.45
-8	2	0	13.54	1.49

8	-2	-1	59.69	4.01
-8	2	1	41.61	3.72
-8	2	1	47.97	3.81
-8	2	1	57.09	3.71
8	-2	-2	88.10	6.24
-8	2	2	96.64	6.19
-8	2	2	83.28	6.31
-8	2	2	91.52	6.35
8	-2	-3	28.55	2.29
8	-2	-3	31.82	2.76
-8	2	3	26.65	2.23
-8	2	3	32.10	2.45
-8	2	3	28.60	2.42
8	-2	-4	8.88	1.34
-8	2	4	7.93	1.06
-8	2	5	6.04	0.84
8	-2	-6	7.14	1.26
-8	2	6	11.32	1.01
8	-2	-7	0.58	0.66
-8	2	7	1.10	0.48
9	-3	6	12.98	1.53
9	-3	6	16.48	1.72
9	-3	5	0.27	0.73
9	-3	5	-0.04	0.50
9	-3	5	-0.50	0.52
9	-3	4	4.52	0.80
9	-3	4	8.04	1.08
9	-3	3	-0.50	0.50
9	-3	3	-0.02	0.47
9	-3	3	1.61	0.84
-9	3	-3	-0.13	0.45
9	-3	2	22.09	1.90
9	-3	1	0.92	0.52
9	-3	1	0.80	0.56
9	-3	1	0.89	0.68
9	-3	1	-0.52	0.56
-9	3	-1	0.18	0.54
-9	3	-1	0.10	0.64
-9	3	-1	-0.73	0.42
9	-3	0	11.96	1.68
9	-3	0	12.07	1.27
9	-3	0	14.67	1.57
-9	3	0	10.02	1.35
-9	3	0	11.15	1.48
-9	3	0	14.64	1.41
9	-3	-1	0.83	0.75
9	-3	-1	0.02	0.45
-9	3	1	0.04	0.55
-9	3	1	0.99	0.57
-9	3	1	0.97	0.62
9	-3	-2	40.02	3.21
-9	3	2	52.46	3.50
-9	3	2	39.26	3.30
9	-3	-3	0.53	0.51
9	-3	-3	1.38	0.96
-9	3	3	0.83	0.55
9	-3	-4	9.63	1.19
9	-3	-4	10.56	1.57
-9	3	4	9.95	1.41
9	-3	-5	-0.61	0.76
9	-3	-5	0.13	0.52
-9	3	5	0.20	0.59
-9	3	6	12.05	1.46
10	-4	5	3.55	1.20
10	-4	5	1.22	0.64
10	-4	5	0.30	0.92
10	-4	4	1.23	0.65
10	-4	4	5.56	1.26
10	-4	4	2.37	0.67
10	-4	3	2.33	0.69
10	-4	3	2.69	0.66
10	-4	2	17.28	1.76
10	-4	2	20.33	1.68
10	-4	1	4.50	0.76
10	-4	1	5.91	0.91
-10	4	-1	4.01	0.81
-10	4	-1	7.63	1.15
10	-4	0	3.73	0.86
10	-4	0	1.57	0.59
-10	4	0	4.86	0.88
-10	4	0	2.31	0.74
10	-4	-1	13.52	1.79
-10	4	1	10.45	1.31
10	-4	-2	50.86	3.87
-10	4	2	42.51	3.59
10	-4	-3	10.10	1.61
-10	4	3	8.69	1.40
10	-4	-4	5.19	1.28
10	-4	-4	2.48	0.69
-10	4	4	4.36	0.84
10	-4	-5	2.69	1.11
-10	4	5	2.60	0.90
11	-5	3	0.85	1.15
11	-5	3	-0.24	0.52
11	-5	2	3.80	0.73
11	-5	2	4.90	1.26
11	-5	1	-0.68	0.50
11	-5	1	0.76	0.66
11	-5	0	8.38	1.40
-11	5	0	9.01	1.28
11	-5	-1	0.48	0.85
11	-5	-1	-0.40	0.52
-11	5	1	-0.08	0.88
-11	5	2	8.93	1.30
11	-5	-3	1.36	1.08
11	-5	-3	-0.91	0.52
-11	5	3	0.21	0.91
-4	11	3	-0.11	0.56
4	-11	-3	0.14	0.32
-4	11	2	2.74	0.67
4	-11	-2	2.10	0.44
-4	11	1	0.34	0.59
4	-11	-1	-0.07	0.32
4	-11	0	5.79	0.73
4	-11	1	-0.04	0.32
4	-11	1	0.26	0.77
4	-11	2	2.48	0.46

4	-11	2	3.44	0.89
4	-11	3	-0.01	0.35
4	-11	3	0.66	0.77
-3	10	5	0.94	0.39
3	-10	-5	0.79	0.34
-3	10	4	8.33	1.07
3	-10	-4	7.45	0.81
-3	10	3	2.59	0.59
3	-10	-3	1.85	0.40
-3	10	2	3.79	0.65
3	-10	-2	3.50	0.49
-3	10	1	3.42	0.72
-3	10	1	3.95	0.62
3	-10	-1	3.98	0.57
3	-10	0	13.65	1.14
3	-10	0	13.45	1.48
3	-10	1	3.76	0.76
3	-10	1	3.80	0.50
3	-10	2	4.55	0.84
3	-10	2	3.14	0.47
3	-10	3	1.74	0.40
3	-10	3	2.84	0.76
3	-10	4	6.56	0.79
3	-10	4	9.65	1.40
3	-10	5	1.68	0.58
3	-10	5	0.62	0.37
-2	9	6	5.89	0.95
2	-9	-6	6.59	0.78
-2	9	5	0.06	0.31
2	-9	-5	0.13	0.28
-2	9	4	4.96	0.76
2	-9	-4	4.10	0.67
2	-9	-4	4.68	0.60
-2	9	3	0.30	0.51
-2	9	3	0.43	0.41
2	-9	-3	0.25	0.32
2	-9	-3	-0.13	0.24
2	-9	-3	0.46	0.33
-2	9	2	16.76	1.67
-2	9	2	18.46	1.44
2	-9	-2	17.11	1.42
2	-9	-2	16.53	1.31
-2	9	1	0.10	0.51
-2	9	1	0.40	0.34
2	-9	-1	0.44	0.38
2	-9	-1	0.32	0.25
-2	9	0	9.26	0.92
-2	9	0	8.38	1.14
2	-9	0	6.93	1.12
2	-9	0	7.63	0.74
-2	9	-1	0.36	0.57
2	-9	1	0.16	0.46
2	-9	1	-0.03	0.24
2	-9	2	17.30	1.36
2	-9	2	18.35	1.66
2	-9	3	0.25	0.26
2	-9	3	0.18	0.55
2	-9	4	5.40	0.70
2	-9	4	4.73	0.63
2	-9	5	0.90	0.64
2	-9	6	5.80	0.89
-1	8	7	0.01	0.42
-1	8	6	12.34	1.15
1	-8	-6	12.47	1.03
-1	8	5	0.49	0.37
1	-8	-5	0.32	0.24
-1	8	4	3.01	0.73
-1	8	4	2.18	0.37
1	-8	-4	1.95	0.34
1	-8	-4	2.10	0.50
-1	8	3	0.33	0.31
-1	8	3	0.68	0.57
1	-8	-3	0.12	0.31
1	-8	-3	0.14	0.24
1	-8	-3	0.23	0.30
-1	8	2	49.69	3.73
-1	8	2	49.87	3.38
1	-8	-2	48.81	3.43
1	-8	-2	46.21	3.35
-1	8	1	0.51	0.31
-1	8	1	0.25	0.54
1	-8	-1	0.27	0.29
1	-8	-1	-0.05	0.60
1	-8	-1	-0.04	0.21
-1	8	0	1.68	0.41
-1	8	0	1.16	0.57
1	-8	0	1.40	0.36
-1	8	-1	0.56	0.52
1	-8	1	0.33	0.24
1	-8	1	0.21	0.30
1	-8	1	0.24	0.43
-1	8	-2	47.09	3.73
1	-8	2	47.96	3.33
1	-8	2	47.65	3.38
1	-8	2	50.59	3.44
1	-8	3	0.52	0.39
1	-8	3	0.17	0.52
1	-8	3	0.19	0.24
1	-8	4	2.35	0.36
1	-8	4	2.35	0.55
1	-8	5	0.21	0.51
1	-8	5	0.29	0.25
1	-8	6	14.29	1.45
1	-8	7	0.03	0.59
0	7	7	0.44	0.38
0	7	6	8.22	1.57
0	7	6	6.43	0.81
0	-7	-6	6.32	0.65
0	7	5	0.81	0.34
0	7	5	0.81	0.69
0	-7	-5	0.50	0.24
0	7	4	19.63	1.43
0	7	4	20.12	2.06
0	-7	-4	17.88	1.42
0	7	3	1.60	0.68
0	7	3	1.11	0.29
0	-7	-3	0.98	0.35
0	-7	-3	0.58	0.30
0	-7	-3	0.53	0.26
0	7	2	21.85	2.08
0	7	2	22.01	1.58
0	-7	-2	20.06	1.61
0	7	1	1.22	0.65
0	7	1	1.00	0.32
0	-7	-1	1.02	0.61
0	-7	-1	0.94	0.31
0	7	0	28.34	2.48
0	7	0	28.18	2.11
0	-7	0	28.07	2.07
0	-7	0	29.42	2.19
0	7	-1	1.36	0.46
0	7	-1	1.02	0.58
0	-7	1	0.73	0.29
0	-7	1	1.01	0.41
0	7	-2	23.00	2.11
0	-7	2	23.17	1.71
0	-7	2	21.25	1.60
0	-7	2	21.63	1.66
0	7	-3	0.62	0.64
0	-7	3	0.71	0.29
0	-7	3	0.58	0.36
0	-7	3	0.70	0.25
0	-7	4	18.18	1.33
0	-7	4	17.58	1.41
0	-7	4	18.08	1.50
0	-7	5	0.08	0.22
0	-7	5	0.26	0.43
0	-7	6	7.88	1.03
0	-7	7	0.30	0.49
1	6	8	6.70	0.81
1	6	7	0.30	0.33
1	6	7	0.65	0.85
1	6	6	5.68	0.69
1	6	6	4.53	1.11
1	6	5	0.49	0.79
1	6	5	1.30	0.34
1	6	4	34.16	3.05
1	6	4	36.76	2.50
1	6	3	3.05	0.47
1	6	3	2.91	0.86
-1	-6	-3	2.77	0.41
-1	-6	-3	3.37	0.45
1	6	2	13.34	1.62
1	6	2	11.64	0.97
-1	-6	-2	11.46	1.01
1	6	1	5.02	0.99
1	6	1	5.56	0.69
-1	-6	-1	5.89	0.64
-1	-6	-1	6.45	0.81
1	6	0	67.38	4.77
1	6	0	66.75	4.76
1	6	0	72.06	5.18
-1	-6	0	73.88	4.84
-1	-6	0	67.73	4.75
1	6	-1	5.89	0.86
1	6	-1	4.46	0.94
-1	-6	1	6.45	0.76
-1	-6	1	5.64	0.63
1	6	-2	12.10	1.54
-1	-6	2	11.34	1.00
-1	-6	2	11.33	0.98
1	6	-3	2.68	0.80
-1	-6	3	4.18	0.56
-1	-6	3	3.45	0.48
-1	-6	4	35.69	2.59
-1	-6	4	36.74	2.59
-1	-6	5	1.44	0.41
-1	-6	5	0.99	0.26
-1	-6	5	1.06	0.33
-1	-6	6	6.46	0.83
1	6	-7	0.18	0.32
-1	-6	7	0.48	0.41
1	6	-8	5.08	0.69
2	5	8	4.04	0.53
2	5	8	6.18	1.43
2	5	7	0.01	0.31
2	5	7	0.08	1.01
2	5	6	12.29	1.05
2	5	5	-0.03	0.84
2	5	5	0.64	0.31
-2	-5	-4	13.24	0.90
-2	-5	-4	9.95	0.93
2	5	3	0.48	0.24
2	5	3	0.47	0.74
-2	-5	-3	0.34	0.25
-2	-5	-3	0.43	0.26
2	5	2	39.07	2.72
2	5	2	38.57	3.31
2	5	2	41.18	2.70
-2	-5	-2	38.18	2.77
-2	-5	-2	37.83	2.73
2	5	1	0.52	0.24
2	5	1	0.70	0.75
2	5	1	1.01	0.38
2	5	1	0.73	0.38
-2	-5	-1	0.54	0.51
-2	-5	-1	0.41	0.26
2	5	0	37.87	2.70
2	5	0	36.69	2.64
2	5	0	39.33	3.23
-2	-5	0	36.30	2.75
-2	-5	0	38.32	2.65
2	5	-1	0.73	0.46
2	5	-1	0.59	0.74
-2	-5	1	0.72	0.29
-2	-5	1	0.53	0.35
2	5	-2	39.05	3.22
-2	-5	2	35.37	2.67
-2	-5	2	40.31	2.72
2	5	-3	0.29	0.64
-2	-5	3	0.47	0.31
2	5	-4	23.28	2.25
2	5	-4	20.15	1.54
-2	-5	4	19.65	1.59

-2	-5	4	22.22	1.63
2	5	-5	0.81	0.26
-2	-5	5	0.30	0.33
-2	-5	5	0.43	0.33
2	5	-6	12.06	1.05
-2	-5	6	13.87	1.10
2	5	-7	0.25	0.28
-2	-5	7	0.61	0.34
2	5	-8	3.65	0.48
3	4	8	4.05	0.53
-3	-4	-8	3.15	1.69
3	4	7	0.38	0.34
3	4	7	1.15	1.00
3	4	6	13.64	1.13
3	4	5	1.65	0.93
3	4	5	1.16	0.34
-3	-4	-4	13.53	1.13
-3	-4	-4	14.79	1.05
3	4	3	3.08	0.51
3	4	3	3.06	0.39
3	4	3	4.66	1.05
-3	-4	-3	2.93	0.38
-3	-4	-3	2.58	0.39
3	4	2	37.00	2.56
3	4	2	36.37	2.53
3	4	2	32.48	2.51
3	4	2	42.62	3.22
-3	-4	-2	36.22	2.52
-3	-4	-2	32.30	2.57
3	4	1	5.12	0.75
3	4	1	5.25	1.09
3	4	1	5.46	0.67
-3	-4	-1	6.35	0.85
-3	-4	-1	4.90	0.63
3	4	0	52.18	3.61
3	4	0	51.48	4.14
3	4	0	52.52	3.61
-3	-4	0	50.05	3.63
-3	-4	0	50.88	3.57
3	4	-1	4.20	1.07
3	4	-1	4.21	0.65
3	4	-1	7.05	0.88
-3	-4	1	4.08	0.54
-3	-4	1	4.78	0.65
3	4	-2	36.83	2.62
3	4	-2	39.24	3.22
-3	-4	2	32.41	2.54
-3	-4	2	37.60	2.63
3	4	-3	2.51	0.39
3	4	-3	3.15	0.51
3	4	-3	3.34	0.91
-3	-4	3	3.61	0.45
-3	-4	3	3.38	0.50
3	4	-4	24.91	1.84
3	4	-4	26.42	2.54
-3	-4	4	25.58	1.96
-3	-4	4	25.13	1.85
3	4	-5	0.97	0.33
-3	-4	5	1.45	0.34
-3	-4	5	1.74	0.43
3	4	-6	14.02	1.17
-3	-4	6	14.49	1.15
3	4	-7	0.70	0.33
3	4	-8	3.49	0.52
4	3	8	4.52	0.58
4	3	8	5.02	1.25
-4	-3	-8	4.29	1.52
4	3	7	0.28	0.35
4	3	7	0.58	0.88
4	3	6	13.90	1.16
4	3	5	2.12	0.92
4	3	5	0.84	0.36
4	3	5	1.60	0.36
4	3	4	24.85	1.90
4	3	4	24.85	1.85
4	3	4	26.06	1.85
4	3	3	3.69	0.57
4	3	3	3.89	0.49
4	3	3	3.47	0.54
-4	-3	-3	2.58	0.37
-4	-3	-3	2.34	0.40
4	3	2	37.80	2.62
4	3	2	36.04	2.58
4	3	2	41.05	2.73
-4	-3	-2	34.25	2.56
-4	-3	-2	33.83	2.64
4	3	1	4.51	0.68
4	3	1	5.46	0.72
4	3	1	6.90	1.20
4	3	1	5.63	0.66
-4	-3	-1	4.78	0.64
4	3	0	48.26	3.66
4	3	0	47.97	4.16
4	3	0	48.59	3.59
4	3	0	55.41	3.65
-4	-3	0	52.31	3.61
-4	-3	0	55.98	3.63
4	3	-1	4.40	0.65
4	3	-1	4.55	0.59
4	3	-1	5.50	1.18
-4	-3	1	5.25	0.74
-4	-3	1	3.69	0.54
4	3	-2	33.94	2.56
4	3	-2	37.55	2.71
4	3	-2	35.19	3.29
-4	-3	2	34.84	2.54
-4	-3	2	38.78	2.66
4	3	-3	2.57	0.92
4	3	-3	3.09	0.56
4	3	-3	2.74	0.43
-4	-3	3	2.76	0.51
-4	-3	3	2.93	0.44
4	3	-4	24.54	1.81
4	3	-4	23.79	2.48
4	3	-4	24.54	1.93
-4	-3	4	24.77	1.93
-4	-3	4	23.77	1.77

4	3	-5	0.82	0.77
4	3	-5	1.21	0.37
-4	-3	5	1.23	0.33
-4	-3	5	1.22	0.45
4	3	-6	12.66	1.21
-4	-3	6	15.72	1.16
4	3	-7	0.40	0.38
4	3	-8	3.37	0.59
5	2	8	5.92	0.91
5	2	8	4.39	0.64
-5	-2	-8	2.82	1.32
5	2	7	0.28	0.42
5	2	7	0.14	0.43
5	2	6	12.53	1.19
5	2	6	12.04	1.17
5	2	6	13.71	1.16
5	2	5	0.33	0.39
5	2	5	-0.14	0.34
5	2	5	0.37	0.75
5	2	5	0.32	0.41
-5	-2	-4	12.96	1.10
-5	-2	-4	11.99	0.97
5	2	3	0.75	0.34
5	2	3	1.72	0.55
5	2	3	0.57	0.38
5	2	3	0.87	0.73
-5	-2	-3	0.52	0.30
5	2	2	88.83	6.70
5	2	2	85.18	6.36
-5	-2	-2	95.06	6.30
-5	-2	-2	97.53	6.36
5	2	1	1.95	0.86
5	2	1	0.55	0.40
5	2	1	0.93	0.48
5	2	1	1.28	0.58
-5	-2	-1	0.98	0.46
-5	-2	-1	1.74	0.46
-5	-2	-1	-0.02	0.31
5	2	0	39.78	3.24
5	2	0	48.79	3.43
-5	-2	0	43.49	3.28
-5	-2	0	53.02	3.20
-5	-2	0	42.74	3.27
5	2	-1	0.27	0.37
5	2	-1	0.07	0.85
5	2	-1	-0.35	0.51
-5	-2	1	0.62	0.34
-5	-2	1	0.57	0.42
5	2	-2	34.08	3.32
5	2	-2	39.21	2.81
5	2	-2	35.82	2.63
-5	-2	2	35.93	2.59
5	2	-3	0.49	0.50
5	2	-3	0.55	0.34
5	2	-3	0.37	0.41
5	2	-3	0.38	0.79
-5	-2	3	0.52	0.37
-5	-2	3	0.83	0.47
5	2	-4	16.86	2.12
5	2	-4	20.87	1.58
5	2	-4	19.98	1.71
5	2	-4	17.31	1.48
-5	-2	4	19.92	1.48
5	2	-5	0.80	0.75
5	2	-5	0.37	0.47
5	2	-5	-0.15	0.39
-5	-2	5	0.24	0.33
-5	-2	5	0.51	0.44
5	2	-6	12.39	1.33
5	2	-6	11.17	1.24
-5	-2	6	13.50	1.07
5	2	-7	0.40	0.49
5	2	-8	4.46	0.75
6	1	8	6.48	0.83
6	1	7	0.29	0.53
6	1	6	6.43	0.84
6	1	6	4.76	0.72
6	1	5	1.24	0.50
6	1	5	1.18	0.47
6	1	5	1.41	0.48
6	1	4	50.15	3.47
6	1	4	46.21	3.75
6	1	3	6.57	0.94
6	1	3	3.84	0.62
6	1	3	5.05	0.98
6	1	3	6.55	0.99
-6	-1	-3	5.77	0.81
6	1	2	27.26	2.19
6	1	2	25.96	2.54
6	1	2	24.28	2.08
-6	-1	-2	28.59	2.20
-6	-1	-2	26.82	2.09
6	1	1	13.60	1.33
6	1	1	13.82	1.46
6	1	1	12.11	1.41
6	1	1	16.43	1.92
-6	-1	-1	11.77	1.24
-6	-1	-1	14.95	1.34
-6	-1	-1	15.38	1.47
6	1	0	134.01	10.27
6	1	0	175.11	10.77
6	1	0	153.94	10.41
-6	-1	0	148.62	10.28
-6	-1	0	149.07	10.22
-6	-1	0	149.54	10.32
6	1	-1	11.98	1.13
6	1	-1	10.48	1.96
6	1	-1	8.92	1.38
-6	-1	1	11.44	1.20
-6	-1	1	14.15	1.23
-6	-1	1	8.89	0.91
6	1	-2	10.21	1.30
6	1	-2	7.86	1.44
-6	-1	2	11.39	0.95
6	1	-3	3.08	0.78
6	1	-3	4.29	0.58
6	1	-3	1.75	0.90

-6	-1	3	3.50	0.59
-6	-1	3	3.34	0.55
6	1	-4	33.18	2.56
6	1	-4	34.41	2.83
-6	-1	4	39.55	2.70
-6	-1	4	33.22	2.53
6	1	-5	1.27	0.51
6	1	-5	0.58	0.36
6	1	-5	0.96	0.58
-6	-1	5	0.77	0.46
-6	-1	5	1.43	0.94
-6	-1	5	1.35	0.42
6	1	-6	5.95	0.77
6	1	-6	5.01	0.90
-6	-1	6	5.84	0.81
6	1	-7	0.72	0.53
6	1	-7	0.61	0.59
6	1	-8	9.31	1.27
7	0	7	0.31	0.67
7	0	6	6.00	0.97
7	0	6	6.11	0.87
7	0	5	0.16	0.44
7	0	5	0.22	0.48
7	0	5	0.86	0.53
7	0	4	18.67	1.60
7	0	4	19.60	1.65
7	0	3	1.01	0.47
7	0	3	1.34	0.50
7	0	3	0.40	0.67
7	0	3	2.01	0.55
-7	0	-3	0.61	0.51
7	0	2	42.43	3.70
7	0	2	43.07	3.46
7	0	2	44.12	3.39
7	0	2	54.61	3.48
-7	0	-2	50.93	3.55
-7	0	-2	47.14	3.34
-7	0	-2	48.78	3.55
-7	0	-2	46.41	3.41
7	0	1	2.37	0.86
7	0	1	2.79	0.71
7	0	1	3.03	0.67
7	0	1	2.53	0.58
-7	0	-1	2.74	0.50
-7	0	-1	2.69	0.70
-7	0	-1	2.22	0.60
7	0	0	71.29	5.06
7	0	0	75.19	5.24
7	0	0	72.58	5.44
-7	0	0	71.66	5.13
-7	0	0	73.61	5.01
-7	0	0	68.24	5.03
7	0	-1	2.89	0.83
7	0	-1	3.29	1.08
7	0	-1	2.08	0.56
-7	0	1	2.50	0.56
-7	0	1	2.24	0.61
-7	0	1	2.68	0.48
7	0	-2	40.15	3.96
7	0	-2	50.51	3.43
7	0	-2	45.27	3.64
-7	0	2	54.08	3.49
-7	0	2	43.78	3.45
7	0	-3	1.85	0.53
7	0	-3	-0.54	0.78
7	0	-3	0.09	0.66
-7	0	3	1.82	0.55
-7	0	3	1.03	0.39
-7	0	3	1.13	0.55
7	0	-4	37.50	2.73
-7	0	4	42.57	2.88
-7	0	4	30.33	2.80
7	0	-5	0.48	0.40
7	0	-5	0.73	0.63
-7	0	5	0.29	0.49
7	0	-6	6.12	1.04
-7	0	6	5.19	0.71
7	0	-7	-0.29	0.59
-7	0	7	-0.40	0.50
8	-1	7	0.27	0.76
8	-1	6	10.50	1.30
8	-1	6	12.94	1.55
8	-1	5	0.15	0.48
8	-1	5	0.65	0.53
8	-1	5	0.37	0.50
8	-1	4	4.23	0.69
8	-1	4	2.18	0.56
8	-1	3	0.59	0.48
8	-1	3	1.82	0.64
8	-1	3	1.07	0.59
-8	1	-3	0.04	0.50
-8	1	-2	95.84	6.77
8	-1	1	0.70	0.63
8	-1	1	0.68	0.55
8	-1	1	1.03	0.79
8	-1	1	1.83	0.63
-8	1	-1	0.56	0.57
-8	1	-1	0.76	0.44
-8	1	-1	1.62	0.53
8	-1	0	5.42	0.79
8	-1	0	5.43	1.15
8	-1	0	5.26	0.94
-8	1	0	4.63	0.70
-8	1	0	4.78	0.79
-8	1	0	4.21	0.68
8	-1	-1	1.26	0.58
8	-1	-1	1.82	0.97
8	-1	-1	2.03	0.82
-8	1	1	1.12	0.46
-8	1	1	0.98	0.51
-8	1	1	1.56	0.57
8	-1	-2	116.98	7.97
8	-1	-2	123.14	8.24
8	-1	-2	118.38	8.48
-8	1	2	107.16	8.01
-8	1	2	113.48	8.00
8	-1	-3	1.08	0.85

8	-1	-3	1.02	0.53
-8	1	3	0.94	0.53
-8	1	3	0.62	0.42
-8	1	3	0.23	0.62
8	-1	-4	1.97	0.88
8	-1	-4	4.48	0.66
-8	1	4	2.46	0.48
8	-1	-5	-0.89	0.71
8	-1	-5	0.22	0.46
-8	1	5	0.37	0.42
8	-1	-6	8.11	1.37
-8	1	6	10.86	1.17
8	-1	-7	-1.20	0.60
-8	1	7	0.17	0.57
9	-2	6	12.39	1.53
9	-2	6	13.25	1.48
9	-2	5	-0.58	0.47
9	-2	5	0.91	0.84
9	-2	5	-0.18	0.60
9	-2	4	10.75	1.32
9	-2	4	12.14	1.75
9	-2	3	-0.08	0.53
9	-2	3	0.27	0.50
9	-2	3	0.66	0.78
-9	2	-3	0.02	0.50
9	-2	2	25.11	2.19
-9	2	-2	28.79	2.45
9	-2	1	0.76	0.65
9	-2	1	0.52	0.74
9	-2	1	0.92	0.67
9	-2	1	0.72	0.51
-9	2	-1	0.34	0.54
-9	2	-1	0.67	0.66
-9	2	-1	0.71	0.44
9	-2	0	19.22	1.95
9	-2	0	22.52	2.33
9	-2	0	14.60	1.69
-9	2	0	23.13	1.80
-9	2	0	18.97	1.84
-9	2	0	18.25	1.73
9	-2	-1	0.27	0.52
9	-2	-1	1.31	0.84
-9	2	1	0.52	0.60
-9	2	1	0.59	0.50
-9	2	1	1.10	0.61
9	-2	-2	36.90	3.27
9	-2	-2	50.78	3.76
-9	2	2	46.07	3.33
9	-2	-3	-0.06	0.55
9	-2	-3	0.67	0.94
-9	2	3	0.74	0.47
9	-2	-4	8.64	1.47
9	-2	-4	10.84	1.23
-9	2	4	8.97	1.33
9	-2	-5	0.19	0.87
9	-2	-5	0.47	0.56
-9	2	5	-0.70	0.58
9	-2	-6	3.92	1.14
10	-3	5	2.67	1.05
10	-3	5	0.81	0.69
10	-3	4	13.10	1.53
10	-3	3	1.63	0.60
10	-3	3	3.26	0.70
10	-3	2	6.82	1.14
10	-3	2	5.97	0.88
10	-3	1	5.33	0.86
10	-3	1	5.60	1.08
10	-3	1	7.50	1.22
-10	3	-1	9.54	1.22
10	-3	0	32.30	2.85
-10	3	0	36.06	2.78
10	-3	-1	6.24	0.93
-10	3	1	9.59	1.15
10	-3	-2	13.15	1.92
-10	3	2	11.25	1.66
10	-3	-3	7.75	1.44
-10	3	3	6.66	1.00
10	-3	-4	20.80	2.31
10	-3	-4	14.12	1.70
-10	3	4	18.51	1.80
-10	3	5	2.22	0.85
11	-4	3	0.95	1.10
11	-4	3	-0.78	0.57
11	-4	2	3.16	0.74
11	-4	2	7.56	1.40
11	-4	1	-0.47	0.54
11	-4	1	0.33	0.75
11	-4	0	16.70	1.84
-11	4	0	15.61	1.95
11	-4	-1	0.63	0.89
11	-4	-1	-0.85	0.53
-11	4	1	0.16	0.86
11	-4	-2	8.91	2.26
-11	4	2	7.61	1.19
11	-4	-3	-0.56	0.55
-11	4	3	-1.23	0.82
-3	11	2	2.01	0.67
3	-11	-2	1.35	0.42
3	-11	-1	-0.05	0.33
3	-11	0	7.33	0.85
3	-11	1	0.23	0.84
3	-11	1	0.46	0.34
3	-11	2	1.88	0.83
3	-11	2	1.47	0.42
-2	10	4	13.32	1.35
2	-10	-4	12.10	1.11
-2	10	3	0.13	0.52
2	-10	-3	0.04	0.30
-2	10	2	0.80	0.54
-2	10	2	0.15	0.61
2	-10	-2	-0.11	0.31
-2	10	1	0.10	0.47
-2	10	1	0.92	0.63
2	-10	-1	0.00	0.29
-2	10	0	25.55	2.23
2	-10	0	22.28	2.22
2	-10	0	21.89	1.74

2	-10	1	0.07	0.70
2	-10	1	0.22	0.31
2	-10	2	0.11	0.30
2	-10	2	0.60	0.71
2	-10	3	-0.01	0.69
2	-10	3	-0.15	0.31
2	-10	4	13.80	1.64
2	-10	4	12.59	1.12
-1	9	5	1.12	0.36
1	-9	-5	0.51	0.33
-1	9	4	7.85	1.03
-1	9	4	8.77	1.41
1	-9	-4	6.85	0.78
-1	9	3	1.97	0.74
-1	9	3	2.22	0.53
1	-9	-3	1.91	0.37
-1	9	2	6.58	0.95
-1	9	2	7.53	1.34
1	-9	-2	6.40	0.72
-1	9	1	3.27	0.82
-1	9	1	3.21	0.53
1	-9	-1	3.13	0.43
-1	9	0	13.23	1.60
-1	9	0	13.53	1.37
1	-9	0	12.78	1.07
1	-9	0	13.61	1.41
-1	9	-1	3.21	0.84
1	-9	1	3.44	0.68
1	-9	1	3.13	0.43
-1	9	-2	6.29	1.34
1	-9	2	6.00	0.86
1	-9	2	6.19	0.70
1	-9	3	2.12	0.67
1	-9	3	2.20	0.39
1	-9	4	7.78	1.02
1	-9	4	6.94	0.77
1	-9	5	0.83	0.34
1	-9	5	0.72	0.60
0	8	6	8.79	1.02
0	-8	-6	8.59	0.86
0	8	5	1.35	0.37
0	8	5	2.31	0.86
0	-8	-5	1.38	0.33
0	8	4	1.54	0.44
0	8	4	2.42	0.83
0	-8	-4	1.35	0.33
0	8	3	5.55	0.85
0	8	3	7.16	1.43
0	-8	-3	5.92	0.79
0	-8	-3	5.63	0.67
0	8	2	34.40	2.88
0	8	2	33.24	2.34
0	-8	-2	29.99	2.30
0	-8	-2	31.30	2.37
0	8	1	8.40	1.44
0	8	1	8.11	0.89
0	-8	-1	8.93	1.25
0	-8	-1	8.43	0.90
0	-8	-1	8.63	0.79
0	8	0	2.31	0.49
0	8	0	2.12	0.80
0	8	0	1.98	0.46
0	-8	0	1.66	0.34
0	-8	0	1.92	0.39
0	-8	0	2.26	0.60
0	8	-1	8.93	1.49
0	-8	1	8.71	0.88
0	-8	1	8.33	1.06
0	-8	1	8.13	0.78
0	8	-2	35.25	2.95
0	-8	2	30.62	2.32
0	-8	2	33.16	2.48
0	8	-3	5.37	1.10
0	-8	3	6.06	0.94
0	-8	3	4.79	0.61
0	-8	4	1.70	0.49
0	-8	4	1.81	0.34
0	-8	5	1.53	0.34
0	-8	5	1.95	0.60
0	-8	6	10.46	1.34
1	7	7	0.42	0.45
1	7	6	12.46	1.21
1	7	6	14.16	2.14
-1	-7	-6	11.73	1.06
1	7	5	0.58	0.82
1	7	5	0.13	0.36
1	7	4	1.93	0.37
1	7	4	2.81	0.94
1	7	3	0.21	0.33
1	7	3	1.12	0.77
-1	-7	-3	0.10	0.30
-1	-7	-3	0.14	0.32
1	7	2	46.08	3.29
1	7	2	49.57	3.93
-1	-7	-2	46.65	3.34
1	7	1	0.58	0.34
1	7	1	0.10	0.75
-1	-7	-1	-0.01	0.57
-1	-7	-1	0.49	0.31
1	7	0	2.21	0.50
1	7	0	1.68	0.79
-1	-7	0	1.54	0.49
-1	-7	0	1.55	0.37
1	7	-1	1.19	0.78
-1	-7	1	0.28	0.39
-1	-7	1	0.32	0.31
1	7	-2	45.03	3.67
-1	-7	2	44.24	3.18
-1	-7	2	44.92	3.17
1	7	-3	-0.14	0.70
-1	-7	3	0.33	0.35
-1	-7	4	2.05	0.36
-1	-7	4	2.52	0.54
-1	-7	5	0.42	0.48
-1	-7	5	0.23	0.26
-1	-7	6	13.37	1.33
-1	-7	7	0.25	0.54

2	6	7	2.07	1.11
2	6	7	1.53	0.48
2	6	6	10.47	1.12
2	6	6	14.64	2.20
2	6	5	3.86	1.18
2	6	5	4.78	0.74
2	6	4	3.98	1.08
-2	-6	-4	3.08	0.50
2	6	3	13.01	1.03
2	6	3	12.01	1.98
-2	-6	-3	12.73	1.10
-2	-6	-3	10.53	1.05
2	6	2	37.91	2.83
2	6	2	44.35	3.61
-2	-6	-2	40.28	2.88
2	6	1	21.63	1.63
2	6	1	23.25	2.45
2	6	1	20.62	1.63
-2	-6	-1	21.24	1.63
-2	-6	-1	20.53	1.83
2	6	0	6.49	0.94
2	6	0	5.87	1.21
-2	-6	0	7.53	0.94
-2	-6	0	7.02	0.76
2	6	-1	20.04	2.27
-2	-6	1	20.96	1.65
-2	-6	1	21.52	1.62
2	6	-2	47.37	3.74
-2	-6	2	41.00	3.07
-2	-6	2	42.78	3.09
2	6	-3	13.65	1.88
-2	-6	3	12.46	1.15
-2	-6	3	13.68	1.16
2	6	-4	4.82	1.14
-2	-6	4	5.73	0.85
-2	-6	5	5.45	0.86
-2	-6	6	12.14	1.19
-2	-6	7	1.34	0.49
3	5	7	0.50	0.44
3	5	7	0.53	1.18
3	5	6	8.10	1.51
3	5	6	4.58	0.62
3	5	5	1.69	0.42
3	5	4	25.94	1.88
3	5	3	7.46	1.34
3	5	3	5.31	0.69
-3	-5	-3	5.80	0.72
3	5	2	5.85	1.26
3	5	2	7.42	0.79
3	5	2	7.64	0.81
-3	-5	-2	7.82	0.76
3	5	1	10.99	1.85
3	5	1	10.57	0.97
3	5	1	10.96	1.05
-3	-5	-1	13.50	1.16
-3	-5	-1	9.89	0.99
3	5	0	59.05	3.95
3	5	0	57.24	3.91
3	5	0	58.12	4.63
-3	-5	0	52.83	3.90
-3	-5	0	53.40	3.94
3	5	-1	14.41	1.11
3	5	-1	9.27	1.89
-3	-5	1	11.30	1.08
-3	-5	1	10.87	1.10
3	5	-2	6.48	1.31
3	5	-2	7.95	0.83
-3	-5	2	7.34	0.80
-3	-5	2	8.18	0.88
3	5	-3	5.19	1.16
-3	-5	3	6.55	0.85
-3	-5	3	5.94	0.76
3	5	-4	28.72	2.88
-3	-5	4	28.05	2.05
-3	-5	4	26.08	2.11
-3	-5	5	2.61	0.48
-3	-5	6	5.53	0.83
-3	-5	7	0.97	0.45
4	4	7	-0.25	0.42
4	4	7	0.05	1.06
4	4	6	2.73	1.12
4	4	5	-0.05	0.37
4	4	5	0.51	0.94
4	4	4	57.52	3.97
4	4	4	57.50	3.96
-4	-4	-3	0.08	0.27
4	4	2	0.83	0.36
4	4	2	0.83	0.41
4	4	2	1.99	0.99
-4	-4	-2	2.08	0.57
-4	-4	-2	0.68	0.30
4	4	1	0.23	0.85
4	4	1	0.23	0.36
4	4	1	-0.11	0.38
-4	-4	-1	0.53	0.38
-4	-4	-1	-0.35	0.27
4	4	0	134.42	8.45
4	4	0	120.45	8.47
4	4	0	119.22	8.99
-4	-4	0	121.88	8.40
-4	-4	0	124.26	8.43
4	4	-1	-1.16	0.88
4	4	-1	-0.23	0.38
-4	-4	1	-0.15	0.34
-4	-4	1	0.05	0.34
4	4	-2	0.39	0.38
4	4	-2	0.24	0.91
-4	-4	2	0.51	0.39
-4	-4	2	0.30	0.32
4	4	-3	1.44	0.98
4	4	-3	-0.10	0.37
-4	-4	3	0.12	0.40
-4	-4	3	-0.04	0.35
4	4	-4	56.25	4.64
4	4	-4	53.51	3.99
-4	-4	4	58.00	4.00
-4	-4	4	54.43	3.85

4	4	-5	-0.07	0.83
-4	-4	5	0.04	0.37
-4	-4	6	0.97	0.40
-4	-4	7	-0.06	0.40
5	3	7	1.10	0.56
5	3	7	0.42	0.50
5	3	7	0.30	1.04
5	3	6	8.40	1.78
5	3	6	4.56	0.68
5	3	6	4.00	0.64
5	3	5	2.30	0.49
5	3	5	1.87	0.48
5	3	5	1.84	0.49
5	3	4	26.94	2.04
5	3	4	25.47	1.94
5	3	3	6.26	0.83
5	3	3	6.21	0.77
-5	-3	-3	4.83	0.71
5	3	2	9.60	0.94
5	3	2	8.19	0.95
-5	-3	-2	7.30	0.84
5	3	1	11.54	1.23
5	3	1	11.42	1.15
5	3	1	10.29	1.08
-5	-3	-1	12.72	1.16
-5	-3	-1	9.49	1.04
5	3	0	50.71	3.98
5	3	0	50.51	3.89
-5	-3	0	55.83	3.92
-5	-3	0	63.59	3.84
5	3	-1	9.02	1.12
5	3	-1	9.87	0.97
5	3	-1	9.03	1.59
-5	-3	1	9.52	1.05
-5	-3	1	9.51	0.96
5	3	-2	6.19	1.39
5	3	-2	5.86	0.80
5	3	-2	7.07	1.07
-5	-3	2	9.86	1.04
-5	-3	2	7.45	0.82
5	3	-3	7.03	0.82
-5	-3	3	7.00	0.86
-5	-3	3	6.06	0.88
5	3	-4	21.66	2.77
5	3	-4	28.42	2.24
-5	-3	4	31.60	2.24
-5	-3	4	26.29	2.03
5	3	-5	1.39	0.93
5	3	-5	1.29	0.58
-5	-3	5	2.23	0.47
-5	-3	6	4.15	0.57
-5	-3	7	1.00	0.45
6	2	7	1.50	0.62
6	2	7	1.47	1.01
6	2	6	11.34	1.28
6	2	6	10.10	1.16
6	2	5	4.20	0.67
6	2	5	4.97	0.64
6	2	4	4.87	0.66
6	2	4	6.39	0.74
-6	-2	-4	3.13	0.60
6	2	3	31.50	2.48
6	2	3	28.24	2.84
-6	-2	-3	32.68	2.52
6	2	2	105.30	7.66
6	2	2	110.77	8.05
-6	-2	-2	116.22	7.88
-6	-2	-2	109.87	7.62
6	2	1	47.10	3.28
6	2	1	34.99	3.06
-6	-2	-1	45.51	3.17
6	2	0	9.27	1.16
6	2	0	12.58	1.45
6	2	0	16.61	2.22
-6	-2	0	12.18	1.10
-6	-2	0	11.02	1.19
-6	-2	0	11.60	1.21
6	2	-1	27.85	2.23
6	2	-1	26.67	2.05
-6	-2	1	25.01	1.89
-6	-2	1	24.93	2.04
6	2	-2	39.32	3.14
6	2	-2	42.81	3.83
6	2	-2	45.12	2.98
-6	-2	2	35.62	2.86
-6	-2	2	40.98	3.00
6	2	-3	10.45	1.38
6	2	-3	7.49	1.55
6	2	-3	11.87	1.10
-6	-2	3	11.72	1.24
-6	-2	3	11.68	1.12
6	2	-4	4.31	0.59
6	2	-4	4.83	0.90
6	2	-4	2.88	1.07
-6	-2	4	3.85	0.72
-6	-2	4	4.65	0.65
6	2	-5	3.91	0.56
6	2	-5	3.43	0.79
6	2	-5	2.53	1.01
-6	-2	5	4.47	0.61
6	2	-6	10.93	1.43
-6	-2	6	12.34	1.21
-6	-2	7	1.29	0.51
7	1	7	0.47	0.71
7	1	6	13.13	1.53
7	1	6	13.71	1.93
7	1	5	-0.43	0.43
7	1	5	0.58	0.51
7	1	5	-0.73	0.76
7	1	4	4.80	0.78
7	1	4	5.45	0.77
7	1	4	4.06	1.03
7	1	3	0.33	0.80
7	1	3	1.21	0.56
7	1	3	1.20	0.64
7	1	3	0.10	0.50
-7	-1	-3	0.28	0.56

7	1	2	116.60	8.01
7	1	2	112.47	7.94
7	1	2	107.27	8.26
-7	-1	-2	124.34	8.00
7	1	1	1.05	0.68
7	1	1	1.47	0.55
7	1	1	0.92	0.55
7	1	1	1.58	0.97
-7	-1	-1	0.35	0.55
-7	-1	-1	0.57	0.42
-7	-1	-1	1.29	0.49
7	1	0	6.04	0.81
7	1	0	4.72	0.93
7	1	0	5.46	1.26
7	1	0	8.11	1.05
-7	-1	0	4.07	0.67
-7	-1	0	3.85	0.71
-7	-1	0	4.53	0.65
7	1	-1	2.93	1.16
7	1	-1	1.32	0.54
7	1	-1	0.51	0.69
-7	-1	1	0.98	0.50
-7	-1	1	1.20	0.38
-7	-1	1	0.74	0.52
7	1	-2	101.15	6.24
7	1	-2	83.18	6.38
7	1	-2	92.86	6.93
-7	-1	2	80.85	6.22
7	1	-3	0.95	0.49
7	1	-3	-1.42	0.83
7	1	-3	-0.47	0.69
-7	-1	3	0.44	0.55
-7	-1	3	0.42	0.39
-7	-1	3	-0.25	0.50
7	1	-4	3.30	0.56
7	1	-4	-0.19	1.00
7	1	-4	1.22	0.73
-7	-1	4	1.78	0.56
7	1	-5	0.18	0.39
7	1	-5	-0.26	0.63
-7	-1	5	0.52	0.54
7	1	-6	8.96	1.52
-7	-1	6	12.60	1.33
-7	-1	7	0.39	0.58
8	0	6	9.08	1.19
8	0	5	2.36	0.72
8	0	5	0.18	0.85
8	0	5	2.05	0.58
8	0	5	4.59	0.73
8	0	4	1.75	0.88
8	0	4	4.11	0.75
8	0	4	3.48	0.69
8	0	4	2.80	0.79
8	0	3	11.23	1.30
8	0	3	8.20	1.63
8	0	3	13.12	1.32
8	0	3	11.07	1.46
-8	0	-3	11.52	1.43
8	0	2	77.15	5.63
8	0	2	81.08	5.54
8	0	2	78.45	5.56
8	0	2	74.77	5.90
-8	0	-2	82.69	5.64
8	0	1	22.46	2.61
8	0	1	22.76	2.20
8	0	1	26.98	2.02
8	0	1	23.22	2.02
-8	0	-1	22.07	1.88
-8	0	-1	22.00	2.05
-8	0	-1	24.06	1.93
8	0	0	8.11	1.13
8	0	0	6.48	1.27
8	0	0	4.57	0.80
-8	0	0	7.95	1.02
-8	0	0	4.38	0.80
-8	0	0	6.37	0.95
8	0	-1	23.04	1.98
8	0	-1	23.64	2.25
8	0	-1	28.68	2.81
-8	0	1	22.77	1.93
-8	0	1	20.52	1.97
-8	0	1	20.18	1.83
8	0	-2	86.34	6.36
8	0	-2	79.53	5.87
8	0	-2	83.36	5.65
-8	0	2	79.83	5.65
-8	0	2	71.76	5.66
8	0	-3	8.89	1.30
-8	0	3	7.10	0.82
8	0	-4	4.28	0.67
8	0	-4	2.01	0.90
-8	0	4	2.35	0.52
8	0	-5	3.46	0.62
8	0	-5	1.27	0.88
-8	0	5	1.41	0.56
8	0	-6	4.68	1.15
-8	0	6	7.82	1.20
9	-1	5	1.89	0.69
9	-1	5	0.86	0.55
9	-1	5	1.47	0.89
9	-1	4	10.04	1.12
9	-1	4	7.45	1.26
9	-1	3	4.49	0.79
9	-1	3	5.25	0.95
9	-1	3	2.78	0.96
9	-1	3	5.38	0.83
-9	1	-3	4.08	0.98
9	-1	2	12.98	1.88
9	-1	2	16.04	1.60
9	-1	2	16.35	1.57
-9	1	-2	15.46	1.74
9	-1	1	8.97	1.27
9	-1	1	9.54	1.94
9	-1	1	10.94	1.58
9	-1	1	13.28	1.44
-9	1	-1	9.10	1.40
-9	1	-1	10.64	1.27

9	-1	0	39.22	3.35
9	-1	0	32.84	2.84
9	-1	0	38.14	3.08
-9	1	0	34.49	2.78
-9	1	0	34.81	2.89
-9	1	0	40.74	2.83
9	-1	-1	10.45	1.64
9	-1	-1	9.21	1.25
9	-1	-1	12.14	2.25
-9	1	1	8.23	1.52
-9	1	1	8.25	1.32
-9	1	1	12.30	1.43
9	-1	-2	19.71	2.13
9	-1	-2	17.20	1.69
-9	1	2	15.79	1.72
9	-1	-3	5.18	1.14
9	-1	-3	5.16	0.79
-9	1	3	4.44	0.64
9	-1	-4	15.52	2.12
9	-1	-4	17.27	1.58
-9	1	4	12.31	1.56
9	-1	-5	0.40	1.00
-9	1	5	1.54	0.59
10	-2	4	14.01	1.39
10	-2	4	8.97	1.73
10	-2	3	0.47	0.59
10	-2	3	-0.97	0.73
10	-2	3	-0.29	0.65
10	-2	3	1.17	0.91
10	-2	2	-0.08	0.80
10	-2	2	-0.11	0.64
10	-2	2	1.24	0.67
10	-2	2	1.98	0.84
10	-2	1	0.97	0.76
10	-2	1	0.31	0.66
10	-2	1	0.62	0.85
10	-2	0	54.21	4.55
10	-2	0	67.19	4.83
-10	2	0	68.91	4.65
10	-2	-1	-0.05	0.59
10	-2	-1	1.58	0.97
-10	2	1	0.31	0.73
10	-2	-2	0.06	0.60
10	-2	-2	1.33	0.97
-10	2	2	1.43	0.78
10	-2	-3	0.97	1.00
10	-2	-3	-0.21	0.61
-10	2	3	0.92	0.75
10	-2	-4	26.21	2.27
-10	2	4	27.23	2.21
11	-3	2	1.72	0.74
11	-3	2	1.79	0.91
11	-3	1	0.83	0.69
11	-3	0	13.14	1.52
11	-3	-1	0.11	0.63
-11	3	1	0.82	0.88
11	-3	-2	2.49	0.78
-11	3	2	3.11	0.92
-1	10	3	0.50	0.77
-1	10	3	0.64	0.57
1	-10	-3	0.96	0.36
-1	10	2	0.71	0.60
-1	10	2	1.14	0.77
1	-10	-2	1.02	0.37
-1	10	1	1.51	0.56
-1	10	1	1.90	0.85
1	-10	-1	0.61	0.36
-1	10	0	11.78	1.73
1	-10	0	10.61	1.00
-1	10	-1	0.89	0.81
1	-10	1	1.38	0.38
1	-10	1	1.57	0.79
1	-10	2	0.45	0.36
1	-10	2	0.78	0.75
1	-10	3	0.69	0.74
1	-10	3	0.36	0.35
0	9	4	7.93	1.13
0	9	4	11.77	1.77
0	-9	-4	7.70	0.84
0	9	3	0.46	0.79
0	9	3	0.05	0.49
0	-9	-3	0.01	0.30
0	9	2	2.59	0.94
0	9	2	4.14	0.64
0	-9	-2	1.97	0.41
0	9	1	0.40	0.42
0	9	1	0.77	0.81
0	-9	-1	0.15	0.31
0	9	0	16.16	1.96
0	-9	0	13.31	1.18
0	-9	0	14.40	1.51
0	9	-1	0.10	0.74
0	-9	1	-0.30	0.29
0	-9	1	0.30	0.65
0	9	-2	1.57	0.88
0	-9	2	2.11	0.40
0	-9	2	2.70	0.74
0	-9	3	0.00	0.31
0	-9	3	0.53	0.66
0	-9	4	7.61	1.14
0	-9	4	7.12	0.81
1	8	5	0.40	0.36
1	8	5	1.70	0.96
-1	-8	-5	0.84	0.37
1	8	4	8.48	1.84
1	8	4	7.19	1.01
-1	-8	-4	7.11	0.81
1	8	3	2.40	0.97
1	8	3	1.85	0.53
1	8	2	8.11	1.63
1	8	2	7.62	0.97
-1	-8	-2	6.26	0.88
1	8	1	3.65	1.07
1	8	1	4.41	0.59
1	8	1	4.01	0.57
-1	-8	-1	2.75	0.65
1	8	0	13.11	1.89

-1	-8	0	14.00	1.41
1	8	-1	3.30	1.01
-1	-8	1	3.33	0.69
1	8	-2	6.27	1.28
-1	-8	2	6.59	0.88
1	8	-3	2.44	0.99
-1	-8	3	1.91	0.40
-1	-8	3	2.23	0.64
-1	-8	4	6.72	0.76
-1	-8	4	6.70	0.93
-1	-8	5	0.94	0.35
-1	-8	5	1.56	0.65
2	7	6	5.24	0.66
2	7	6	4.91	1.36
2	7	5	-0.24	0.32
2	7	5	-0.89	0.93
2	7	4	4.02	1.19
2	7	4	3.44	0.57
2	7	3	-0.02	0.86
2	7	3	0.23	0.43
2	7	2	16.98	1.36
2	7	2	14.88	1.37
2	7	2	16.17	2.20
-2	-7	-2	16.20	1.37
2	7	1	0.36	0.36
2	7	1	-0.03	0.86
-2	-7	-1	0.30	0.34
-2	-7	-1	-0.40	0.47
2	7	0	5.80	1.30
2	7	0	6.91	1.08
-2	-7	0	7.73	0.85
-2	-7	0	6.99	1.01
2	7	-1	-0.09	0.87
-2	-7	1	0.06	0.46
-2	-7	1	0.57	0.47
2	7	-2	16.06	2.11
-2	-7	2	15.44	1.48
2	7	-3	-0.38	0.80
-2	-7	3	0.08	0.44
2	7	-4	3.19	1.14
-2	-7	4	4.28	0.61
-2	-7	5	0.05	0.32
-2	-7	5	0.31	0.55
-2	-7	6	7.16	1.14
3	6	6	6.13	0.96
3	6	5	0.31	1.05
3	6	5	-0.01	0.32
3	6	4	6.39	1.39
3	6	4	3.91	0.60
3	6	3	1.21	0.49
3	6	3	1.85	0.48
3	6	2	23.45	2.68
3	6	2	22.45	1.74
-3	-6	-2	22.59	1.76
3	6	1	0.43	0.38
3	6	1	0.14	0.99
-3	-6	-1	0.24	0.34
-3	-6	-1	0.16	0.37
3	6	0	6.56	1.01
3	6	0	5.25	1.34
-3	-6	0	6.03	0.92
-3	-6	0	5.19	0.79
3	6	-1	-0.07	0.96
-3	-6	1	-0.19	0.40
-3	-6	1	0.17	0.35
3	6	-2	23.82	2.76
-3	-6	2	19.98	1.96
-3	-6	2	23.19	1.81
3	6	-3	-0.17	0.88
-3	-6	3	-0.30	0.34
3	6	-4	3.66	1.24
-3	-6	4	4.31	0.64
-3	-6	5	0.37	0.51
-3	-6	6	7.37	1.09
4	5	6	2.68	0.59
4	5	5	0.48	1.00
4	5	5	0.40	0.35
4	5	5	-0.32	0.32
4	5	4	11.90	1.21
4	5	4	11.49	1.19
4	5	3	0.35	0.42
4	5	3	-0.41	1.00
4	5	2	9.31	1.01
-4	-5	-2	10.00	1.15
4	5	1	0.66	0.42
4	5	1	-0.04	0.37
4	5	1	-1.16	0.89
-4	-5	-1	-0.13	0.31
-4	-5	-1	0.00	0.33
4	5	0	20.52	1.69
4	5	0	24.06	1.80
4	5	0	17.66	2.68
-4	-5	0	20.79	1.65
-4	-5	0	18.64	1.65
4	5	-1	-0.05	0.38
4	5	-1	0.07	1.00
-4	-5	1	-0.41	0.36
-4	-5	1	-0.05	0.39
4	5	-2	9.97	1.08
4	5	-2	7.75	1.59
-4	-5	2	10.44	1.08
-4	-5	2	9.14	1.08
4	5	-3	-0.82	0.89
-4	-5	3	-0.32	0.34
4	5	-4	8.50	1.67
-4	-5	4	12.54	1.19
4	5	-5	-0.15	0.92
-4	-5	5	0.06	0.48
-4	-5	6	3.55	0.63
5	4	6	2.96	0.61
5	4	6	2.89	0.61
5	4	5	0.84	0.37
5	4	5	-0.45	1.04
5	4	4	11.69	1.24
5	4	3	0.42	0.95
5	4	3	0.06	0.46
5	4	2	9.05	1.03

5	4	2	10.26	1.10
5	4	2	6.28	1.02
5	4	1	-0.49	0.42
5	4	1	-0.16	0.42
5	4	1	-0.85	0.39
5	4	1	-0.35	1.00
-5	-4	-1	-0.15	0.33
-5	-4	-1	0.31	0.37
-5	-4	-1	-0.17	0.43
5	4	0	18.67	1.80
-5	-4	0	20.97	1.74
-5	-4	0	23.99	1.64
5	4	-1	-0.15	0.47
5	4	-1	-1.01	1.01
-5	-4	1	-0.36	0.40
-5	-4	1	-0.39	0.36
5	4	-2	8.37	1.70
5	4	-2	8.35	1.10
-5	-4	2	9.97	1.07
-5	-4	2	8.95	1.08
5	4	-3	-0.31	0.49
5	4	-3	-1.24	0.85
-5	-4	3	-0.03	0.34
5	4	-4	11.86	1.33
5	4	-4	7.97	1.65
-5	-4	4	10.94	1.18
5	4	-5	0.41	0.96
-5	-4	5	0.16	0.48
-5	-4	6	4.35	0.69
6	3	6	7.08	0.98
6	3	5	-0.10	0.44
6	3	5	0.29	1.00
6	3	5	-0.45	0.47
6	3	4	3.28	0.66
6	3	4	4.04	0.73
6	3	3	1.37	1.00
6	3	3	0.09	0.52
6	3	3	1.00	0.57
6	3	2	25.06	2.04
6	3	2	23.33	1.93
6	3	1	0.09	1.06
6	3	1	-0.33	0.47
6	3	1	-0.33	0.54
6	3	1	0.24	0.48
-6	-3	-1	0.87	0.43
-6	-3	-1	-0.31	0.45
-6	-3	-1	-0.63	0.35
6	3	0	6.98	1.10
6	3	0	5.85	0.80
-6	-3	0	6.31	0.89
-6	-3	0	7.36	1.00
6	3	-1	-0.20	1.08
6	3	-1	-0.40	0.45
6	3	-1	-0.40	0.52
-6	-3	1	0.58	0.33
-6	-3	1	0.00	0.44
-6	-3	1	0.06	0.38
6	3	-2	24.85	2.99
6	3	-2	17.80	1.70
6	3	-2	22.21	1.97
-6	-3	2	19.56	1.73
-6	-3	2	19.31	1.66
6	3	-3	-1.09	0.98
6	3	-3	-0.80	0.56
6	3	-3	-0.69	0.39
-6	-3	3	0.12	0.38
6	3	-4	3.26	0.79
6	3	-4	1.80	1.13
-6	-3	4	4.68	0.69
6	3	-5	-0.09	0.93
6	3	-5	-0.49	0.58
-6	-3	5	0.57	0.57
-6	-3	6	8.16	1.20
7	2	6	4.42	0.71
7	2	6	6.49	0.86
7	2	5	-0.46	0.52
7	2	5	0.13	1.02
7	2	5	0.00	0.65
7	2	4	8.19	1.13
7	2	4	4.64	0.87
7	2	3	-0.40	0.58
7	2	3	1.57	0.68
7	2	3	0.51	0.57
7	2	3	0.16	0.93
-7	-2	-3	1.14	0.61
7	2	2	32.33	2.76
7	2	2	41.01	3.45
7	2	1	1.00	0.54
7	2	1	0.94	1.04
7	2	1	0.50	0.69
7	2	1	0.88	0.54
-7	-2	-1	0.30	0.45
-7	-2	-1	1.72	0.51
-7	-2	-1	0.42	0.53
7	2	0	20.45	2.65
7	2	0	16.54	1.78
7	2	0	14.80	1.56
-7	-2	0	14.35	1.52
-7	-2	0	15.74	1.52
-7	-2	0	17.37	1.41
7	2	-1	-0.16	0.64
7	2	-1	1.64	1.23
7	2	-1	0.48	0.52
-7	-2	1	0.11	0.52
-7	-2	1	-0.01	0.45
-7	-2	1	0.76	0.41
7	2	-2	22.11	2.02
-7	-2	2	17.00	1.56
-7	-2	2	21.58	1.75
7	2	-3	-0.15	0.46
7	2	-3	-0.86	1.05
7	2	-3	-0.66	0.64
-7	-2	3	-0.17	0.35
7	2	-4	4.47	0.66
7	2	-4	2.62	0.93
-7	-2	4	3.47	0.70
7	2	-5	-0.72	0.42
7	2	-5	-1.00	0.64
-7	-2	5	0.12	0.61
-7	-2	6	5.79	0.88
8	1	5	1.46	1.06
8	1	5	2.83	0.69
8	1	5	0.75	0.79
8	1	4	15.01	2.34
8	1	4	16.41	1.69
8	1	4	18.93	1.80
8	1	3	3.50	0.88
8	1	3	4.69	1.18
8	1	3	7.03	1.17
8	1	3	5.61	0.87
-8	-1	-3	5.77	1.11
8	1	2	18.02	1.86
8	1	2	15.73	1.64
8	1	2	16.13	1.64
8	1	2	18.07	2.42
8	1	1	11.26	1.28
8	1	1	10.13	1.94
8	1	1	9.07	1.25
8	1	1	8.71	1.43
-8	-1	-1	8.30	1.26
-8	-1	-1	11.17	1.18
8	1	0	34.36	2.69
8	1	0	34.42	2.87
-8	-1	0	32.46	2.61
-8	-1	0	34.28	2.56
-8	-1	0	33.80	2.70
8	1	-1	9.80	1.79
8	1	-1	7.39	1.19
8	1	-1	10.90	1.28
-8	-1	1	9.01	1.25
-8	-1	1	7.47	1.13
-8	-1	1	6.65	1.15
8	1	-2	17.25	2.78
8	1	-2	12.21	1.71
-8	-1	2	11.26	1.35
8	1	-3	2.30	0.91
8	1	-3	4.01	1.49
8	1	-3	4.56	0.71
-8	-1	3	2.34	0.44
8	1	-4	8.69	1.37
-8	-1	4	7.17	1.06
8	1	-5	-1.02	1.18
-8	-1	5	1.39	0.64
9	0	4	14.07	2.12
9	0	4	17.29	1.75
9	0	4	15.44	1.95
9	0	4	19.48	1.77
9	0	3	0.02	0.87
9	0	3	0.98	0.74
9	0	3	1.12	0.82
9	0	3	0.95	0.67
9	0	2	7.01	0.99
9	0	2	5.66	1.30
9	0	2	9.49	1.45
9	0	2	6.84	1.00
9	0	1	1.21	0.80
9	0	1	0.66	0.64
9	0	1	0.44	0.93
-9	0	-1	0.60	0.63
-9	0	-1	0.73	0.87
9	0	0	47.85	3.96
9	0	0	38.45	3.22
9	0	0	44.35	3.45
-9	0	0	36.66	3.31
-9	0	0	41.32	3.15
9	0	-1	-0.04	1.15
9	0	-1	0.52	0.84
9	0	-1	0.44	0.61
-9	0	1	0.78	0.66
9	0	-2	5.38	1.12
9	0	-2	5.72	0.85
-9	0	2	5.33	0.82
9	0	-3	1.09	0.60
9	0	-3	-0.07	0.81
-9	0	3	0.22	0.56
9	0	-4	14.30	2.50
9	0	-4	17.68	1.59
-9	0	4	13.15	1.36
10	-1	3	2.43	0.95
10	-1	3	2.53	0.83
10	-1	3	1.09	0.75
10	-1	3	1.43	1.01
10	-1	2	3.49	0.79
10	-1	2	4.42	0.98
10	-1	2	3.28	0.89
10	-1	2	2.34	1.07
10	-1	1	4.17	1.17
10	-1	1	3.96	0.97
10	-1	1	2.91	0.79
10	-1	0	37.65	3.42
10	-1	0	27.26	2.68
10	-1	0	31.72	2.88
-10	1	0	36.07	2.77
10	-1	-1	4.13	1.10
10	-1	-1	2.65	0.78
-10	1	1	4.26	0.88
10	-1	-2	2.07	0.76
10	-1	-2	4.43	1.44
-10	1	2	3.03	0.86
10	-1	-3	1.96	0.72
-10	1	3	1.91	0.80
0	10	1	2.59	1.01
0	-10	-1	2.02	0.45
0	10	0	1.72	1.02
0	-10	0	1.31	0.42
0	10	-1	2.11	1.07
0	-10	1	2.67	0.49
0	-10	1	3.30	0.91
1	9	3	1.02	0.58
1	9	3	1.24	1.02
-1	-9	-3	0.75	0.41
1	9	2	1.39	0.61
1	9	2	1.00	0.63
1	9	2	1.10	0.99

-1	-9	-2	1.30	0.42
1	9	1	1.16	0.95
-1	-9	-1	0.99	0.43
1	9	0	11.66	1.96
-1	-9	0	10.77	1.06
1	9	-1	0.99	1.01
-1	-9	1	1.39	0.43
-1	-9	1	1.64	0.79
1	9	-2	1.23	1.02
-1	-9	2	1.27	0.75
-1	-9	2	1.04	0.40
1	9	-3	0.62	1.02
-1	-9	3	0.65	0.37
-1	-9	3	1.14	0.75
2	8	4	9.31	1.25
2	8	4	12.02	2.31
2	8	3	0.41	0.56
2	8	3	0.39	0.99
2	8	3	0.40	0.58
2	8	2	0.48	1.00
2	8	2	0.50	0.55
2	8	2	0.17	0.56
2	8	1	-0.05	1.04
2	8	1	-0.06	0.45
2	8	0	23.61	2.76
-2	-8	0	21.78	1.97
2	8	-1	0.18	0.98
-2	-8	1	0.33	0.67
2	8	-2	0.15	0.98
-2	-8	2	0.22	0.66
2	8	-3	0.02	1.04
-2	-8	3	0.17	0.65
2	8	-4	11.90	2.49
-2	-8	4	11.80	1.13
-2	-8	4	12.08	1.27
3	7	5	1.06	0.58
3	7	5	1.58	1.27
3	7	4	5.43	0.80
3	7	4	7.03	1.10
3	7	4	8.59	1.70
3	7	3	1.59	1.16
3	7	3	1.86	0.61
3	7	3	2.20	0.60
3	7	2	1.68	1.17
3	7	2	2.97	0.63
3	7	1	3.78	0.62
3	7	1	2.22	1.22
3	7	0	8.85	1.72
-3	-7	0	11.34	1.21
3	7	-1	2.67	1.31
-3	-7	1	3.91	0.77
3	7	-2	3.23	1.30
-3	-7	2	3.71	0.76
3	7	-3	1.41	1.20
-3	-7	3	2.38	0.67
3	7	-4	5.87	1.54
-3	-7	4	5.37	0.72
-3	-7	5	0.88	0.65
4	6	5	0.72	0.61
4	6	5	0.89	0.58
4	6	5	1.76	1.30
4	6	4	1.39	0.63
4	6	4	3.46	1.34
4	6	3	2.47	0.65
4	6	3	3.99	1.38
4	6	2	15.03	1.45
4	6	1	4.52	0.68
4	6	1	3.74	1.48
-4	-6	-1	4.21	0.62
4	6	0	0.39	1.13
4	6	-1	2.79	1.39
-4	-6	1	4.22	0.75
4	6	-2	13.99	2.62
-4	-6	2	15.71	1.57
4	6	-3	1.40	1.18
-4	-6	3	2.37	0.64
4	6	-4	1.40	1.24
-4	-6	4	1.41	0.48
4	6	-5	0.78	1.27
-4	-6	5	1.85	0.66
5	5	5	-0.03	0.62
5	5	5	1.45	1.23
5	5	4	2.61	1.33
5	5	4	0.70	0.62
5	5	3	-0.24	1.12
5	5	3	0.01	0.58
5	5	2	23.00	1.94
5	5	1	-0.35	0.45
5	5	1	-0.89	1.14
-5	-5	-1	0.26	0.40
-5	-5	-1	-0.15	0.37
5	5	0	1.17	0.52
5	5	0	0.77	1.29
-5	-5	0	-0.48	0.43
-5	-5	0	0.66	0.34
5	5	-1	-0.17	0.47
5	5	-1	-1.10	1.15
-5	-5	1	0.34	0.50
5	5	-2	19.42	1.77
5	5	-2	16.94	2.99
-5	-5	2	20.12	1.76
5	5	-3	0.02	0.50
-5	-5	3	-0.18	0.53
5	5	-4	-0.52	1.20
-5	-5	4	0.25	0.43
5	5	-5	1.00	1.13
-5	-5	5	0.71	0.64
6	4	5	2.61	1.30
6	4	5	1.17	0.70
6	4	4	1.45	0.70
6	4	4	5.80	1.42
6	4	3	2.98	0.75
6	4	2	13.61	1.55
6	4	2	16.30	1.55
6	4	1	6.84	1.07
6	4	1	11.77	2.34
6	4	1	5.91	0.96

6	4	0	4.85	1.54
6	4	0	2.53	0.72
-6	-4	0	2.73	0.59
-6	-4	0	1.68	0.50
6	4	-1	3.35	0.82
6	4	-1	6.77	1.74
-6	-4	1	4.95	0.63
6	4	-2	12.86	1.57
6	4	-2	18.06	2.98
-6	-4	2	14.26	1.47
6	4	-3	0.01	1.20
6	4	-3	1.25	0.70
-6	-4	3	2.29	0.61
6	4	-4	-0.36	1.15
6	4	-4	0.96	0.69
-6	-4	4	1.34	0.50
6	4	-5	-1.16	1.09
-6	-4	5	1.26	0.67
7	3	5	0.20	0.65
7	3	5	0.74	0.77
7	3	5	2.93	1.36
7	3	4	6.52	1.01
7	3	4	8.63	1.24
7	3	3	3.54	0.78
7	3	3	5.50	1.36
7	3	3	1.96	0.75
7	3	2	5.44	0.85
7	3	2	5.39	0.89
7	3	1	6.32	0.99
7	3	1	8.13	1.39
7	3	1	9.94	1.79
-7	-3	-1	10.90	1.57
7	3	0	21.72	2.00
7	3	0	18.54	1.87
-7	-3	0	22.66	1.90
-7	-3	0	20.78	1.84
7	3	-1	4.32	0.78
7	3	-1	8.08	1.87
7	3	-1	4.46	0.96
-7	-3	1	4.30	0.63
-7	-3	1	3.61	0.78
7	3	-2	3.92	0.72
7	3	-2	3.92	0.93
-7	-3	2	3.89	0.61
7	3	-3	1.84	1.46
7	3	-3	0.44	0.76
7	3	-3	1.30	0.62
-7	-3	3	1.65	0.57
7	3	-4	2.28	1.39
7	3	-4	4.21	0.99
-7	-3	4	5.23	0.66
-7	-3	5	0.40	0.70
8	2	4	30.77	3.25
8	2	4	25.06	2.35
8	2	3	0.36	1.08
8	2	3	0.76	0.66
8	2	3	0.32	0.74
8	2	2	1.19	0.72
8	2	2	0.86	0.69
8	2	2	0.38	0.65
8	2	2	0.90	1.06
8	2	1	0.40	0.73
8	2	1	0.12	0.62
8	2	1	1.14	1.20
8	2	0	62.12	4.49
8	2	0	51.12	4.30
-8	-2	0	62.51	4.34
-8	-2	0	60.15	4.23
8	2	-1	0.87	0.78
8	2	-1	2.13	0.66
8	2	-1	0.40	1.30
-8	-2	1	0.73	0.58
8	2	-2	-0.26	1.32
8	2	-2	0.18	0.76
8	2	-2	0.89	0.60
-8	-2	2	0.61	0.53
8	2	-3	-0.23	0.79
8	2	-3	-0.98	1.25
8	2	-3	0.77	0.61
-8	-2	3	0.32	0.48
8	2	-4	15.60	1.50
-8	-2	4	12.40	1.21
9	1	3	2.76	0.78
9	1	3	2.32	0.92
9	1	3	1.94	1.19
9	1	3	3.84	0.91
9	1	2	3.15	1.16
9	1	2	4.25	0.91
9	1	2	2.94	0.86
9	1	2	3.82	0.83
9	1	1	3.83	0.85
9	1	1	4.31	0.97
9	1	1	4.43	1.39
9	1	0	30.86	2.70
9	1	0	29.85	2.57
-9	-1	0	30.50	2.52
9	1	-1	3.49	1.00
9	1	-1	3.54	0.81
9	1	-1	5.13	1.62
-9	-1	1	3.52	0.80
9	1	-2	2.97	1.00
9	1	-2	2.90	1.88
9	1	-2	3.59	0.75
-9	-1	2	2.85	0.71
9	1	-3	1.94	0.66
-9	-1	3	1.68	0.72
10	0	1	5.85	0.97
10	0	1	6.14	1.45
10	0	0	7.53	1.58
10	0	0	4.71	0.95
-10	0	0	5.36	0.96
10	0	-1	5.80	0.95
-10	0	1	6.76	1.02
3	8	2	0.98	0.69
3	8	2	0.82	1.24
3	8	1	-0.16	1.14
3	8	0	4.89	1.53

3	8	-1	-0.53	1.13
-3	-8	1	0.49	0.70
3	8	-2	0.09	1.19
-3	-8	2	1.68	0.78
4	7	3	-0.28	1.23
4	7	3	-0.08	0.64
4	7	2	3.60	1.51
4	7	2	2.22	0.78
4	7	1	-0.73	1.21
4	7	0	4.79	1.65
-4	-7	0	7.44	1.07
-4	-7	1	0.12	0.66
4	7	-2	0.62	1.35
-4	-7	2	2.17	0.76
4	7	-3	-1.30	1.20
-4	-7	3	0.76	0.73
5	6	3	0.28	1.27
5	6	3	0.20	0.66
5	6	2	3.77	0.84
5	6	1	-0.13	1.33
5	6	0	6.39	1.83
-5	-6	0	4.82	0.67
5	6	-1	-0.86	1.37
5	6	-1	0.18	0.50
-5	-6	1	0.04	0.55
5	6	-2	1.30	1.42
-5	-6	2	3.54	0.79
5	6	-3	-1.12	1.26
-5	-6	3	0.34	0.67
6	5	3	-0.35	1.28
6	5	3	-0.34	0.70
6	5	2	4.32	0.83
6	5	1	0.32	1.34
6	5	0	9.94	2.05
-6	-5	0	8.88	1.19
6	5	-1	-0.74	0.57
6	5	-1	-1.08	1.36
-6	-5	1	0.10	0.44
6	5	-2	3.13	0.83
6	5	-2	4.81	1.82
-6	-5	2	3.82	0.72
6	5	-3	-0.62	0.58
-6	-5	3	0.08	0.66
7	4	3	0.24	1.26
7	4	3	-0.05	0.74
7	4	2	3.63	0.86
7	4	2	1.68	0.79
7	4	2	6.90	1.70
7	4	1	-0.14	1.29
7	4	0	18.63	3.13
7	4	0	9.31	1.57
-7	-4	0	13.11	1.40
7	4	-1	-0.90	0.71
7	4	-1	-0.93	1.50
-7	-4	1	0.49	0.52
7	4	-2	4.03	1.77
7	4	-2	1.51	0.77
-7	-4	2	1.97	0.54
7	4	-3	-2.09	1.23
-7	-4	3	-0.02	0.60
8	3	2	2.73	0.81
8	3	2	4.20	1.48
8	3	1	0.16	0.68
8	3	1	1.88	1.39
8	3	1	0.84	0.78
8	3	0	11.29	1.65
8	3	0	15.76	1.86
-8	-3	0	16.50	1.66
8	3	-1	0.25	1.46
8	3	-1	-0.03	0.79
8	3	-1	0.83	0.66
-8	-3	1	1.07	0.62
8	3	-2	1.73	0.73
8	3	-2	4.10	1.87
-8	-3	2	1.78	0.62
0	0	0	0.00	0.00

;

_shelx_hkl_checksum 85585
